# Supplementary material for: Nickel/photoredox dual catalyzed arylalkylation of nonactivated alkenes
Source: Nat Commun. 2023 Nov 30;14:7917. doi: 10.1038/s41467-023-43748-4 (PMC10689762; doi:10.1038/s41467-023-43748-4)

Supplementary Information

Nickel/Photoredox Dual Catalyzed Arylalkylation of Nonactivated Alkenes

Yuxi Gao, Lijuan Gao, Endiao Zhu, Yunhong Yang, Mi Jie, Jiaqian Zhang, Zhiqiang Pan,* and Chengfeng Xia*

Key Laboratory of Medicinal Chemistry for Natural Resource, Ministry of Education, Yunnan Key Laboratory of Research and Development of Natural Products, School of Pharmacy, Yunnan University, Kunming, Yunnan 650500, China

Table of Contents

Supplementary Methods

General information S2
Experimental setup for dual catalyzed arylalkylation S3
General procedure A for the preparation of nonactivated alkenes S4
General procedure B for the preparation of nonactivated alkenes S4
General procedure C for the preparation of nonactivated alkenes S5
General procedure D for the preparation of nonactivated alkenes S5
General procedure E for the nickel/photoredox dual catalyzed arylalkylation S6
General procedure F for the asymmetric arylalkylation..S7
Supplementary Note 1. Reaction optimization for enantioselective synthesis........................S8
Supplementary Note 2. Mechanistic investigation..S12
Supplementary Note 3. Cyclic voltammentry...S14
Supplementary Note 4. Stern-Volmer experiments...S17
Supplementary Note 5. Light-dark interval experiments S19
Supplementary Note 6. Identification of unactivated alkenes..S21
Supplementary Note 7. Identification of arylalkylation compounds....................................S28
Supplementary Note 8. Identification of enatioselective arylalkylation compounds............S46
Supplementary Note 9. Synthesis of Melatonin receptor agonist (55).................................S58
Supplementary Note 10. Synthesis of S20242 (57) S61
Supplementary Note 11. Gram-scale synthesis of compound 3...S64
Supplementary Note 12. Unsuccessful examples S65

NMR spectra..S66

General information

Unless otherwise noted, materials were purchased from commercial suppliers and used without further purification. All solvents were treated according to general methods. Thin layer chromatography was carried out a GF254 plates (0.25 mm layer thickness). Flash column chromatography with was performed using 200-300 mesh silica gel. All reactions were carried out in flame-dried glass tube under a dry argon atmosphere, glass tube was dried in an oven at 150 °C or flame dried and cooled under a dry atmosphere. Reactions were monitored by TLC and visualized by a dual short wave/long wave UV lamp. Photochemical reactions were carried with two 18 W LEDs (455 nm) from a distance of 8 cm. Fans are employed for maintaining the reaction temperature at 60 °C. ^{1}H NMR spectra recorded on Bruker 600 (600 MHz), Bruker 500 (500 MHz), and Bruker 400 (400 MHz) spectrophotometers. Chemical shifts (δ) are reported in ppm from the solvent resonance as the internal standard ($CDCl_3$; 7.26 ppm). Data are reported as follows: chemical shift, multiplicity (s = single, d = doublet, t = triplet, dd = doublet of doublets, m = multiplet or unresolved, br = broad, q = quartet, coupling constant (s) in Hz, integration). ^{13}C NMR spectra were recorded on Bruker 600 (150 MHz), Bruker 500 (125 MHz), and Bruker 400 (100 MHz) with complete proton decoupling spectrophotometers ($CDCl_3$; 77.16 ppm). ^{19}F NMR spectra were recorded on Bruker 600 (565 MHz) and Bruker 500 (471MHz) Bruker 400 (376 MHz) spectrophotometers. Structural assignments were made with additional information from ^{1}H-^{1}H COSY, HSQC, HMBC and ROSEY experiments. HRMS (ESI) were taken on Agilent 6540 Q-TOF spectrometer. HRMS (EI) were taken on Thermo Fisher Scientific DFS spectrometer. HPLC were taken on Agilent 1260 II Infinity Liquid chromatography. The emission spectra were recorded on a Fluorescence Spectrophotometer F-7000.

Experimental setup for dual catalyzed arylalkylation

A 10 mL oven dried glass tube equipped with a tiring bar and filled with argon, the stirred reaction mixture is irradiated with two parallel 18 W LEDs (455 nm,) from a distance of approximate 8 cm, fans are employed for maintaining the reaction temperature at 60 °C.

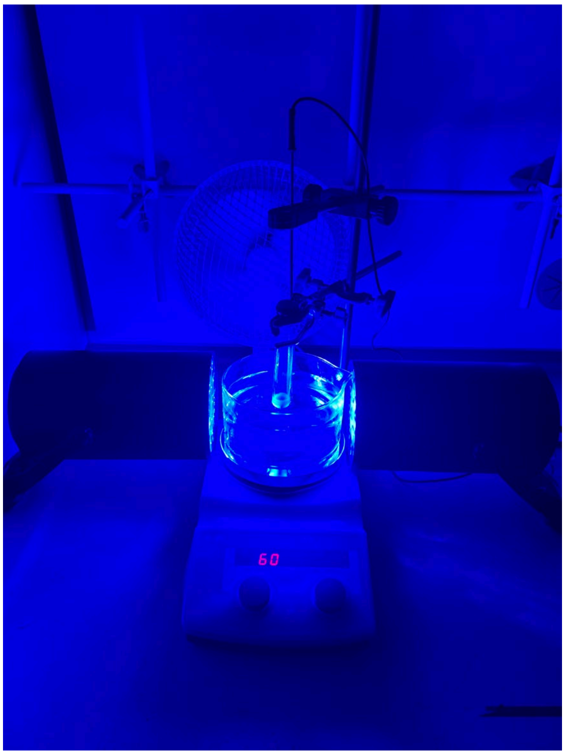

Supplementary Figure 1. Experimental setup

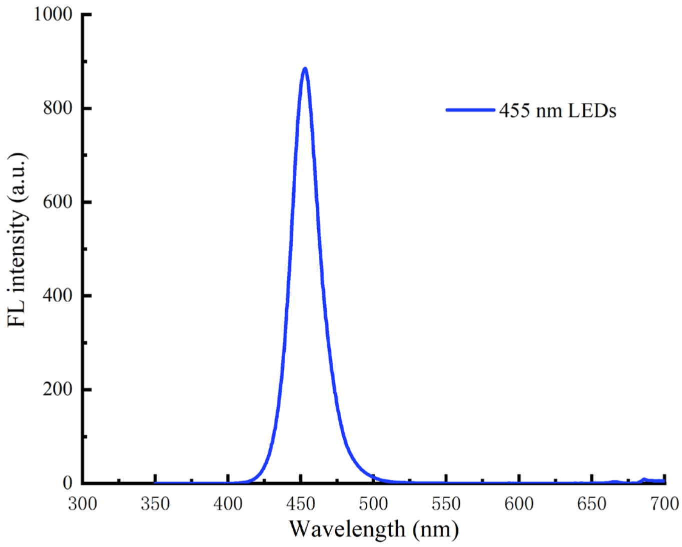

Supplementary Figure 2. Emission spectra of the 18 W blue LEDs

General procedure A for the preparation of nonactivated alkenes

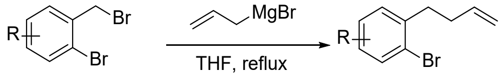

In a flame-dried 100 mL round-bottom flask, equipped with a magnetic stir bar and rubber septum under nitrogen, was placed Benzyl bromide (8.0 mmol, 1.0 equiv.) and anhydrous THF (30 mL). The flask was placed in an ice water bath and allylmagnesium bromide (1.0 M in diethyl ether, 12.0 mmol, 1.5 equiv) was added dropwise via syringe. The reaction vessel was then fitted with a reflux condenser and heated to reflux temperature in an oil bath overnight. The reaction was then cooled to 0 °C and quenched by the addition of saturated NH_4Cl solution. The resulting solution was extracted with EtOAc (3 x 20 mL). The combined organic layers were dried over Na_2SO_4, filtered, and evaporated. The residue was purified by silica gel column chromatography.

General procedure B for the preparation of nonactivated alkenes

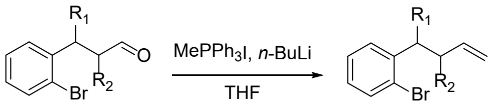

In a flame-dried 100 mL round-bottom flask, was placed 3-(2-bromophenyl)-2,2-dimethylpropanal (6.6 mmol, 1.1 equiv.) and THF (30 mL). *n*-BuLi (6.6 mmol, 1.1 equiv.) was added at 0 °C for half an hour, followed by room temperature for an hour. The flask was placed in an ice water bath and 3-(2-bromophenyl)-2,2-dimethylpropanal (dissolved in 10 mL THF) was added, two hours later the reaction was carried out in ice bath and at room temperature overnight. The reaction was quenched by the addition of water. The resulting solution was extracted with EtOAc (3 x 20 mL). The combined organic layers were dried over Na_2SO_4, filtered, and evaporated. The residue was purified by silica gel column chromatography.

General procedure C for the preparation of nonactivated alkenes

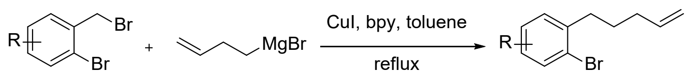

In a flame-dried 100 mL round-bottom flask, was placed Benzyl bromide (8.0 mmol, 1.0 equiv), CuI (0.80 mmol, 0.1 equiv) and 2,2'-bipyridyl (0.80 mmol, 0.1 equiv) in anhydrous toluene (10 mL) at 0 °C. The Grignard solution was then slowly added to this mixture, then allowed to warm to room temperature and stirred for an additional 2 h when TLC indicated the reaction was complete. The reaction was quenched by the addition of saturated NH_4Cl solution. The resulting solution was extracted with EtOAc (3 x 20 mL). The combined organic layers were dried over Na_2SO_4, filtered, and evaporated. The residue was purified by silica gel column chromatography to give the product.

General procedure D for the preparation of nonactivated alkenes

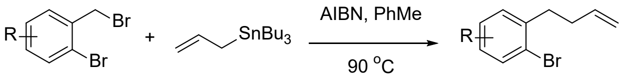

In a flame-dried 100 mL round-bottom flask, was placed Benzyl bromide (2.0 mmol, 1.0 equiv.), AIBN (0.40 mmol, 0.2 equiv.) in anhydrous toluene (25.0 mL) under nitrogen. The allyltributyltin (3.0 mmol, 1.5 equiv.) was then added to this mixture The reaction vessel was then heated to 90 °C in an oil bath overnight when TLC indicated the reaction was complete. The solvent was evaporated and concentrated, the residue was purified by silica chromatography.

General procedure E for the nickel/photoredox dual catalyzed arylalkylation

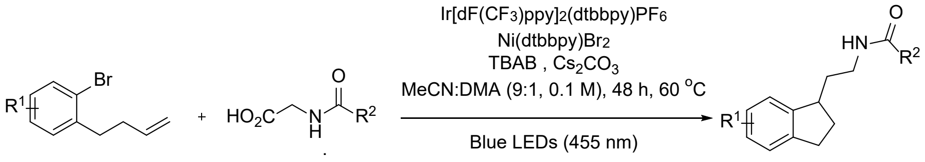

To a 10 mL glass tube equipped with a septum and a magnetic stir bar was added Ni(dtbbpy)Br_2 (19.5 mg, 0.04 mmol, 20 mol%), Amino acid derivatives (0.40 mmol, 2.0 equiv.), TBAB (16.1 mg, 0.05 mmol, 25 mol%), Cs_2CO_3 (108 mg, 0.33 mmol, 1.65 equiv.), Ir[dF(CF_3)ppy]$_2$(dtbbpy)PF_6 (4.5 mg, 0.004 mmol, 2.0 mol%) and MeCN (1.8 mL) and DMA (0.2 mL) in the glove box. The corresponding unactivated alkenes (0.20 mmol, 1.0 equiv.) was added to the glass tube with a pipette gun under the argon. The resulting mixture was then sealed and wrapped with electrical tape and then irradiated with two parallel 18 W LEDs (455 nm,) from a distance of approximate 8 cm for 48 h. The reaction was maintained at 60 °C by heating in an oil bath and cooling by a fan. Then, the solvent was evaporated and concentrated, the residue was purified by silica chromatography.

General procedure F for the asymmetric arylalkylation

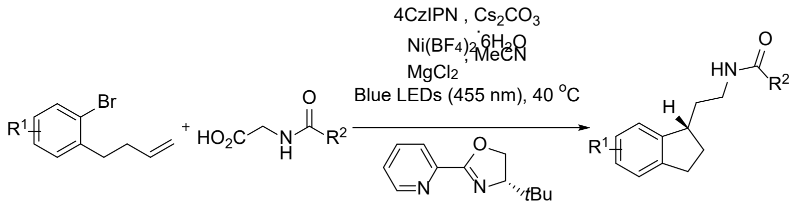

To a 10 mL glass tube equipped with a septum and a magnetic stir bar was added $Ni(BF_4)_2{\cdot}6H_2O$ (13.6 mg, 0.04 mmol, 20 mol%), Ligand (9.0 mg, 0.044 mmol, 22 mol%) and MeCN (2.0 mL) in the glove box. The mixture was stirred at room temperature for 30 min. Amino acid derivatives (0.30 mmol, 1.5 equiv.), $MgCl_2$ (4.8 mg, 0.05 mmol, 25 mol%), Cs_2CO_3 (108 mg, 0.33 mmol, 1.65 equiv.), 4CzIPN (3.2 mg, 0.004 mmol, 2 mol%) and the corresponding unactivated alkenes (0.20 mmol, 1.0 equiv.) were then added in sequence under the argon. The resulting mixture was then sealed and wrapped with electrical tape and removed from the glove box. The reaction mixture was irradiated with two parallel 18 W LEDs (455 nm,) from a distance of approximate 8 cm for 48 h. The reaction was maintained at 40 °C by heating in an oil bath and cooling by a fan. Then, the solvent was evaporated and concentrated, the residue was purified by silica chromatography.

Supplementary Note 1. Reaction optimization for enantioselective synthesis

Supplementary Table 1. Evaluation of chiral ligands.

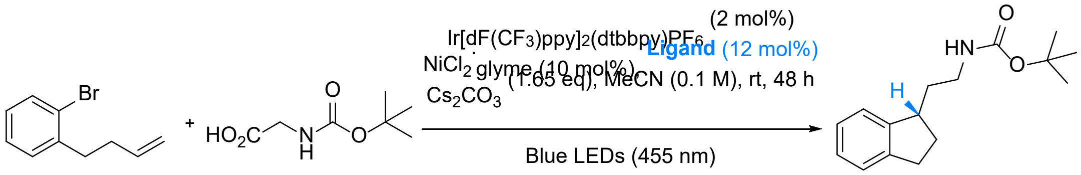

1 + 2 (1.5 eq) → (*S*)-**39**

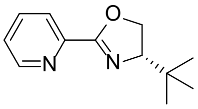

L1
14%, 89:11 e.r.

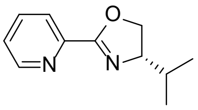

L2
13%, 55:45 e.r.

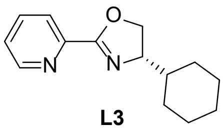

L3
19%, 78:22 e.r.

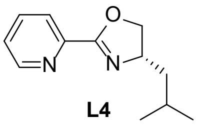

L4
17%, 80:20 e.r.

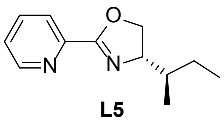

L5
23%, 76:24 e.r.

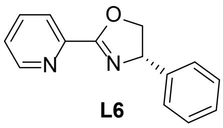

L6
8%, 85:15 e.r.

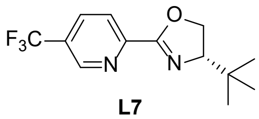

L7
8%, 82:18 e.r.

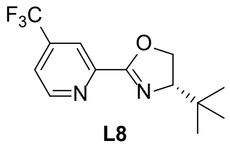

L8
7%, 76:24 e.r.

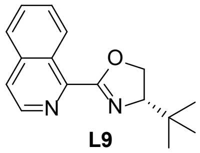

L9
4%, 86:14 e.r.

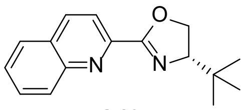

L10
12%, 60:40 e.r.

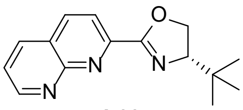

L11
14%, 55:45 e.r.

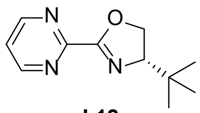

L12
17%, 85:15 e.r.

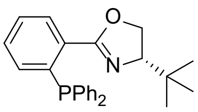

L13
n.r.

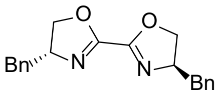

L14
29%, 57:43 e.r.

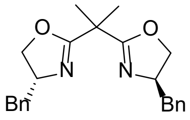

L15
8%, 69:31 e.r.

Supplementary Table 2. Evaluation of solvents.

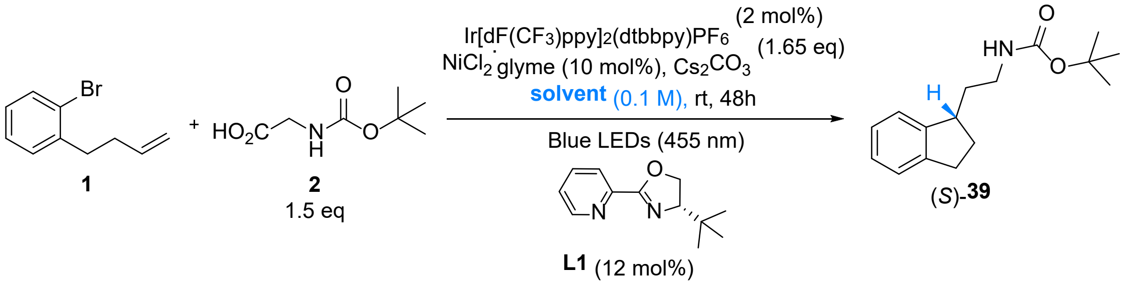

Entry	Solvent	Yeild (%)	e.r.
1	PhMe	6	72:28
2	THF	12	75:25
3	DCE	trace	--
4	DCM	trace	--
5	DME	11	63:37
6	1,4-dioxane	10	76:24
7	MeCN	14	89:11
8	acetone	6	60:40
9	DMSO	31	58:42
10	DMA	14	50:50
11	DMF	18	58:42
12	PhCN	trace	--
13	CH_3NO_2	trace	--
14	$PhCF_3$	6	76:24

Supplementary Table 3. Evaluation of nickel catalysts.

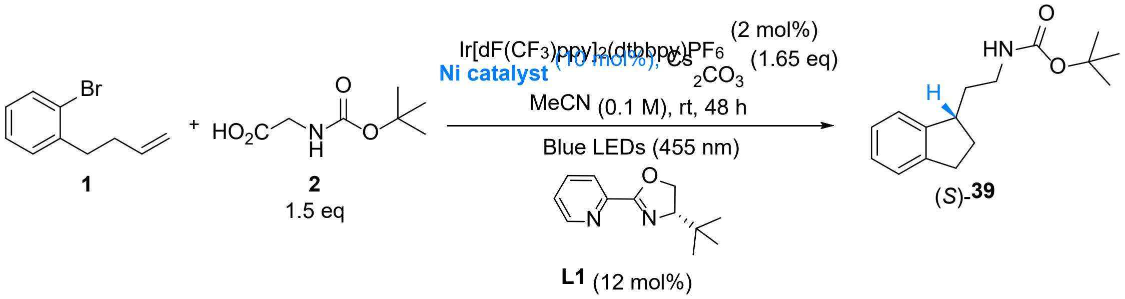

Entry	Ni Source	Yeild (%)	e.r.
1	$NiBr_2$	13	68:32
2	$NiBr_2$·glyme	12	85:15
3	$Ni(BF_4)_2$·$6H_2O$	15	91:9
4	$Ni(COD)_2$	17	82:18
5	$Ni(OTf)_2$	16	90:10
6	$Ni(OAc)_2$	10	82:18
7	$Ni(acac)_2$	16	76:24

Supplementary Table 4. Evaluation of photocatalysts.

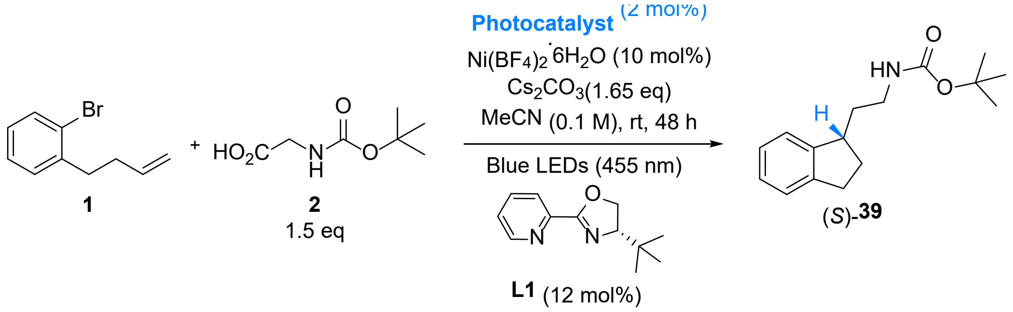

Entry	Photocatalyst	Yeild (%)	e.r.
1	Ir(dmppy)$_2$(dtbbpy)PF$_6$	n.r.	--
2	*fac*-Ir(ppy)$_3$	n.r.	--
3	Ir(dtbbpy)(ppy)$_2$PF$_6$	n.r.	--
4	Ir(dtbppy)$_2$(dtbbpy)PF$_6$	16	80:20
5	4CzIPN	25	93:7
6	4DPAIPN	n.r.	--
7	Ir[dF(CF$_3$)ppy]$_2$(dtbbpy)PF$_6$	15	91:9

Supplementary Table 5. Evaluation of equivalent of nickel catalysts and ligands.

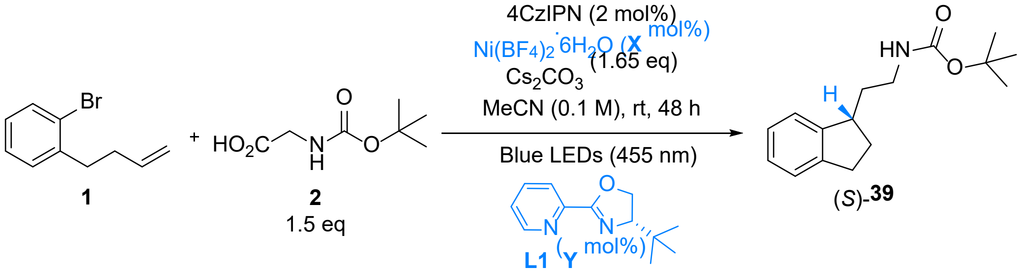

Entry	Ni(BF$_4$)$_2$·6H$_2$O (X mol%)	Ligand (Y mol%)	Yeild (%)	e.r.
1	10	12	25	93:7
2	15	16.5	42	94:6
3	15	30	38	95:5
4	20	22	60	95:5
5	20	30	32	95:5
6	25	27.5	40	95:5
7	30	33	35	94:6

Supplementary Table 6. Evaluation of temperature.

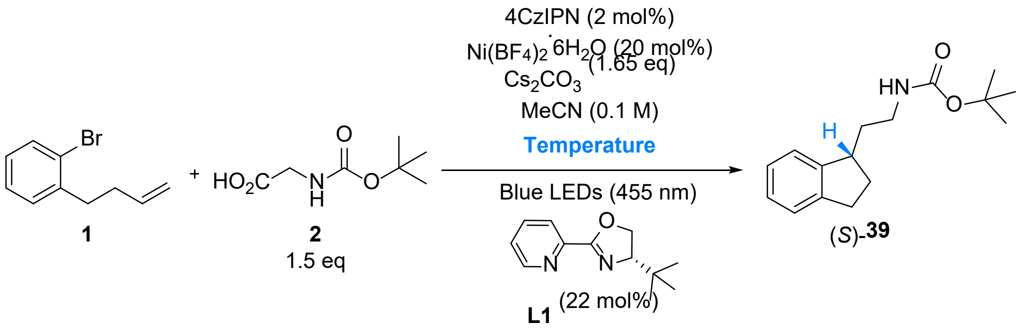

Entry	Temperature	Yeild (%)	e.r.
1	rt, 48 h	60	95:5
2	0 °C, 5d	40	93:7
3	40 °C, 48 h	85	95:5
4	50 °C, 48 h	65	95:5

Supplementary Table 7. Evaluation of additive for Enantioselective Synthesis of (*S*)-**3**.

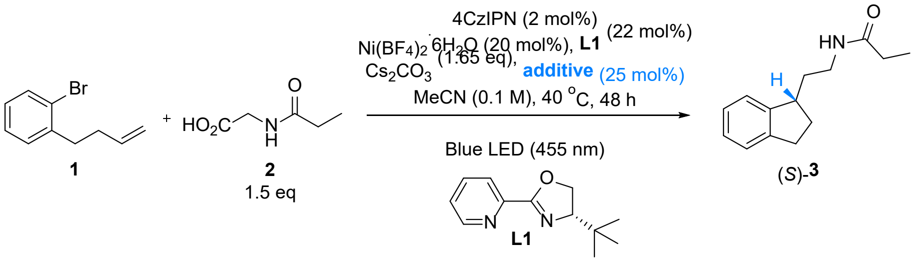

Entry	Addictive	Yeild (%)	ee (%)
1	none	22	95:5
2	$MgCl_2$, 0.25 eq	55	95:5
3	TBAB, 0.25 eq	33	95:5
4	NaI	n.r.	--
5	B_2Pin_2	n.r.	--
6	3 Å MS	29	93:7
7	4 Å MS	23	95:5
8	5 Å MS	19	95:5

Supplementary Note 2. Mechanistic investigation

Radical trapping experiments

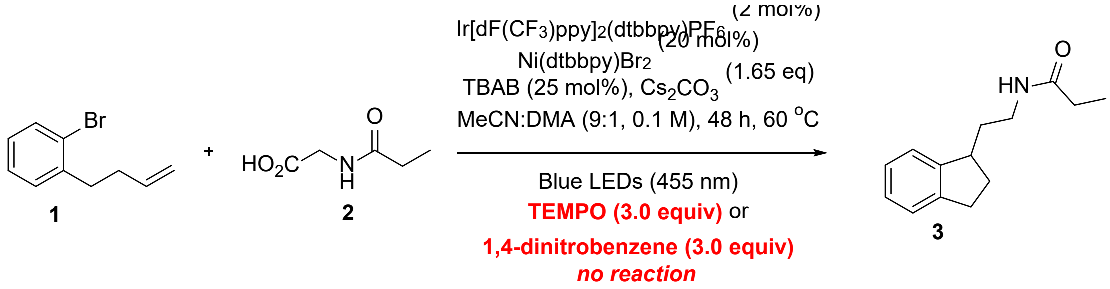

To an 10 mL glass tube equipped with a septum and a magnetic stir bar was added $Ni(dtbbpy)Br_2$ (19.5 mg, 0.04 mmol, 20 mol%), Amino acid **2** (52.0 mg, 0.40 mmol, 2.0 equiv.), TBAB (16.1 mg, 0.05 mmol, 25 mol%), Cs_2CO_3 (108.0 mg, 0.33 mmol, 1.65 equiv.), $Ir[dF(CF_3)ppy]_2(dtbbpy)PF_6$ (4.5 mg, 0.004 mmol, 2.0 mol%), TEMPO (94.0 mg, 0.6 mmol, 3.0 equiv.) or 1,4-dinitrobenzene (100.0 mg, 0.6 mmol, 3.0 equiv.) and MeCN (1.8 mL) and DMA (0.2 mL) in the glove box. The unactivated alkene **1** (42.0 mg, 0.20 mmol, 1.0 equiv.) was added to the glass tube with a pipette gun under the argon. The resulting mixture was then sealed and wrapped with electrical tape and then irradiated with two parallel 18 W LEDs (455 nm,) from a distance of approximate 8 cm for 48 h. The reaction was maintained at 60 °C by heating in an oil bath and cooling by a fan. Then, the solvent was evaporated and concentrated, the residue was purified by silica chromatography to afford compound **1** (40 mg, 0.19 mmol). The product **3** was not obtained.

In the presence of TEMPO (2,2,6,6-tetramethyl-1-piperidinyloxy) or 1,4-dinitrobenzene, the dual catalytic arylalkylation was strongly inhibited, which points towards a radical mechanism.

Formation of product 19 and protonated byproduct 60

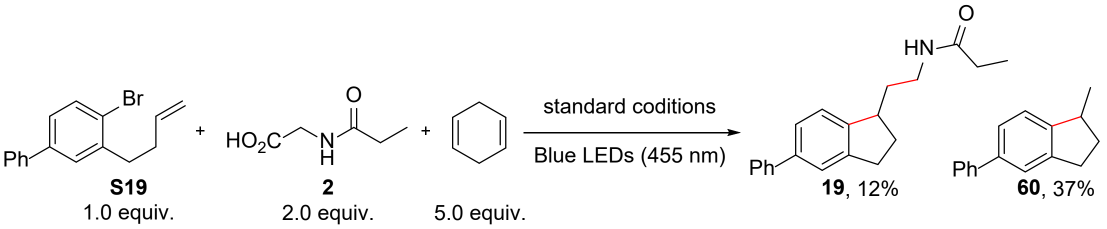

To a 10 mL glass tube equipped with a septum and a magnetic stir bar was added Ni(dtbbpy)Br_2 (19.5 mg, 0.04 mmol, 20 mol%), Amino acid derivative **2** (52.0 mg, 0.40 mmol, 2.0 equiv.), TBAB (16.1 mg, 0.05 mmol, 25 mol%), Cs_2CO_3 (108 mg, 0.33 mmol, 1.65 equiv.), Ir[dF(CF_3)ppy]$_2$(dtbbpy)PF_6 (4.5 mg, 0.004 mmol, 2.0 mol%) and MeCN (1.8 mL) and DMA (0.2 mL) in the glove box. The unactivated alkene **S19** (57.0 mg, 0.20 mmol, 1.0 equiv.) and 1,4-cyclohexadiene (80.0 mg, 1.0 mmol, 5.0 equiv.) was added to the glass tube with a pipette gun under the argon. The resulting mixture was then sealed and wrapped with electrical tape and then irradiated with two parallel 18 W LEDs (455 nm,) from a distance of approximate 8 cm for 48 h. The reaction was maintained at 60 ^{o}C by heating in an oil bath and cooling by a fan. Then, the solvent was evaporated and concentrated, the residue was purified by silica chromatography to afford **19** (7 mg, 12% yield) and **60** (16 mg, 38% yield).

1-methyl-5-phenyl-2,3-dihydro-1*H*-indene (60, known)

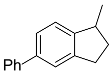

60 was purified as colorless oil (16 mg, 38% yield) by flash column chromatography (petroleum ether). The ^{1}H NMR and ^{13}C NMR are consistent with that reported in *Org. Lett.* **2022**, *24*, 4281.
Analytical data: ^{1}H NMR (400 MHz, $CDCl_3$) δ 7.58 (d, J = 7.6 Hz, 2H), 7.42 (t, J = 8.3 Hz, 4H), 7.32 (t, J = 7.3 Hz, 1H), 7.26 (d, J = 7.6 Hz, 1H), 3.28 – 3.19 (m, 1H), 3.01 – 2.86 (m, 2H), 2.40 – 2.32 (m, 1H), 1.71 – 1.61 (m, 1H), 1.33 (d, J = 6.8 Hz, 3H). ^{13}C NMR (100 MHz, $CDCl_3$) δ 148.2, 144.8, 141.9, 139.7, 128.8, 127.3, 127.0, 125.5, 123.6, 123.4, 39.3, 35.1, 31.6, 20.0.

Supplementary Note 3. Cyclic voltammentry

Tetrabutylammonium hexafluorophosphate (116.1 mg, 0.30 mmol) was added to a 0.01 M solution of the **1** in 3.0 mL of dry MeCN and the solution was vigorously bubbled with N_2 for 5 minutes prior to the measurement. The oxidation potential was measured using a glassy carbon working electrode, a platinum wire counter electrode, and a saturated calomel electrode (SCE) at 0.1 V/s scan rate. A completely irreversiblereduction wave was observed with E_p = 2.37 V versus SCE in MeCN.

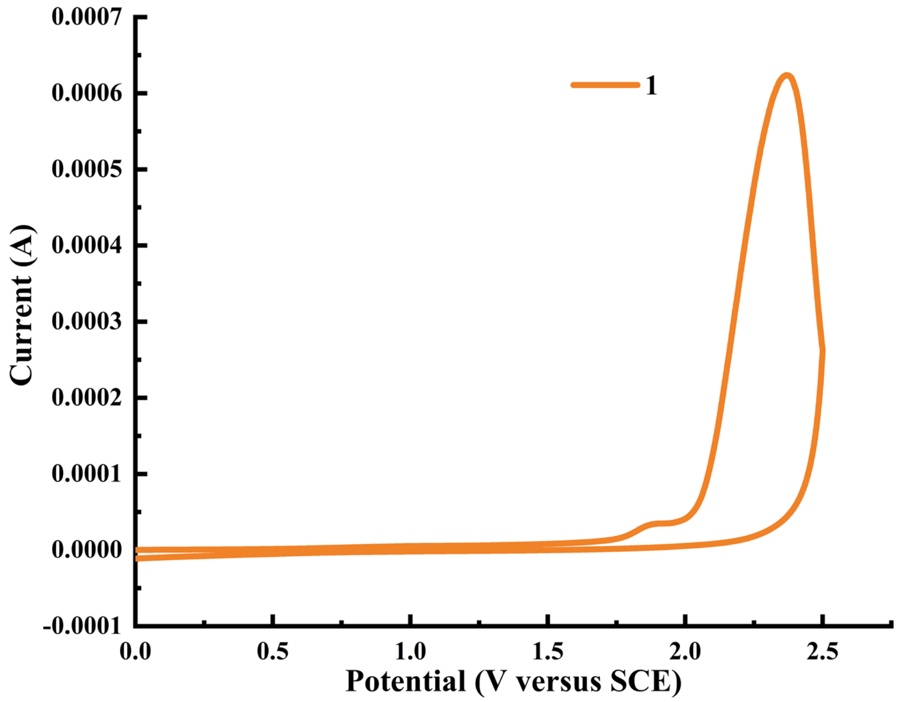

Supplementary Figure 3. Cyclic voltammogram of the alkene **1** versus SCE in MeCN at 0.1 V/s.

Tetrabutylammonium hexafluorophosphate (116.1 mg, 0.30 mmol) was added to a 0.01 M solution of the carboxylic anion of **2** (generated in situ by the deprotonation of **2** with 1.5 equiv Cs_2CO_3) in 3.0 mL of dry DMSO and the solution was vigorously bubbled with N_2 for 5 minutes prior to the measurement. The oxidation potential was measured using a glassy carbon working electrode, a platinum wire counter electrode, and a saturated calomel electrode (SCE) at 0.1 V/s scan rate. A completely irreversible reduction wave was observed with $E_{p/2}$(**2**$^-$/**2**$^\cdot$) = +0.98 V versus SCE in DMSO.

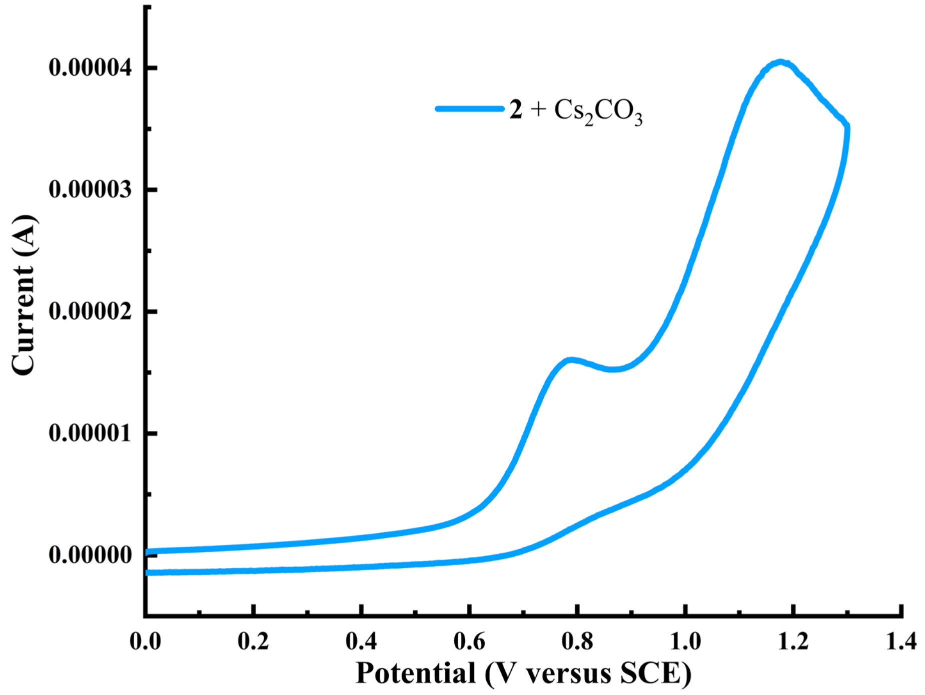

Supplementary Figure 4. Cyclic voltammogram of the carboxylic anion of **2** versus SCE in DMSO at 0.1 V/s.

General procedure: A 10 mM solution of catalyst was prepared in MeCN with 0.1 M tetrabutylammonium hexafluorophosphate electrolyte. Scan rate was set at 0.2 V/s. Reference electrode was Ag wire in 0.1 M AgCl. $E_{1/2}^{red}$ ([Ir^{III}/Ir^{II}] = –1.30 V versus Ag/AgCl in MeCN. $E_{p/2}$ [Ni^{I}/Ni^{0}] = –1.29 V versus Ag/AgCl in MeCN. $E_{p/2}$ [Ni^{II}/Ni^{I}] = –1.07 V versus Ag/AgCl in MeCN.

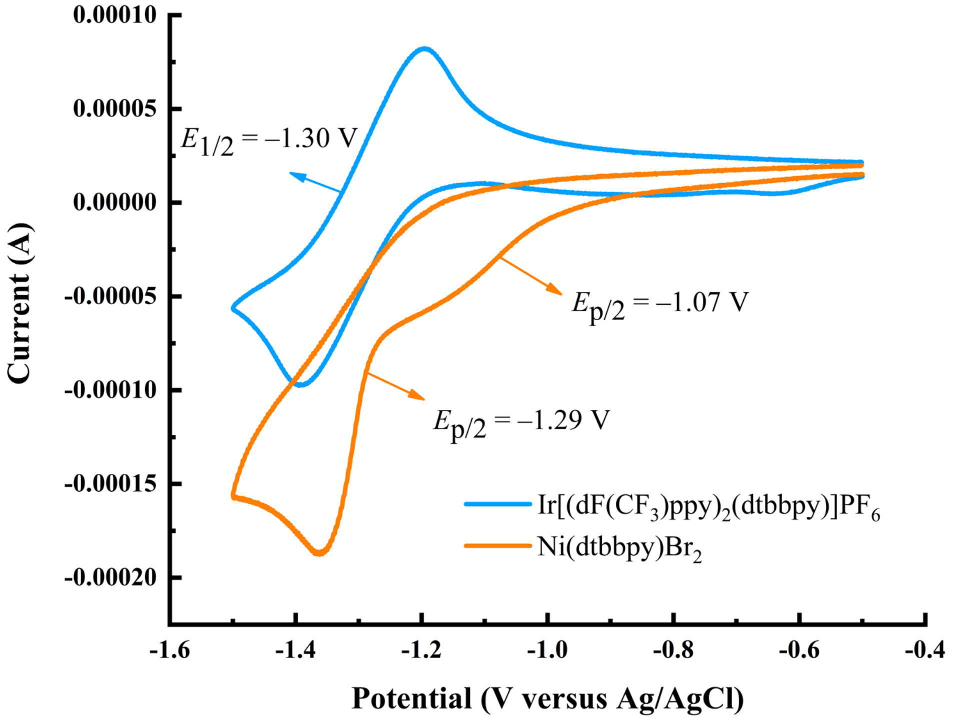

Supplementary Figure 5. Cyclic voltammogram of $Ir[(dF(CF_3)ppy)_2(dtbbpy)]PF_6$ and $Ni(dtbbpy)Br_2$ (taken separately).

Supplementary Note 4. Stern-Volmer experiments

The samples were prepared mixing the photocatalyst of Ir[dF(CF_3)ppy]$_2$(dtbbpy)PF_6 of (5 × 10^{-5} M with the required amount of **2**, freshly prepared in situ by the deprotonation of **2** with 1.5 equiv Cs_2CO_3) in a total volume of 1 mL of dry DMSO (rigorously degassed by freeze/pump/thaw) (DMSO was used as solvent to increase the solubility of **2**) in a 10 × 10 mm light path quartz fluorescence cuvette under an argon atmosphere. The samples were vigorously bubbled with dry argon for 5 minutes prior to the measurement. The excitation wavelength was fixed at 375 nm, the emission light was acquired from 395 nm to 900 nm.

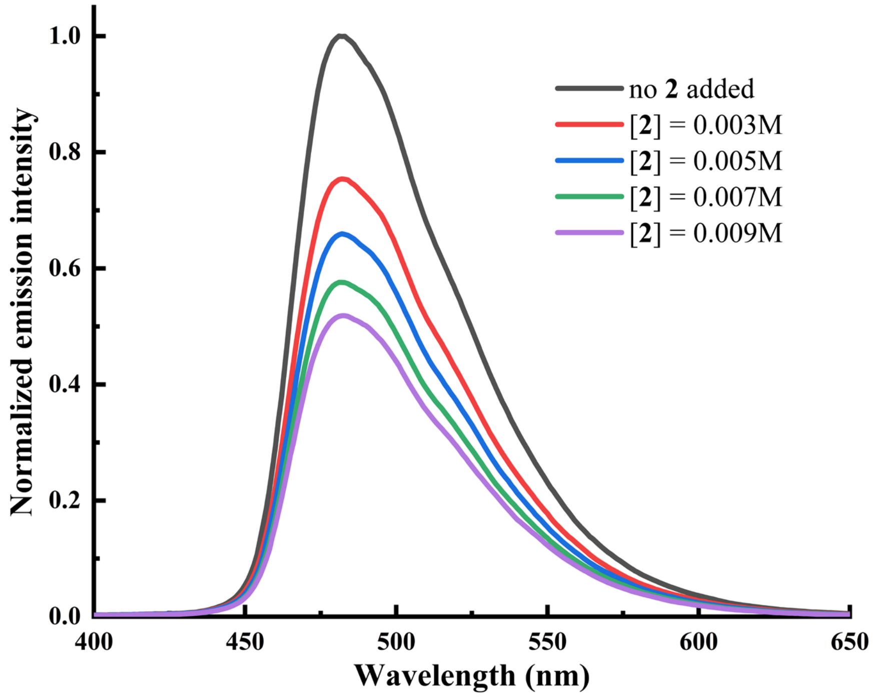

Supplementary Figure 6. Quenching of the photocatalyst of Ir[dF(CF_3)ppy]$_2$(dtbbpy)PF_6 emission (5 × 10^{-5} M in DMSO) in the presence of increasing amounts of **2**

The Stern-Volmer plot shows a linear correlation between the amounts of **2** and the ratio I_0/I.

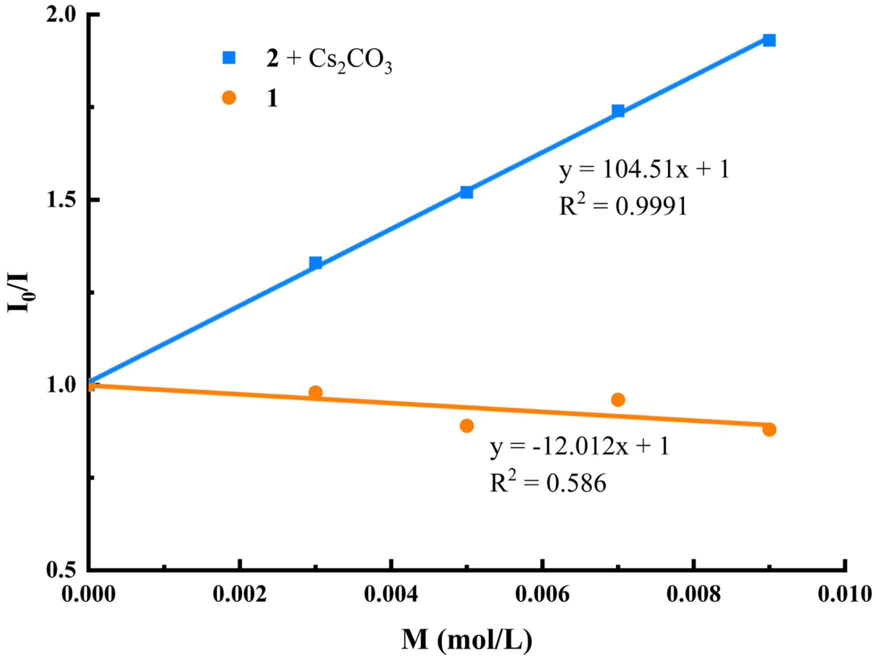

Supplementary Figure 7. Stern-Volmer quenching plot

Supplementary Note 5. Light-dark interval experiments

Following the general procedure E, the photochemical reaction was conducted for light-dark interval experiment. Aliquots of samples were taken out at various time points during the reaction. The crude NMR was taken on the concentrated crude reaction mixture and analyzed by ^{1}H NMR using dimethyl terephthalate as an internal standard.

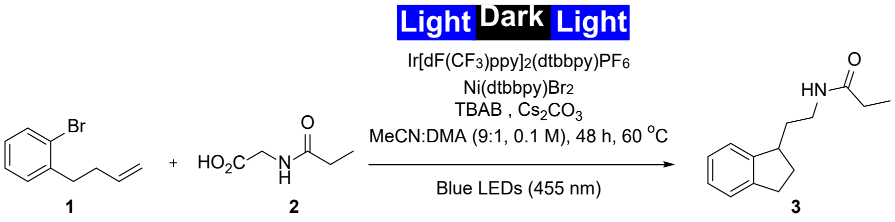

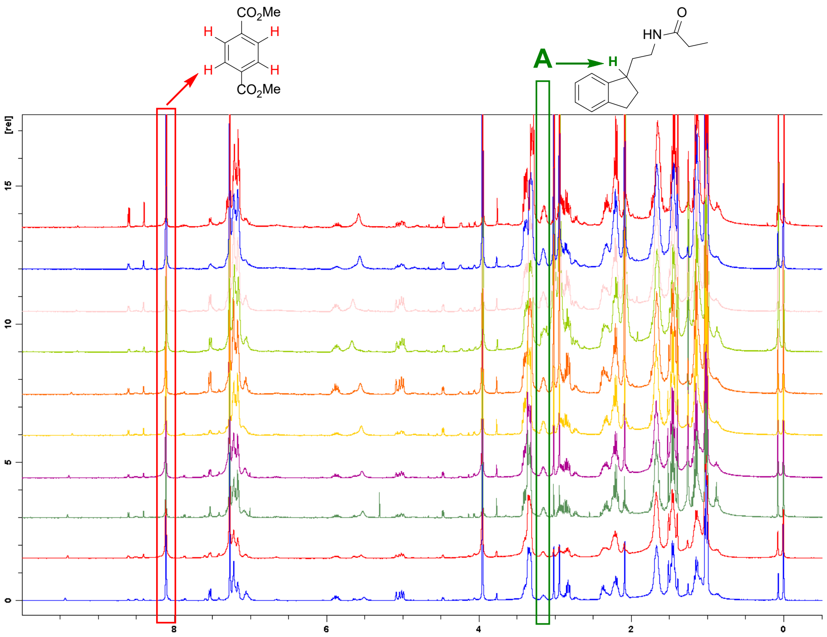

Supplementary Figure 8. From the bottom to top, ^{1}H NMR (400 MHz, in $CDCl_3$) spectra of **3** (as reference) and the crude product from the light-dark interval experiments. Yields were inferred by area integration ratio (signals A).

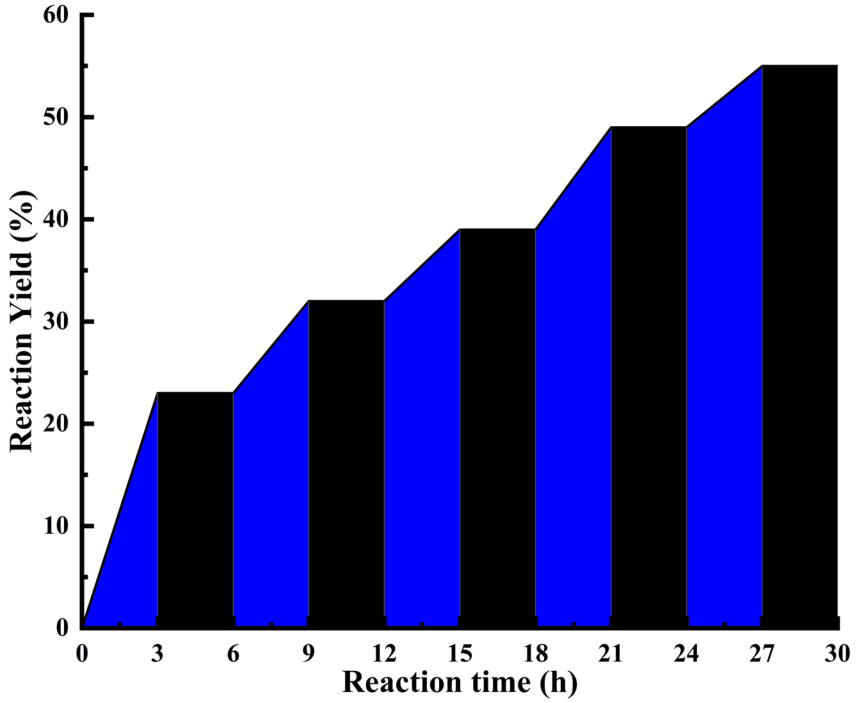

Supplementary Figure 9. Light dark interval experiment of compound **3** yield changing with time.

Supplementary Note 6. Identification of unactivated alkenes

1-bromo-2-(but-3-en-1-yl)benzene (1)

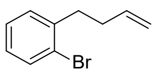

Following the general procedure **A**, **1** was purified as colorless liquid (1.51 g, 90% yield) by flash column chromatography (petroleum ether). Analytical data: ^{1}H NMR (400 MHz, $CDCl_3$) δ 7.58 (d, J = 7.7 Hz, 1H), 7.29 – 7.26 (m, 2H), 7.12 – 7.08 (m, 1H), 5.98 – 5.88 (m, 1H), 5.14 – 5.04 (m, 2H), 2.88 (t, J=7.64, 2H), 2.45 – 2.40 (m, 2H). ^{13}C NMR (100 MHz, $CDCl_3$) δ 141.2, 137.8, 132.9, 130.5, 127.7, 127.4, 124.6, 115.3, 35.7, 34.0. HRMS (ESI) calcd for $C_{10}H_{12}Br$ $[M + H]^+$: 211.0117, Found: 211.0118.

2-bromo-1-(but-3-en-1-yl)-4-fluorobenzene (S4)

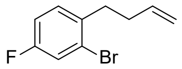

Following the general procedure **A**, **S4** was purified as colorless liquid (0.81 g, 95% yield) by flash column chromatography (petroleum ether). Analytical data: ^{1}H NMR (500 MHz, $CDCl_3$) δ 7.29 – 7.26 (m, 1H), 7.16 (t, J = 7.5 Hz, 1H), 6.95 (t, J = 8.4 Hz, 1H), 5.89 – 5.81 (m, 1H), 5.05 (d, J = 17.1 Hz, 1H), 5.00 (d, J = 10.2 Hz, 1H), 2.79 (t, J = 7.9 Hz, 2H), 2.34 (dt, J = 7.6, 7.5 Hz, 2H). ^{13}C NMR (125 MHz, $CDCl_3$) δ 160.8 (d, J = 248.3 Hz), 137.4, 136.9 (d, J = 3.6 Hz), 130.9 (d, J = 8.2 Hz), 124.1 (d, J = 9.2 Hz), 119.8 (d, J = 24.1 Hz), 115.4, 114.4 (d, J = 20.6 Hz), 34.8, 33.9. ^{19}F NMR (471 MHz, $CDCl_3$) δ -115.4. HRMS (HREI) calcd for $C_{10}H_{10}BrF$ [M]: 227.9944, Found: 227.9944.

1-bromo-2-(but-3-en-1-yl)-4-fluorobenzene (S5)

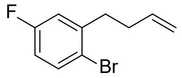

Following the general procedure **A**, **S5** was purified as colorless liquid (0.71 g, 85% yield) by flash column chromatography (petroleum ether). Analytical data: ^{1}H NMR (500 MHz, $CDCl_3$) δ 7.48 (t, J = 7.5 Hz, 1H), 6.94 (d, J = 9.0 Hz, 1H), 6.79 (t, J = 8.0 Hz, 1H), 5.90 – 5.82 (m, 1H), 5.07 (d, J = 17.1 Hz, 1H), 5.02 (d, J = 10.2 Hz, 1H), 2.80 (t, J = 8.0 Hz, 2H), 2.37 (dt, J = 7.6, 7.4 Hz, 2H). ^{13}C NMR (125 MHz, $CDCl_3$) δ 161.9 (d, J = 246.2 Hz), 143.1 (d, J = 7.3 Hz), 137.1, 133.8 (d, J = 8.1 Hz), 118.4 (d, J = 3.0 Hz), 117.2 (d, J = 22.4 Hz), 115.6, 114.7 (d, J = 22.4 Hz), 35.7, 33.4. ^{19}F NMR (471 MHz, $CDCl_3$) δ -115.4. HRMS (HREI) calcd for $C_{10}H_{10}BrF$ [M]: 227.9944, Found: 227.9948.

2-bromo-1-(but-3-en-1-yl)-3-fluorobenzene (S6)

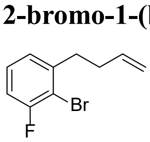

Following the general procedure **A**, **S6** was purified as colorless liquid (0.56 g, 92% yield) by flash column chromatography (petroleum ether). Analytical data: ^{1}H NMR (400 MHz, $CDCl_3$) δ 7.22 – 7.17 (m, 1H), 7.01 – 6.94 (m, 2H), 5.91 – 5.81 (m, 1H), 5.06 (dd, J = 17.1, 1.5 Hz, 1H), 4.98 (d, J = 10.2 Hz, 1H), 2.86 (t, J = 7.6 Hz, 2H), 2.37 (dt, J = 8.0, 7.6 Hz, 2H). ^{13}C NMR (100 MHz, $CDCl_3$) δ 159.4 (d, J = 247.0 Hz), 143.7, 137.4, 128.2 (d, J = 8.2 Hz), 125.6 (d, J = 3.3 Hz), 115.6, 114.0 (d, J = 23.0 Hz), 35.4 (d, J = 2.5 Hz), 33.8. ^{19}F NMR (376 MHz, $CDCl_3$) δ -104.7. HRMS (HREI) calcd

for $C_{10}H_{10}BrF$ [M]: 227.9944, Found: 227.9944.

1-bromo-2-(but-3-en-1-yl)-4,5-difluorobenzene (S7)

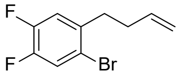

Following the general procedure **A**, **S7** was purified as colorless liquid (0.26 g, 61% yield) by flash column chromatography (petroleum ether). Analytical data: ^{1}H NMR (400 MHz, $CDCl_3$) δ 7.36 (dd, *J* = 9.5, 7.7 Hz, 1H), 7.04 (dd, *J* = 10.8, 8.5 Hz, 1H), 5.88 – 5.78 (m, 1H), 5.07 – 5.00 (m, 2H), 2.76 (t, *J* = 7.6 Hz, 2H), 2.34 (dt, *J* = 7.6, 7.1 Hz, 2H). ^{13}C NMR (100 MHz, $CDCl_3$) δ 147.0 (d, *J* = 282.0 Hz), 139.4 (d, *J* = 269.0 Hz), 137.0, 121.6 (d, *J* = 3.6 Hz), 121.4 (d, *J* = 3.7 Hz), 118.5 (d, *J* = 14.7 Hz), 115.9, 35.1, 33.7. ^{19}F NMR (376 MHz, $CDCl_3$) δ -138.81, -138.87, -138.91, -138.97. HRMS (HREI) calcd for $C_{10}H_9BrF_2$ [M]: 245.9850, Found: 245.9851.

2-bromo-1-(but-3-en-1-yl)-4-(trifluoromethyl)benzene (S8)

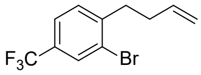

Following the general procedure **A**, **S8** was purified as colorless liquid (0.3 g, 67% yield) by flash column chromatography (petroleum ether). Analytical data: ^{1}H NMR (400 MHz, $CDCl_3$) δ 7.80 (s, 1H), 7.49 (d, *J* = 7.6 Hz, 1H), 7.32 (d, *J* = 8.0 Hz, 1H), 5.91 – 5.81 (m, 1H), 5.06 (d, *J* = 14.0 Hz, 1H), 5.02 (d, *J* = 8.4 Hz, 1H), 2.88 (t, *J* = 7.6 Hz, 2H), 2.39 (dt, *J* = 7.6, 5.7 Hz, 2H). ^{13}C NMR (100 MHz, $CDCl_3$) δ 145.2, 136.9, 130.6, 130.0 (q, *J* = 32.9 Hz), 129.7, 124.5, 124.2 (q, *J* = 3.7 Hz), 123.3 (q, *J* = 272.3 Hz), 115.7, 35.5, 33.4. ^{19}F NMR (376 MHz, $CDCl_3$) δ -62.5. HRMS (HREI) calcd for $C_{11}H_{10}BrF_3$ [M]: 277.9912, Found: 277.9909.

1-bromo-2-(but-3-en-1-yl)-3-(difluoromethyl)benzene (S9)

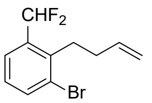

Following the general procedure **A**, **S9** was purified as colorless liquid (0.76 g, 87% yield) by flash column chromatography (petroleum ether). Analytical data: ^{1}H NMR (500 MHz, $CDCl_3$) δ 7.68 (d, *J* = 8.0 Hz, 1H), 7.52 (d, *J* = 7.7 Hz, 1H), 7.19 – 7.17 (m, 1H), 6.80 (tt, *J* = 55.0, 2.0 Hz, 1H), 5.95 – 5.89 (m, 1H), 5.14 – 5.07 (m, 2H), 2.97 (br s, 2H), 2.35 (br s, 2H). ^{13}C NMR (125 MHz, $CDCl_3$) δ 139.6 (t, *J* = 4.3 Hz), 137.1, 135.5, 134.0 (t, *J* = 21.3 Hz), 127.7, 126.1, 125.3 (t, *J* = 7.4 Hz), 115.5, 113.6 (t, *J* = 237.5 Hz), 33.6, 31.5. ^{19}F NMR (471 MHz, $CDCl_3$) δ -110.6. HRMS (HREI) calcd for $C_{11}H_{11}BrF_2$ [M]: 260.0007, Found: 260.0008.

methyl 3-bromo-2-(but-3-en-1-yl)benzoate (S10)

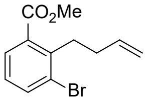

Following the general procedure **D**, **S10** was purified as colorless liquid (0.35 g, 40% yield) by flash column chromatography (ethyl acetate/petroleum ether = 1/60, V/V). Analytical data: ^{1}H NMR (400 MHz, $CDCl_3$) δ 7.74 (dd, *J* = 7.8, 1.2 Hz, 1H), 7.70 (dd, *J* = 8.0, 1.2 Hz, 1H), 7.11 (t, *J* = 7.9 Hz, 1H), 5.98 – 5.88 (m, 1H), 5.12 – 4.99 (m, 2H), 3.90 (s, 3H), 3.16 – 3.12 (m, 2H), 2.39 – 2.33 (m,

2H),. ^{13}C NMR (100 MHz, $CDCl_3$) δ 167.8, 142.1, 138.0, 136.5, 132.6, 129.6, 127.2, 126.7, 115.1, 52.5, 33.8, 33.3. HRMS (ESI) calcd for $C_{12}H_{14}BrO_2$ $[M + H]^+$: 269.0172, Found: 269.0167.

methyl 2-bromo-3-(but-3-en-1-yl)-6-fluorobenzoate (S11)

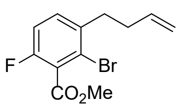

Following the general procedure **D**, **S11** was purified as colorless liquid (0.2 g, 44% yield) by flash column chromatography (ethyl acetate/petroleum ether = 1/50, V/V). Analytical data: ^{1}H NMR (400 MHz, $CDCl_3$) δ 7.24 – 7.22 (m, 1H), 7.02 (t, J = 8.5 Hz, 1H), 5.88 – 5.78 (m, 1H), 5.07 – 4.99 (m, 2H), 3.97 (s, 3H), 2.82 (t, J = 7.6 Hz, 2H), 2.38 – 2.31 (m, 2H). ^{13}C NMR (100 MHz, $CDCl_3$) δ 165.0, 157.6 (d, J = 251.5 Hz), 137.8 (d, J = 3.6 Hz), 137.1, 132.0 (d, J = 8.3 Hz), 125.3 (d, J = 20.5 Hz), 121.4 (d, J = 4.5 Hz), 115.8, 114.8 (d, J = 20.8 Hz), 53.2, 35.2, 33.7. ^{19}F NMR (376 MHz, $CDCl_3$) δ -116.0. HRMS (ESI) calcd for $C_{12}H_{13}BrFO_2$ $[M + H]^+$: 287.0077, Found: 287.0076.

1-bromo-2-(but-3-en-1-yl)-3-chlorobenzene (S12)

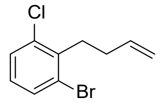

Following the general procedure **A**, **S12** was purified as colorless liquid (0.56 g, 95% yield) by flash column chromatography (petroleum ether). Analytical data:^{1}H NMR (400 MHz, $CDCl_3$) δ 7.46 (dd, J = 8.0, 1.3 Hz, 1H), 7.31 (dt, J = 8.0, 1.3 Hz, 1H), 6.98 (t, J = 8.0 Hz, 1H), 6.00 – 5.90 (m, 1H), 5.10 (dd, J = 17.1, 1.6 Hz, 1H), 4.98 (d, J = 10.2, 0.5 Hz, 1H), 3.06 – 3.02 (m, 2H), 2.36 – 2.30 (m 2H). ^{13}C NMR (100 MHz, $CDCl_3$) δ 139.2, 137.5, 135.0, 131.5, 128.8, 128.0, 125.5, 115.2, 33.6, 32.2. HRMS (HREI) calcd for $C_{10}H_{10}BrCl$ [M]: 243.9649, Found: 243.9651.

2-bromo-1-(but-3-en-1-yl)-4-methylbenzene (S13)

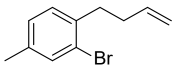

Following the general procedure **A**, **S13** was purified as colorless liquid (1.12 g, 88% yield) by flash column chromatography (petroleum ether). Analytical data: ^{1}H NMR (400 MHz, $CDCl_3$) δ 7.37 (s, 1H), 7.09 (d, J = 7.7 Hz, 1H), 7.03 (d, J = 7.8, 1H), 5.93 – 5.83 (m, 1H), 5.01 – 4.98 (m, 2H), 2.79 (t, J = 7.6 Hz, 2H), 2.38 – 2.32 (m, 2H), 2.30 (s, 3H). ^{13}C NMR (100 MHz, $CDCl_3$) δ 138.0, 137.9, 137.7, 133.3, 130.2, 128.3, 124.3, 115.2, 35.3, 34.1, 20.7. HRMS (ESI) calcd for $C_{11}H_{14}Br$ $[M + H]^+$: 225.0273, Found: 225.0276.

2-bromo-1-(but-3-en-1-yl)-4-(tert-butyl)benzene (S14)

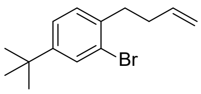

Following the general procedure **A**, **S14** was purified as colorless liquid (1.22 g, 92% yield) by flash column chromatography (petroleum ether). Analytical data: ^{1}H NMR (400 MHz, $CDCl_3$) δ 7.53 (d, J = 2.0 Hz, 1H), 7.24 (dd, J = 7.9, 2.1 Hz, 1H), 7.14 (d, J = 8.0 Hz, 1H), 5.94 – 5.84 (m, 1H), 5.07 (dd, J = 17.1, 1.5 Hz, 1H), 5.00 (dd, J = 10.2, 0.9 Hz, 1H), 2.79 (t, J = 7.6 Hz, 2H), 2.36 (dt, J = 8.8,

6.8 Hz, 2H), 1.29 (s, 9H). ^{13}C NMR (100 MHz, $CDCl_3$) δ 151.1, 138.04, 138.00, 130.0, 129.9, 124.6, 124.4, 115.2, 35.3, 34.6, 34.0, 31.4. HRMS (ESI) calcd for $C_{14}H_{20}Br$ $[M + H]^+$: 267.0743, Found: 267.0749.

1-bromo-2-(but-3-en-1-yl)-4-methoxybenzene (S15)

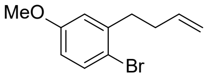

Following the general procedure **A**, **S15** was purified as colorless liquid (0.50 g, 83% yield) by flash column chromatography (petroleum ether). Analytical data: ^{1}H NMR (500 MHz, $CDCl_3$) δ 7.41 (d, *J* = 8.6 Hz, 1H), 6.77 (s, 1H), 6.63 (d, *J* = 9.0 Hz, 1H), 5.93 – 5.85 (m, 1H), 5.08 (d, *J* = 17.1 Hz, 1H), 5.01 (d, *J* = 10.2 Hz, 1H), 3.78 (s, 3H), 2.79 (t, *J* = 8.0 Hz, 2H), 2.37 (dt, *J* = 7.4, 7.0 Hz, 2H). ^{13}C NMR (125 MHz, $CDCl_3$) δ 158.9, 142.1, 137.6, 133.2, 116.1, 115.2, 114.9, 113.1, 55.4, 35.8, 33.8. HRMS (ESI) calcd for $C_{11}H_{14}BrO$ $[M + H]^+$: 241.0223, Found: 241.0215.

2-bromo-1-(but-3-en-1-yl)-3,4-dimethoxybenzene (S16)

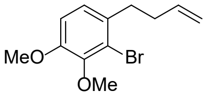

Following the general procedure **A**, **S16** was purified as colorless liquid (0.34 g, 88% yield) by flash column chromatography (petroleum ether). Analytical data: ^{1}H NMR (400 MHz, $CDCl_3$) δ 6.93 (d, *J* = 8.4 Hz, 1H), 6.80 (d, *J* = 8.4 Hz, 1H), 5.92 – 5.82 (m, 1H), 5.08 – 4.97 (m, 2H), 3.85 (s, 3H), 3.84 (s, 3H), 2.80 – 2.76 (m, 2H), 2.37 – 2.31 (m, 2H). ^{13}C NMR (100 MHz, $CDCl_3$) δ 151.7, 146.5, 137.8, 134.1, 124.9, 120.1, 115.1, 111.2, 60.4, 56.1, 35.3, 34.1. HRMS (ESI) calcd for $C_{12}H_{16}BrO_2$ $[M + H]^+$: 271.0328, Found: 271.0326.

***tert*-butyl (3-bromo-4-(but-3-en-1-yl)phenyl)carbamate (S17)**

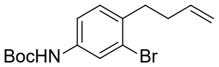

Following the general procedure **A**, **S17** was purified as colorless liquid (0.18 g, 78% yield) by flash column chromatography (petroleum ether). Analytical data: ^{1}H NMR (400 MHz, $CDCl_3$) δ 7.66 (br s, 1H), 7.16 (d, *J* = 8.4 Hz, 1H), 7.09 (d, *J* = 8.0 Hz, 1H)), 6.44 (br s, 1H), 5.04 (d, *J* = 17.2 Hz, 1H), 4.98 (d, *J* = 10.2 Hz, 1H), 5.01 – 4.97 (m, 2H), 2.76 (t, *J* = 7.2 Hz, 2H), 2.32 (dt, *J* = 7.8, 7.2 Hz, 2H), 1.51 (s, 9H). ^{13}C NMR (100 MHz, $CDCl_3$) δ 152.6, 137.8, 137.5, 135.6, 130.5, 124.5, 122.6, 117.6, 115.3, 81.0, 35.0, 34.1, 28.4. HRMS (ESI) calcd for $C_{15}H_{20}BrNNaO_2$ $[M + Na]^+$: 348.0570, Found:348.0572

2-bromo-1,4-di(but-3-en-1-yl)benzene (S18)

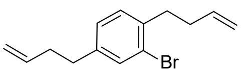

Following the general procedure **A**, **S18** was purified as colorless liquid (0.67 g, 86% yield) by flash column chromatography (petroleum ether). Analytical data: ^{1}H NMR (400 MHz, $CDCl_3$) δ 7.37 (s, 1H), 7.12 (d, *J* = 7.7 Hz, 1H), 7.05 (dd, *J* = 7.8, 1.8 Hz, 1H), 5.92 – 5.80 (m, 2H), 5.09 – 4.98 (m, 4H), 2.81 – 2.77 (m, 2H), 2.67 – 2.63 (m, 2H), 2.38 – 2.32 (m, 4H). ^{13}C NMR (100 MHz, $CDCl_3$) δ 141.7, 138.5, 137.9, 137.8, 132.7, 130.3, 127.6, 124.4, 115.4, 115.2, 35.4, 35.3, 34.6, 34.1. HRMS

(HREI) calcd for $C_{14}H_{17}Br$ [M]: 264.0508, Found: 264.0508.

4-bromo-3-(but-3-en-1-yl)-1,1'-biphenyl (S19)

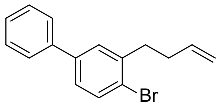

Following the general procedure **A**, **S19** was purified as colorless liquid (1.22 g, 92% yield) by flash column chromatography (petroleum ether). Analytical data: ^{1}H NMR (400 MHz, $CDCl_3$) δ 7.64 – 7.56 (m, 3H), 7.50 – 7.43 (m, 3H), 7.41 – 7.35 (m, 1H), 7.30 (dd, *J* = 8.3, 2.3 Hz, 1H), 5.94 – 5.87 (m, 1H), 5.12 – 5.00 (m, 2H), 2.90 – 2.86 (m, 2H), 2.45 – 2.39 (m, 2H). ^{13}C NMR (100 MHz, $CDCl_3$) δ 141.4, 140.5, 140.2, 137.6, 133.1, 129.1, 128.9, 127.6, 127.0, 126.3, 123.6, 115.3, 35.8, 33.9. HRMS (ESI) calcd for $C_{16}H_{16}Br$ $[M + H]^+$: 287.0430, Found: 287.0436.

4-bromo-5-(but-3-en-1-yl)-2,3-dihydrobenzofuran (S20)

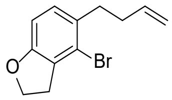

Following the general procedure **A**, **S20** was purified as colorless liquid (0.67 g, 86% yield) by flash column chromatography (petroleum ether). Analytical data: ^{1}H NMR (400 MHz, $CDCl_3$) δ 6.95 (d, *J* = 8.1 Hz, 1H), 6.65 (d, *J* = 8.1 Hz, 1H), 5.92 – 5.82 (m, 1H), 5.08 – 4.97 (m, 2H), 4.59 (t, *J* = 8.8 Hz, 2H), 3.22 (t, *J* = 8.8 Hz, 2H), 2.75 (t, *J* = 7.6 Hz, 2H), 2.35 – 2.30 (m, 2H). ^{13}C NMR (100 MHz, $CDCl_3$) δ 158.5, 138.0, 132.7, 129.6, 128.9, 121.1, 115.2, 108.1, 71.0, 34.8, 34.5, 32.4. HRMS (ESI) calcd for $C_{12}H_{14}BrO$ $[M + H]^+$: 253.0223, Found: 253.0219.

***tert*-butyl (4-bromo-5-(but-3-en-1-yl)-2,3-dihydrobenzofuran-7-yl)carbamate (S21)**

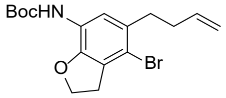

Following the general procedure **A**, **S21** was purified as colorless liquid (0.43 g, 86% yield) by flash column chromatography (ethyl acetate/petroleum ether = 1/30, V/V). Analytical data: ^{1}H NMR (400 MHz, $CDCl_3$) δ 7.74 (br s, 1H), 6.50 (br s, 1H), 5.93 – 5.83 (m, 1H), 5.06 (dd, *J* = 17.1, 1.5 Hz, 1H), 4.98 (d, *J* = 10.2 Hz, 1H), 4.62 (t, *J* = 8.7 Hz, 2H), 3.24 (t, *J* = 8.7 Hz, 2H), 2.76 – 2.72 (m, 2H), 2.35 – 2.30 (m, 2H), 1.51 (s, 9H). ^{13}C NMR (100 MHz, $CDCl_3$) δ 152.7, 146.9, 138.1, 133.6, 127.9, 121.9, 119.0, 115.0, 113.3, 80.8, 71.6, 35.2, 34.8, 32.8, 28.4. HRMS (ESI) calcd for $C_{17}H_{21}BrNO_3^-$ $[M]^-$: 366.0710, Found: 366.0716.

1-bromo-2-(2,2-dimethylbut-3-en-1-yl)benzene (S22)

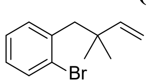

Following the general procedure **B**, **S22** was purified as colorless liquid (1.21 g, 85% yield) by flash column chromatography (petroleum ether). Analytical data: ^{1}H NMR (400 MHz, $CDCl_3$) δ 7.54 (d, *J* = 7.9 Hz, 1H), 7.20 – 7.19 (m, 2H), 7.07 – 7.01 (m, 2H), 5.94 (dd, *J* = 17.4, 10.7 Hz, 1H), 4.93 (dd, *J* = 10.7, 1.3 Hz, 1H), 4.85 (dd, *J* = 17.4, 1.3 Hz, 1H), 2.84 (s, 2H), 1.08 (s, 6H). ^{13}C NMR (100 MHz, $CDCl_3$) δ 148.0, 138.6, 133.0, 132.6, 127.7, 126.6, 126.2, 110.9, 47.0, 39.0, 26.7. HRMS

(HREI) calcd for $C_{12}H_{15}Br$ [M]: 238.0352, Found: 238.0353.

1-bromo-2-(pent-4-en-2-yl)benzene (S23)

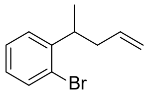

Following the general procedure **A**, **S23** was purified as colorless liquid (1.82 g, 90% yield) by flash column chromatography (petroleum ether). Analytical data: ^{1}H NMR (400 MHz, $CDCl_3$) δ 7.55 (d, J = 8.0 Hz, 1H), 7.33 – 7.23 (m, 2H), 7.07 – 7.03 (m, 1H), 5.81 – 5.69 (m, 1H), 5.05 – 4.98 (m, 2H), 3.39 – 3.31 (m, 1H), 2.47 – 2.40 (m, 1H), 2.30 – 2.23 (m, 1H), 1.24 (d, J = 6.8 Hz, 3H). ^{13}C NMR (100 MHz, $CDCl_3$) δ 145.7, 136.7, 132.9, 127.6, 127.49, 127.47, 124.8, 116.4, 41.4, 37.9, 20.4. HRMS (ESI) calcd for $C_{11}H_{14}Br$ $[M + H]^+$: 225.0273, Found: 225.0267.

1-bromo-2-(1-phenylbut-3-en-1-yl)benzene (S24)

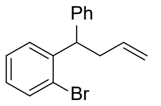

Following the general procedure **B**, **S24** was purified as colorless liquid (0.47 g, 85% yield) by flash column chromatography (petroleum ether). Analytical data: ^{1}H NMR (400 MHz, $CDCl_3$) δ 7.52 (d, J = 1.2 Hz, 1H), 7.30 – 7.21 (m, 6H), 7.20 – 7.17 (m, 1H), 7.06 – 7.01 (m, 1H), 5.80 – 5.69 (m, 1H), 5.07 – 4.95 (m, 2H), 4.58 (t, J = 7.9 Hz, 1H), 2.81 – 2.77 (m, 2H). ^{13}C NMR (100 MHz, $CDCl_3$) δ 143.5, 143.1, 136.3, 133.2, 129.1, 128.5, 128.4, 127.9, 127.6, 126.5, 125.4, 116.8, 49.3, 39.8. HRMS (ESI) calcd for $C_{16}H_{16}Br$ $[M + H]^+$: 287.0430, Found: 287.0439.

1-bromo-2-(pent-4-en-1-yl)benzene (**S25**)

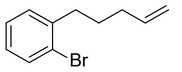

Following the general procedure **C**, **S25** was purified as colorless liquid (1.21 g, 85% yield) by flash column chromatography (petroleum ether). Analytical data: ^{1}H NMR (400 MHz, $CDCl_3$) δ 7.52 (d, J = 7.6 Hz, 1H), 7.25 – 7.20 (m, 2H), 7.08 – 7.02 (m, 1H), 5.91 – 5.80 (m, 1H), 5.07 – 4.97 (m, 2H), 2.76 – 2.72 (m, 2H), 2.16 – 2.11 (m, 2H), 1.75 – 1.68 (m, 2H). ^{13}C NMR (100 MHz, $CDCl_3$) δ 141.9, 138.6, 132.9, 130.5, 127.6, 127.5, 124.6, 115.0, 35.8, 33.5, 29.2. HRMS (ESI) calcd for $C_{11}H_{14}Br$ $[M + H]^+$: 225.0273, Found: 225.0279.

1-bromo-4-fluoro-2-(pent-4-en-1-yl)benzene (**S26**)

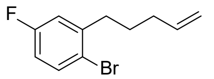

Following the general procedure **C**, **S26** was purified as colorless liquid (0.46 g, 72% yield) by flash column chromatography (petroleum ether). Analytical data: ^{1}H NMR (400 MHz, $CDCl_3$) δ 7.47 (dd, J = 8.7, 5.6 Hz, 1H), 6.94 (dt, J = 9.4, 1.9 Hz, 1H), 6.81 – 6.76 (m, 1H), 5.89 – 5.79 (m, 1H), 5.06 (d, J = 17.2 Hz, 1H), 5.00 (d, J = 10.2 Hz, 1H), 2.71 (t, J = 7.6 Hz, 2H), 2.14 (dt, J = 7.2, 7.0 Hz, 2H), 1.74 – 1.67 (m, 2H). ^{13}C NMR (100 MHz, $CDCl_3$) δ 161.9 (d, J = 246.2 Hz), 143.9 (d, J = 7.3 Hz), 138.1, 133.8 (d, J = 8.1 Hz), 118.4 (d, J = 3.1 Hz), 117.1 (d, J = 22.4 Hz), 115.1, 114.6 (d, J = 22.5 Hz), 35.8, 33.4, 28.8. ^{19}F NMR (471 MHz, $CDCl_3$) δ -115.5. HRMS (HREI) calcd for

$C_{11}H_{12}BrF$ [M]: 242.0101, Found: 242.0101.

2-bromo-4-methoxy-1-(pent-4-en-1-yl)benzene (**S27**)

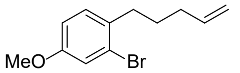

Following the general procedure **C**, **S27** was purified as colorless liquid (0.84 g, 66% yield) by flash column chromatography (petroleum ether). Analytical data: ^{1}H NMR (400 MHz, $CDCl_3$) δ 7.12 – 7.10 (m, 2H), 6.79 (d, J = 8.4 Hz, 1H), 5.91 – 5.81 (m, 1H), 5.05 (d, J = 16.8 Hz, 1H), 5.00 (d, J = 10.4 Hz, 1H), 3.78 (s, 3H), 2.68 (t, J = 7.6 Hz, 2H), 2.12 (dt, J = 7.2, 6.8 Hz, 2H), 1.72 – 1.65 (m, 2H). ^{13}C NMR (100 MHz, $CDCl_3$) δ 158.4, 138.6, 133.8, 130.7, 124.5, 118.0, 114.9, 113.7, 55.6, 34.8, 33.5, 29.5. HRMS (ESI) calcd for $C_{12}H_{16}BrO$ [M + H]$^+$: 255.0379, Found: 255.0376.

Supplementary Note 7. Identification of arylalkylation compounds

_N_-(2-(2,3-dihydro-1_H_-inden-1-yl)ethyl)propionamide (3)

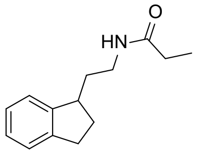

Following the general procedure **E**, **3** was purified as colorless powder (39 mg, 90% yield) by flash column chromatography (ethyl acetate/petroleum ether = 1/1.5, V/V). Analytical data: ^{1}H NMR (400 MHz, $CDCl_3$) δ 7.22 – 7.14 (m, 4H), 5.47 (br s, 1H), 3.42 – 3.37 (m, 2H), 3.19 – 3.11 (m, 1H), 2.97 – 2.80 (m, 2H), 2.36 – 2.28 (m, 1H), 2.19 (q, J = 7.6 Hz, 2H), 2.11 – 2.03 (m, 1H), 1.76 – 1.57 (m, 2H), 1.15 (t, J = 7.6 Hz, 3H). ^{13}C NMR (100 MHz, $CDCl_3$) δ 173.8, 146.6, 143.9, 126.5, 126.2, 124.6, 123.5, 42.5, 38.0, 34.9, 32.0, 31.4, 29.8, 9.9. HRMS (ESI) calcd for $C_{14}H_{20}NO$ $[M + H]^+$: 218.1539, Found: 218.1543.

_N_-(2-(6-fluoro-2,3-dihydro-1_H_-inden-1-yl)ethyl)propionamide (4)

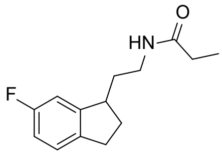

Following the general procedure **E**, **4** was purified as colorless powder (34 mg, 72% yield) by flash column chromatography (ethyl acetate/petroleum ether = 1/1, V/V). Analytical data: ^{1}H NMR (400 MHz, $CDCl_3$) δ 7.12 (dd, J = 8.2, 5.3 Hz, 1H), 6.87 – 6.80 (m, 2H), 5.55 (br s, 1H), 3.42 – 3.32 (m, 2H), 3.15 – 3.08 (m, 1H), 2.87 – 2.76 (m, 2H), 2.38 – 2.30 (m, 1H), 2.20 (q, J = 7.6 Hz, 2H), 2.07 – 1.98 (m, 1H), 1.78 – 1.69 (m, 1H), 1.64 – 1.55 (m, 1H), 1.15 (t, J = 7.6 Hz, 3H). ^{13}C NMR (100 MHz, $CDCl_3$) δ 173.9, 162.2 (d, J = 242.6 Hz), 149.0 (d, J = 7.4 Hz), 139.2 (d, J = 2.5 Hz), 125.4 (d, J = 8.7 Hz), 113.4 (d, J = 22.3 Hz), 110.6 (d, J = 22.0 Hz), 42.8 (d, J = 2.1 Hz), 37.9, 34.9, 32.7, 30.8, 29.9, 10.0. ^{19}F NMR (376 MHz, $CDCl_3$) δ -117.6. HRMS (ESI) calcd for $C_{14}H_{19}FNO$ $[M + H]^+$: 236.1445, Found: 236.1450.

_N_-(2-(5-fluoro-2,3-dihydro-1_H_-inden-1-yl)ethyl)propionamide (5)

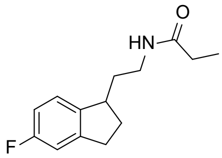

Following the general procedure **E**, **5** was purified as colorless powder (42 mg, 89% yield) by flash column chromatography (ethyl acetate/petroleum ether = 1/1, V/V). Analytical data: ^{1}H NMR (400 MHz, $CDCl_3$) δ 7.12 (dd, J = 8.2, 5.2 Hz, 1H), 6.91 – 6.81 (m, 2H), 5.45 (br s, 1H), 3.40 – 3.35 (m, 2H), 3.13 – 3.05 (m, 1H), 2.94 – 2.77 (m, 2H), 2.38 – 2.30 (m, 1H), 2.20 (q, J = 7.6 Hz, 2H), 2.06 – 1.98 (m, 1H), 1.78 – 1.69 (m, 1H), 1.63 – 1.54 (m, 1H), 1.15 (t, J = 7.6 Hz, 3H). ^{13}C NMR (100 MHz, $CDCl_3$) δ 173.9, 162.3 (d, J = 243.1 Hz), 146.2 (d, J = 8.1 Hz), 142.1, 124.4 (d, J = 8.9 Hz), 113.1 (d, J = 22.5 Hz), 111.6 (d, J = 21.8 Hz), 41.9, 38.0, 35.2, 32.6, 31.6 (d, J = 2.2 Hz), 29.9, 10.0. ^{19}F NMR (376 MHz, $CDCl_3$) δ -117.4. HRMS (ESI) calcd for $C_{14}H_{19}FNO$ $[M + H]^+$: 236.1445, Found: 236.1440.

***N*-(2-(7-fluoro-2,3-dihydro-1*H*-inden-1-yl)ethyl)propionamide** (**6**)

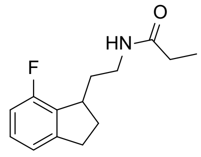

Following the general procedure **E**, **6** was purified as colorless powder (31 mg, 66% yield) by flash column chromatography (ethyl acetate/petroleum ether = 1/4, V/V). Analytical data: ^{1}H NMR (400 MHz, $CDCl_3$) δ 7.11 (td, *J* = 7.8, 5.2 Hz, 1H), 6.98 (d, *J* = 7.4 Hz, 1H), 6.81 (t, *J* = 8.9 Hz, 1H), 5.67 (br s, 1H), 3.45 – 3.33 (m, 2H), 3.28 – 3.20 (m, 1H), 3.03 – 2.95 (m, 1H), 2.87 – 2.80 (m, 1H), 2.32 – 2.23 (m, 1H), 2.18 (q, *J* = 7.6 Hz, 2H), 2.03 – 1.94 (m, 1H), 1.89 – 1.82 (m, 1H), 1.78 – 1.69 (m, 1H), 1.13 (t, *J* = 7.6 Hz, 3H). ^{13}C NMR (100 MHz, $CDCl_3$) δ 173.9, 159.8 (d, *J* = 245.5 Hz), 147.6 (d, *J* = 5.9 Hz), 132.3 (d, *J* = 16.2 Hz), 128.5 (d, *J* = 7.4 Hz), 120.5 (d, *J* = 3.1 Hz), 113.1 (d, *J* = 21.4 Hz), 40.2 (d, *J* = 2.1 Hz), 37.9, 34.0 (d, *J* = 1.9 Hz), 31.7 (d, *J* = 1.6 Hz), 31.6, 29.8, 10.0. ^{19}F NMR (376 MHz, $CDCl_3$) δ -119.4. HRMS (ESI) calcd for $C_{14}H_{19}FNO$ $[M + H]^+$: 236.1445, Found: 236.1445.

***N*-(2-(5,6-difluoro-2,3-dihydro-1*H*-inden-1-yl)ethyl)propionamide** (**7**)

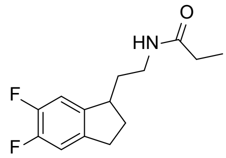

Following the general procedure **E**, **7** was purified as colorless powder (36 mg, 71% yield) by flash column chromatography (ethyl acetate/petroleum ether = 1/2, V/V). Analytical data: ^{1}H NMR (400 MHz, $CDCl_3$) δ 6.99 – 6.94 (m, 2H), 5.50 (br s, 1H), 3.41 – 3.34 (m, 2H), 3.11 – 3.07 (m, 1H), 2.90 – 2.76 (m, 2H), 2.38 – 2.33 (m, 1H), 2.20 (q, *J* = 7.6 Hz, 2H), 2.02 – 1.96 (m, 1H), 1.78 – 1.71 (m, 1H), 1.61 – 1.55 (m, 1H), 1.16 (t, *J* = 7.6 Hz, 3H). ^{13}C NMR (150 MHz, $CDCl_3$) δ 173.9, 150.3 (q, *J* = 164.6 Hz), 148.7 (q, *J* = 164.6 Hz), 142.7 (q, *J* = 3.3 Hz), 139.6 (q, *J* = 3.2 Hz), 113.1 (d, *J* = 17.4 Hz), 112.1 (d, *J* = 17.6 Hz), 42.4, 37.9, 35.1, 32.6, 31.2, 29.9, 10.0. ^{19}F NMR (376 MHz, $CDCl_3$) δ -141.0, -141.1, -141.2, -141.3. HRMS (ESI) calcd for $C_{14}H_{18}F_2NO$ $[M + H]^+$: 254.1351, Found: 254.1354.

***N*-(2-(6-(trifluoromethyl)-2,3-dihydro-1*H*-inden-1-yl)ethyl)propionamide** (**8**)

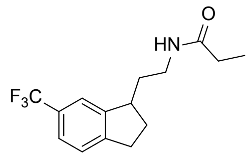

Following the general procedure **E**, **8** was purified as colorless powder (39 mg, 69% yield) by flash column chromatography (ethyl acetate/petroleum ether = 1/2, V/V). Analytical data: ^{1}H NMR (600 MHz, $CDCl_3$) δ 7.41 (d, *J* = 7.8 Hz, 2H), 7.30 (d, *J* = 7.8 Hz, 1H), 5.49 (br s, 1H), 3.45 – 3.36 (m, 2H), 3.20 – 3.15 (m, 1H), 3.00 – 2.95 (m, 1H), 2.91 – 2.86 (m, 1H), 2.41 – 2.36 (m, 1H), 2.20 (q, *J* = 7.6 Hz, 2H), 2.13 – 2.08 (m, 1H), 1.80 – 1.74 (m, 1H), 1.66 – 1.59 (m, 1H), 1.16 (t, *J* = 7.6 Hz, 3H). ^{13}C NMR (150 MHz, $CDCl_3$) δ 173.9, 148.3, 147.6, 128.9 (q, *J* = 31.8 Hz), 125.6, 124.9, 123.9 (q, *J* = 3.9 Hz), 120.4 (d, *J* = 3.9 Hz), 42.5, 38.0, 35.0, 32.1, 31.6, 29.9, 10.0. ^{19}F NMR (376 MHz, $CDCl_3$) δ -61.8. HRMS (ESI) calcd for $C_{15}H_{19}F_3NO$ $[M + H]^+$: 286.1413, Found: 286.1419.

N-(2-(4-(difluoromethyl)-2,3-dihydro-1H-inden-1-yl)ethyl)propionamide (9)

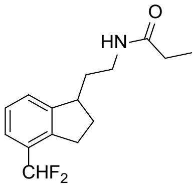

Following the general procedure **E**, **9** was purified as colorless powder (51 mg, 95% yield) by flash column chromatography (ethyl acetate/petroleum ether = 1/2, V/V). Analytical data: ^{1}H NMR (400 MHz, $CDCl_3$) δ 7.31 – 7.21 (m, 3H), 6.65 (t, *J* = 55.8 Hz, 1H), 5.67 (br s, 1H), 3.41 – 3.35 (m, 2H), 3.18 – 2.88 (m, 2H), 2.96 – 2.88 (m, 1H), 2.40 – 2.32 (m, 1H), 2.19 (q, *J* = 7.6 Hz, 2H), 2.10 – 2.01 (m, 1H), 1.79 – 1.70 (m, 1H), 1.65 – 1.58 (m, 1H), 1.14 (t, *J* = 7.6 Hz, 3H). ^{13}C NMR (100 MHz, $CDCl_3$) δ 174.0, 148.2, 141.9 (t, *J* = 4.2 Hz), 130.1 (t, *J* = 21.9 Hz), 126.8, 126.0, 123.9 (t, *J* = 6.9 Hz), 114.8 (t, *J* = 237.8 Hz), 42.2, 38.0, 35.0, 32.0, 29.8, 29.5, 10.0. ^{19}F NMR (376 MHz, $CDCl_3$) δ -111.1, -111.9, -112.0, -112.09, -112.11, -112.9. HRMS (ESI) calcd for $C_{15}H_{20}F_2NO$ $[M + H]^+$: 268.1507, Found: 268.1507.

methyl 1-(2-propionamidoethyl)-2,3-dihydro-1H-indene-4-carboxylate (10)

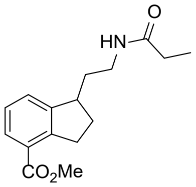

Following the general procedure **E**, **10** was purified as colorless powder (44 mg, 79% yield) by flash column chromatography (ethyl acetate/petroleum ether = 1/2, V/V). Analytical data: ^{1}H NMR (400 MHz, $CDCl_3$) δ 7.83 (d, *J* = 7.8 Hz, 1H), 7.38 (d, *J* = 7.4 Hz, 1H), 7.23 (d, *J* = 7.6 Hz, 1H), 5.48 (br s, 1H), 3.89 (s, 3H), 3.40 – 3.31 (m, 3H), 3.19 – 3.11 (m, 2H), 2.38 – 2.31 (m, 1H), 2.20 (q, *J* = 7.6 Hz, 2H), 2.10 – 2.02 (m, 1H), 1.77 – 1.72 (m, 1H), 1.65 – 1.59 (m, 1H), 1.16 (t, *J* = 7.6 Hz, 3H). ^{13}C NMR (100 MHz, $CDCl_3$) δ 173.9, 167.7, 148.4, 146.5, 128.7, 127.9, 126.7, 126.5, 51.9, 42.2, 38.0, 35.2, 32.6, 31.8, 29.9, 10.0. HRMS (ESI) calcd for $C_{16}H_{22}NO_3$ $[M + H]^+$: 276.1594, Found: 276.1592.

methyl 5-fluoro-3-(2-propionamidoethyl)-2,3-dihydro-1H-indene-4-carboxylate (11)

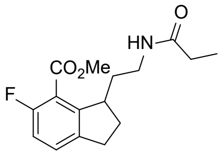

Following the general procedure **E**, **11** was purified as colorless powder (33 mg, 57% yield) by flash column chromatography (ethyl acetate/petroleum ether = 2/1, V/V). Analytical data: ^{1}H NMR (400 MHz, $CDCl_3$) δ 7.29 – 7.27 (m, 1H), 6.92 (dd, *J* = 10.7, 8.3 Hz, 1H), 6.05 (br s, 1H), 3.92 (s, 3H), 3.60 (t, *J* = 8.9 Hz, 1H), 3.40 – 3.24 (m, 2H), 3.01 – 2.92 (m, 1H), 2.81 – 2.75 (m, 1H), 2.26 (d, *J* = 7.6 Hz, 2H), 2.19 – 2.14 (m, 1H), 2.00 – 1.95 (m, 1H), 1.72 – 1.55 (m, 2H), 1.19 (t, *J* = 7.6 Hz, 3H). ^{13}C NMR (100 MHz, $CDCl_3$) δ 174.1, 166.1, 163.0 (d, *J* = 252.6 Hz), 150.8, 140.0 (d, *J* = 3.0 Hz), 129.0 (d, *J* = 9.6 Hz), 115.5 (d, *J* = 13.3 Hz), 114.9 (d, *J* = 24.0 Hz), 52.3, 42.1 (d, *J* = 2.2 Hz), 38.3, 33.5, 30.9, 29.9, 29.8, 9.9. ^{19}F NMR (376 MHz, $CDCl_3$) δ -114.1. HRMS (ESI) calcd for

$C_{16}H_{21}FNO_3$ [M + H]$^+$: 294.1500, Found: 294.1500.

***N*-(2-(4-chloro-2,3-dihydro-1*H*-inden-1-yl)ethyl)propionamide (12)**

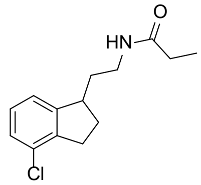

Following the general procedure **E**, **12** was purified as colorless powder (45 mg, 90% yield) by flash column chromatography (ethyl acetate/petroleum ether = 1/2, V/V). Analytical data: ^{1}H NMR (400 MHz, $CDCl_3$) δ 7.10 – 7.04 (m, 3H), 5.63 (br s, 1H), 3.38 – 3.33 (m, 2H), 3.23 – 3.16 (m, 1H), 3.02 – 2.94 (m, 1H), 2.89 – 2.81 (m, 1H), 2.36 – 2.28 (m, 1H), 2.19 (q, *J* = 7.6 Hz, 2H), 2.07 – 1.98 (m, 1H), 1.76 – 1.56 (m, 2H), 1.14 (t, *J* = 7.6 Hz, 3H). ^{13}C NMR (100 MHz, $CDCl_3$) δ 173.9, 148.8, 142.1, 130.8, 127.9, 126.7, 121.8, 43.4, 37.8, 35.0, 31.2, 30.7, 29.8, 9.9. HRMS (ESI) calcd for $C_{14}H_{19}ClNO$ [M + H]$^+$: 252.1150, Found: 252.1148.

***N*-(2-(6-methyl-2,3-dihydro-1*H*-inden-1-yl)ethyl)propionamide (13)**

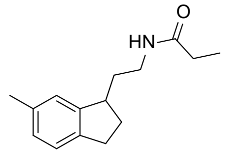

Following the general procedure **E**, **13** was purified as colorless powder (33 mg, 71% yield) by flash column chromatography (ethyl acetate/petroleum ether = 1/3.5, V/V). Analytical data: ^{1}H NMR (400 MHz, $CDCl_3$) δ 7.10 (d, *J* = 7.5 Hz, 1H), 7.01 (s, 1H), 6.97 (d, *J* = 7.6 Hz, 1H), 5.50 (br s, 1H), 3.42 – 3.36 (m, 2H), 3.14 – 3.07 (m, 1H), 2.92 – 2.75 (m, 2H), 2.35 – 2.27 (m, 1H), 2.32 (s, 3H), 2.19 (q, *J* = 7.6 Hz, 2H), 2.10 – 2.02 (m, 1H), 1.72 – 1.55 (m, 2H), 1.15 (t, *J* = 7.6 Hz, 3H). ^{13}C NMR (100 MHz, $CDCl_3$) δ 173.8, 146.9, 141.0, 135.9, 127.4, 124.4, 124.3, 42.6, 38.2, 35.0, 32.4, 31.1, 29.9, 21.4, 10.0. HRMS (ESI) calcd for $C_{15}H_{22}NO$ [M + H]$^+$: 232.1696, Found: 232.1700.

***N*-(2-(6-(*tert*-butyl)-2,3-dihydro-1*H*-inden-1-yl)ethyl)propionamide (14)**

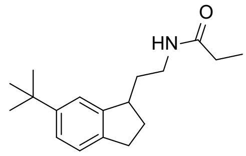

Following the general procedure **E**, **14** was purified as colorless powder (43 mg, 79% yield) by flash column chromatography (ethyl acetate/petroleum ether = 1/3.5, V/V). Analytical data: ^{1}H NMR (400 MHz, $CDCl_3$) δ7.21 (d, *J* = 8.0 Hz, 2H), 7.15 (d, *J* = 7.7 Hz, 1H), 5.47 (br s, 1H), 3.43 – 3.38 (m, 2H), 3.16 – 3.09 (m, 1H), 2.93 – 2.76 (m, 2H), 2.36 – 2.28 (m, 1H), 2.20 (q, *J* = 7.6 Hz, 2H), 2.15 – 2.07 (m, 1H), 1.75 – 1.68 (m, 1H), 1.63 – 1.55 (m, 1H), 1.31 (s, 9H), 1.15 (t, *J* = 7.6 Hz, 3H). ^{13}C NMR (100 MHz, $CDCl_3$) δ 173.8, 149.5, 146.6, 141.1, 124.2, 123.8, 120.4, 42.8, 38.2, 35.2, 34.8, 32.4, 31.8, 31.1, 30.0, 10.0. HRMS (ESI) calcd for $C_{18}H_{28}NO$ [M + H]$^+$: 274.2165, Found: 274.2170.

***N*-(2-(5-methoxy-2,3-dihydro-1*H*-inden-1-yl)ethyl)propionamide** (**15**)

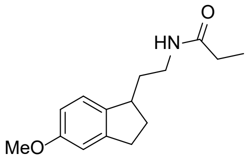

Following the general procedure **E**, **15** was purified as colorless powder (24 mg, 48% yield) by flash column chromatography (ethyl acetate/petroleum ether = 1/2, V/V). Analytical data: ^{1}H NMR (600 MHz, $CDCl_3$) δ 7.08 (d, *J* = 8.2 Hz, 1H), 6.77 (s, 1H), 6.71 (d, *J* = 8.2 Hz, 1H), 5.44 (br s, 1H), 3.77 (s, 3H), 3.39 – 3.35 (m, 2H), 3.10 – 3.05 (m, 1H), 2.91 – 2.78 (m, 2H), 2.34 – 2.29 (m, 1H), 2.18 (q, *J* = 7.5 Hz, 2H), 2.03 – 1.98 (m, 1H), 1.73 – 1.67 (m, 1H), 1.62 – 1.55 (m, 1H), 1.14 (t, *J* = 7.6 Hz, 3H). ^{13}C NMR (150 MHz, $CDCl_3$) δ 173.8, 159.0, 145.6, 138.8, 124.1, 112.2, 110.1, 55.5, 41.8, 38.1, 35.3, 32.6, 31.8, 29.9, 10.0. HRMS (ESI) calcd for $C_{15}H_{22}NO_2$ $[M + H]^+$: 248.1645, Found: 248.1646.

***N*-(2-(6,7-dimethoxy-2,3-dihydro-1*H*-inden-1-yl)ethyl)propionamide** (**16**)

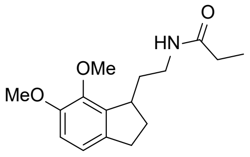

Following the general procedure **E**, **16** was purified as colorless powder (31 mg, 56% yield) by flash column chromatography (ethyl acetate/petroleum ether = 1/1.7, V/V). Analytical data: ^{1}H NMR (400 MHz, $CDCl_3$) δ 6.89 (d, *J* = 8.0 Hz, 1H), 6.74 (d, *J* = 8.0 Hz, 1H), 5.91 (br s, 1H), 3.86 (s, 3H), 3.84 (s, 3H), 3.48 – 3.40 (m, 1H), 3.35 – 3.28 (m, 1H), 3.14 – 3.01 (m, 1H), 2.96 – 2.88 (m, 1H), 2.77 – 2.70 (m, 1H), 2.28 – 2.13 (m, 1H), 2.18 (q, *J* = 7.6 Hz, 2H), 1.88 – 1.74 (m, 3H), 1.14 (t, *J* = 7.6 Hz, 3H). ^{13}C NMR (100 MHz, $CDCl_3$) δ 173.8, 151.0, 145.6, 139.7, 137.7, 119.9, 111.7, 60.7, 56.2, 40.5, 38.0, 33.8, 32.6, 30.7, 30.0, 10.1. HRMS (ESI) calcd for $C_{16}H_{24}NO_3$ $[M + H]^+$: 278.1751, Found: 278.1750.

***tert*-butyl(3-(2-propionamidoethyl)-2,3-dihydro-1*H*-inden-5-yl)carbamate** (**17**)

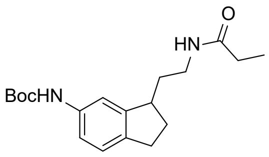

Following the general procedure **E**, **17** was purified as colorless powder (27 mg, 40% yield) by flash column chromatography (ethyl acetate/petroleum ether = 1/2, V/V). Analytical data:^{1}H NMR (400 MHz, $CDCl_3$) δ 7.35 (br s, 1H), 7.10 (d, *J* = 8.0 Hz, 1H), 6.98 (d, *J* = 8.0 Hz, 1H), 6.52 (br s, 1H), 5.55 (br s, 1H), 3.39 – 3.34 (m, 2H), 3.13 – 3.06 (m, 1H), 2.89 – 2.73 (m, 2H), 2.35 – 2.26 (m, 1H), 2.19 (q, *J* = 7.6 Hz, 2H), 2.08 – 1.99 (m, 1H), 1.74 – 1.65 (m, 1H), 1.63 – 1.56 (m, 1H), 1.51 (s, 9H), 1.14 (t, J = 7.6 Hz, 3H). ^{13}C NMR (100 MHz, $CDCl_3$) δ 173.9, 153.2, 147.8, 138.8, 136.8, 124.8, 117.5, 114.4, 80.4, 42.8, 38.0, 34.9, 32.4, 31.0, 29.9, 28.5, 10.0. HRMS (ESI) calcd for $C_{19}H_{29}N_2O_3$ $[M + H]^+$: 333.2173, Found: 333.2175.

***N*-(2-(6-(but-3-en-1-yl)-2,3-dihydro-1*H*-inden-1-yl)ethyl)propionamide (18)**

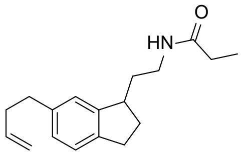

Following the general procedure **E**, **18** was purified as colorless powder (41 mg, 76% yield) by flash column chromatography (ethyl acetate/petroleum ether = 1/3, V/V). Analytical data: ^{1}H NMR (400 MHz, $CDCl_3$) δ 7.13 (d, *J* = 7.6 Hz, 1H), 7.02 (s, 1H), 6.99 (d, *J* = 7.5 Hz, 1H), 5.92 – 5.82 (m, 1H), 5.43 (br s, 1H), 5.08 – 4.96 (m, 2H), 3.43 – 3.37 (m, 2H), 3.13 – 3.10 (m, 1H), 2.93 – 2.77 (m, 2H), 2.70 – 2.66 (m, 2H), 2.39 – 2.28 (m, 3H), 2.19 (q, *J* = 7.6 Hz, 2H), 2.12 – 2.04 (m, 1H), 1.75 – 1.56 (m, 2H), 1.15 (t, *J* = 7.6 Hz, 3H). ^{13}C NMR (100 MHz, $CDCl_3$) δ 173.8, 146.9, 141.5, 140.1, 138.4, 126.9, 124.5, 123.7, 114.9, 42.6, 38.2, 36.0, 35.5, 35.1, 32.4, 31.2, 30.0, 10.0. HRMS (ESI) calcd for $C_{18}H_{26}NO$ [M + H]$^+$: 272.2009, Found: 272.2007.

***N*-(2-(5-phenyl-2,3-dihydro-1*H*-inden-1-yl)ethyl)propionamide (19)**

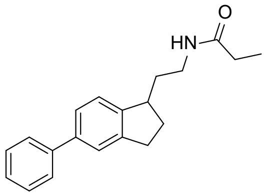

Following the general procedure **E**, **19** was purified as colorless powder (40 mg, 68% yield) by flash column chromatography (ethyl acetate/petroleum ether = 1/3, V/V). Analytical data: ^{1}H NMR (400 MHz, $CDCl_3$) δ 7.57 (d, *J* = 7.6 Hz, 2H), 7.45 – 7.39 (m, 4H), 7.32 (t, *J* = 7.2 Hz, 1H), 7.27 (d, *J* = 5.4 Hz, 1H), 5.50 (br s, 1H), 3.45 – 3.40 (m, 2H), 3.23 – 3.16 (m, 1H), 3.03 – 2.84 (m, 2H), 2.42 – 2.34 (m, 1H), 2.20 (q, *J* = 7.6 Hz, 2H), 2.15 – 2.05 (m, 1H), 1.81 – 1.70 (m, 1H), 1.67 – 1.61 (m, 1H), 1.16 (t, *J* = 7.6 Hz, 3H). ^{13}C NMR (100 MHz, $CDCl_3$) δ 173.9, 146.0, 144.8, 141.6, 140.1, 128.8, 127.3, 127.1, 125.5, 123.9, 123.5, 42.4, 38.1, 35.1, 32.4, 31.6, 29.9, 10.0. HRMS (ESI) calcd for $C_{20}H_{24}NO$ [M + H]$^+$: 294.1852, Found: 294.1851.

***N*-(2-(1,6,7,8-tetrahydro-2*H*-indeno[5,4-b]furan-8-yl)ethyl)propionamide (20)**

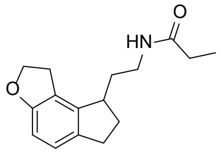

Following the general procedure **E**, **20** was purified as colorless powder (21 mg, 41% yield) by flash column chromatography (ethyl acetate/petroleum ether = 1/1.5, V/V). Analytical data: ^{1}H NMR (400 MHz, $CDCl_3$) δ 6.95 (d, *J* = 8.0 Hz, 1H), 6.61 (d, *J* = 7.9 Hz, 1H), 5.43 (br s, 1H), 4.61 – 4.48 (m, 2H), 3.37 – 3.32 (m, 2H), 3.29 – 3.06 (m, 3H), 2.93 – 2.73 (m, 2H), 2.32 – 2.23 (m, 1H), 2.17 (q, *J* = 7.6 Hz, 2H), 2.06 – 1.98 (m, 1H), 1.86 – 1.78 (m, 1H), 1.67 – 1.59 (m, 1H), 1.14 (t, *J* = 7.6, 1.5 Hz, 3H). ^{13}C NMR (100 MHz, $CDCl_3$) δ 173.8, 159.5, 143.2, 135.9, 123.5, 122.3, 107.6, 71.3, 42.3, 38.2, 33.6, 31.9, 30.8, 29.9, 28.7, 10.0. HRMS (ESI) calcd for $C_{16}H_{22}NO_2$ [M + H]$^+$: 260.1645, Found: 260.1649.

***tert*-butyl(8-(2-propionamidoethyl)-1,6,7,8-tetrahydro-2*H*-indeno[5,4-b]furan-4yl)carbamate (21)**

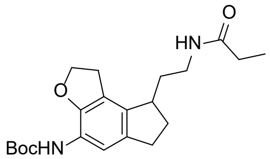

Following the general procedure **E**, **21** was purified as colorless powder (32 mg, 43% yield) by flash column chromatography (ethyl acetate/petroleum ether = 1/1.5, V/V). Analytical data: ^{1}H NMR (600 MHz, $CDCl_3$) δ 7.65 (br s, 1H), 6.53 (br s, 1H), 5.38 (br s, 1H), 4.63 – 4.51 (m, 2H), 3.34 – 3.25 (m, 3H), 3.15 – 3.10 (m, 2H), 2.91 – 2.86 (m, 1H), 2.79 – 2.74 (m, 1H), 2.28 – 2.22 (m, 1H), 2.16 (q, *J* = 7.6 Hz, 2H), 2.00 – 1.94 (m, 1H), 1.80 – 1.75 (m, 1H), 1.61 – 1.58 (m, 1H), 1.50 (s, 9H), 1.14 (t, *J* = 7.6 Hz, 3H). ^{13}C NMR (150 MHz, $CDCl_3$) δ 173.7, 152.9, 147.8, 136.5, 121.5, 121.2, 113.5, 80.3, 71.9, 41.8, 38.0, 33.8, 31.8, 31.2, 29.8, 29.3, 28.3, 9.9. HRMS (ESI) calcd for $C_{21}H_{31}N_2O_4$ [M + H]$^+$: 375.2278, Found: 375.2273.

***N*-(2-(2,2-dimethyl-2,3-dihydro-1*H*-inden-1-yl)ethyl)propionamide (22)**

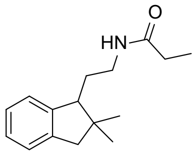

Following the general procedure **E**, **22** was purified as colorless powder (35 mg, 71% yield) by flash column chromatography (ethyl acetate/petroleum ether = 1/3, V/V). Analytical data: ^{1}H NMR (400 MHz, $CDCl_3$) δ 7.21 – 7.12 (m, 4H), 5.49 (br s, 1H), 3.46 – 3.41 (m, 2H), 2.72 – 2.63 (m, 3H), 2.19 (q, *J* = 7.6 Hz, 2H), 1.85 – 1.76 (m, 1H), 1.70 – 1.61 (m, 1H), 1.15 (t, *J* = 7.6 Hz, 3H), 1.12 (s, 3H), 0.96 (s, 3H). ^{13}C NMR (100 MHz, $CDCl_3$) δ 173.8, 146.6, 142.8, 126.5, 126.2, 124.9, 124.4, 52.2, 47.1, 43.8, 38.9, 30.3, 29.9, 28.8, 23.1, 10.0. HRMS (ESI) calcd for $C_{16}H_{24}NO$ [M + H]$^+$: 246.1852, Found: 246.1856.

***N*-(2-(3-methyl-2,3-dihydro-1*H*-inden-1-yl)ethyl)propionamide (23)**

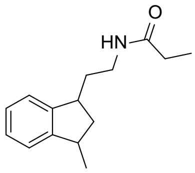

Following the general procedure **E**, **23** was purified as colorless powder (38 mg, 81% yield, d.r. = 1.2:1) by flash column chromatography (ethyl acetate/petroleum ether = 1/2.5, V/V). The reported d.r. was determined by ^{1}H NMR analysis of purified **23** and is consistent with that of the crude reaction mixture. The following analytical data correspond to the mixture of diastereomers. Analytical data: ^{1}H NMR (400 MHz, $CDCl_3$) δ 7.18 (s, 4H), 5.57 (br s, 0.45H), 5.48 (br s, 0.55H), 3.47 – 3.38 (m, 2H), 3.31 – 3.05 (m, 2H), 2.57 – 2.50 (m, 0.6H), 2.25 – 2.15 (m, 2.4H), 2.06 – 2.01 (m, 1H), 1.92 – 1.84 (m, 1H), 1.69 – 1.52 (m, 1H), 1.32 (d, *J* = 6.4 Hz, 1.4H), 1.24 (d, *J* = 6.4 Hz, 1.6H), 1.18 – 1.12 (m, 3H). ^{13}C NMR (100 MHz, $CDCl_3$) δ 173.8, 148.6, 148.5, 146.5, 146.3, 126.9, 126.7, 126.5, 126.4, 124.0, 123.7, 123.2, 123.0, 42.4, 41.3, 41.1, 40.8, 38.2, 38.1, 37.8, 35.3, 34.8, 29.93, 29.90, 20.5, 19.5, 10.0. HRMS (ESI) calcd for $C_{15}H_{22}NO$ [M + H]$^+$: 232.1696, Found:

232.1695.

***N*-(2-(3-phenyl-2,3-dihydro-1*H*-inden-1-yl)ethyl)propionamide (24)**

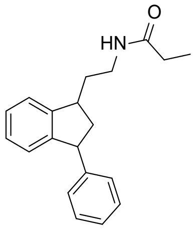

Following the general procedure **E**, **24** was purified as colorless powder (41 mg, 69% yield, d.r. = 1.5:1) by flash column chromatography (ethyl acetate/petroleum ether = 1/2, V/V). The reported d.r. was determined by ^{1}H NMR analysis of purified **24** and is consistent with that of the crude reaction mixture. The following analytical data correspond to the mixture of diastereomers. Analytical data: ^{1}H NMR (400 MHz, $CDCl_3$) δ 7.34 – 7.27 (m, 3H), 7.24 – 7.21 (m, 3H), 7.18 – 7.11 (m, 2H), 7.00 (d, *J* = 7.4 Hz, 0.4H), 6.90 (d, *J* = 7.4 Hz, 1H), 5.53 (br s, 0.6H), 5.46 (br s, 0.4H), 4.44 (t, *J* = 7.4 Hz, 0.4H), 4.25 (t, *J* = 7.4 Hz, 0.4H), 3.51 – 3.29 (m, 2.4H), 3.20 – 3.18 (m, 0.6H), 2.82 – 2.76 (m, 0.6H), 2.37 – 2.30 (m, 1.4H), 2.27 – 2.13 (m, 2H), 2.02 – 1.94 (m, 0.6H), 1.78 – 1.70 (m, 1.4H), 1.18 – 1.12 (m, 3H). ^{13}C NMR (100 MHz, $CDCl_3$) δ 173.9, 173.8, 147.03, 146.96, 146.8, 146.4, 145.4, 144.8, 128.64, 128.62, 128.5, 128.1, 127.2, 127.0, 126.9, 126.8, 126.6, 126.5, 125.5, 125.0, 124.0, 123.0, 50.7, 50.0, 44.1, 42.5, 41.6, 41.5, 38.2, 38.1, 35.3, 34.7, 29.94, 29.92, 10.0. HRMS (ESI) calcd for $C_{20}H_{24}NO$ $[M + H]^+$: 294.1852, Found: 294.1851.

***N*-(2-(1,2,3,4-tetrahydronaphthalen-1-yl)ethyl)propionamide (25)**

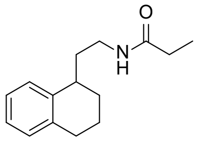

Following the general procedure **E**, **25** was purified as colorless powder (24 mg, 51% yield) by flash column chromatography (ethyl acetate/petroleum ether = 1/2, V/V). Analytical data: ^{1}H NMR (400 MHz, $CDCl_3$) δ 7.15 – 7.05 (m, 4H), 5.40 (br s, 1H), 3.44 – 3.30 (m, 2H), 2.86 – 2.69 (m, 3H), 2.18 (q, *J* = 7.6 Hz, 2H), 1.92 – 1.70 (m, 6H), 1.14 (t, *J* = 7.7 Hz, 3H). ^{13}C NMR (100 MHz, $CDCl_3$) δ 173.8, 140.4, 137.3, 129.4, 128.6, 125.9, 125.8, 37.9, 36.7, 35.6, 29.9, 29.7, 27.7, 19.9, 10.0. HRMS (ESI) calcd for $C_{15}H_{22}NO$ $[M + H]^+$: 232.1696, Found: 232.1698.

***N*-(2-(6-fluoro-1,2,3,4-tetrahydronaphthalen-1-yl)ethyl)propionamide (26)**

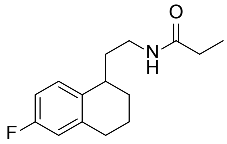

Following the general procedure **E**, **26** was purified as colorless powder (24 mg, 49% yield) by flash column chromatography (ethyl acetate/petroleum ether = 1/1.5, V/V). Analytical data: ^{1}H NMR (400 MHz, $CDCl_3$) δ 7.09 (dd, *J* = 8.6, 5.8 Hz, 1H), 6.83 – 6.73 (m, 2H), 5.47 (br s, 1H), 3.42 – 3.27 (m, 2H), 2.82 – 2.70 (m, 3H), 2.19 (q, *J* = 7.6 Hz, 2H), 1.90 – 1.67 (m, 6H), 1.14 (t, *J* = 7.6 Hz, 3H). ^{13}C NMR (100 MHz, $CDCl_3$) δ 173.9, 160.9 (d, *J* = 242.1 Hz), 139.3 (d, *J* = 7.1 Hz), 135.9 (d, *J* =

3.0 Hz), 129.9 (d, J = 8.0 Hz), 115.3 (d, J = 20.1 Hz), 112.8 (d, J = 21.1 Hz), 37.7, 36.8, 34.9, 29.9, 29.8, 27.5, 19.5, 10.0. ^{19}F NMR (376 MHz, CDCl$_3$) δ -118.0. HRMS (ESI) calcd for $C_{15}H_{21}FNO$ $[M + H]^+$: 250.1602, Found: 250.1604.

***N*-(2-(7-methoxy-1,2,3,4-tetrahydronaphthalen-1-yl)ethyl)propionamide (27)**

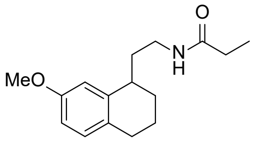

Following the general procedure **E**, **27** was purified as colorless powder (29 mg, 55% yield) by flash column chromatography (ethyl acetate/petroleum ether = 1/1.5, V/V). Analytical data: ^{1}H NMR (400 MHz, CDCl$_3$) δ 6.97 (d, J = 8.2 Hz, 1H), 6.67 (d, J = 8.4 Hz, 2H), 5.40 (br s, 1H), 3.77 (s, 3H), 3.40 – 3.33 (m, 2H), 2.80 (br s, 1H), 2.68 (br s, 2H), 2.17 (q, J = 7.6 Hz, 2H), 1.90 – 1.66 (m, 6H), 1.13 (t, J = 7.6 Hz, 3H). ^{13}C NMR (100 MHz, CDCl$_3$) δ 173.8, 157.7, 141.5, 130.2, 129.4, 113.5, 111.9, 55.4, 37.8, 36.7, 35.8, 30.0, 28.9, 27.7, 20.2, 10.0. HRMS (ESI) calcd for $C_{16}H_{24}NO_2$ $[M + H]^+$: 262.1802, Found: 262.1800.

***N*-(2-(2,3-dihydrobenzofuran-3-yl)ethyl)propionamide (28)**

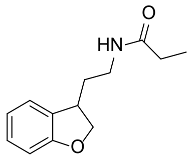

Following the general procedure **E**, reaction for 96 hours, **28** was purified as colorless powder (23 mg, 52% yield) by flash column chromatography (ethyl acetate/petroleum ether = 1/1, V/V). Analytical data: ^{1}H NMR (400 MHz, CDCl$_3$) δ 7.20 (d, J = 7.4 Hz, 1H), 7.13 (t, J = 7.9 Hz, 1H), 6.87 (td, J = 7.4, 0.9 Hz, 1H), 6.80 (d, J = 8.0 Hz, 1H), 5.44 (br s, 1H), 4.64 (t, J = 8.9 Hz, 1H), 4.27 (dd, J = 8.9, 5.9 Hz, 1H), 3.51 – 3.28 (m, 3H), 2.19 (q, J = 7.6 Hz, 2H), 2.01 – 1.93 (m, 1H), 1.83 – 1.74 (m, 1H), 1.14 (t, J = 7.6 Hz, 3H). ^{13}C NMR (100 MHz, CDCl$_3$) δ 173.9, 160.0, 130.2, 128.6, 124.5, 120.7, 109.9, 76.7, 39.8, 37.4, 35.0, 29.9, 10.0. HRMS (ESI) calcd for $C_{13}H_{18}NO_2$ $[M + H]^+$: 220.1332, Found: 220.1328.

***N*-(2-(1-acetylindolin-3-yl)ethyl)propionamide (29)**

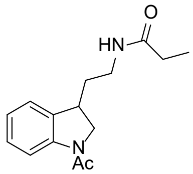

Following the general procedure **E**, reaction for 24 hours, **29** was purified as colorless powder (40 mg, 77% yield) by flash column chromatography (ethyl acetate/petroleum ether = 2/1, V/V). Analytical data: ^{1}H NMR (400 MHz, CDCl$_3$) δ 8.20 (d, J = 8.0 Hz, 1H), 7.22 (t, J = 8.0 Hz, 1H), 7.17 (d, J = 7.2 Hz, 1H), 7.03 (t, J = 7.4 Hz, 1H), 5.54 (br s, 1H), 4.21 (t, J = 10.0 Hz, 1H), 3.77 (dd, J = 10.0, 5.8 Hz, 1H), 3.51 – 3.26 (m, 3H), 2.24 (s, 3H), 2.20 (q, J = 7.6 Hz, 2H), 2.05 – 1.96 (m, 1H), 1.84 – 1.73 (m, 1H), 1.15 (t, J = 7.6 Hz, 3H). ^{13}C NMR (100 MHz, CDCl$_3$) δ 174.1, 168.9, 142.8, 134.3, 128.3, 123.8, 117.2, 55.0, 37.9, 37.1, 35.7, 29.9, 24.4, 10.0. HRMS (ESI) calcd for

$C_{15}H_{21}N_2O_2$ [M + H]$^+$: 261.1598, Found: 261.1596.

***N*-(2-(chroman-4-yl)ethyl)propionamide (30)**

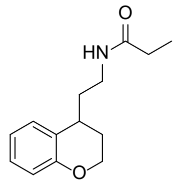

Following the general procedure **E**, reaction for 96 hours, **30** was purified as colorless powder (21 mg, 45% yield) by flash column chromatography (ethyl acetate/petroleum ether = 1/1, V/V). Analytical data: ^{1}H NMR (400 MHz, $CDCl_3$) δ 7.12 – 7.07 (m, 2H), 6.86 (td, *J* = 7.4, 1.2 Hz, 1H), 6.79 (d, *J* = 8.1 Hz, 1H), 5.43 (br s, 1H), 4.23 – 4.15 (m, 2H), 3.47 – 3.34 (m, 2H), 2.90 – 2.83 (m, 1H), 2.19 (q, *J* = 7.6 Hz, 2H), 2.16 – 2.08 (m, 1H), 2.04 – 1.95 (m, 1H), 1.88 – 1.72 (m, 2H), 1.15 (t, *J* = 7.6 Hz, 3H). ^{13}C NMR (100 MHz, $CDCl_3$) δ 173.9, 154.7, 129.0, 127.7, 125.7, 120.4, 117.1, 63.6, 37.3, 36.4, 31.5, 29.9, 27.0, 10.0. HRMS (ESI) calcd for $C_{14}H_{20}NO_2$ [M + H]$^+$: 234.1489, Found: 234.1488.

***N*-(2-(2,3,4,5-tetrahydrobenzo[b]oxepin-5-yl)ethyl)propionamide (31)**

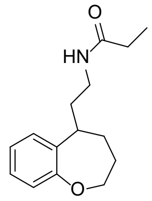

Following the general procedure **E**, reaction for 96 hours, **31** was purified as colorless powder (13 mg, 25% yield) by flash column chromatography (ethyl acetate/petroleum ether = 1/1, V/V). Analytical data: ^{1}H NMR (400 MHz, $CDCl_3$) δ 7.16 – 7.10 (m, 2H), 7.01 – 6.97 (m, 2H), 5.21 (br s, 1H), 4.35 – 4.30 (m, 1H), 3.62 (td, *J* = 11.8, 1.8 Hz, 1H), 3.40 – 3.31 (m, 1H), 3.03 – 2.95 (m, 1H), 2.89 – 2.83 (m, 1H), 2.27 – 2.15 (m, 1H), 2.11 – 2.04 (m, 3H), 1.94 – 1.83 (m, 2H), 1.82 – 1.72 (m, 2H), 1.08 (t, *J* = 7.6 Hz, 3H). ^{13}C NMR (100 MHz, $CDCl_3$) δ 173.6, 159.8, 137.8, 130.3, 128.0, 123.9, 122.3, 73.9, 43.0, 38.6, 32.2, 31.0, 29.9, 27.4, 10.0. HRMS (ESI) calcd for $C_{15}H_{22}NO_2$ [M + H]$^+$: 248.1645, Found: 248.1645.

***N*-(2-(1-methyl-2,3-dihydro-1*H*-inden-1-yl)ethyl)propionamide (32)**

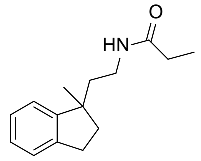

Following the general procedure **E**, **32** was purified as colorless powder (23 mg, 50% yield) by flash column chromatography (ethyl acetate/petroleum ether = 1/2, V/V). Analytical data: ^{1}H NMR (400 MHz, $CDCl_3$) δ 7.21 – 7.12 (m, 4H), 5.14 (br s, 1H), 3.38 – 3.29 (m, 1H), 3.21 – 3.12 (m, 1H), 2.90 (t, *J* = 7.3 Hz, 2H), 2.11 – 2.04 (m, 3H), 1.92 – 1.71 (m, 3H), 1.29 (s, 3H), 1.07 (t, *J* = 7.6 Hz, 3H). ^{13}C NMR (100 MHz, $CDCl_3$) δ 173.5, 150.5, 143.0, 126.6, 126.4, 124.8, 122.5, 46.4, 40.9, 38.5, 36.4, 30.3, 29.7, 27.2, 9.8. HRMS (ESI) calcd for $C_{15}H_{22}NO$ [M + H]$^+$: 232.1696, Found: 232.1694.

N-(2-(1-acetyl-3-methylindolin-3-yl)ethyl)propionamide (33)

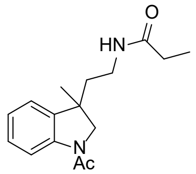

Following the general procedure **E**, reaction for 24 hours, **33** was purified as colorless powder (39 mg, 71% yield) by flash column chromatography (ethyl acetate/petroleum ether = 2/1, V/V). Analytical data: ^{1}H NMR (400 MHz, $CDCl_3$) δ 8.19 (d, J = 8.0 Hz, 1H), 7.25 – 7.20 (m, 1H), 7.12 (d, J = 6.6 Hz, 1H), 7.06 (t, J = 7.4 Hz, 1H), 5.23 (br s, 1H), 4.02 (d, J = 10.4 Hz, 1H), 3.73 (d, J = 10.4 Hz, 1H), 3.38 – 3.29 (m, 1H), 3.04 – 2.95 (m, 1H), 2.24 (s, 3H), 2.06 (q, J = 7.6 Hz, 2H), 1.93 – 1.79 (m, 2H), 1.39 (s, 3H), 1.07 (t, J = 7.6 Hz, 3H). ^{13}C NMR (100 MHz, $CDCl_3$) δ 173.8, 168.9, 142.3, 138.2, 128.4, 124.0, 122.4, 117.3, 61.2, 42.8, 41.4, 36.0, 29.8, 27.3, 24.4, 9.8. HRMS (ESI) calcd for $C_{16}H_{23}N_2O_2$ [M + H]$^+$: 275.1754, Found: 275.1753.

N-(2-(2,3-dihydro-1H-inden-1-yl)ethyl)-3-methylbutanamide (**34**)

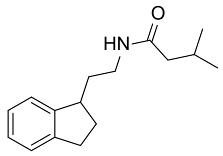

Following the general procedure **E**, **34** was purified as colorless powder (40 mg, 81% yield) by flash column chromatography (ethyl acetate/petroleum ether = 1/3, V/V). Analytical data: ^{1}H NMR (400 MHz, $CDCl_3$) δ 7.22 – 7.14 (m, 4H), 5.43 (br s, 1H), 3.42 – 3.70 (m, 2H), 3.16 – 3.13 (m, 1H), 2.95 – 2.82 (m, 2H), 2.34 – 2.29 (m, 1H), 2.17 – 1.99 (m, 4H), 1.74 – 1.56 (m, 2H), 0.95 (d, J = 6.5 Hz, 6H). ^{13}C NMR (100 MHz, $CDCl_3$) δ 172.6, 146.7, 144.1, 126.7, 126.3, 124.7, 123.6, 46.4, 42.6, 38.1, 35.1, 32.1, 31.6, 26.3, 22.6. HRMS (ESI) calcd for $C_{16}H_{24}NO$ [M + H]$^+$: 246.1852, Found: 246.1853.

N-(2-(2,3-dihydro-1H-inden-1-yl)ethyl)heptanamide (**35**)

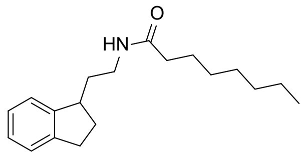

Following the general procedure **E**, **35** was purified as colorless powder (40 mg, 69% yield) by flash column chromatography (ethyl acetate/petroleum ether = 1/8, V/V). Analytical data: ^{1}H NMR (600 MHz, $CDCl_3$) δ 7.22 – 7.14 (m, 4H), 5.42 (br s, 1H), 3.41 – 3.38 (m, 2H), 3.17 – 3.12 (m, 1H), 2.96 – 2.82 (m, 2H), 2.35 – 2.29 (m, 1H), 2.14 (t, J = 7.2 Hz, 2H), 2.08 – 2.03 (m, 1H), 1.74 – 1.68 (m, 1H), 1.66 – 1.58 (m, 2H), 1.29 – 1.26 (m, 9H), 0.88 – 0.86 (m, 3H). ^{13}C NMR (150 MHz, $CDCl_3$) δ 173.2, 146.7, 144.0, 128.5, 126.3, 124.7, 123.6, 42.6, 38.1, 37.1, 35.0, 32.1, 31.8, 31.6, 29.4, 29.1, 25.9, 22.7, 14.2. HRMS (ESI) calcd for $C_{19}H_{30}NO$ [M + H]$^+$: 288.2322, Found: 288.2327.

N-(2-(2,3-dihydro-1H-inden-1-yl)ethyl)cyclopropanecarboxamide (36)

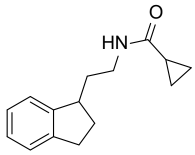

Following the general procedure **E, 36** was purified as colorless powder (20 mg, 43% yield) by flash column chromatography (ethyl acetate/petroleum ether = 1/8, V/V). Analytical data: ^{1}H NMR (400 MHz, $CDCl_3$) δ 7.22 – 7.14 (m, 4H), 5.66 (br s, 1H), 3.44 – 3.39 (m, 2H), 3.19 – 3.12 (m, 1H), 2.97 – 2.80 (m, 2H), 2.37 – 2.28 (m, 1H), 2.12 – 2.04 (m, 1H), 1.76 – 1.58 (m, 2H), 1.33 – 1.25 (m, 1H), 0.98 – 0.95 (m, 2H), 0.74 – 0.70 (m, 2H). ^{13}C NMR (100 MHz, $CDCl_3$) δ 173.6, 146.8, 144.0, 126.7, 126.3, 124.7, 123.6, 42.6, 38.4, 35.1, 32.2, 31.6, 14.9, 7.2. HRMS (ESI) calcd for $C_{15}H_{20}NO$ $[M + H]^+$: 230.1539, Found: 230.1540.

N-(2-(2,3-dihydro-1H-inden-1-yl)ethyl)-2-phenylacetamide (37)

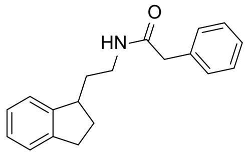

Following the general procedure **E**, **37** was purified as colorless powder (54 mg, 97% yield) by flash column chromatography (ethyl acetate/petroleum ether = 1/5, V/V). Analytical data: ^{1}H NMR (400 MHz, $CDCl_3$) δ 7.47 – 6.88 (m, 8H), 5.40 (br s, 1H), 3.58 (s, 2H), 3.38 – 3.32 (m, 2H), 3.09 – 3.01 (m, 1H), 2.93 – 2.77 (m, 2H), 2.28 – 2.20 (m, 1H), 2.01 – 1.93 (m, 1H), 1.69 – 1.48 (m, 2H). ^{13}C NMR (100 MHz, $CDCl_3$) δ 171.1, 146.6, 144.0, 135.1, 129.6, 129.2, 127.5, 126.7, 126.3, 124.7, 123.6, 44.1, 42.6, 38.4, 34.8, 32.1, 31.5. HRMS (ESI) calcd for $C_{19}H_{22}NO$ $[M + H]^+$: 280.1696, Found: 280.1699.

N-(2-(2,3-dihydro-1H-inden-1-yl)ethyl)-3-phenylpropanamide (38)

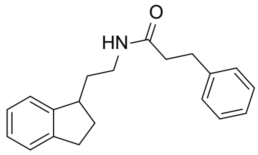

Following the general procedure **E**, **38** was purified as colorless powder (42 mg, 72% yield) by flash column chromatography (ethyl acetate/petroleum ether = 1/3, V/V). Analytical data: ^{1}H NMR (400 MHz, $CDCl_3$) δ 7.29 – 7.15 (m, 8H), 5.34 (br s, 1H), 3.38 – 3.32 (m, 2H), 3.9 – 3.01 (m, 1H), 2.96 (t, J = 7.9 Hz, 2H), 2.94 – 2.79 (m, 2H), 2.46 (t, J = 7.9 Hz, 2H), 2.31 – 2.23 (m, 1H), 2.02 – 1.94 (m, 1H), 1.70 – 1.64 (m, 2H), 1.62 – 1.49 (m, 1H). ^{13}C NMR (100 MHz, $CDCl_3$) δ 172.1, 146.7, 144.0, 141.0, 128.7, 128.5, 126.7, 126.4, 126.3, 124.7, 123.6, 42.5, 38.7, 38.1, 34.8, 32.1, 31.9, 31.5. HRMS (ESI) calcd for $C_{20}H_{24}NO$ $[M + H]^+$: 294.1852, Found: 294.1851.

***tert*-butyl (2-(2,3-dihydro-1H-inden-1-yl)ethyl)carbamate (39)**

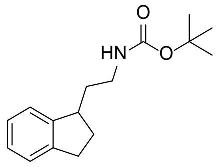

Following the general procedure **E**, **39** was purified as colorless powder (44 mg, 85% yield) by flash column chromatography (ethyl acetate/petroleum ether = 1/5, V/V). Analytical data: ^{1}H NMR (600 MHz, $CDCl_3$) δ 7.25 – 7.14 (m, 4H), 4.56 (br s, 1H), 3.27 – 3.26 (m, 2H), 3.17 – 3.12 (m, 1H), 2.96 – 2.82 (m, 2H), 2.37 – 2.29 (m, 1H), 2.05 – 2.04 (m, 1H), 1.74 – 1.68 (m, 1H), 1.62 – 1.56 (m, 1H), 1.45 (s, 9H). ^{13}C NMR (150 MHz, $CDCl_3$) δ 156.1, 146.9, 144.0, 126.6, 126.3, 124.7, 123.6, 79.3, 42.6, 39.3, 35.5, 32.2, 31.6, 28.6. HRMS (ESI) calcd for $C_{16}H_{23}NO_2Na$ $[M + Na]^+$: 284.1621, Found: 284.1627.

benzyl (2-(2,3-dihydro-1*H*-inden-1-yl)ethyl)carbamate (**40**)

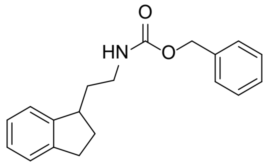

Following the general procedure **E**, **40** was purified as colorless powder (30 mg, 51% yield) by flash column chromatography (ethyl acetate/petroleum ether = 1/10, V/V). Analytical data: ^{1}H NMR (400 MHz, $CDCl_3$) δ 7.37 – 7.24 (m, 5H), 7.23 – 7.15 (m, 4H), 5.12 (s, 2H), 4.81 (br s, 1H), 3.35 – 3.32 (m, 2H), 3.19 – 3.12 (m, 1H), 2.97 – 2.81 (m, 2H), 2.36 – 2.28 (m, 1H), 2.12 – 2.04 (m, 1H), 1.75 – 1.57 (m, 2H). ^{13}C NMR (100 MHz, $CDCl_3$) δ 156.4, 146.6, 143.9, 136.6, 128.6, 128.1, 126.5, 126.2, 124.6, 123.5, 66.7, 42.3, 39.6, 35.2, 32.1, 31.4. HRMS (ESI) calcd for $C_{19}H_{22}NO_2$ $[M + H]^+$: 296.1645, Found: 296.1650.

***N*-(2-(2,3-dihydro-1*H*-inden-1-yl)ethyl)benzamide** (**41**)

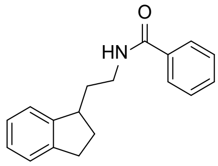

Following the general procedure **E**, **41** was purified as colorless powder (36 mg, 68% yield) by flash column chromatography (ethyl acetate/petroleum ether = 1/8, V/V). Analytical data: ^{1}H NMR (400 MHz, $CDCl_3$) δ 7.73 (d, J = 7.4 Hz, 2H), 7.49 (t, J = 7.3 Hz, 1H), 7.41 (d, J = 7.8 Hz, 2H), 7.23 – 7.22 (m, 2H), 7.18 – 7.16 (m, 2H), 6.26 (br s, 1H), 3.63 – 3.58 (m, 2H), 3.26 – 3.19 (m, 1H), 2.99 – 2.83 (m, 2H), 2.41 – 2.24 (m, 1H), 2.22 – 2.15 (m, 1H), 1.81 – 1.70 (m, 2H). ^{13}C NMR (100 MHz, $CDCl_3$) δ 167.6, 146.7, 144.1, 134.8, 131.5, 128.7, 126.9, 126.7, 126.3, 124.7, 123.6, 42.7, 38.6, 34.8, 32.1, 31.6. HRMS (ESI) calcd for $C_{18}H_{20}NO$ $[M + H]^+$: 266.1539, Found: 266.1538.

***N*-(2-(2,3-dihydro-1*H*-inden-1-yl)ethyl)-4-methoxybenzamide** (**42**)

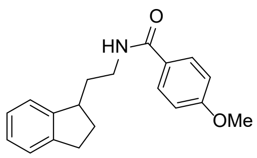

Following the general procedure **E**, **42** was purified as colorless powder (42 mg, 71% yield) by flash column chromatography (ethyl acetate/petroleum ether = 1/5, V/V). Analytical data: ^{1}H NMR (400 MHz, $CDCl_3$) δ 7.70 – 7.68 (m, 2H), 7.24 – 7.22 (m, 2H), 7.17 – 7.15 (m, 2H), 6.91 – 6.89 (m, 2H), 6.10 (br s, 1H), 3.84 (s, 3H), 3.61 – 3.56 (m, 2H), 3.26 – 3.19 (m, 1H), 2.99 – 2.82 (m, 2H), 2.40 – 2.32 (m, 1H), 2.22 – 2.14 (m, 1H), 1.81 – 1.70 (m, 2H). ^{13}C NMR (100 MHz, $CDCl_3$) δ 167.1, 162.2,

146.8, 144.1, 128.7, 127.1, 126.7, 126.4, 124.8, 123.6, 113.9, 55.5, 42.8, 38.6, 35.0, 32.1, 31.6. HRMS (ESI) calcd for $C_{19}H_{22}NO_2$ $[M + H]^+$: 296.1645, Found: 296.1649.

***N*-(2-(2,3-dihydro-1*H*-inden-1-yl)ethyl)-3-(trifluoromethyl)benzamide** (**43**)

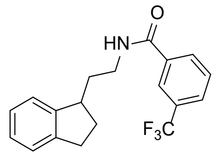

Following the general procedure **E**, **43** was purified as colorless powder (41 mg, 62% yield) by flash column chromatography (ethyl acetate/petroleum ether = 1/15, V/V). Analytical data: ^{1}H NMR (400 MHz, $CDCl_3$) δ 7.96 (s, 1H), 7.90 (d, *J* = 7.8 Hz, 1H), 7.74 (d, *J* = 7.8 Hz, 1H), 7.55 (t, *J* = 7.8 Hz, 1H), 7.24 – 7.16 (m, 4H), 6.21 (br s, 1H), 3.67 – 3.56 (m, 2H), 3.29 – 3.21 (m, 1H), 3.01 – 2.84 (m, 2H), 2.42 – 2.33 (m, 1H), 2.24 – 2.16 (m, 1H), 1.84 – 1.74 (m, 2H). ^{13}C NMR (100 MHz, $CDCl_3$) δ 166.1, 146.5, 144.1, 135.6, 131.2 (d, *J* = 33.0 Hz), 130.3, 129.3, 128.1 (d, *J* = 3.7 Hz), 126.8, 126.4, 124.8, 123.9 (q, *J* = 3.8 Hz), 123.8 (d, *J* = 272.6 Hz), 123.6, 42.7, 38.8, 34.7, 32.0, 31.6. ^{19}F NMR (376 MHz, $CDCl_3$) δ -62.7. HRMS (ESI) calcd for $C_{19}H_{19}F_3NO$ $[M + H]^+$: 334.1413, Found: 334.1415.

***N*-(2-(2,3-dihydro-1*H*-inden-1-yl)ethyl)nicotinamide** (**44**)

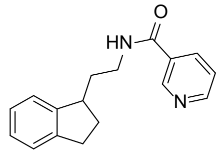

Following the general procedure **E**, **44** was purified as colorless powder (37 mg, 70% yield) by flash column chromatography (ethyl acetate/petroleum ether = 1/1, V/V). Analytical data: ^{1}H NMR (600 MHz, $CDCl_3$) δ 8.87 (s, 1H), 8.71 (d, *J* = 4.6 Hz, 1H), 8.05 (d, *J* = 7.9 Hz, 1H), 7.37 (dd, *J* = 7.9, 4.8 Hz, 1H), 7.23 – 7.15 (m, 4H), 6.12 (br s, 1H), 3.67 – 3.59 (m, 2H), 3.27 – 3.22 (m, 1H), 2.99 – 2.85 (m, 2H), 2.39 – 2.34 (m, 1H), 2.22 – 2.16 (m, 1H), 1.83 – 1.75 (m, 2H). ^{13}C NMR (150 MHz, $CDCl_3$) δ 165.7, 152.4, 147.8, 146.5, 144.1, 135.2, 130.4, 126.8, 126.4, 124.9, 123.7, 123.6, 42.7, 38.8, 34.7, 32.0, 31.6. HRMS (ESI) calcd for $C_{17}H_{19}N_2O$ $[M + H]^+$: 267.1492, Found: 267.1496.

***N*-(1-(2,3-dihydro-1*H*-inden-1-yl)propan-2-yl)benzamide** (**45**)

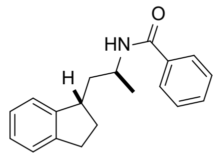

Following the general procedure **E**, **45** was purified as colorless powder (51 mg, 92% yield, d.r. = 4:1) by flash column chromatography (ethyl acetate/petroleum ether = 1/10, V/V). The reported d.r. was determined by ^{1}H NMR analysis of purified **45** and is consistent with that of the crude reaction mixture. The following analytical data correspond to the mixture of diastereomers. Analytical data: ^{1}H NMR (400 MHz, $CDCl_3$) δ 7.79 – 7.68 (m, 2H), 7.52 – 7.39 (m, 3H), 7.29 – 7.13 (m, 4H), 5.95 (d, *J* = 8.9 Hz, 1H), 4.52 – 4.38 (m, 1H), 3.27 – 3.18 (m, 1H), 2.99 – 2.79 (m, 2H), 2.50 – 2.30 (m, 1H), 2.13 – 1.98 (m, 1H), 1.86 – 1.59 (m, 2H), 1.33 (d, *J* = 7.4 Hz, 3H). ^{13}C NMR (100 MHz, $CDCl_3$) δ 167.1, 166.8, 147.0, 144.3, 144.0, 135.1, 131.53, 131.46, 128.73, 128.67, 127.0, 126.9, 126.64,

126.59, 126.4, 126.2, 124.74, 124.70, 123.69, 123.2, 44.8, 44.4, 43.0, 42.5, 42.3, 42.0, 32.9, 32.5, 31.60, 31.56, 22.6, 21.4. HRMS (ESI) calcd for $C_{19}H_{22}NO$ [M + H]$^+$: 280.1696, Found: 280.1699.

***N*-(1-(2,3-dihydro-1*H*-inden-1-yl)-4-methylpentan-2-yl)benzamide (46)**

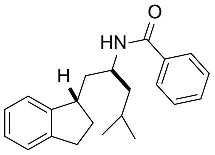

Following the general procedure **E**, **46** was purified as colorless powder (58 mg, 90% yield, d.r. = 4:1) by flash column chromatography (ethyl acetate/petroleum ether = 1/20, V/V). The reported d.r. was determined by ^{1}H NMR analysis of purified **46** and is consistent with that of the crude reaction mixture. The following analytical data correspond to the mixture of diastereomers. Analytical data: ^{1}H NMR (400 MHz, $CDCl_3$) δ 7.86 – 7.60 (m, 2H), 7.55 – 7.30 (m, 3H), 7.24 – 7.08 (m, 4H), 5.80 (d, J = 9.5 Hz, 1H), 4.56 – 4.39 (m, 1H), 3.28 – 3.15 (m, 1H), 2.99 – 2.73 (m, 2H), 2.65 – 2.21 (m, 1H), 2.09 – 1.94 (m, 1H), 1.81 – 1.60 (m, 3H), 1.57 – 1.36 (m, 2H), 1.04 – 0.89 (m, 6H). ^{13}C NMR (100 MHz, $CDCl_3$) δ 167.2, 147.2, 144.4, 143.9, 135.1, 131.5, 131.4, 128.74, 128.70, 128.5, 128.4, 127.0, 126.9, 126.5, 126.4, 126.2, 124.69, 124.66, 123.9, 123.1, 47.2, 46.6, 46.3, 45.4, 42.3, 42.0, 41.9, 41.8, 33.0, 32.9, 31.62, 31.59, 29.9, 25.3, 25.2, 23.6, 23.1, 22.7, 22.4. HRMS (ESI) calcd for $C_{22}H_{28}NO$ [M + H]$^+$: 322.2165, Found: 322.2166.

***N,N'*-(6-(2,3-dihydro-1*H*-inden-1-yl)hexane-1,5-diyl)dibenzamide (47)**

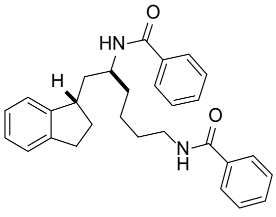

Following the general procedure **E**, **47** was purified as colorless powder (51 mg, 58% yield, d.r. = 2.3:1) by flash column chromatography (ethyl acetate/petroleum ether = 1/2, V/V). The reported d.r. was determined by ^{1}H NMR analysis of purified **47** and is consistent with that of the crude reaction mixture. The following analytical data correspond to the mixture of diastereomers. Analytical data: ^{1}H NMR (400 MHz, $CDCl_3$) δ 7.74 – 7.72 (m, 4H), 7.48 – 7.33 (m, 6H), 7.22 – 7.13 (m, 4H), 6.50 – 6.49 (m, 1H), 6.13 – 6.11 (m, 1H), 4.45 – 4.36 (m, 1H), 3.56 – 3.35 (m, 2H), 3.24 – 3.17 (m, 1H), 2.94 – 2.77 (m, 2H), 2.49 – 2.41 (m, 1H), 2.12 – 2.01 (m, 1H), 1.80 – 1.47 (m, 8H). ^{13}C NMR (100 MHz, $CDCl_3$) δ 168.0, 167.9, 147.0, 144.3, 134.8, 131.6, 131.3, 128.7, 128.63, 128.57, 127.1, 127.0, 126.6, 126.4, 126.2, 124.7, 123.8, 123.1, 48.3, 47.9, 42.3, 41.9, 41.5, 41.3, 39.7, 36.3, 35.3, 32.8, 31.5, 29.8, 29.1, 23.2. HRMS (ESI) calcd for $C_{29}H_{33}N_2O_2$ [M + H]$^+$: 441.2537, Found: 441.2537.

tert-butyl _(S)_-2-((2-(2,3-dihydro-1_H_-inden-1-yl)ethyl)carbamoyl)pyrrolidine-1-carboxylate (48)

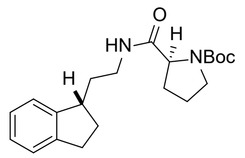

Following the general procedure **E**, **48** was purified as colorless powder (62 mg, 86% yield, d.r. = 1.2:1) by flash column chromatography (ethyl acetate/petroleum ether = 1/2.5, V/V). $[\alpha]_D^{20}$ + 77.0 (*c* 1.0, $CHCl_3$). The reported d.r. was determined by ^{1}H NMR analysis of purified **48** and is consistent with that of the crude reaction mixture. The following analytical data correspond to the mixture of diastereomers. Analytical data: ^{1}H NMR (600 MHz, $CDCl_3$) δ 7.22 – 7.14 (m, 4H), 7.03 (br s, 0.55H), 6.10 (br s, 0.45H), 4.28 – 4.22 (m, 1H), 3.37 – 3.36 (m, 4H), 3.16 – 3.11 (m, 1H), 2.95 – 2.90 (m, 1H), 2.86 – 2.81 (m, 1H), 2.38 – 2.32 (m, 2H), 2.17 – 2.01 (m, 2H), 1.95 – 1.82 (m, 2H), 1.73 – 1.67 (m, 1H), 1.63 – 1.55 (m, 1H), 1.44 (s, 9H). ^{13}C NMR (150 MHz, $CDCl_3$) δ 172.7, 172.0, 156.1, 154.9, 146.8, 144.00, 143.97, 126.6, 126.3, 124.6, 123.6, 80.5, 61.5, 60.1, 47.2, 42.50, 42.49, 38.0, 34.8, 32.22, 32.16, 31.51, 31.50, 31.1, 28.5, 28.0, 24.7, 23.4. HRMS (ESI) calcd for $C_{21}H_{31}N_2O_3$ $[M + H]^+$: 359.2329, Found: 359.2331.

tert-butyl ((2_S_)-1-((2-(2,3-dihydro-1_H_-inden-1-yl)ethyl)amino)-1-oxopropan-2-yl)carbamate (49)

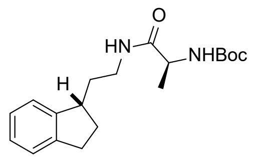

Following the general procedure **E**, **49** was purified as colorless powder (52 mg, 78% yield, d.r. = 1:1) by flash column chromatography (ethyl acetate/petroleum ether = 1/3, V/V). $[\alpha]_D^{20}$ – 55.2 (*c* 1.0, $CHCl_3$). The reported d.r. was determined by ^{1}H NMR analysis of purified **49** and is consistent with that of the crude reaction mixture. The following analytical data correspond to the mixture of diastereomers. Analytical data: ^{1}H NMR (400 MHz, $CDCl_3$) δ 7.24 – 7.08 (m, 4H), 6.22 (br s, 1H), 4.92 (br s, 1H), 4.12 – 4.10 (m, 1H), 3.42 – 3.36 (m, 2H), 3.17 – 3.10 (m, 1H), 2.96 – 2.78 (m, 2H), 2.35 – 2.27 (m, 1H), 2.10 – 2.02 (m, 1H), 1.74 – 1.56 (m, 2H), 1.43 (s, 9H), 1.34 (d, *J* = 6.9 Hz, 3H). ^{13}C NMR (100 MHz, $CDCl_3$) δ 172.6, 155.8, 153.0, 146. 7, 144.02, 144.00, 126.7, 126.3, 124.7, 123.63, 126.61, 80.3, 42.5, 42.5, 38.1, 34.8, 32.1, 31.5, 28.5, 18.3. HRMS (ESI) calcd for $C_{19}H_{29}N_2O_3$ $[M + H]^+$: 333.2173, Found: 333.2174.

tert-butyl (2_S_)-1-((2-(2,3-dihydro-1_H_-inden-1-yl)ethyl)amino)-4-methyl-1-oxopentan-2-yl)carbamate (50)

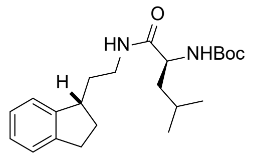

Following the general procedure **E**, **50** was purified as colorless powder (55 mg, 74% yield, d.r. = 1:1) by flash column chromatography (ethyl acetate/petroleum ether = 1/5, V/V). $[\alpha]_D^{20}$ – 20.7 (*c* 1.0, $CHCl_3$). The reported d.r. was determined by ^{1}H NMR analysis of purified **50** and is consistent

with that of the crude reaction mixture. The following analytical data correspond to the mixture of diastereomers. Analytical data: ^{1}H NMR (400 MHz, $CDCl_3$) δ 7.22 – 7.14 (m, 4H), 6.18 (br s, 1H), 4.83 (br s, 1H), 4.05 – 4.04 (m, 1H), 3.41 – 3.36 (m, 2H), 3.17 – 3.10 (m, 1H), 2.96 – 2.79 (m, 2H), 2.35 – 2.27 (m, 1H), 2.10 – 2.01 (m, 1H), 1.75 – 1.58 (m, 5H), 1.42 (s, 9H), 0.94 – 0.92 (m, 6H). ^{13}C NMR (100 MHz, $CDCl_3$) δ 172.4, 155.8, 146.6, 143.89, 143.87, 128.4, 126.5, 126.2, 124.6, 123.52, 123.50, 80.1, 53.2, 42.4, 41.2, 37.9, 34.7, 32.0, 31.4, 28.3, 24.8, 22.9, 22.1. HRMS (ESI) calcd for $C_{22}H_{35}N_2O_3$ [M + H]$^+$: 375.2642, Found: 375.2645.

***tert*-butyl (2*S*)-1-((2-(2,3-dihydro-1*H*-inden-1-yl)ethyl)amino)-1-oxo-3-phenylpropan-2-yl)carbamate (51)**

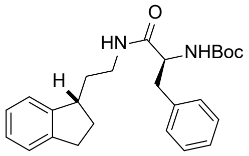

Following the general procedure **E**, **51** was purified as colorless powder (41 mg, 50% yield, d.r. = 1:1) by flash column chromatography (ethyl acetate/petroleum ether = 1/5, V/V). $[\alpha]_D^{20}$ + 13.4 (*c* 1.0, $CHCl_3$). The reported d.r. was determined by ^{1}H NMR analysis of purified **51** and is consistent with that of the crude reaction mixture. The following analytical data correspond to the mixture of diastereomers. Analytical data: ^{1}H NMR (400 MHz, $CDCl_3$) δ 7.31 – 7.13 (m, 9H), 5.69 (br s, 1H), 5.04 (br s, 1H), 4.27 – 4.24 (m, 1H), 3.32 – 3.27 (m, 2H), 3.12 – 2.99 (m, 3H), 2.93 – 2.79 (m, 2H), 2.27 – 2.22 (m, 1H), 1.90 (br s, 1H), 1.66 – 1.56 (m, 1H), 1.47 – 1.45 (m, 1H), 1.40 (s, 9H). ^{13}C NMR (101 MHz, $CDCl_3$) δ 171.1, 155.4, 146.4, 143.8, 129.3, 128.7, 127.0, 126.5, 126.2, 124.5, 123.5, 123.4, 42.3, 38.7, 37.9, 34.5, 31.9, 31.4, 28.3. HRMS (ESI) calcd for $C_{25}H_{33}N_2O_3$ [M + H]$^+$: 409.2486, Found: 409.2482.

***tert*-butyl ((2*S*)-1-((1-(2,3-dihydro-1*H*-inden-1-yl)propan-2-yl)amino)-3-methyl-1-oxobutan-2-yl)carbamate (52)**

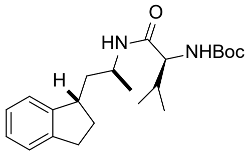

Following the general procedure **E**, **52** was purified as colorless powder (51 mg, 68% yield, d.r. = 1.5:1) by flash column chromatography (ethyl acetate/petroleum ether = 1/8, V/V). $[\alpha]_D^{20}$ – 9.3 (*c* 1.0, $CHCl_3$).The reported d.r. was determined by ^{1}H NMR analysis of purified **52** and is consistent with that of the crude reaction mixture. The following analytical data correspond to the mixture of diastereomers. Analytical data: ^{1}H NMR (400 MHz, $CDCl_3$) δ 7.24 – 7.12 (m, 4H), 5.80 (br s, 1H), 5.06 – 4.98 (m, 1H), 4.24 – 4.11 (m, 1H), 3.84 – 3.76 (m, 1H), 3.15 – 3.10 (m, 1H), 2.96 – 2.76 (m, 2H), 2.41 – 2.25 (m, 1H), 2.12 – 2.11 (m, 1H), 2.01 – 1.94 (m, 0.6H), 1.90 – 1.82 (m, 0.4H), 1.75 – 1.47 (m, 2H), 1.44 – 1.40 (m, 9H), 1.22 – 1.20 (m, 3H), 0.98 – 0.85 (m, 6H). ^{13}C NMR (100 MHz, $CDCl_3$) δ 171.1, 170.8, 156.1, 147.0, 144.2, 143.9, 128.5, 128.4, 126.64, 126.61, 126.57, 126.5, 126.3, 126.2, 124.7, 124.6, 123.8, 123.3, 123.2, 80.1, 44.3, 44.2, 43.9, 42.7, 42.5, 42.3, 42.0, 41.9, 41.8, 32.64, 32.59, 32.5, 31.5, 30.8, 30.7, 28.44, 28.42, 22.5, 21.3, 19.6, 18.0. HRMS (ESI) calcd for $C_{22}H_{35}N_2O_3$ [M + H]$^+$: 375.2642, Found: 375.2637.

***tert*-butyl ((2*S*)-1-((1-(2,3-dihydro-1*H*-inden-1-yl)-4-methylpentan-2-yl)amino)-4-methyl-1-oxopentan-2-yl)carbamate (53)**

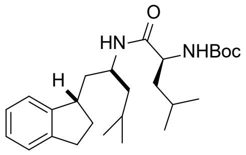

Following the general procedure **E**, **53** was purified as colorless powder (70 mg, 81% yield, d.r. = 1.5:1) by flash column chromatography (ethyl acetate/petroleum ether = 1/17, V/V). The reported d.r. was determined by ^{1}H NMR analysis of purified **53** and is consistent with that of the crude reaction mixture. The following analytical data correspond to the mixture of diastereomers. Analytical data: ^{1}H NMR (400 MHz, $CDCl_3$) δ 7.31 – 7.13 (m, 4H), 5.91 – 5.84 (m, 1H), 4.83 (br s, 0.6H), 4.74 (br s, 0.4H), 4.22 – 4.18 (m, 1H), 4.06 – 4.00 (m, 1H), 3.15 – 3.08 (m, 1H), 2.96 – 2.76 (m, 2H), 2.46 – 2.38 (m, 0.6H), 2.34 – 2.23 (m, 0.4H), 1.92 – 1.85 (m, 1H), 1.72 – 1.60 (m, 3H), 1.49 – 1.28 (m, 14H), 0.94 – 0.87 (m, 12H). ^{13}C NMR (100 MHz, $CDCl_3$) δ 172.0, 171.7, 155.9, 147.3, 144.3, 143.9, 126.5, 126.44, 126.38, 126.3, 126.1, 124.5, 124.0, 123.2, 80.2, 46.5, 46.0, 45.0, 42.0, 41.9, 41.7, 41.6, 40.8, 32.9, 32.7, 31.5, 28.4, 28.3, 25.1, 25.0, 24.9, 23.6, 23.5, 23.1, 22.9, 22.4, 22.1. HRMS (ESI) calcd for $C_{26}H_{43}N_2O_3$ [M + H]$^+$: 431.3268, Found: 431.3268.

Supplementary Note 8. Identification of enatioselective arylalkylation compounds

(*S*)-*N*-(2-(2,3-dihydro-1*H*-inden-1-yl)ethyl)propionamide ((*S*)-3)

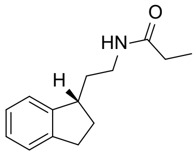

Following the general procedure **F**, **(*S*)-3** was purified as colorless powder (24 mg, 55% yield, 95:5 e.r.) by flash column chromatography (ethyl acetate/petroleum ether = 1/1.5, V/V), the NMR and HRMS (ESI) was absolutely in consistent with **3**. **(*S*)-3**: $[\alpha]_D^{20}$ – 8.4 (*c* 1.0, $CHCl_3$). The enantiomeric ratio was determined by Daicel Chiralpak AS-H (4.6 mm × 250 mm), Hexanes/IPA = 80/20, 0.5 mL/min, λ = 210 nm, t_R (major) = 17.24 min, t_R (minor) = 15.38 min

Supplementary Figure 10. HPLC chromatographs of compound **3**

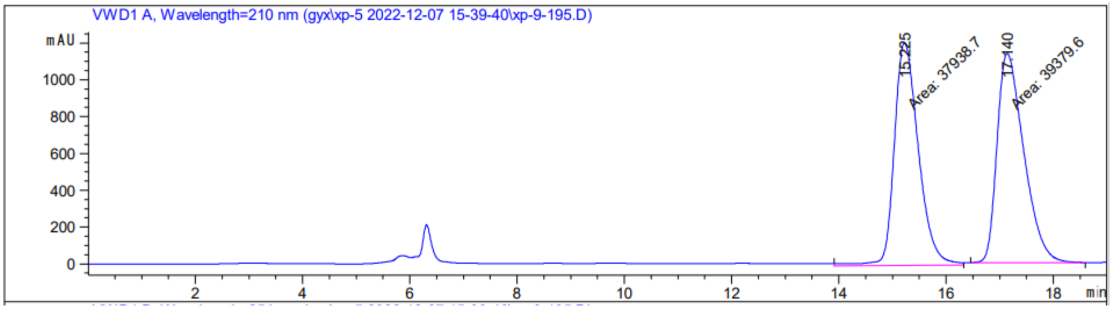

No.	Retention Time min	Area mAU*s	Height mAU	Relative Area %
1	15.225	3.79387e4	1208.82373	49.0682
2	17.140	3.93796e4	1136.68372	50.9318

Supplementary Figure 11. HPLC chromatographs of compound **(*S*)-3**

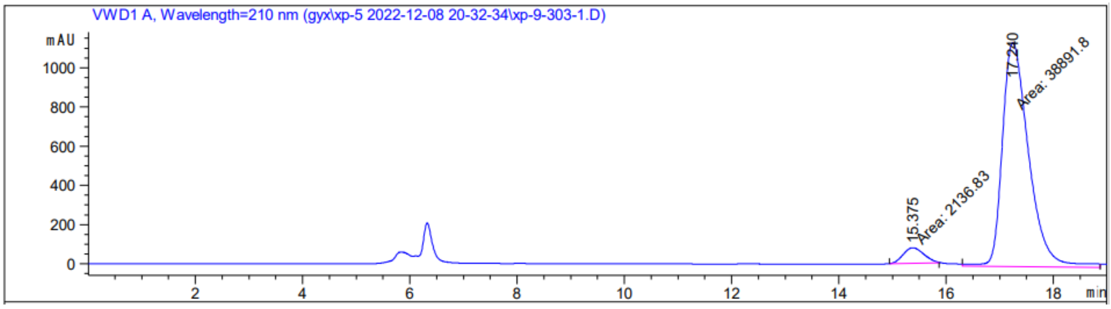

No.	Retention Time min	Area mAU*s	Height mAU	Relative Area %
1	15.375	2136.83008	79.57676	5.2081
2	17.240	3.88918e4	1140.33435	94.7919

(*S*)-*N*-(2-(4-(difluoromethyl)-2,3-dihydro-1*H*-inden-1-yl)ethyl)propionamide ((*S*)-9)

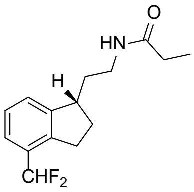

Following the general procedure **F**, **(*S*)-9** was purified as colorless powder (21 mg, 40% yield, 85:15 e.r.) by flash column chromatography (ethyl acetate/petroleum ether = 1/2, V/V) , the NMR and HRMS (ESI) was absolutely in consistent with **9**. (*S*)-**9**: $[\alpha]_D^{20}$ – 5.2 (*c* 1.0, $CHCl_3$). The enantiomeric ratio was determined by Daicel Chiralpak AS-H (4.6 mm × 250 mm), Hexanes/IPA = 80/20, 0.5 mL/min, λ = 210 nm, t_R (major) = 19.22 min, t_R (minor) = 16.73 min.

Supplementary Figure 12. HPLC chromatographs of compound **9**

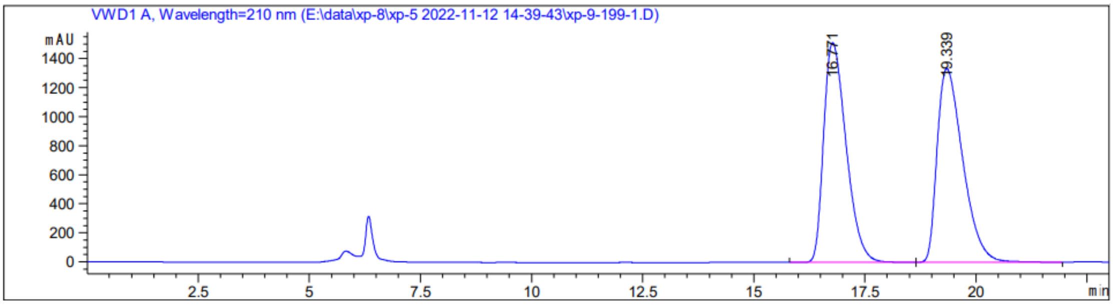

No.	Retention Time<br>min	Area<br>mAU*s	Height<br>mAU	Relative Area<br>%
1	16.771	5.31840e4	1512.52051	49.8220
2	19.339	5.35640e4	1333.24182	50.1780

Supplementary Figure 13. HPLC chromatographs of compound **(*S*)-9**

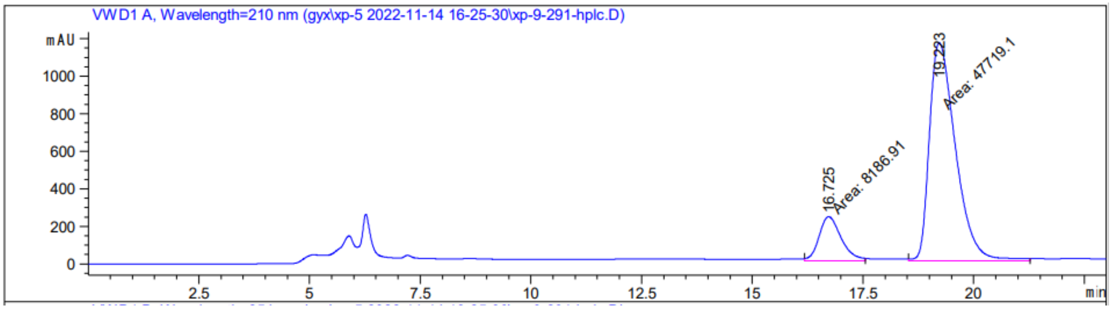

No.	Retention Time<br>min	Area<br>mAU*s	Height<br>mAU	Relative Area<br>%
1	16.725	8186.90967	234.88469	14.6441
2	19.223	4.77191e4	1160.48804	85.3559

(*S*)-*N*-(2-(6-(tert-butyl)-2,3-dihydro-1H-inden-1-yl)ethyl)propionamide ((*S*)-14)

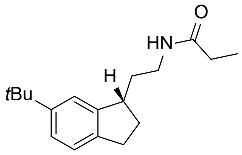

Following the general procedure **F**, **(*S*)-14** was purified as colorless powder (37 mg, 68% yield, 96:4 e.r.) by flash column chromatography (ethyl acetate/petroleum ether = 1/2, V/V), the NMR and HRMS (ESI) was absolutely in consistent with **14**. **(*S*)-14**: $[\alpha]_D^{20}$ – 7.9 (*c* 1.0, $CHCl_3$). The enantiomeric ratio was determined by Daicel Chiralpak AS-H (4.6 mm × 250 mm), Hexanes/IPA = 90/10, 0.5 mL/min, λ = 210 nm, t_R (major) = 22.48 min, t_R (minor) = 18.00 min.

Supplementary Figure 14. HPLC chromatographs of compound **14**

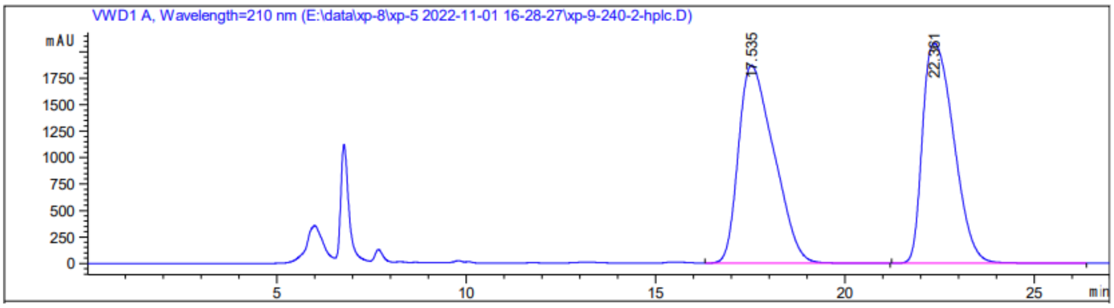

No.	Retention Time min	Area mAU*s	Height mAU	Relative Area %
1	17.535	1.21552e5	1866.58984	50.5833
2	22.361	1.18748e5	2077.80933	49.4167

Supplementary Figure 15. HPLC chromatographs of compound **(*S*)-14**

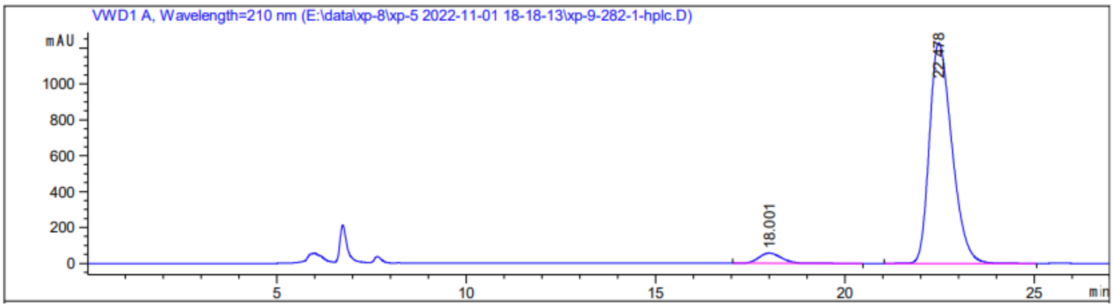

No.	Retention Time min	Area mAU*s	Height mAU	Relative Area %
1	18.001	2383.37256	57.04013	4.4817
2	22.478	5.07972e4	1226.62268	95.5183

(*S*)-*N*-(2-(6-(but-3-en-1-yl)-2,3-dihydro-1*H*-inden-1-yl)ethyl)propionamide ((*S*)-18)

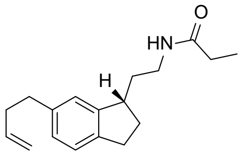

Following the general procedure **F**, **(*S*)-18** was purified as colorless powder (34 mg, 62% yield, 96:4 e.r.) by flash column chromatography (ethyl acetate/petroleum ether = 1/3, V/V) , the NMR and HRMS (ESI) was absolutely in consistent with **18**. **(*S*)-18**: $[\alpha]_D^{20}$ – 7.8 (*c* 1.0, $CHCl_3$). The enantiomeric ratio was determined by Daicel Chiralpak AS-H (4.6 mm × 250 mm), Hexanes/IPA = 90/10, 0.5 mL/min, λ = 210 nm, t_R (major) = 32.78 min, t_R (minor) = 26.05 min.

Supplementary Figure 16. HPLC chromatographs of compound **18**

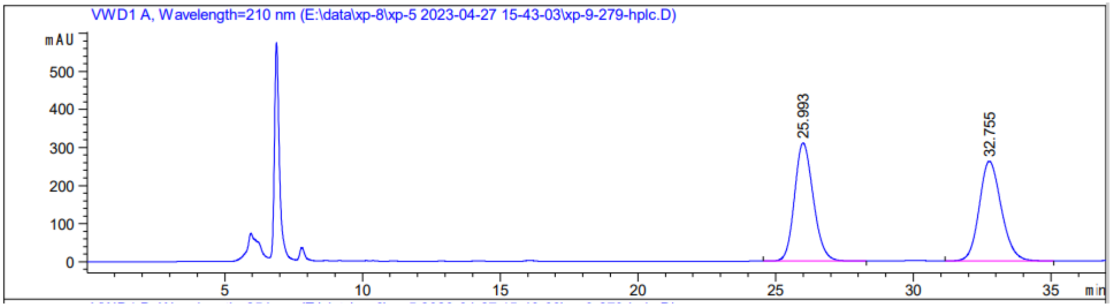

No.	Retention Time min	Area mAU*s	Height mAU	Relative Area %
1	25.993	1.46961e4	310.34140	50.1718
2	32.755	1.45955e4	262.01385	49.8282

Supplementary Figure 17. HPLC chromatographs of compound **(*S*)-18**

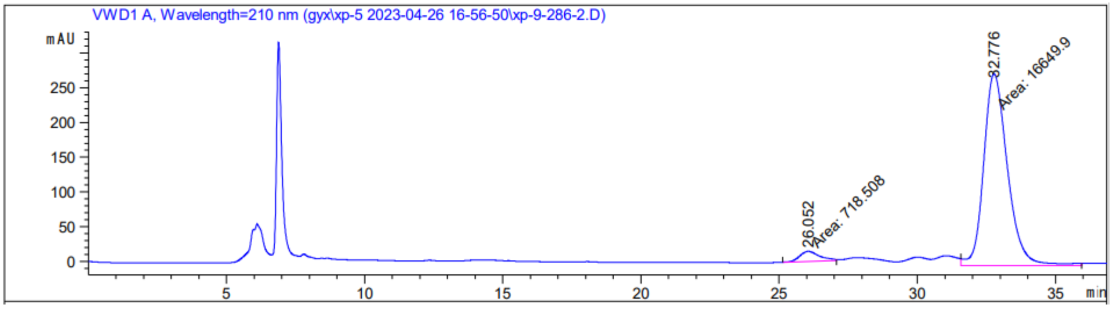

No.	Retention Time min	Area mAU*s	Height mAU	Relative Area %
1	26.052	718.50842	14.43932	4.1369
2	32.776	1.66499e4	276.09882	95.8631

(*S*)-*N*-(2-(5-phenyl-2,3-dihydro-1H-inden-1-yl)ethyl)propionamide ((*S*)-19)

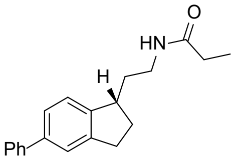

Following the general procedure **F**, **(*S*)-19** was purified as colorless powder (30 mg, 51% yield, 94:6 e.r.) by flash column chromatography (ethyl acetate/petroleum ether = 1/2, V/V), the NMR and HRMS (ESI) was absolutely in consistent with **19**. **(*S*)-19**: $[\alpha]_D^{20}$ – 14.0 (*c* 0.5, $CHCl_3$). The enantiomeric ratio was determined by Daicel Chiralpak AS-H (4.6 mm × 250 mm), Hexanes/IPA = 90/10, 0.8 mL/min, λ = 254 nm, t_R (major) = 35.81 min, t_R (minor) = 32.52 min.

Supplementary Figure 18. HPLC chromatographs of compound **19**

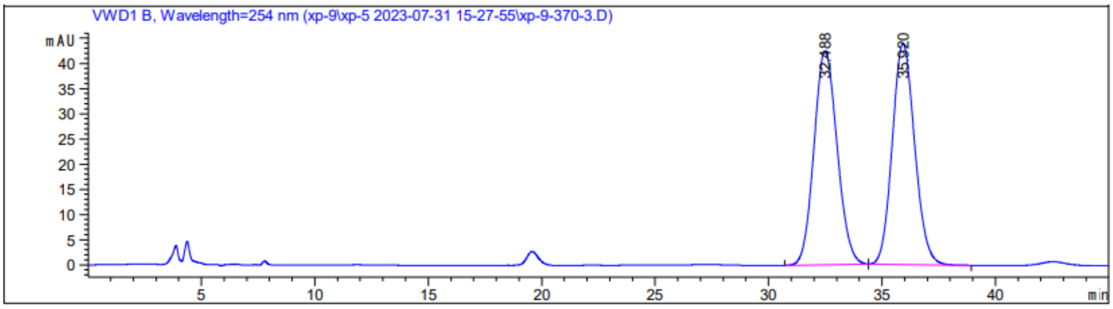

No.	Retention Time<br>min	Area<br>mAU*s	Height<br>mAU	Relative Area<br>%
1	32.488	3009.47217	42.46587	49.9794
2	35.920	3011.95215	43.83153	50.0206

Supplementary Figure 19. HPLC chromatographs of compound **(*S*)-19**

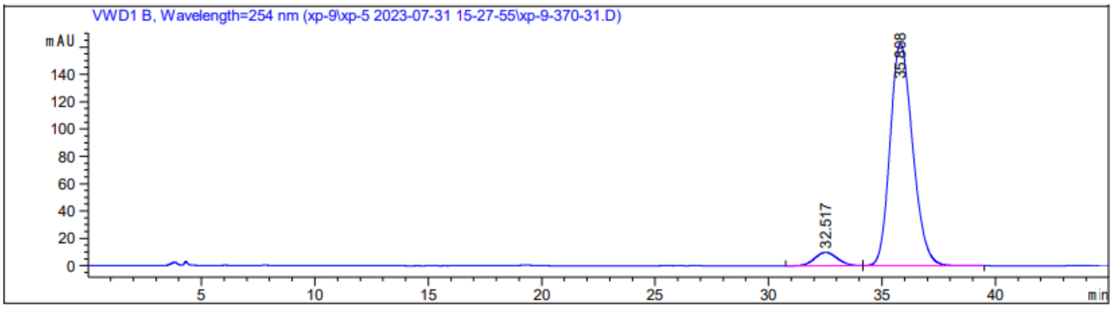

No.	Retention Time<br>min	Area<br>mAU*s	Height<br>mAU	Relative Area<br>%
1	32.517	676.39856	9.80555	5.6788
2	35.808	1.12345e4	162.44754	94.3212

(*S*)-*N*-(2-(2,2-dimethyl-2,3-dihydro-1*H*-inden-1-yl)ethyl)propionamide ((*S*)-22)

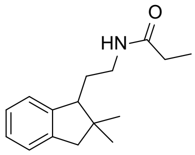

Following the general procedure **F**, **(*S*)-22** was purified as colorless powder (23 mg, 46% yield, 96:4 e.r.) by flash column chromatography (ethyl acetate/petroleum ether = 1/3, V/V) , the NMR and HRMS (ESI) was absolutely in consistent with **22**. **(*S*)-22**: $[\alpha]_D^{20}$ + 27.1 (*c* 1.0, $CHCl_3$). The enantiomeric ratio was determined by Daicel Chiralpak AS-H (4.6 mm × 250 mm), Hexanes/IPA = 90/10, 0.5 mL/min, λ = 210 nm, t_R (major) = 20.21 min, t_R (minor) = 17.84 min.

Supplementary Figure 20. HPLC chromatographs of compound **22**

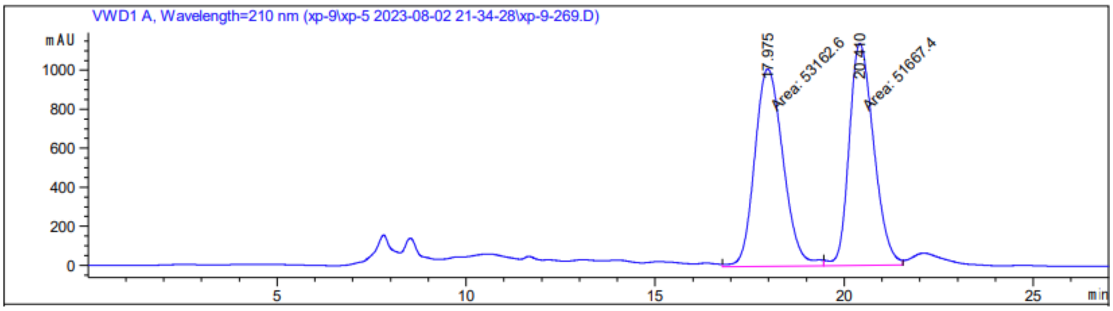

No.	Retention Time min	Area mAU*s	Height mAU	Relative Area %
1	17.975	5.31626e4	1012.82910	50.7132
2	20.410	5.16674e4	1138.24170	49.2868

Supplementary Figure 21. HPLC chromatographs of compound **(*S*)-22**

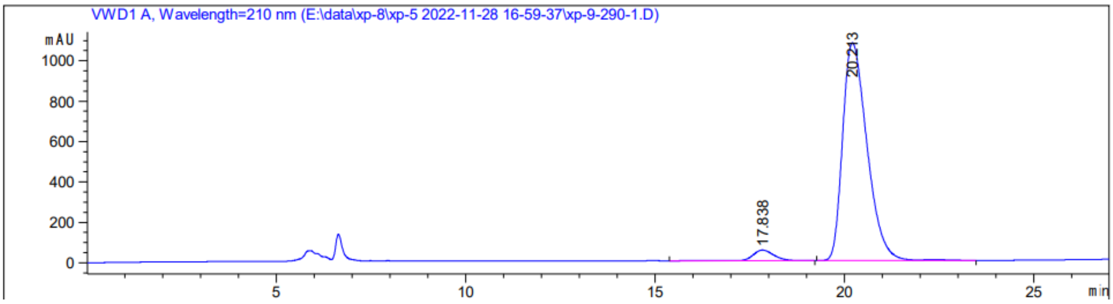

No.	Retention Time min	Area mAU*s	Height mAU	Relative Area %
1	17.838	2064.49365	52.58988	4.0988
2	20.213	4.83040e4	1078.38696	95.9012

(*S*)-*N*-(2-(2,3-dihydro-1*H*-inden-1-yl)ethyl)-2-phenylacetamide ((*S*)-37)

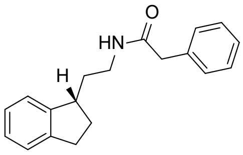

Following the general procedure **F**, **(*S*)-37** was purified as colorless powder (43 mg, 76% yield, 95:5 e.r.) by flash column chromatography (ethyl acetate/petroleum ether = 1/5, V/V) , the NMR and HRMS (ESI) was absolutely in consistent with **37**. **(*S*)-37**: $[\alpha]_D^{20}$ – 3.3 (*c* 1.0, $CHCl_3$). The enantiomeric ratio was determined by Daicel Chiralpak AS-H (4.6 mm × 250 mm), Hexanes/IPA = 70/30, 0.5 mL/min, λ = 210 nm, t_R (major) = 23.28 min, t_R (minor) = 20.89 min.

Supplementary Figure 22. HPLC Chromatographs of compound **37**

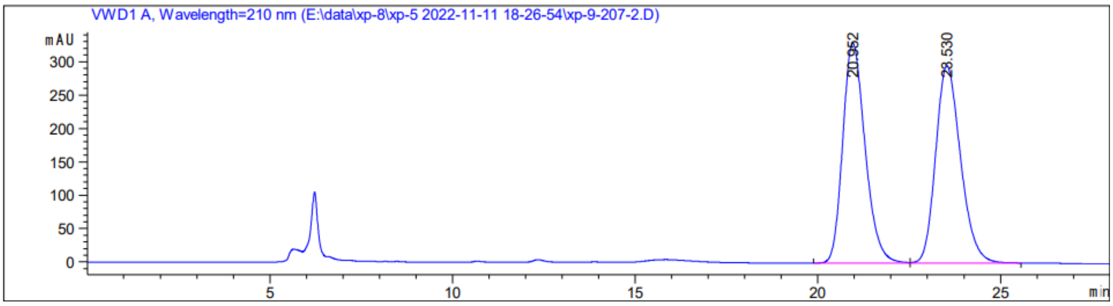

No.	Retention Time min	Area mAU*s	Height mAU	Relative Area %
1	20.952	1.40098e4	329.50058	50.0571
2	23.530	1.39779e4	295.22205	49.9429

Supplementary Figure 23. HPLC chromatographs of compound **(*S*)-37**

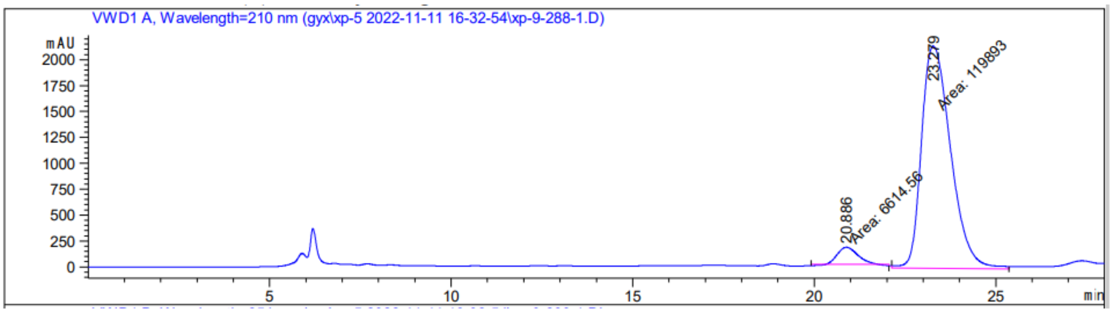

No.	Retention Time min	Area mAU*s	Height mAU	Relative Area %
1	20.886	6614.56055	166.00122	5.2286
2	23.279	1.19893e5	2145.08081	94.7714

(*S*)-*tert*-butyl (2-(2,3-dihydro-1*H*-inden-1-yl)ethyl)carbamate ((*S*)-39)

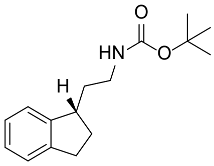

Following the general procedure **F**, **(*S*)-39** was purified as colorless powder (44 mg, 85% yield, 95:5 e.r.) by flash column chromatography (ethyl acetate/petroleum ether = 1/5, V/V) , the NMR and HRMS (ESI) was absolutely in consistent with **39**. **(*S*)-39**: $[\alpha]_D^{20}$ – 3.2 (c 1.0, $CHCl_3$). The enantiomeric ratio was determined by Daicel Chiralpak AS-H (4.6 mm × 250 mm), Hexanes/IPA = 90/10, 0.5 mL/min, λ = 210 nm, t_R (major) = 9.85 min, t_R (minor) = 11.03 min.

Supplementary Figure 24. HPLC chromatographs of compound **39**

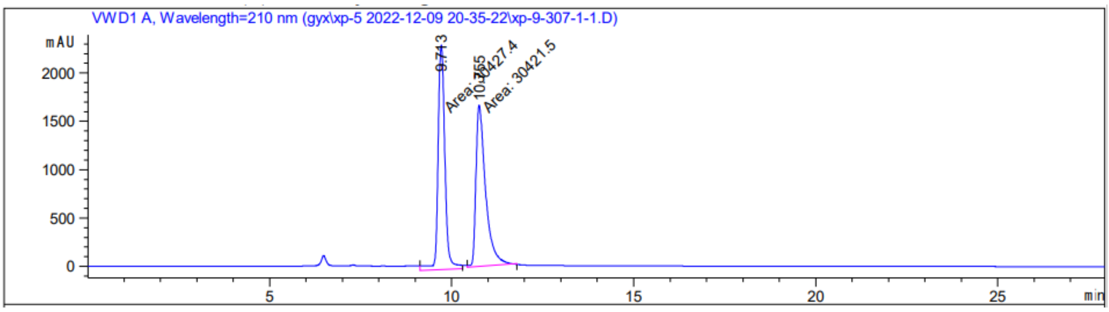

No.	Retention Time min	Area mAU*s	Height mAU	Relative Area %
1	9.713	3.04274e4	2318.73267	50.0048
2	10.755	3.04215e4	1664.67346	49.9952

Supplementary Figure 25. HPLC chromatographs of compound **(*S*)-39**

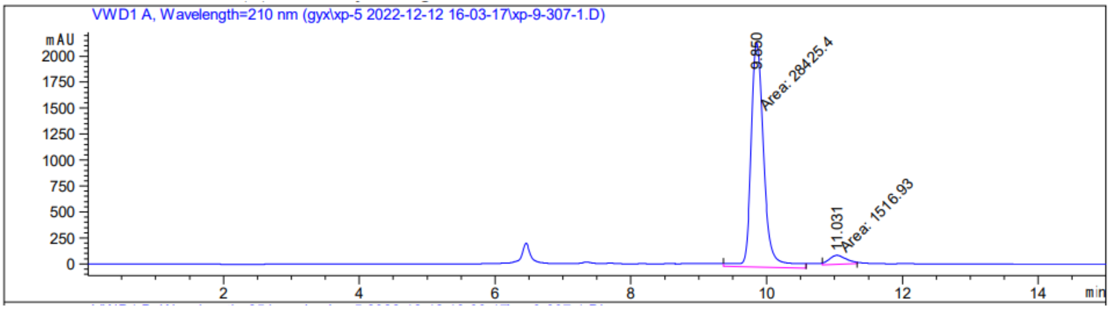

No.	Retention Time min	Area mAU*s	Height mAU	Relative Area %
1	9.850	2.84254e4	2159.67627	94.9338
2	11.031	1516.93091	86.54574	5.0662

(*S*)-*N*-(2-(2,3-dihydro-1*H*-inden-1-yl)ethyl)benzamide ((*S*)-41)

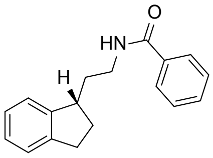

Following the general procedure **F**, **(*S*)-41** was purified as colorless powder (24 mg, 45% yield, 93:7 e.r.) by flash column chromatography (ethyl acetate/petroleum ether = 1/5, V/V), the NMR and HRMS (ESI) was absolutely in consistent with **41**. **(*S*)-41**: $[\alpha]_D^{20}$ – 8.7 (*c* 1.5, $CHCl_3$). The enantiomeric ratio was determined by Daicel Chiralpak ID (4.6 mm × 250 mm), Hexanes/IPA = 90/10, 0.8 mL/min, λ = 210 nm, t_R (major) = 28.25 min, t_R (minor) = 33.95 min.

Supplementary Figure 26. HPLC chromatographs of compound **41**

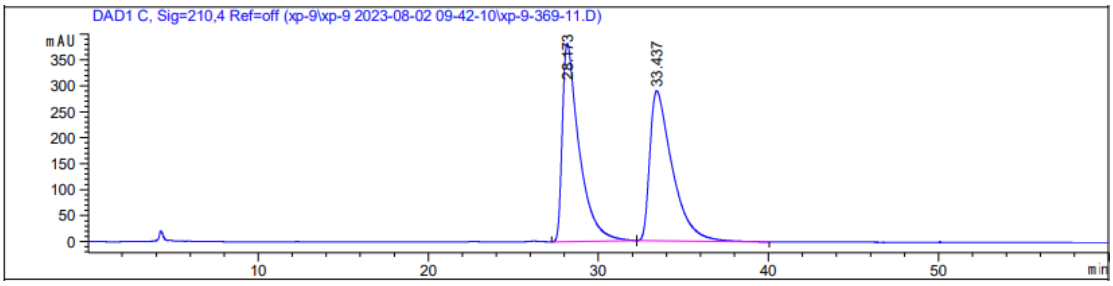

No.	Retention Time min	Area mAU*s	Height mAU	Relative Area %
1	28.173	2.63151e4	381.90042	49.8492
2	33.437	2.64744e4	288.57874	50.1508

Supplementary Figure 27. HPLC chromatographs of compound **(*S*)-41**

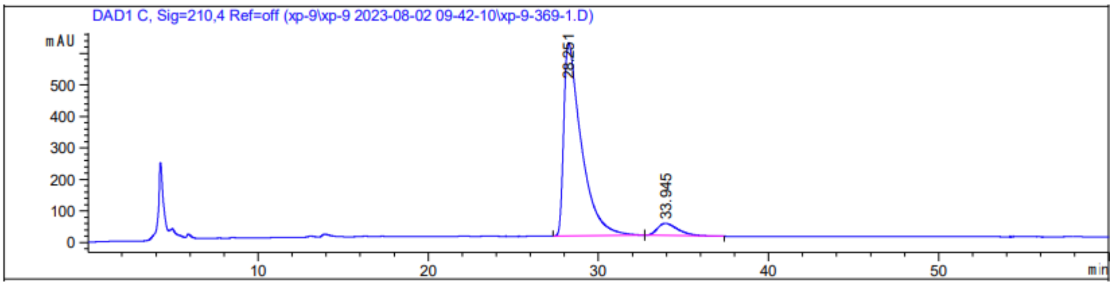

No.	Retention Time min	Area mAU*s	Height mAU	Relative Area %
1	28.251	4.40953e4	613.71259	93.0938
2	33.945	3271.22290	38.72849	6.9062

(*S*)-*N*-(1-(2,3-dihydro-1*H*-inden-1-yl)propan-2-yl)benzamide ((*S*)-45)

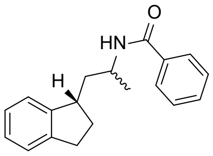

Following the general procedure **F**, **(*S*)-45** was purified as colorless powder (42 mg, 75% yield, 1:1 d.r., 94:6 e.r.; 90:10 e.r.) by flash column chromatography (ethyl acetate/petroleum ether = 1/5, V/V), the NMR and HRMS (ESI) was absolutely in consistent with **45**. **(*S*)-45**: $[\alpha]_D^{20}$ – 9.9 (*c* 1.0, $CHCl_3$). The enantiomeric ratio was determined by Daicel Chiralpak AS-H (4.6 mm × 250 mm), Hexanes/IPA = 98/2, 0.5 mL/min, λ = 210 nm. **45** (Major): t_R (major) = 94.96 min, t_R (minor) = 64.90 min; **45** (Minor): t_R (major) = 79.54 min, t_R (minor) = 85.22 min.

Supplementary Figure 28. HPLC chromatographs of compound **45**

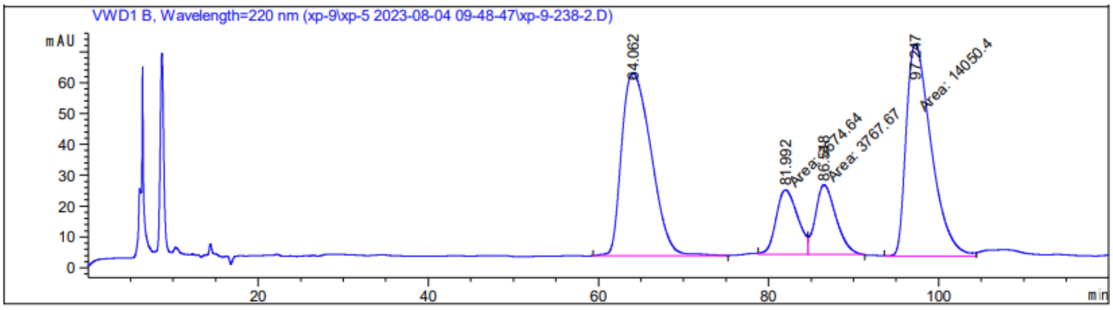

No.	Retention Time<br>min	Area<br>mAU*s	Height<br>mAU	Relative Area<br>%
1	64.062	1.42463e4	59.24515	39.8620
2	81.992	3674.63501	20.80709	10.2819
3	86.518	3767.66968	22.44749	10.5422
4	97.247	1.40504e4	68.59006	39.3139

Supplementary Figure 29. HPLC chromatographs of compound **(*S*)-45**

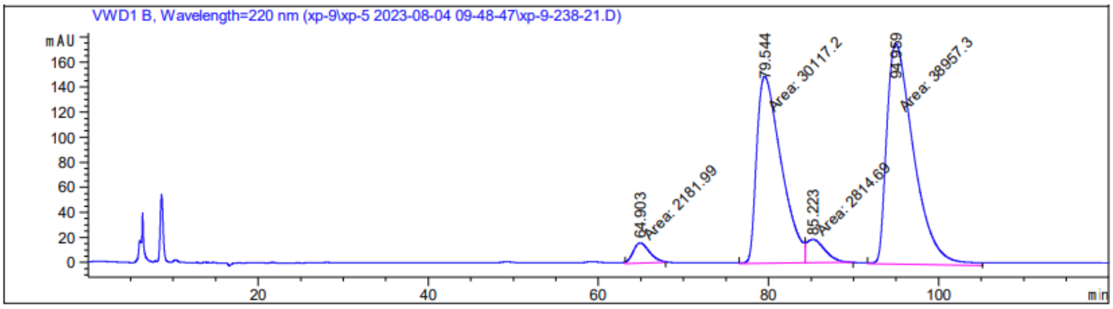

No.	Retention Time<br>min	Area<br>mAU*s	Height<br>mAU	Relative Area<br>%
1	64.903	2181.99170	16.22157	2.9458
2	79.544	3.01172e4	149.32738	40.6599
3	85.223	2814.68848	18.64703	3.8000
4	94.959	3.89573e4	176.10608	52.5944

(*S*)-*N*-(1-(2,3-dihydro-1*H*-inden-1-yl)-4-methylpentan-2-yl)benzamide ((*S*)-46)

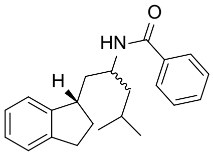

Following the general procedure **F**, **(*S*)-46** was purified as colorless powder (53 mg, 83% yield, 1:1 d.r., 91:9 e.r.; 96:4 e.r.) by flash column chromatography (ethyl acetate/petroleum ether = 1/10, V/V), the NMR and HRMS (ESI) was absolutely in consistent with **46**. **(*S*)-46**: $[\alpha]_D^{20}$ – 11.2 (*c* 1.0, $CHCl_3$). The enantiomeric ratio was determined by Daicel Chiralpak AD-H (4.6 mm × 250 mm), Hexanes/IPA = 95/5, 0.5 mL/min, λ = 210 nm. **46** (Major): t_R (major) = 22.04 min, t_R (minor) = 19.51 min; **46** (Minor): t_R (major) = 24.60 min, t_R (minor) = 28.74 min.

Supplementary Figure 30. HPLC Chromatographs of compound **46**

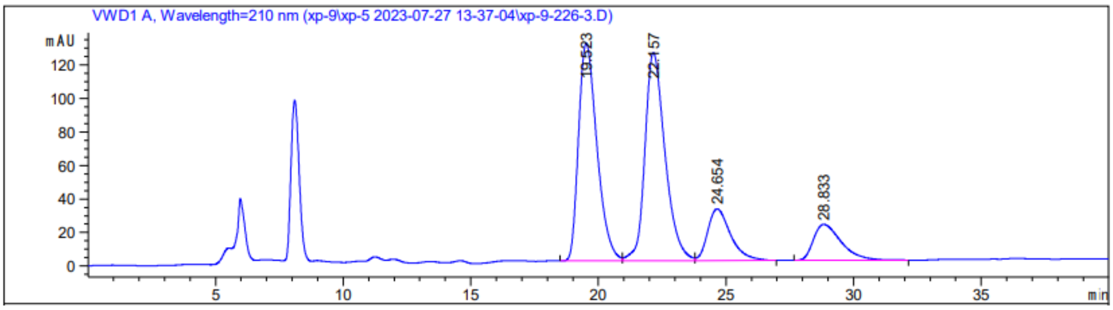

No.	Retention Time<br>min	Area<br>mAU*s	Height<br>mAU	Relative Area<br>%
1	19.523	6512.01514	129.70677	38.5844
2	22.157	6813.85986	124.28479	40.3729
3	24.654	1925.21936	30.83324	11.4071
4	28.833	1626.23242	21.48740	9.6356

Supplementary Figure 31. HPLC chromatographs of compound **(*S*)-46**

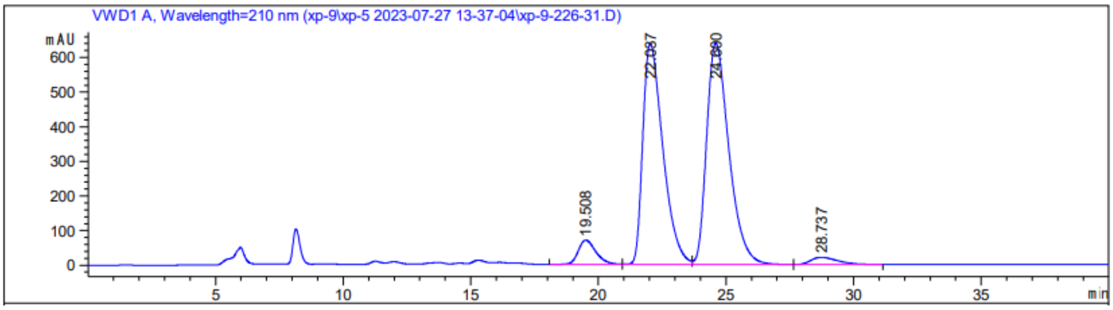

No.	Retention Time<br>min	Area<br>mAU*s	Height<br>mAU	Relative Area<br>%
1	19.508	3471.29761	70.81324	4.3993
2	22.037	3.49716e4	637.70325	44.3209
3	24.600	3.89145e4	639.78442	49.3178
4	28.737	1548.13635	20.98323	1.9620

(*S*)-*N*,*N'*-(6-(2,3-dihydro-1*H*-inden-1-yl)hexane-1,5-diyl)dibenzamide ((*S*)-47)

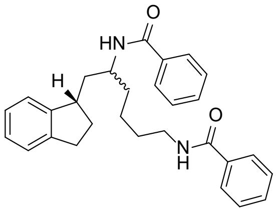

Following the general procedure **F**, **(*S*)-47** was purified as colorless powder (40 mg, 45% yield, 2.2:1 d.r., 97:3 e.r.; 77:23 e.r.) by flash column chromatography (ethyl acetate/petroleum ether = 1/3, V/V), the NMR and HRMS (ESI) was absolutely in consistent with **47**. **(*S*)-47**: $[\alpha]_D^{20}$ – 15.8 (*c* 1.0, $CHCl_3$). The enantiomeric ratio was determined by Daicel Chiralpak ID (4.6 mm × 250 mm), Hexanes/IPA = 85/15, 0.8 mL/min, λ = 220 nm. **47** (Major): t_R (major) = 41.18 min, t_R (minor) = 63.32 min; **47** (Minor): t_R (major) = 86.03 min, t_R (minor) = 36.62 min.

Supplementary Figure 32. HPLC chromatographs of compound **47**

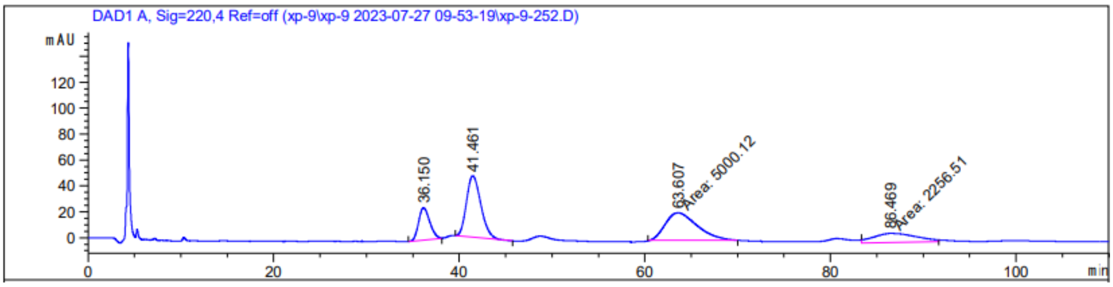

No.	Retention Time min	Area mAU*s	Height mAU	Relative Area %
1	36.150	2073.74731	24.80023	14.3789
2	41.461	5091.76074	47.25567	35.3052
3	63.607	5000.11914	21.09720	34.6697
4	86.469	2256.51465	7.01677	15.6462

Supplementary Figure 33. HPLC chromatographs of compound **(*S*)-47**

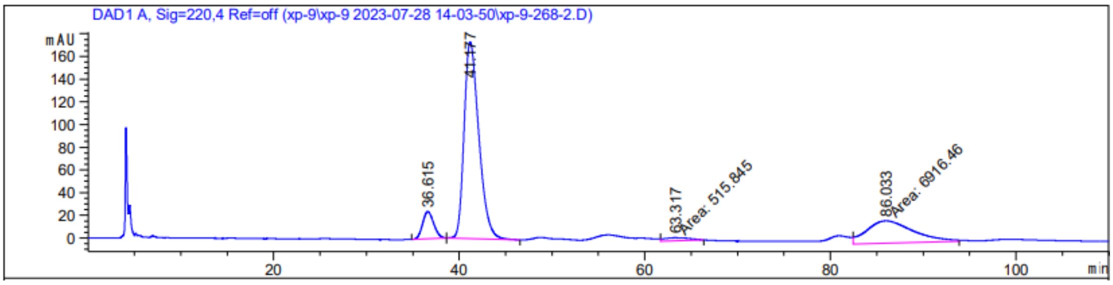

No.	Retention Time min	Area mAU*s	Height mAU	Relative Area %
1	36.615	1989.59009	23.95823	6.9860
2	41.177	1.90578e4	173.24030	66.9171
3	63.317	515.84540	2.75136	1.8113
4	86.033	6916.46240	19.75338	24.2856

Supplementary Note 9. Synthesis of Melatonin receptor agonist (55)

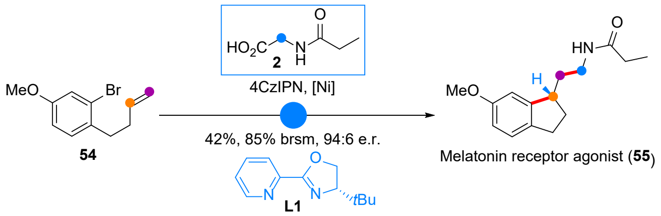

2-bromo-1-(but-3-en-1-yl)-4-methoxybenzene (54)

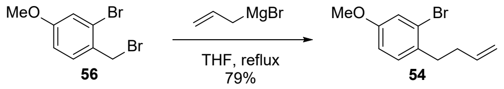

In a flame-dried 100 mL round-bottom flask, equipped with a magnetic stir bar and rubber septum under nitrogen, was placed 2-bromo-1-(bromomethyl)-4-methoxybenzene (**56**) (1.11 g, 4.0 mmol, 1.0 equiv.) and anhydrous THF (15 mL). The flask was placed in an ice water bath and allylmagnesium bromide (6.0 mL, 1.0 M in diethyl ether, 6.0 mmol, 1.5 equiv) was added dropwise via syringe. The reaction vessel was then fitted with a reflux condenser and heated to reflux temperature in an oil bath overnight. The reaction was then cooled to 0 °C and quenched by the addition of saturated NH_4Cl solution. The resulting solution was extracted with EtOAc (3 x 20 mL). The combined organic layers were dried over Na_2SO_4, filtered, and evaporated. The residue was purified by silica gel column chromatography (petroleum ether) to afford **54** colorless liquid (0.76 g, 79% yield) by flash column chromatography. Analytical data: ^{1}H NMR (400 MHz, $CDCl_3$) δ 7.11 (d, J = 8.4 Hz, 2H), 6.80 (dd, J = 8.4, 2.5 Hz, 1H), 5.92 – 5.82 (m, 1H), 5.05 (d, J = 16.8 Hz, 1H), 4.99 (d, J = 10.0 Hz, 1H), 3.78 (s, 3H), 2.77 (t, J = 7.6 Hz, 2H), 2.34 (dt, J = 7.9, 7.0 Hz, 2H). ^{13}C NMR (100 MHz, $CDCl_3$) δ 158.4, 137.9, 133.1, 130.8, 124.6, 118.0, 115.2, 113.6, 55.6, 34.8, 34.3. HRMS (ESI) calcd for $C_{11}H_{14}BrO$ $[M + H]^+$: 241.0223, Found: 241.0218.

(*S*)-*N*-(2-(6-methoxy-2,3-dihydro-1*H*-inden-1-yl)ethyl)propionamide (55)

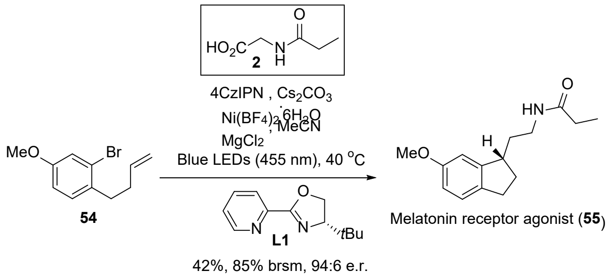

To an 10 mL glass tube equipped with a septum and a magnetic stir bar was added $Ni(BF_4)_2{\cdot}6H_2O$ (13.6 mg, 0.04 mmol, 20 mol%), **L1** (9.0 mg, 0.044 mmol, 22 mol%) and MeCN (2.0 mL) in the glove box. The mixture was stirred at room temperature for 30 min. Amino acid **2** (39.0 mg, 0.3 mmol, 1.5 equiv.), $MgCl_2$ (4.8 mg, 0.05 mmol, 0.25 equiv.), Cs_2CO_3 (108 mg, 0.33 mmol, 1.65 equiv.), 4CziPN (3.2 mg, 0.004 mmol, 2 mol%) and the unactivated alkene **54** (48 mg, 0.2 mmol, 1.0 equiv.) were then added in sequence under the argon. The resulting mixture was then sealed and wrapped with electrical tape and removed from the glove box. The reaction mixture was irradiated with two 18 W 455 nm LEDs for 48 h. The reaction was maintained at 40 °C by heating in an oil bath and cooling by a fan. Then, the solvent was evaporated and concentrated, the residue was purified by silica chromatography (ethyl acetate/petroleum ether = 1/2, V/V), **(*S*)-55** (21 mg, 42% yield, 94:6 e.r.) was afforded as colorless powder. **(*S*)-55**: $[\alpha]_D^{20}$ – 7.9 (*c* 1.0, EtOH). Analytical data: ^1H NMR (400 MHz, $CDCl_3$) δ 7.11 (d, *J* = 8.0 Hz, 1H), 6.75 (d, *J* = 2.2 Hz, 1H), 6.71 (dd, *J* = 8.1, 2.4 Hz, 1H), 5.49 (br s, 1H), 3.78 (s, 3H), 3.42 – 3.36 (m, 2H), 3.15 – 3.07 (m, 1H), 2.89 – 2.73 (m, 2H), 2.36 – 2.28 (m, 1H), 2.19 (q, *J* = 7.6 Hz, 2H), 2.10 – 2.01 (m, 1H), 1.76 – 1.67 (m, 1H), 1.65 – 1.55 (m, 1H), 1.15 (t, *J* = 7.6 Hz, 3H). ^{13}C NMR (100 MHz, $CDCl_3$) δ 173.9, 158.8, 148.3, 135.9, 125.1, 112.4, 109.3, 55.6, 42.8, 38.1, 35.0, 32.6, 30.7, 29.9, 10.0. HRMS (ESI) calcd for $C_{15}H_{22}NO_2$ $[M + H]^+$: 248.1645, Found: 248.1645. The enantiomeric ratio was determined by Daicel Chiralpak AS-H (4.6 mm × 250 mm), Hexanes/IPA = 90/10, 1.0 mL/min, λ = 290 nm, t_R (major) = 26.59 min, t_R (minor) = 23.43 min.

Supplementary Figure 34. HPLC chromatographs of compound **55**

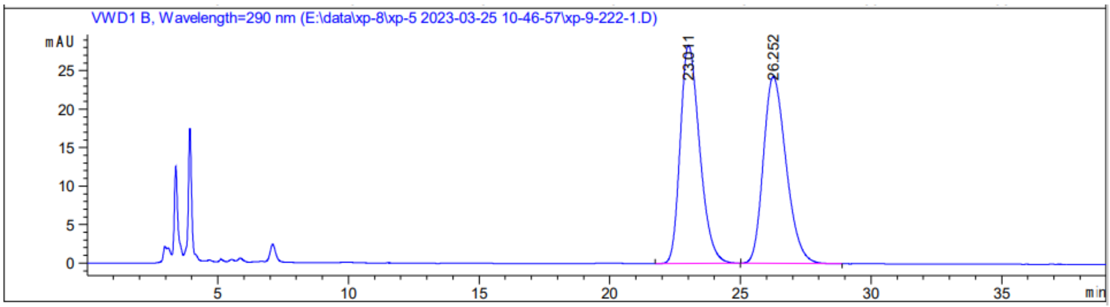

No.	Retention Time<br>min	Area<br>mAU*s	Height<br>mAU	Relative Area<br>%
1	23.010	1466.12500	28.34593	49.9225
2	26.252	1470.67798	24.26499	50.0775

Supplementary Figure 35. HPLC chromatographs of compound **(*S*)-55**

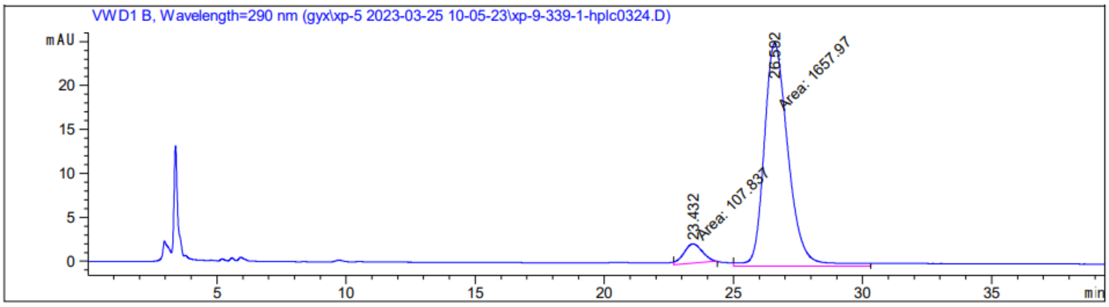

No.	Retention Time<br>min	Area<br>mAU*s	Height<br>mAU	Relative Area<br>%
1	23.432	107.83689	2.16738	6.1070
2	26.592	1657.96509	25.38324	93.8930

Supplementary Note 10. Synthesis of S20242 (57)

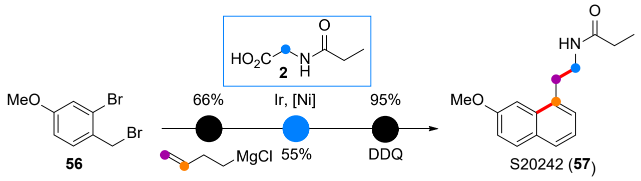

2-bromo-4-methoxy-1-(pent-4-en-1-yl)benzene (S27)

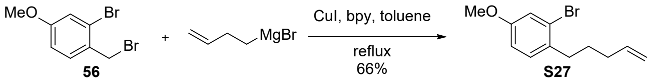

In a flame-dried 100 mL round-bottom flask, was placed benzyl bromide **56** (1.39 g, 5.0 mmol, 1.0 equiv), CuI (95.0 mg, 0.50 mmol, 0.1 equiv) and 2,2'-bipyridyl (78.0 mg, 0.50 mmol, 0.1 equiv) in anhydrous toluene (5.0 mL) at 0 °C. The Grignard solution was then slowly added to this mixture, then allowed to warm to room temperature and stirred for an additional 2 h when TLC indicated the reaction was complete. The reaction was quenched by the addition of saturated NH_4Cl solution. The resulting solution was extracted with EtOAc (3 x 20 mL). The combined organic layers were dried over Na_2SO_4, filtered, and evaporated. The residue was purified by silica gel column chromatography (petroleum ether) to give the product **S27** as colorless liquid (0.84 g, 66% yield). Analytical data: ^{1}H NMR (400 MHz, $CDCl_3$) δ 7.12 – 7.10 (m, 2H), 6.79 (d, J = 8.4 Hz, 1H), 5.91 – 5.81 (m, 1H), 5.05 (d, J = 16.8 Hz, 1H), 5.00 (d, J = 10.4 Hz, 1H), 3.78 (s, 3H), 2.68 (t, J = 7.6 Hz, 2H), 2.12 (dt, J = 7.2, 6.8 Hz, 2H), 1.72 – 1.65 (m, 2H). ^{13}C NMR (100 MHz, $CDCl_3$) δ 158.4, 138.6, 133.8, 130.7, 124.5, 118.0, 114.9, 113.7, 55.6, 34.8, 33.5, 29.5. HRMS (ESI) calcd for $C_{12}H_{16}BrO$ $[M + H]^+$: 255.0379, Found: 255.0376.

***N*-(2-(7-methoxy-1,2,3,4-tetrahydronaphthalen-1-yl)ethyl)propionamide (27)**

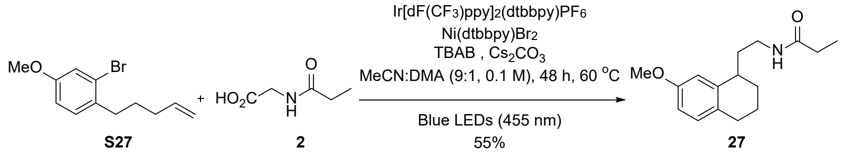

To an 10 mL glass tube equipped with a septum and a magnetic stir bar was added Ni(dtbbpy)Br_2 (19.5 mg, 0.04 mmol, 20 mol%), Amino acid **2** (52.0 mg, 0.40 mmol, 2.0 equiv.), TBAB (16.1 mg, 0.05 mmol, 25 mol%), Cs_2CO_3 (108.0 mg, 0.33 mmol, 1.65 equiv.), Ir[dF(CF_3)ppy]$_2$(dtbbpy)PF_6 (4.5 mg, 0.004 mmol, 2.0 mol%) and MeCN (1.8 mL) and DMA (0.2 mL) in the glove box. The unactivated alkene **S27** (54.0 mg, 0.20 mmol, 1.0 equiv.) was added to the glass tube with a pipette gun under the argon. The resulting mixture was then sealed and wrapped with electrical tape and then irradiated with two parallel 18 W LEDs (455 nm,) from a distance of approximate 8 cm for 48 h. The reaction was maintained at 60 ^{o}C by heating in an oil bath and cooling by a fan. Then, the solvent was evaporated and concentrated, the residue was purified by silica chromatography (ethyl acetate/petroleum ether = 1/1.5, V/V) to afford **27** as colorless powder (29 mg, 55% yield). Analytical data: ^{1}H NMR (400 MHz, $CDCl_3$) δ 6.97 (d, *J* = 8.2 Hz, 1H), 6.67 (d, *J* = 8.4 Hz, 2H), 5.40 (br s, 1H), 3.77 (s, 3H), 3.40 – 3.33 (m, 2H), 2.80 (br s, 1H), 2.68 (br s, 2H), 2.17 (q, *J* = 7.6 Hz, 2H), 1.90 – 1.66 (m, 6H), 1.13 (t, *J* = 7.6 Hz, 3H). ^{13}C NMR (100 MHz, $CDCl_3$) δ 173.8, 157.7, 141.5, 130.2, 129.4, 113.5, 111.9, 55.4, 37.8, 36.7, 35.8, 30.0, 28.9, 27.7, 20.2, 10.0. HRMS (ESI) calcd for $C_{16}H_{24}NO_2$ $[M + H]^+$: 262.1802, Found: 262.1800.

***N*-(2-(7-methoxynaphthalen-1-yl)ethyl)propionamide (57)**

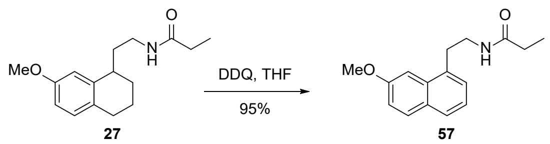

In a flame-dried 10 mL round-bottom flask, was placed *N*-(2-(1,2,3,4-tetrahydronaphthalen-1-yl)ethyl)propionamide **27** (24.0 mg, 0.09 mmol, 1.0 equiv.) and THF (3.0 mL). DDQ (61.0 mg, 0.27 mmol, 3.0 equiv.) was added to the solution. After the reaction mixture was stirred at reflux for 3 hours, 10% NaOH was added to quenched reaction. The organic layer was extracted with dichloromethane (3 × 5 mL), dried over Na_2SO_4, filtered, and concentrated. The residue was purified by silica gel column chromatography (ethyl acetate/petroleum ether = 1/1.5, V/V) to afford **57** as colorless powder (22 mg, 95% yield). Analytical data: ^{1}H NMR (400 MHz, $CDCl_3$) δ 7.57 (d, *J* = 8.9 Hz, 1H), 7.50 – 7.49 (m, 1H), 7.26 (s, 1H), 7.09 – 7.07 (m, 2H), 6.97 (d, *J* = 8.9 Hz, 1H), 5.35 (br s, 1H), 3.79 (s, 3H), 3.47 – 3.42 (m, 2H), 3.06 (t, *J* = 7.0 Hz, 2H), 1.98 (q, *J* = 7.6 Hz, 2H), 0.93 (t, *J* = 7.6 Hz, 3H). 13C NMR (100 MHz, CDCl3) δ 174.0, 158.1, 133.8, 133.3, 130.4, 129.5, 127.3, 127.2, 123.3, 118.5, 102.6, 55.7, 40.0, 33.3, 29.9, 10.0. HRMS (ESI) calcd for $C_{16}H_{20}NO_2$ $[M + H]^+$: 258.1489, Found: 258.1487.

Supplementary Note 11. Gram-scale synthesis of compound 3

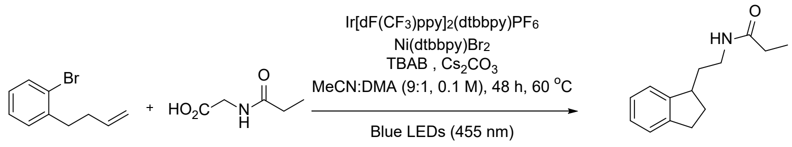

To an oven dried 200.0 mL sealed tube with a magnetic stirring bar was added Ni(dtbbpy)Br_2 (0.78 g, 1.6 mmol, 20 mol%), amino acid **2** (2.10 g, 16.0 mmol, 2.0 equiv.), TBAB (0.64 g, 2.0 mmol, 25 mol%), Cs_2CO_3 (4.30 g, 13.2 mmol, 1.65 equiv.), Ir[dF(CF_3)ppy]$_2$(dtbbpy)PF_6 (0.18 g, 0.16 mmol, 2.0 mol%) and MeCN (72.0 mL) and DMA (8.0 mL) in the glove box. The unactivated alkene **1** (1.68 g, 8.0 mmol, 1.0 equiv.) was added to the glass tube with a pipette gun under the argon. The resulting mixture was then sealed and wrapped with electrical tape and then irradiated with two parallel 18 W LEDs (455 nm,) from a distance of approximate 8 cm for 48 h. The reaction was maintained at 60 ^{o}C by heating in an oil bath and cooling by a fan. Then, the solvent was evaporated and concentrated, the residue was purified by silica chromatography (ethyl acetate/petroleum ether = 1/2, V/V) to afford **3** as colorless powder (1.35 g, 78% yield).

Supplementary Note 12. Unsuccessful examples

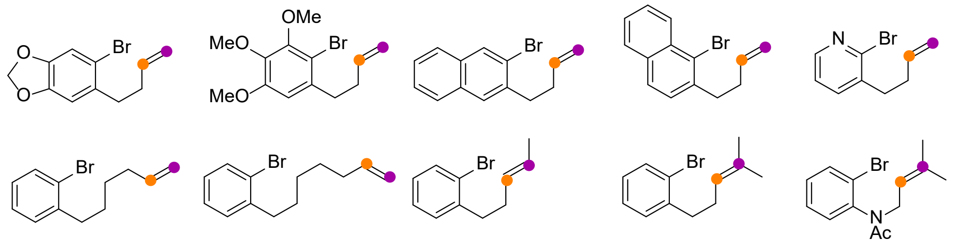

NMR spectra

Supplementary Figure 36. ^{1}H NMR spectra of compound **1** (400 MHz, $CDCl_3$)

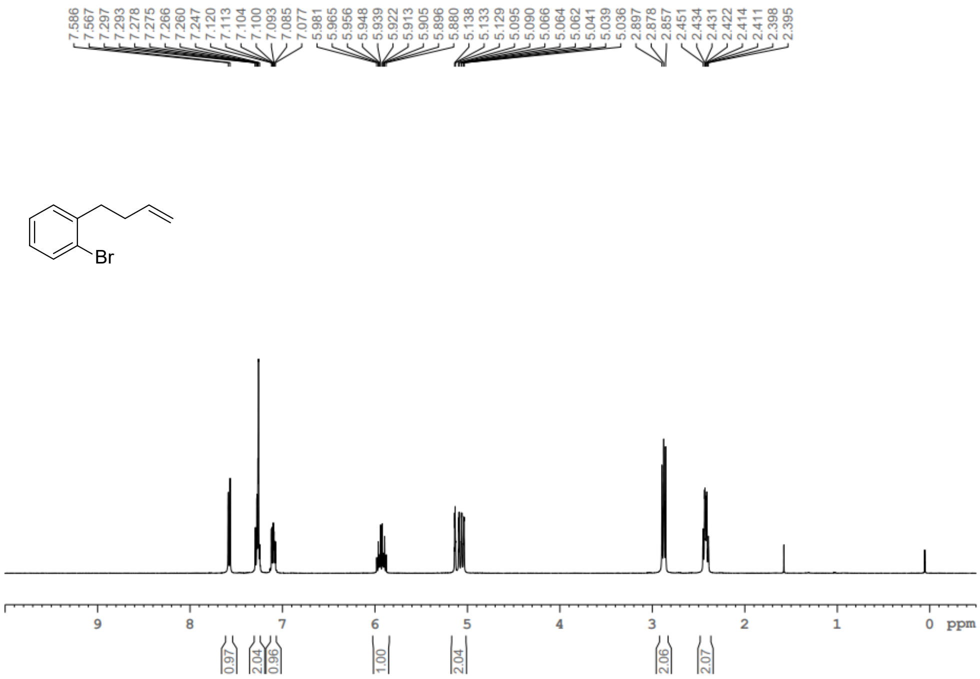

Supplementary Figure 37. ^{13}C NMR spectra of compound **1** (100 MHz, $CDCl_3$)

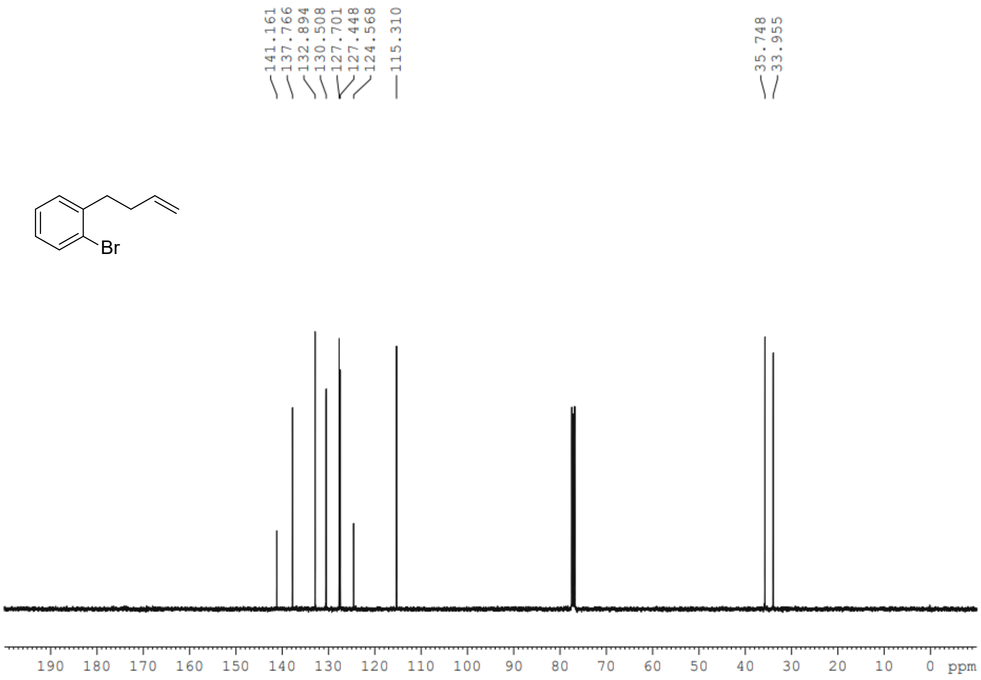

Supplementary Figure 38. ^{1}H NMR spectra of compound **S4** (500 MHz, $CDCl_3$)

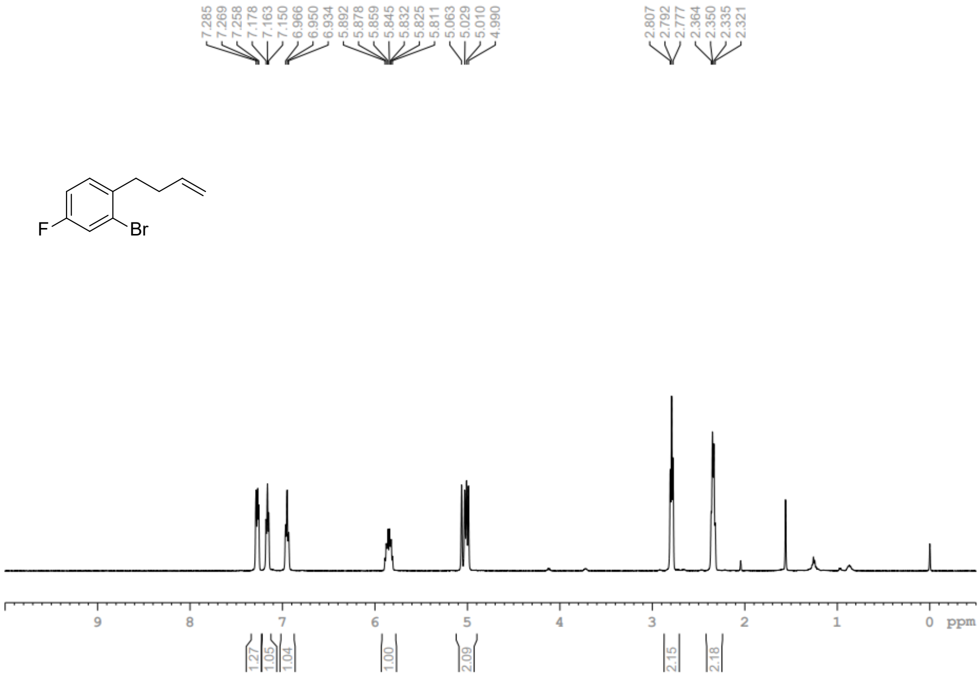

Supplementary Figure 39. ^{13}C NMR spectra of compound **S4** (125 MHz, $CDCl_3$)

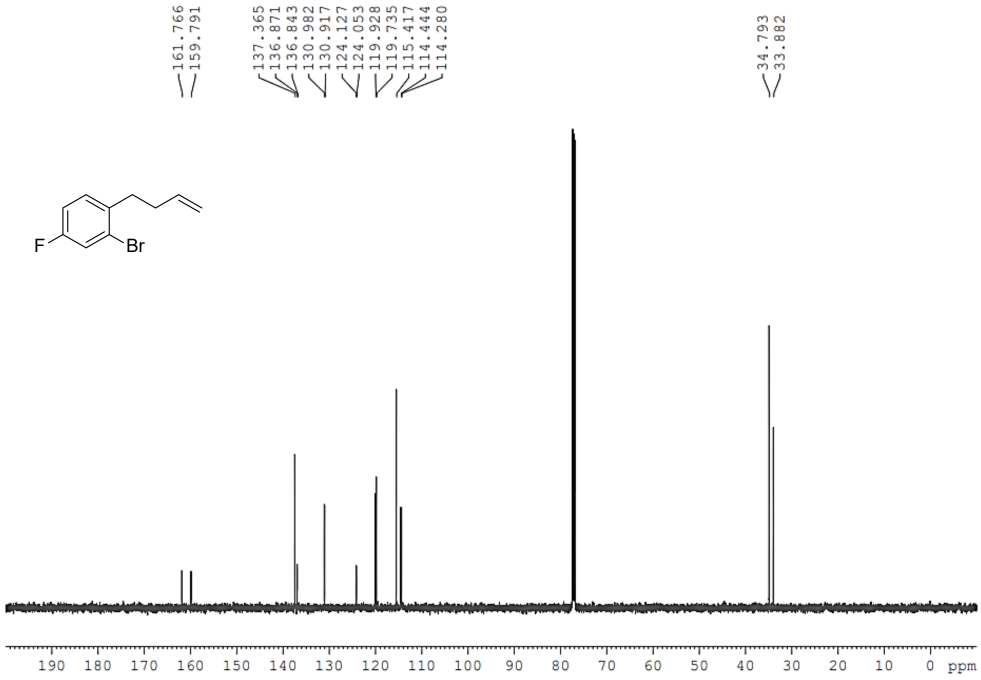

Supplementary Figure 40. ^{19}F NMR spectra of compound **S4** (471 MHz, $CDCl_3$)

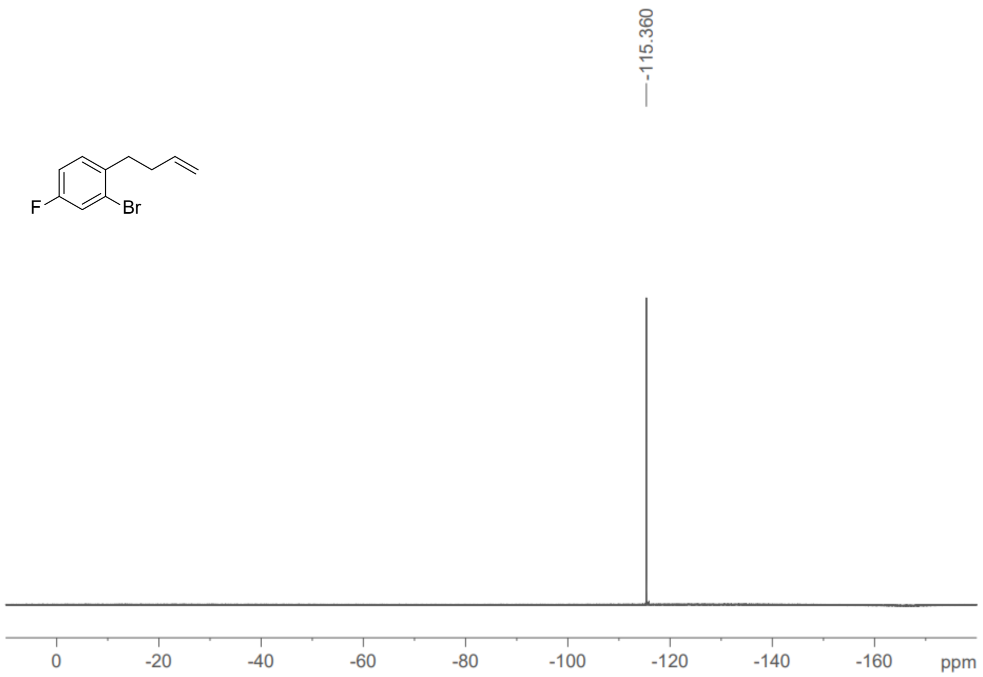

Supplementary Figure 41. ^{1}H NMR spectra of compound **S5** (500 MHz, $CDCl_3$)

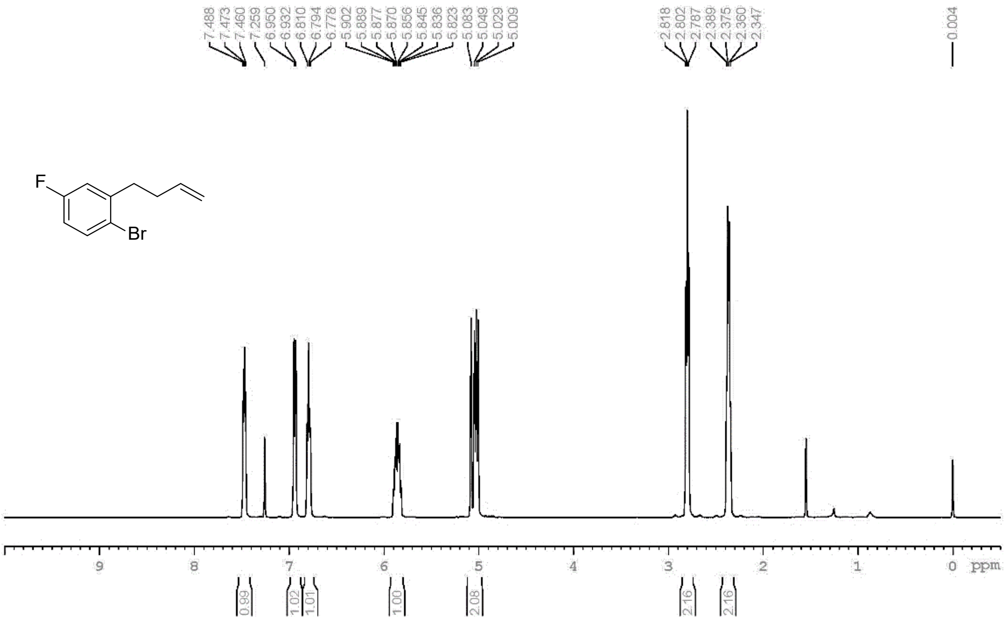

Supplementary Figure 42. ^{13}C NMR spectra of compound **S5** (125 MHz, $CDCl_3$)

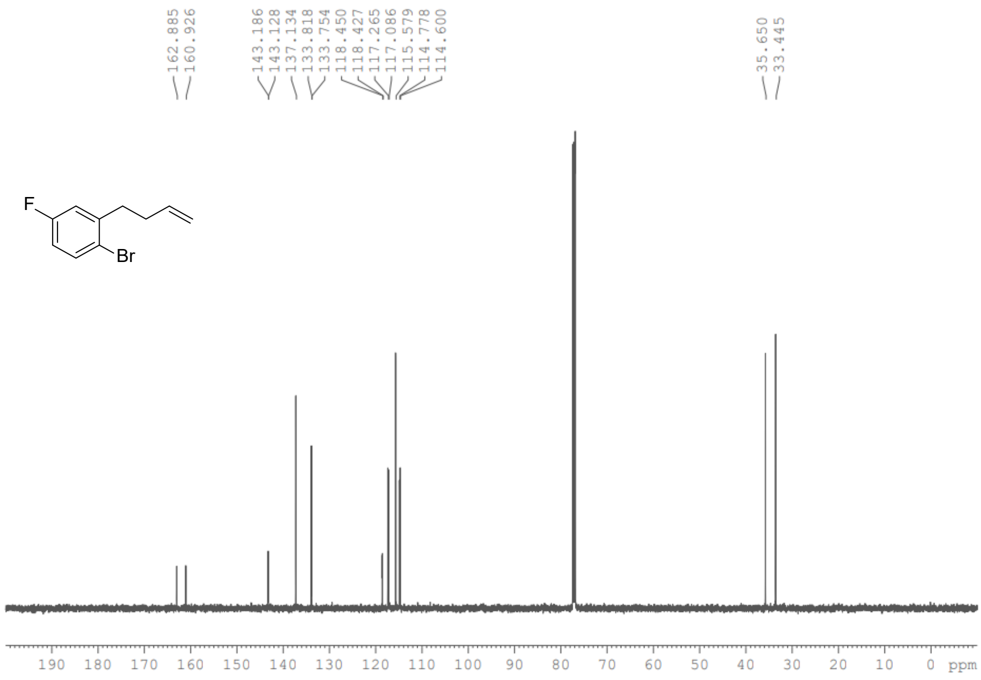

Supplementary Figure 43. ^{19}F NMR spectra of compound **S5** (471 MHz, $CDCl_3$)

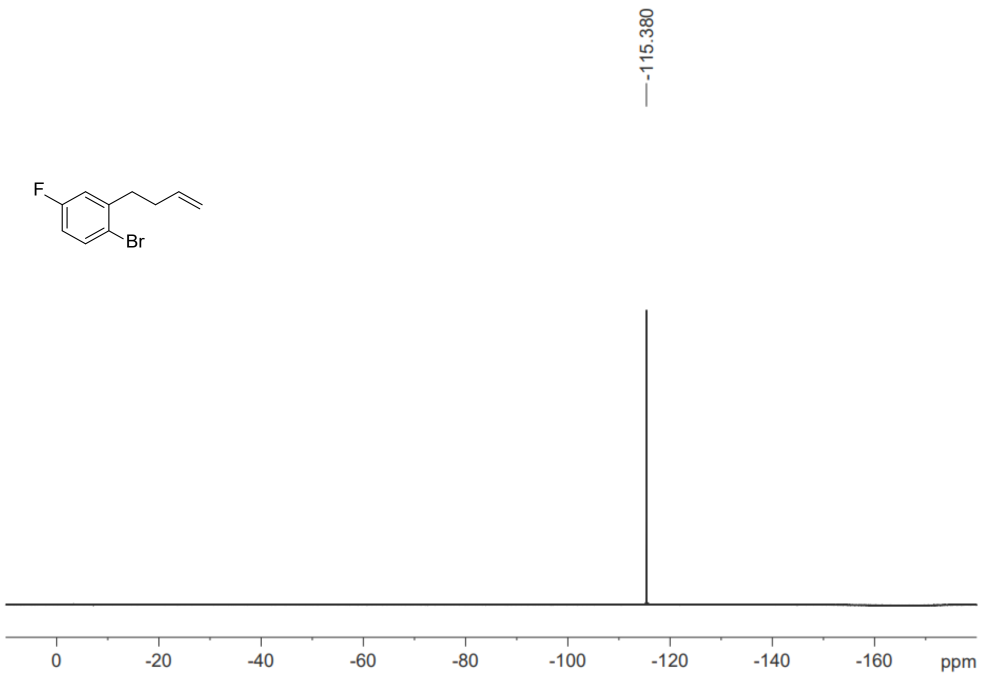

Supplementary Figure 44. ^{1}H NMR spectra of compound **S6** (400 MHz, $CDCl_3$)

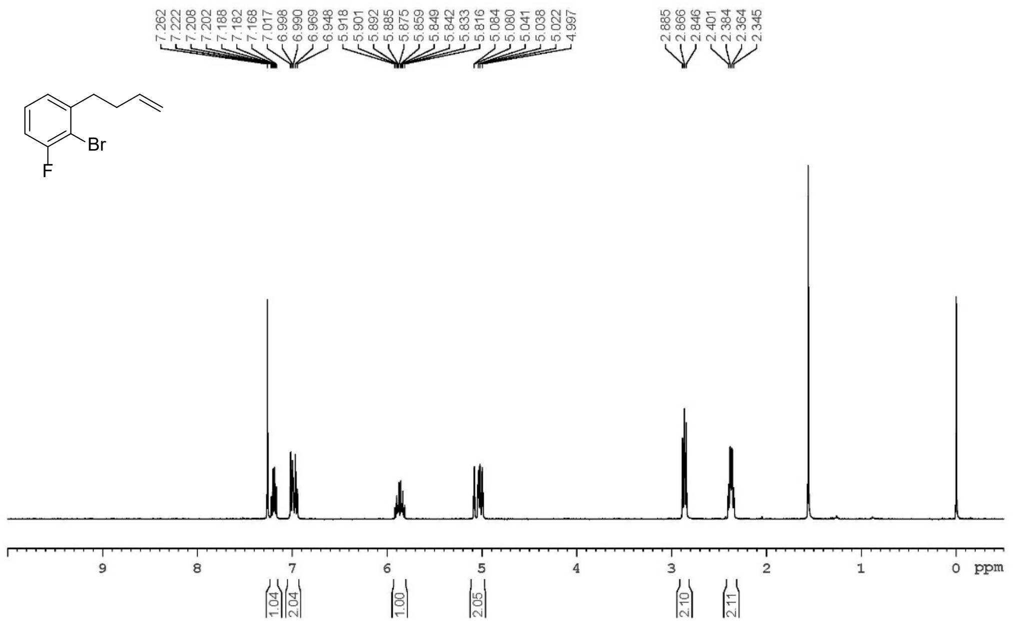

Supplementary Figure 45. ^{13}C NMR spectra of compound **S6** (100 MHz, $CDCl_3$)

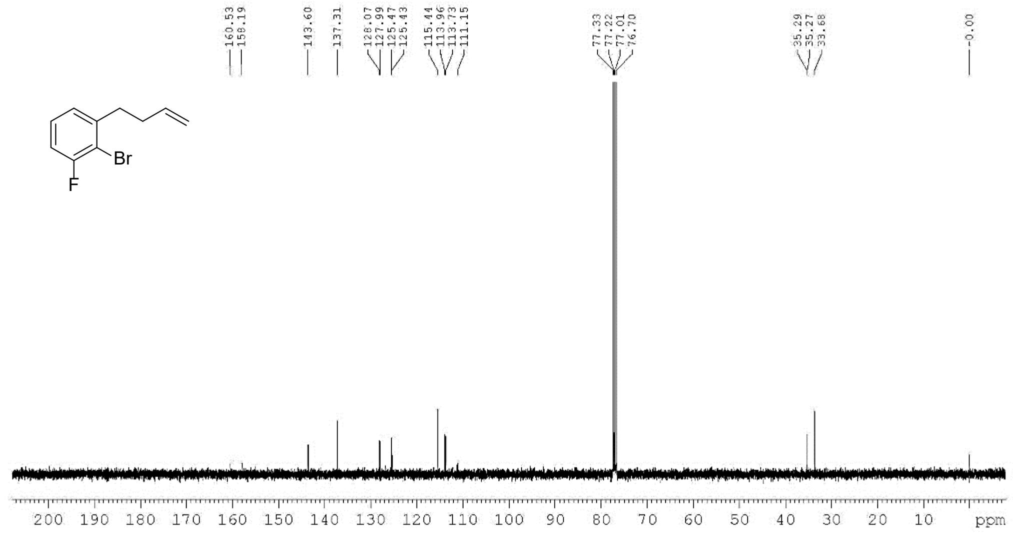

Supplementary Figure 46. ^{19}F NMR spectra of compound **S6** (376 MHz, $CDCl_3$)

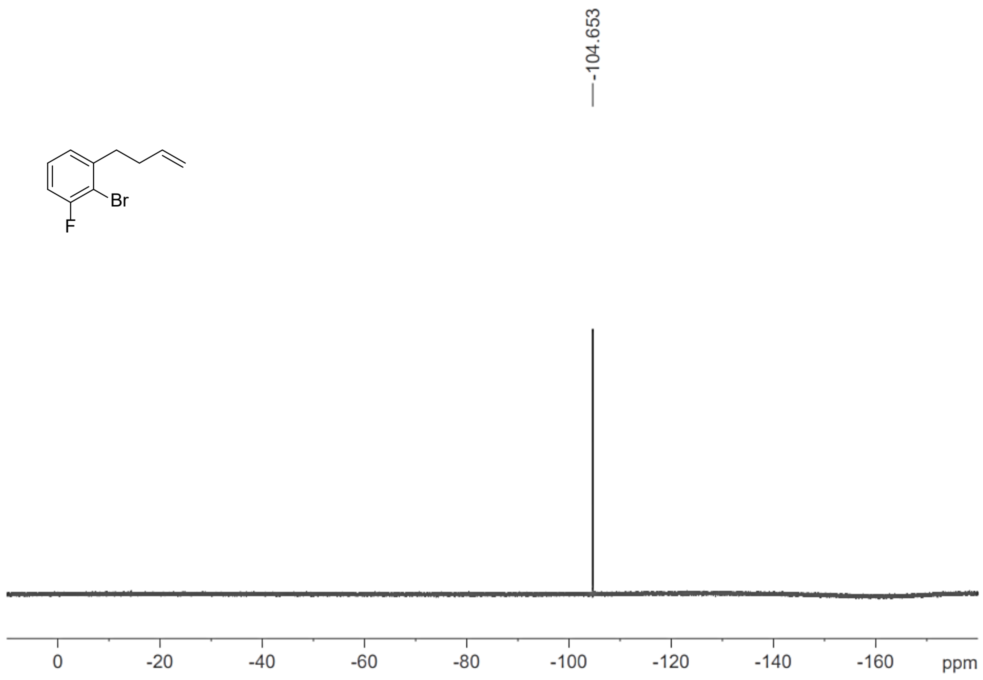

Supplementary Figure 47. ^{1}H NMR spectra of compound **S7** (400 MHz, $CDCl_3$)

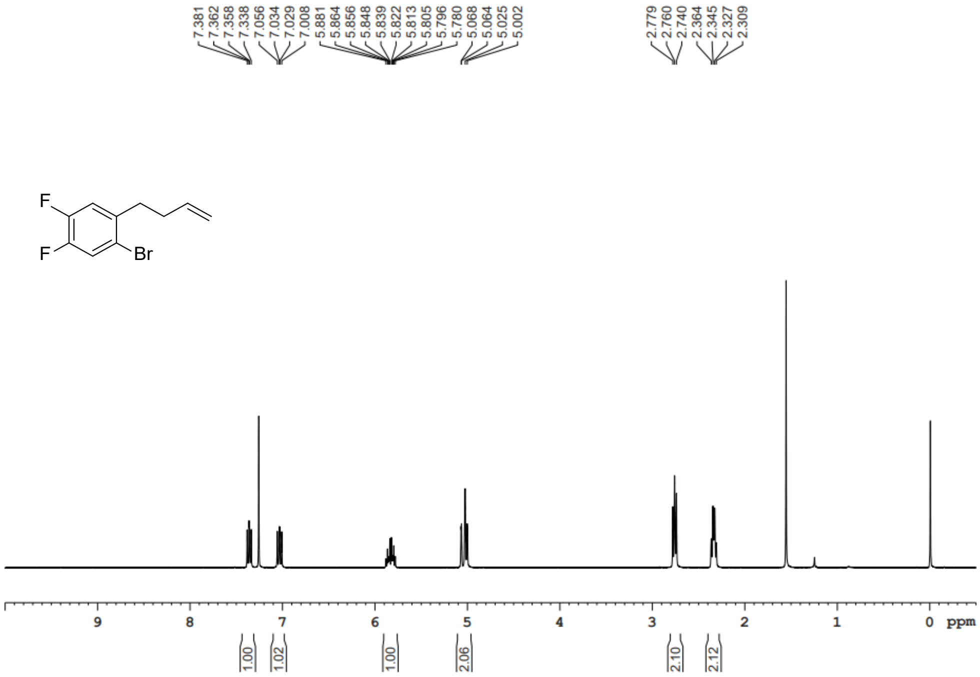

Supplementary Figure 48. ^{13}C NMR spectra of compound **S7** (100 MHz, $CDCl_3$)

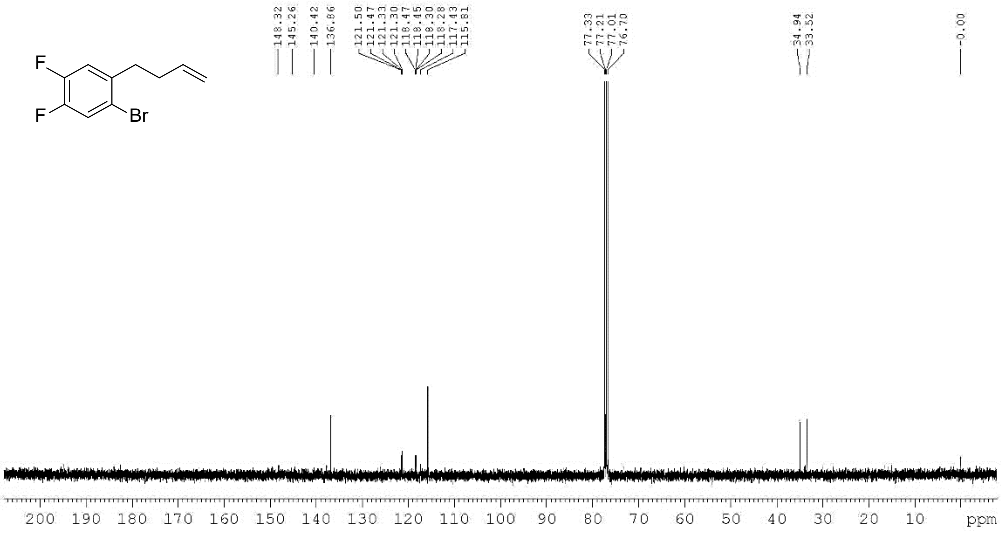

Supplementary Figure 49. ^{19}F NMR spectra of compound **S7** (376 MHz, $CDCl_3$)

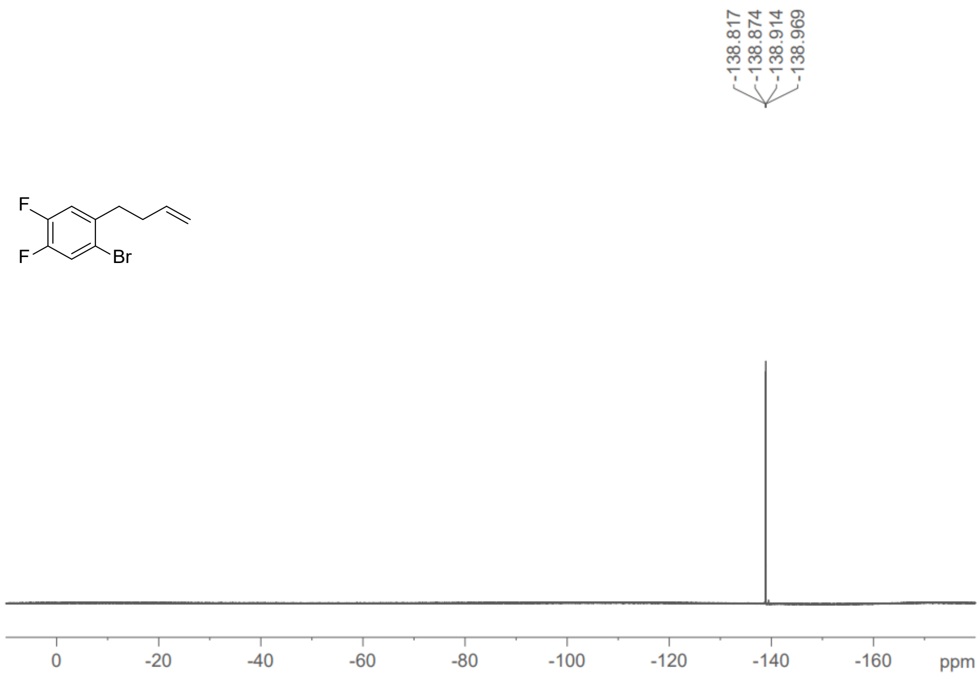

Supplementary Figure 50. ^{1}H NMR spectra of compound **S8** (400 MHz, $CDCl_3$)

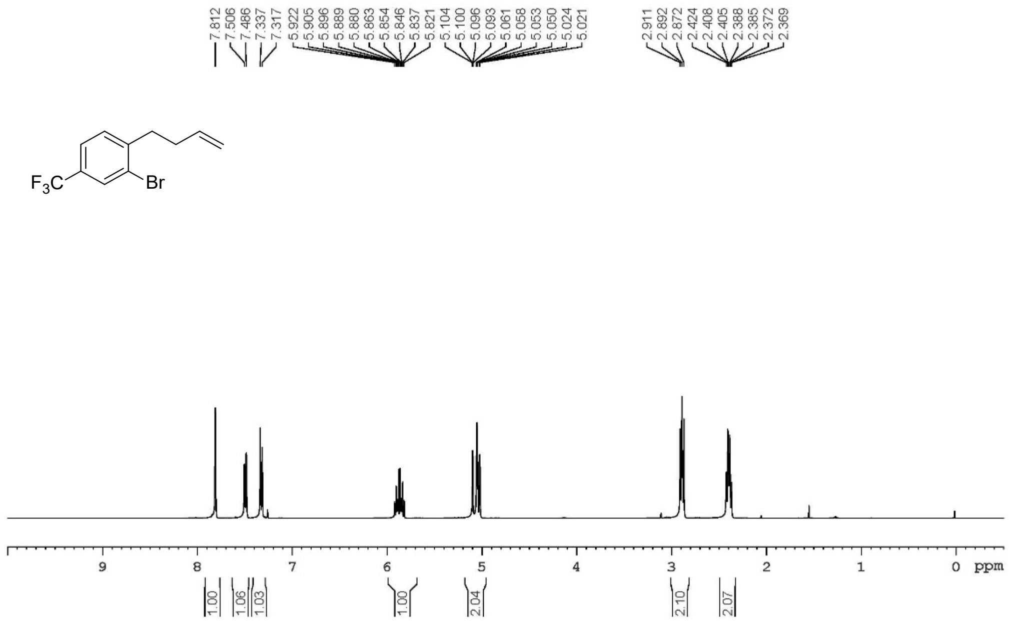

Supplementary Figure 51. ^{13}C NMR spectra of compound **S8** (100 MHz, $CDCl_3$)

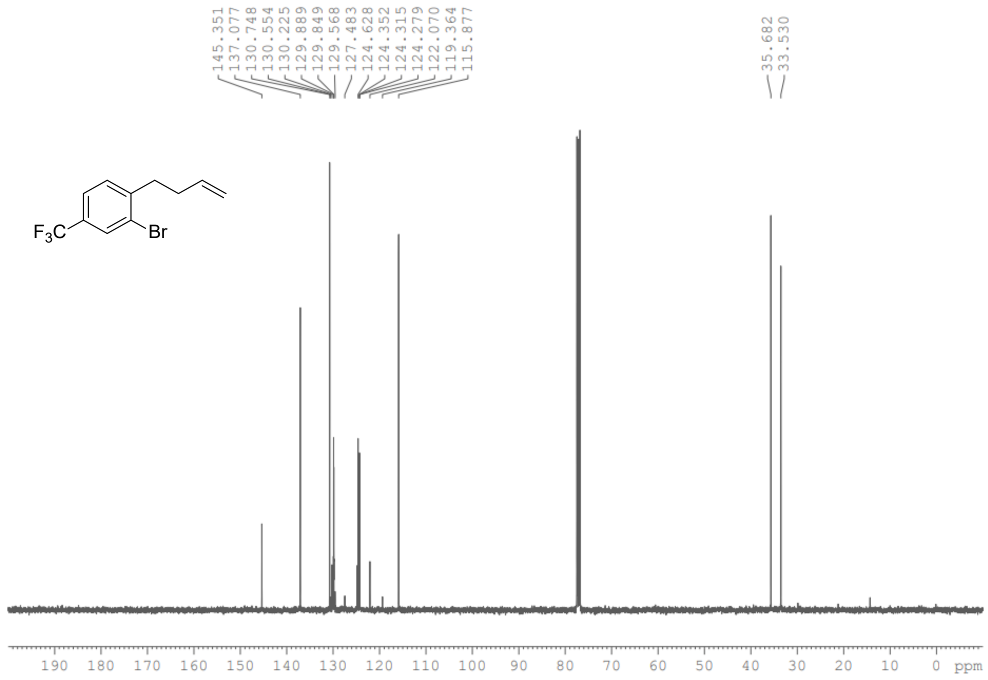

Supplementary Figure 52. ^{19}F NMR spectra of compound **S8** (376 MHz, $CDCl_3$)

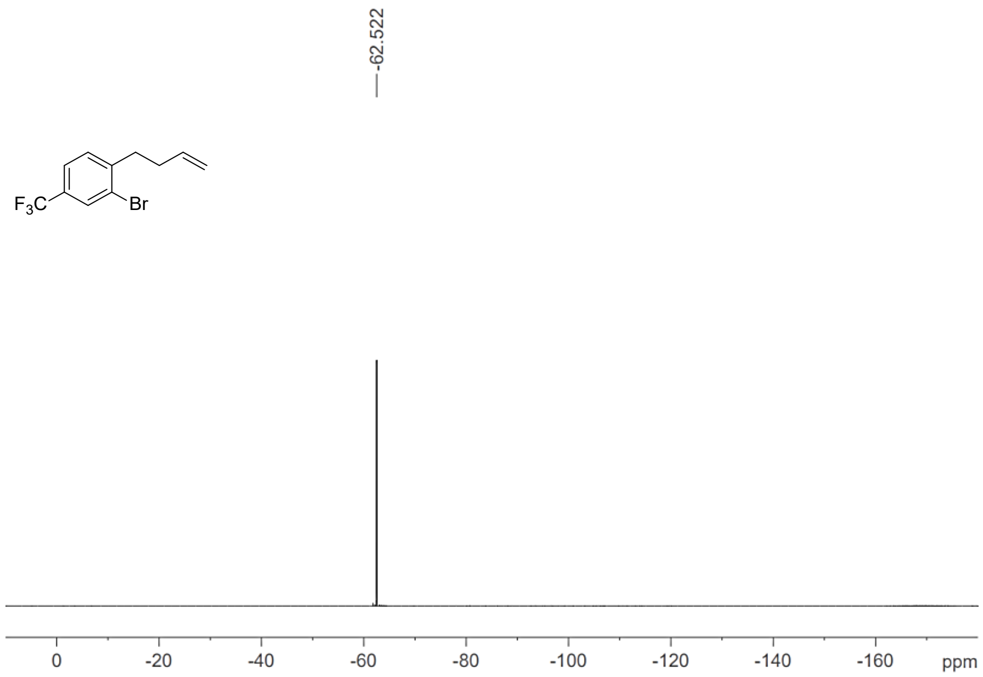

Supplementary Figure 53. ^{1}H NMR spectra of compound **S9** (500 MHz, $CDCl_3$)

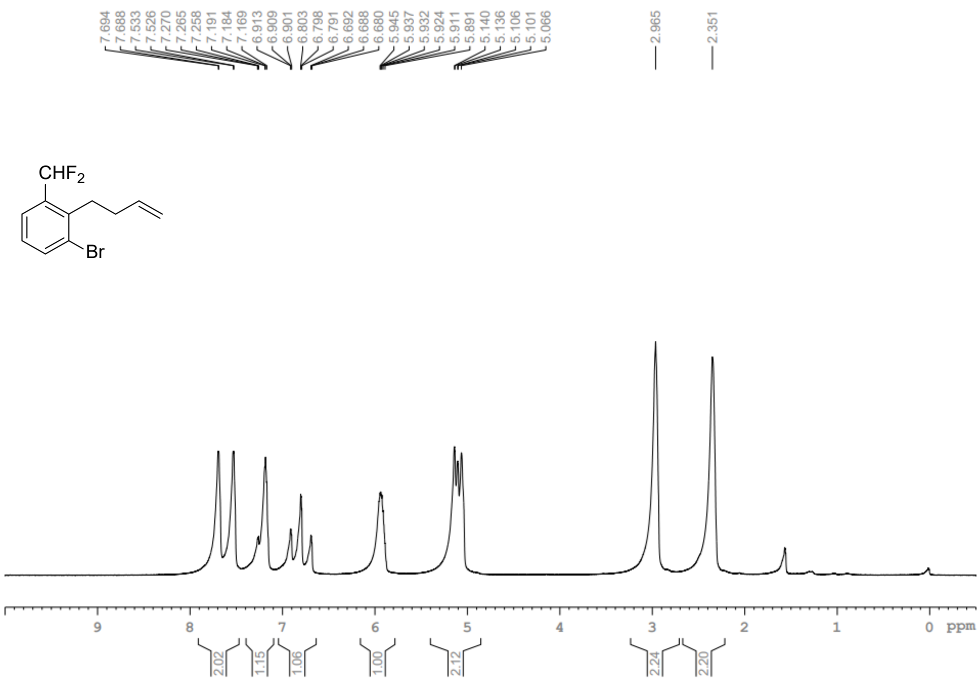

Supplementary Figure 54. ^{13}C NMR spectra of compound **S9** (125 MHz, $CDCl_3$)

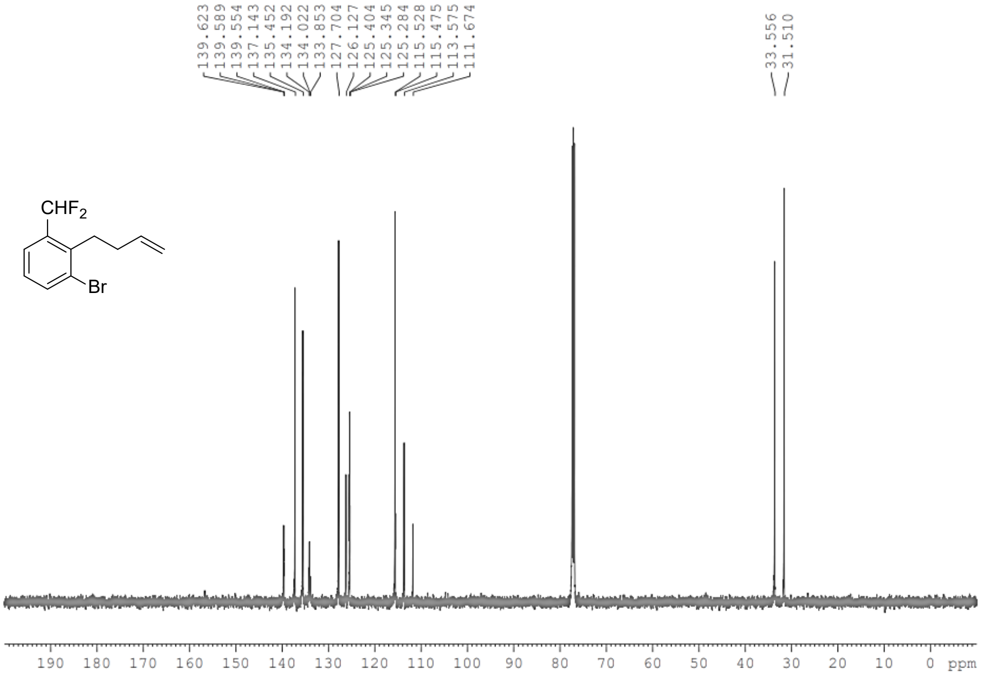

Supplementary Figure 55. ^{19}F NMR spectra of compound **S9** (471 MHz, $CDCl_3$)

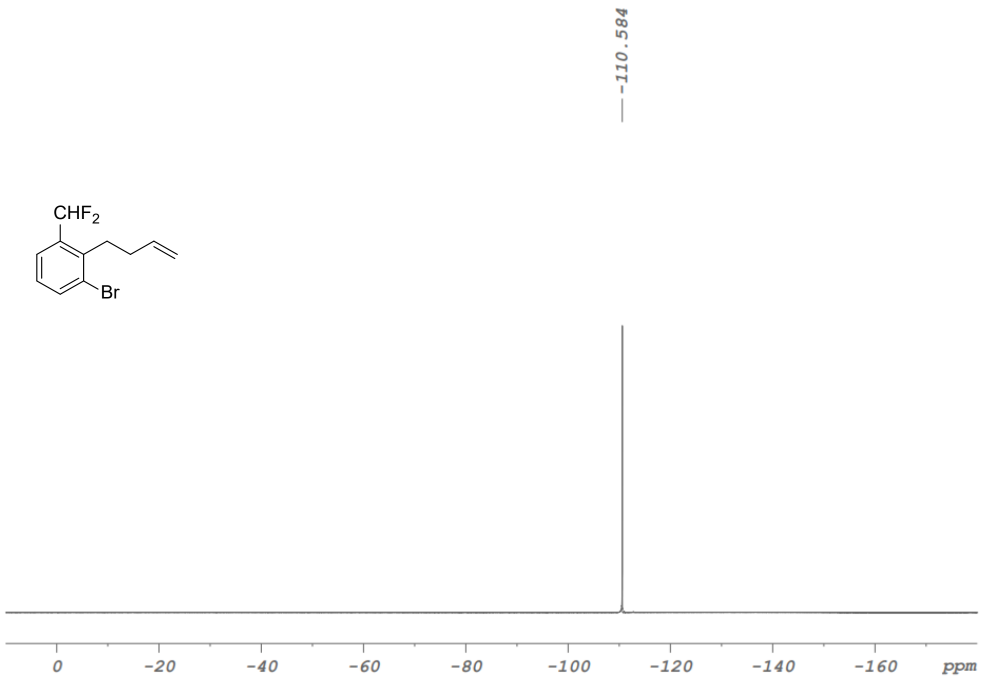

Supplementary Figure 56. ^{1}H NMR spectra of compound **S10** (400 MHz, $CDCl_3$)

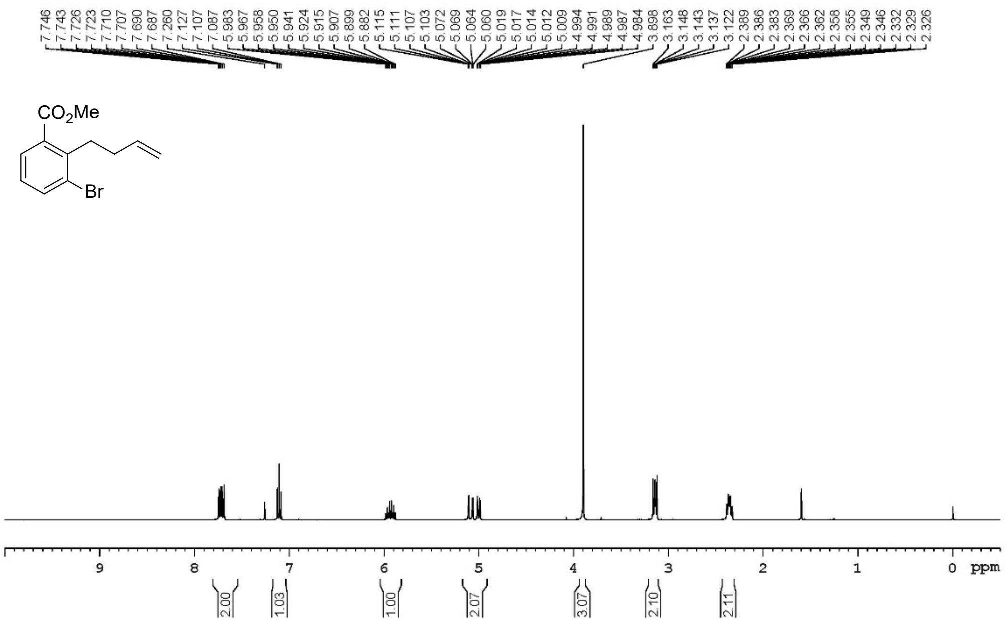

Supplementary Figure 57. ^{13}C NMR spectra of compound **S10** (100 MHz, $CDCl_3$)

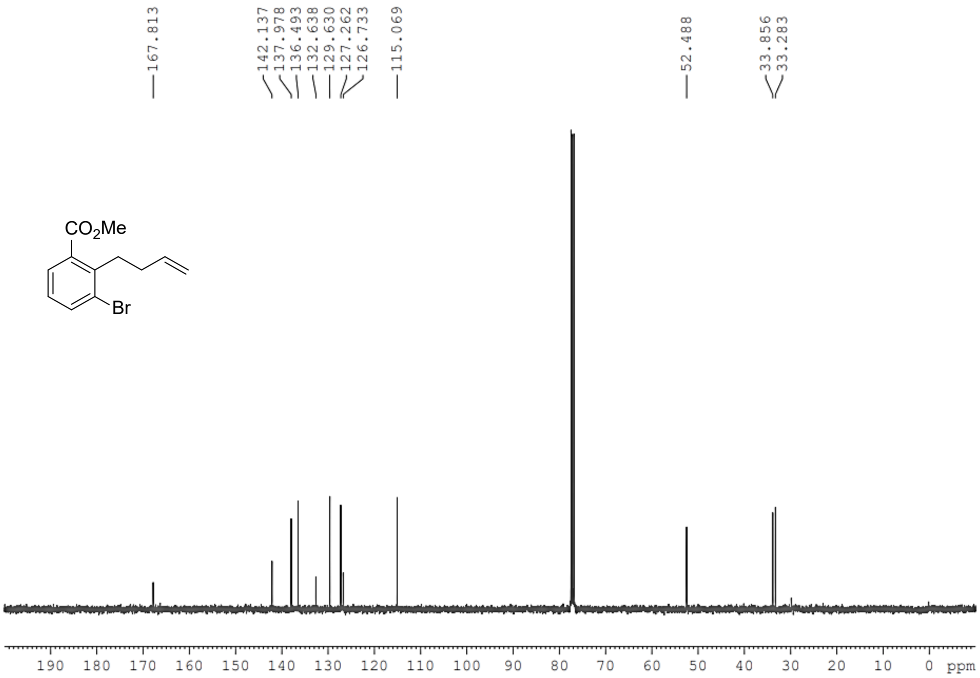

Supplementary Figure 58. ^{1}H NMR spectra of compound **S11** (400 MHz, $CDCl_3$)

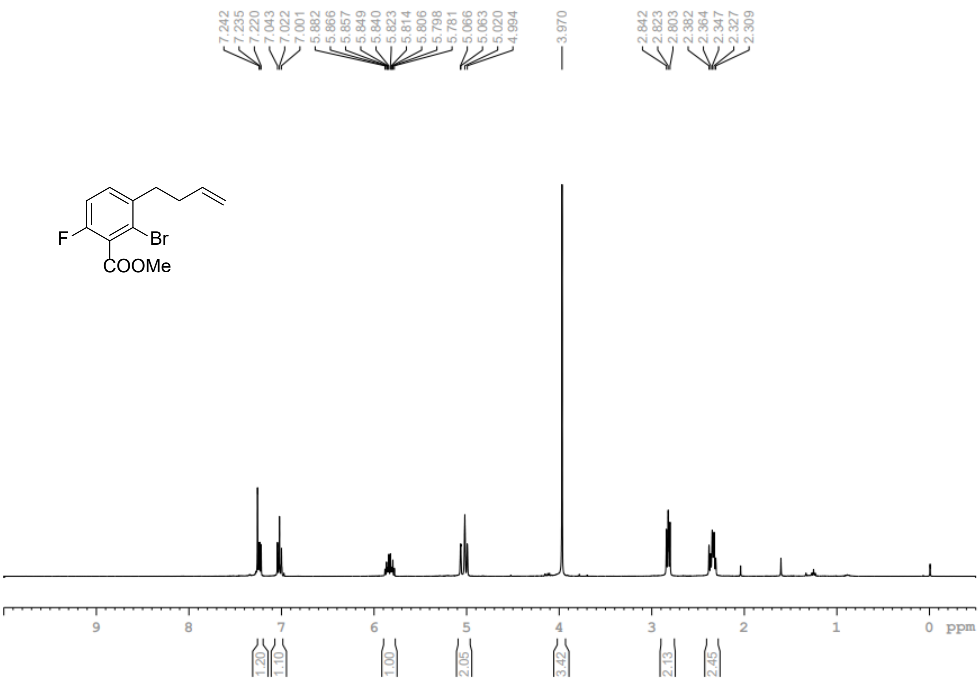

Supplementary Figure 59. ^{13}C NMR spectra of compound **S11** (100 MHz, $CDCl_3$)

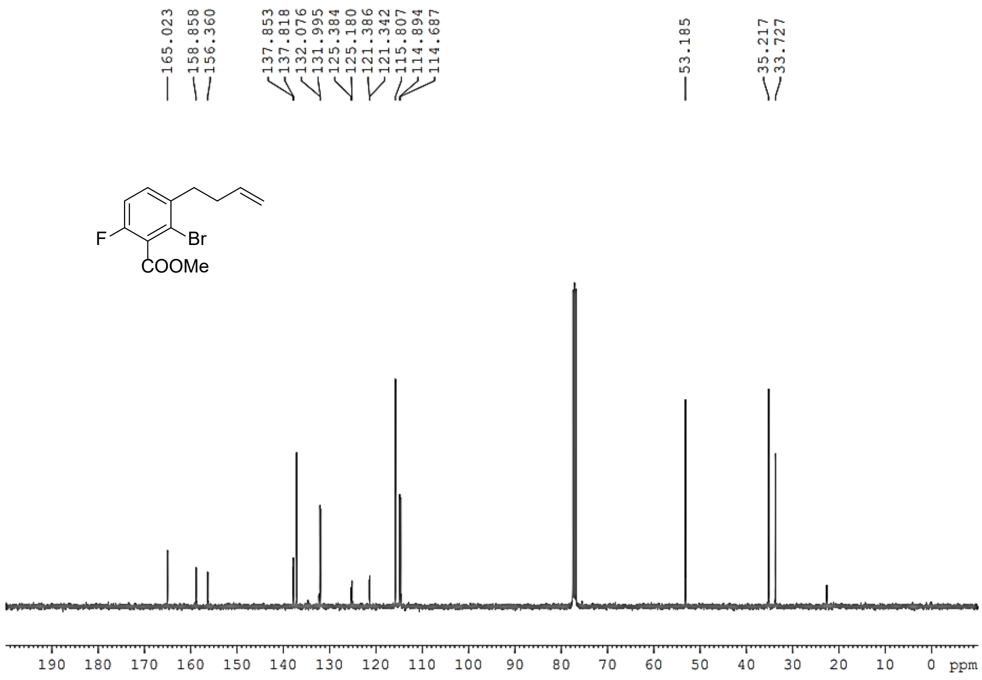

Supplementary Figure 60. ^{19}F NMR spectra of compound **S11** (376 MHz, $CDCl_3$)

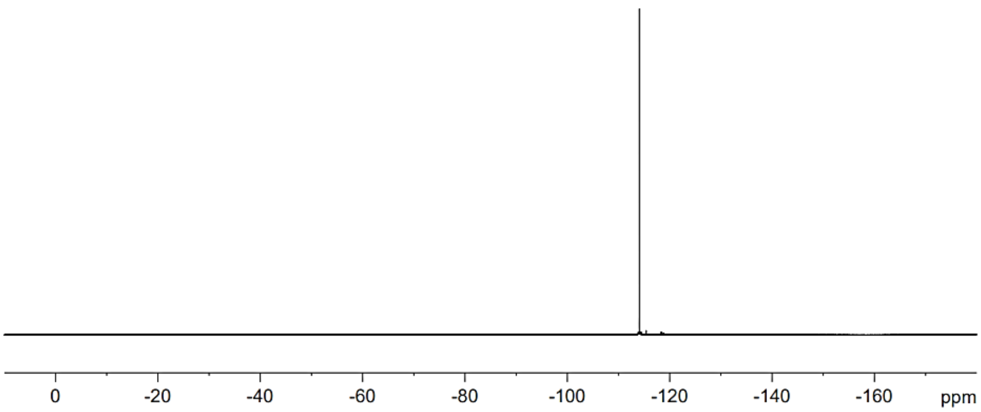

Supplementary Figure 61. ¹H NMR spectra of compound **S12** (400 MHz, $CDCl_3$)

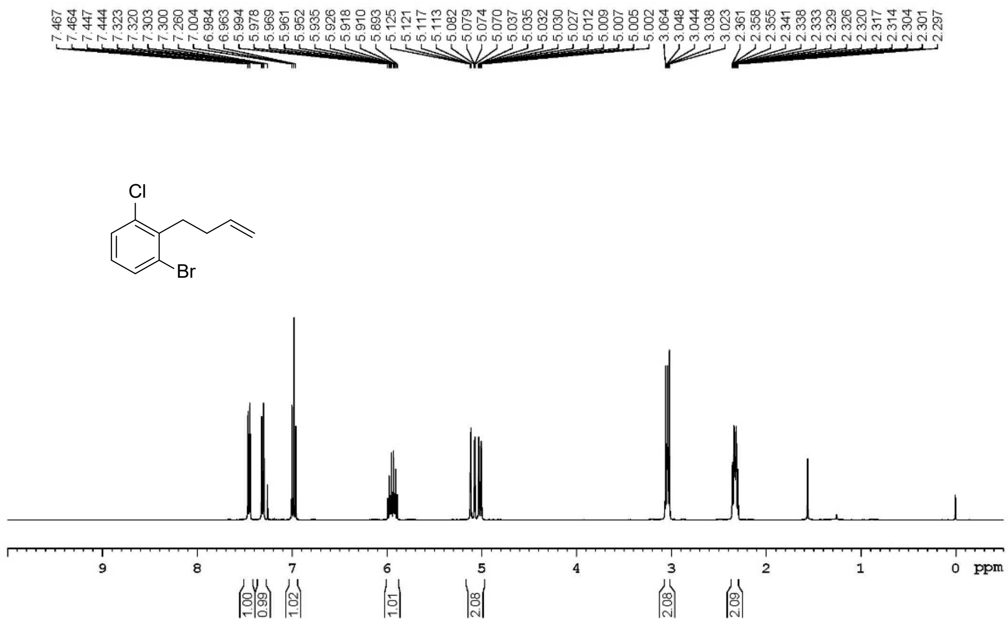

Supplementary Figure 62. ¹³C NMR spectra of compound **S12** (100 MHz, $CDCl_3$)

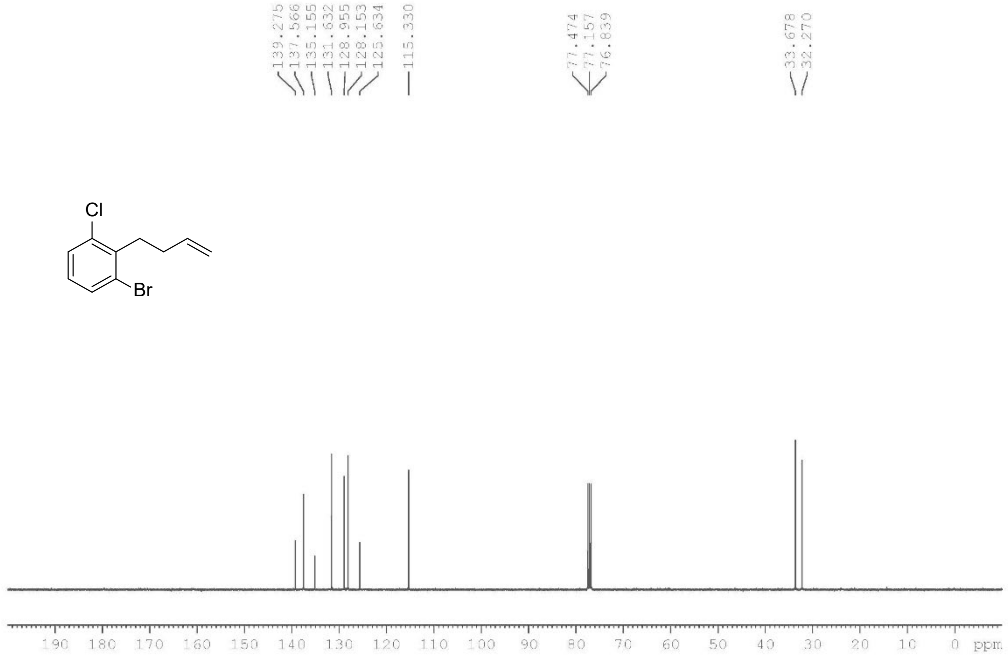

Supplementary Figure 63. ^{1}H NMR spectra of compound **S13** (400 MHz, $CDCl_3$)

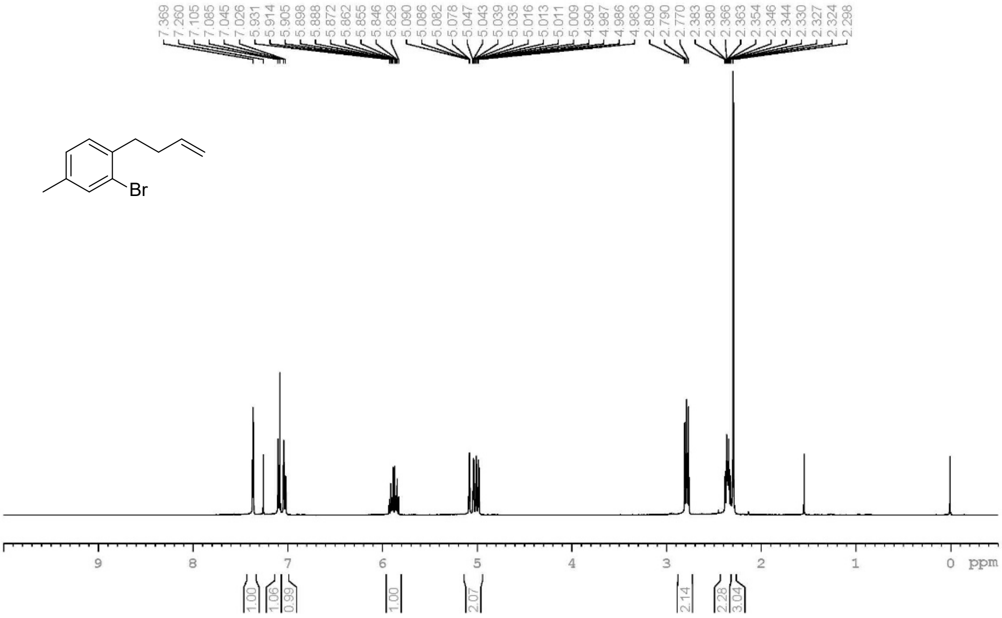

Supplementary Figure 64. ^{13}C NMR spectra of compound **S13** (100 MHz, $CDCl_3$)

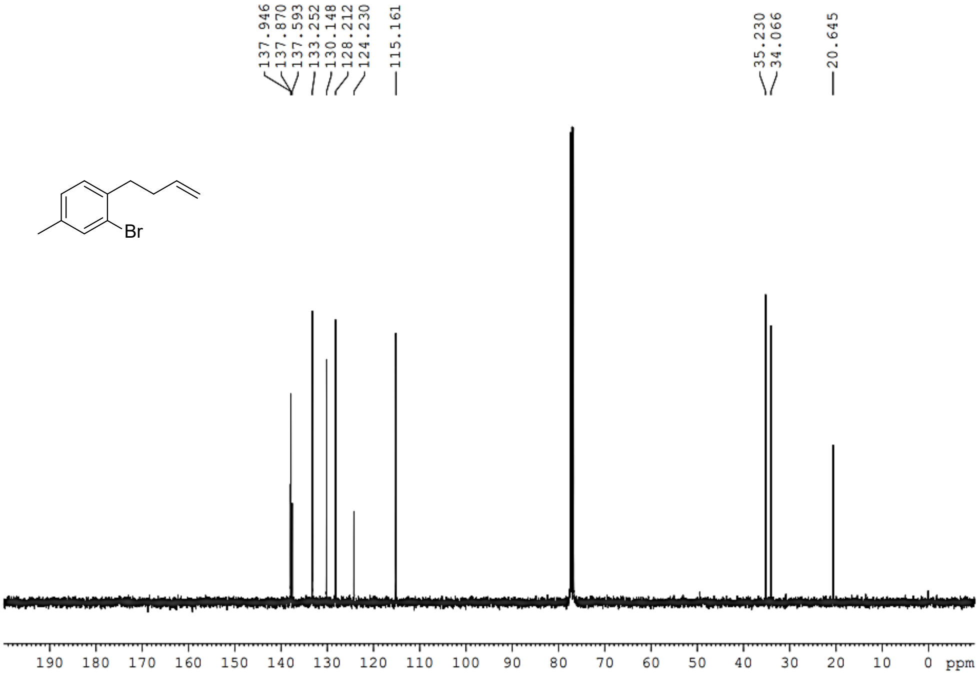

Supplementary Figure 65. ^{1}H NMR spectra of compound **S14** (400 MHz, $CDCl_3$)

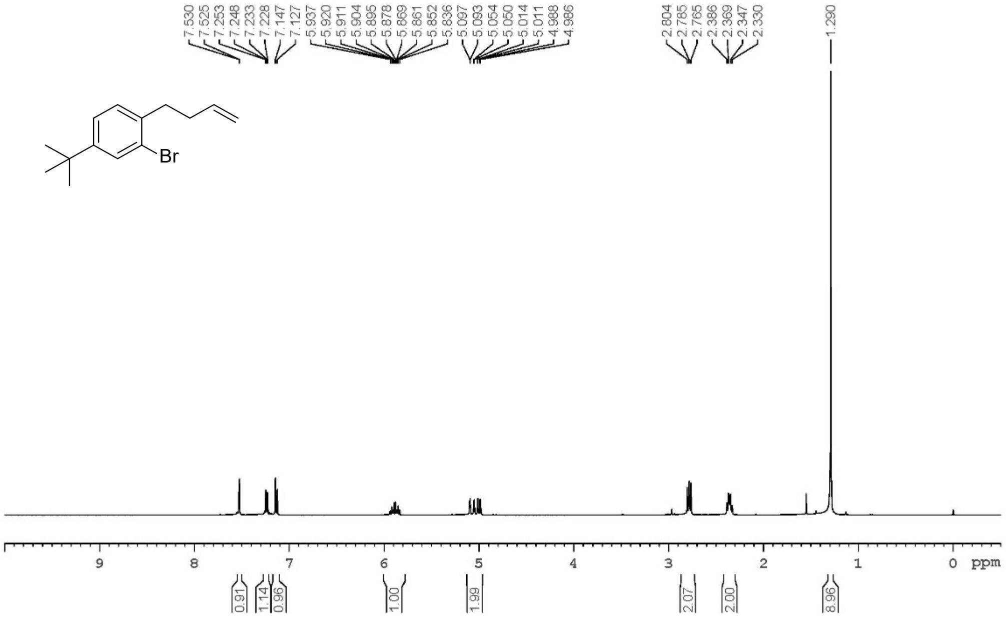

Supplementary Figure 66. ^{13}C NMR spectra of compound **S14** (100 MHz, $CDCl_3$)

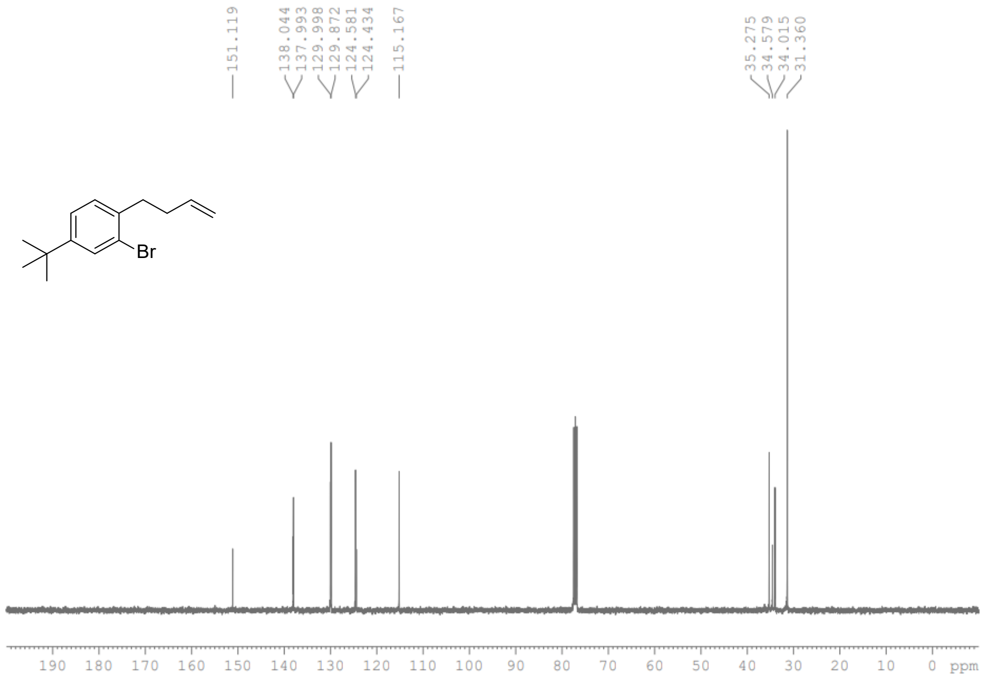

Supplementary Figure 67. ^{1}H NMR spectra of compound **S15** (500 MHz, $CDCl_3$)

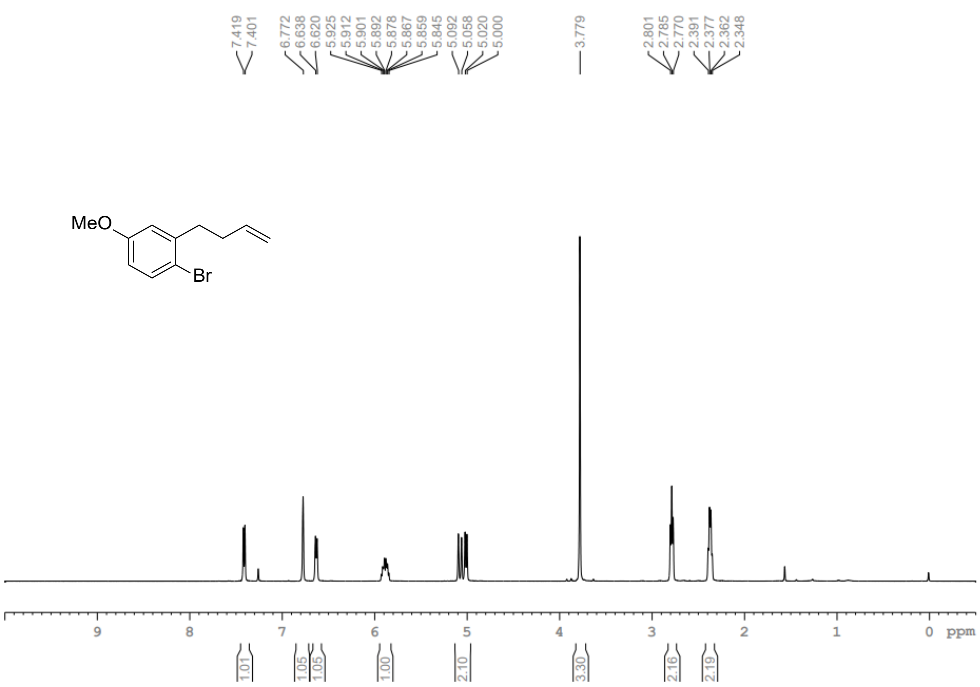

Supplementary Figure 68. ^{13}C NMR spectra of compound **S15** (125 MHz, $CDCl_3$)

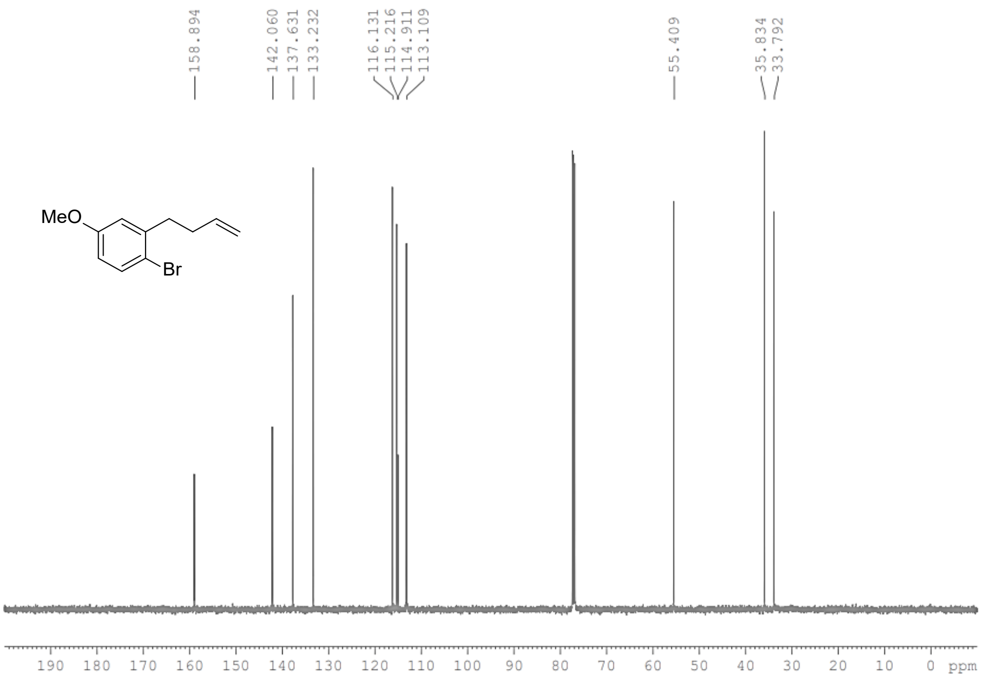

Supplementary Figure 69. ^{1}H NMR spectra of compound **S16** (400 MHz, $CDCl_3$)

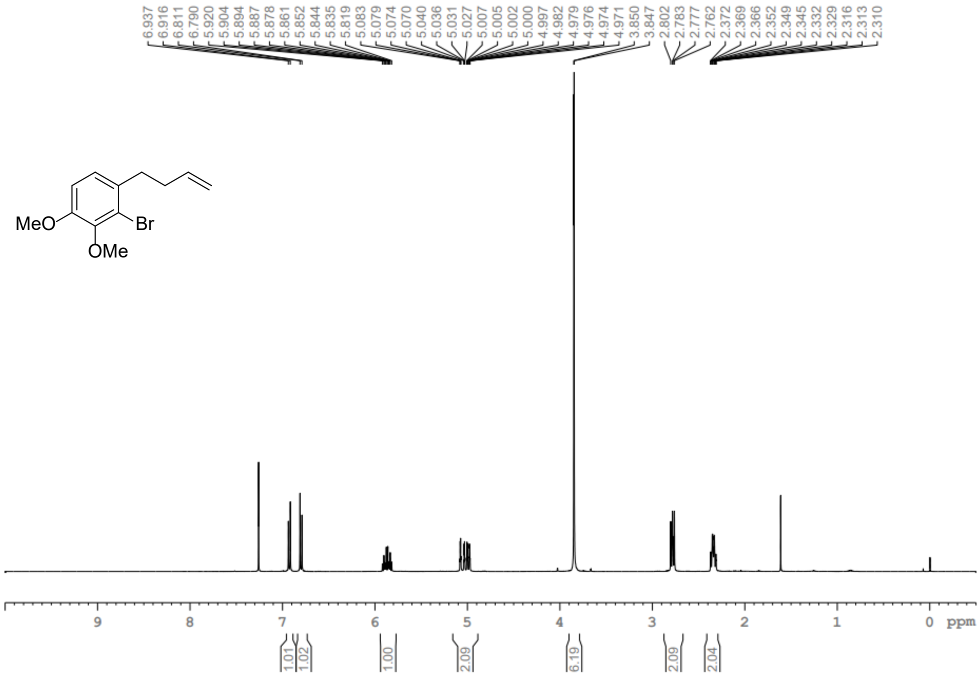

Supplementary Figure 70. ^{13}C NMR spectra of compound **S16** (100 MHz, $CDCl_3$)

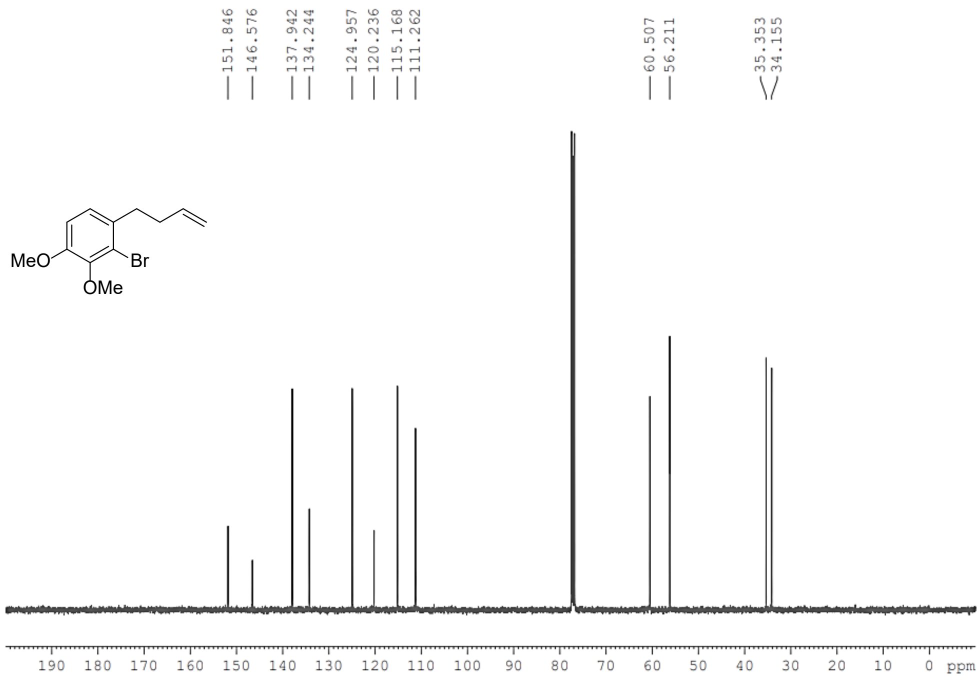

Supplementary Figure 71. ^{1}H NMR spectra of compound **S17** (400 MHz, $CDCl_3$)

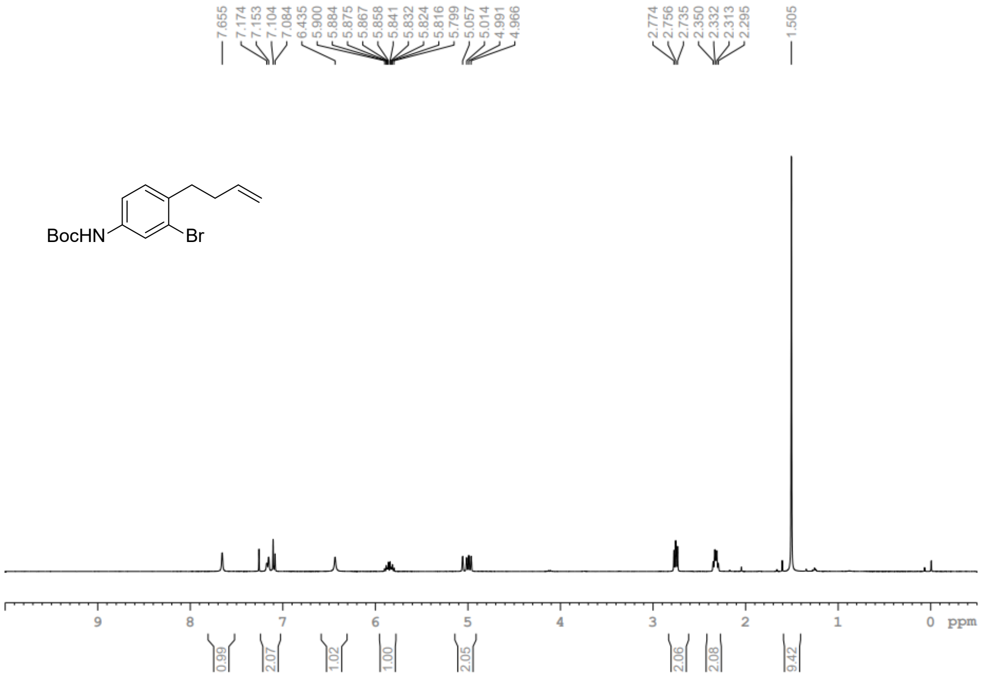

Supplementary Figure 72. ^{13}C NMR spectra of compound **S17** (100 MHz, $CDCl_3$)

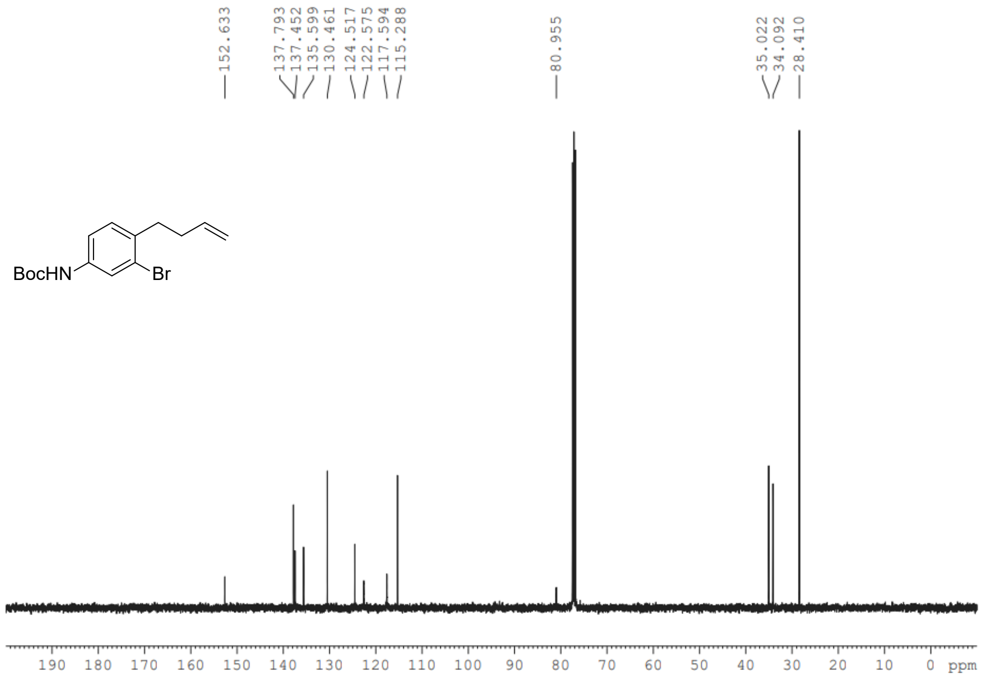

Supplementary Figure 73. ^{1}H NMR spectra of compound **S18** (400 MHz, $CDCl_3$)

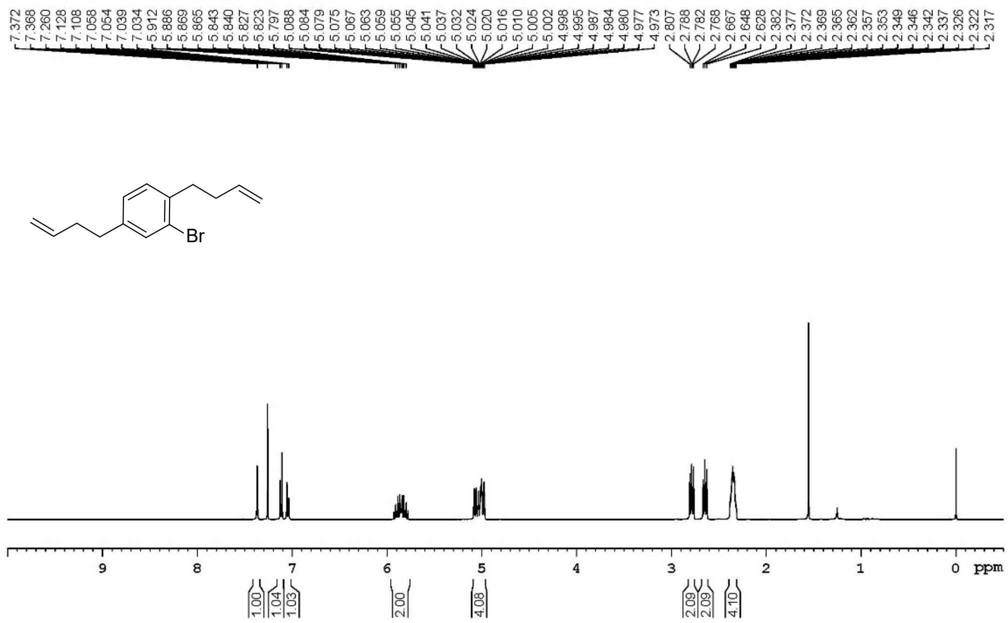

Supplementary Figure 74. ^{13}C NMR spectra of compound **S18** (100 MHz, $CDCl_3$)

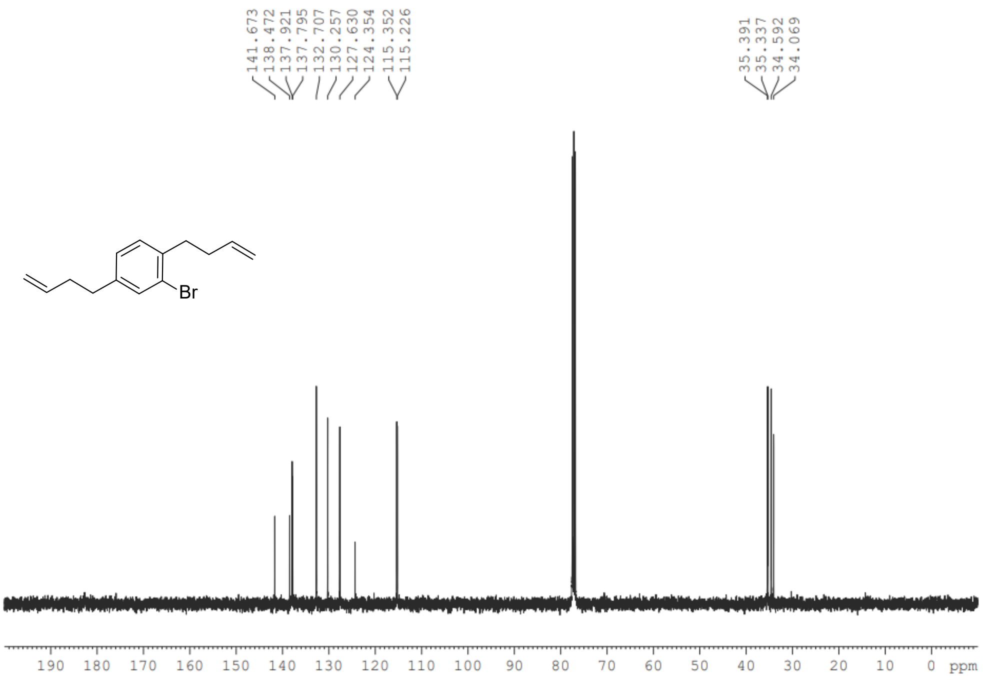

Supplementary Figure 75. ^{1}H NMR spectra of compound **S19** (400 MHz, $CDCl_3$)

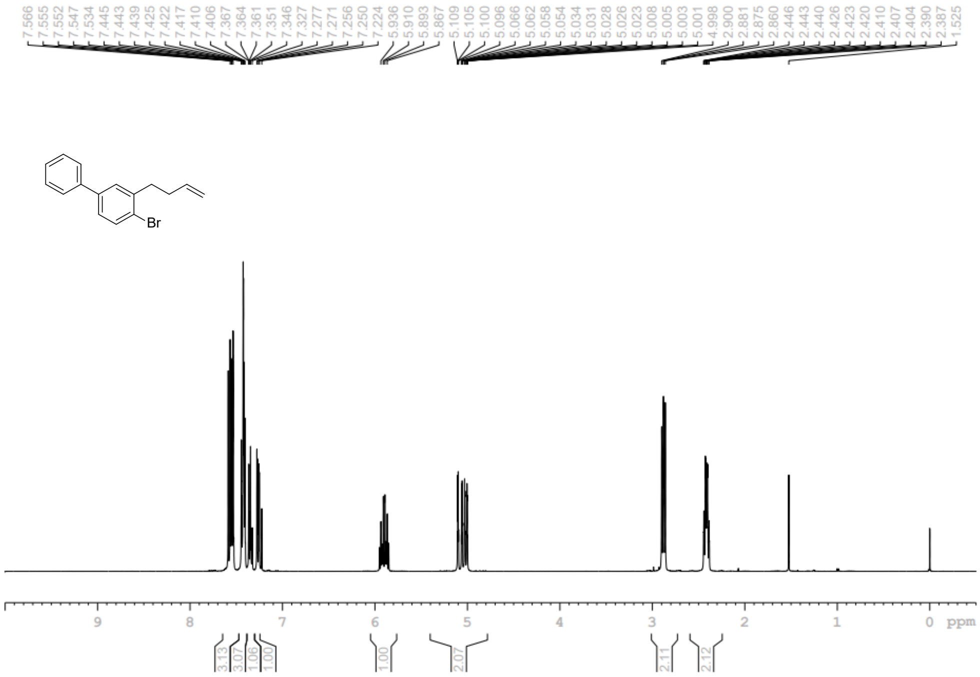

Supplementary Figure 76. ^{13}C NMR spectra of compound **S19** (100 MHz, $CDCl_3$)

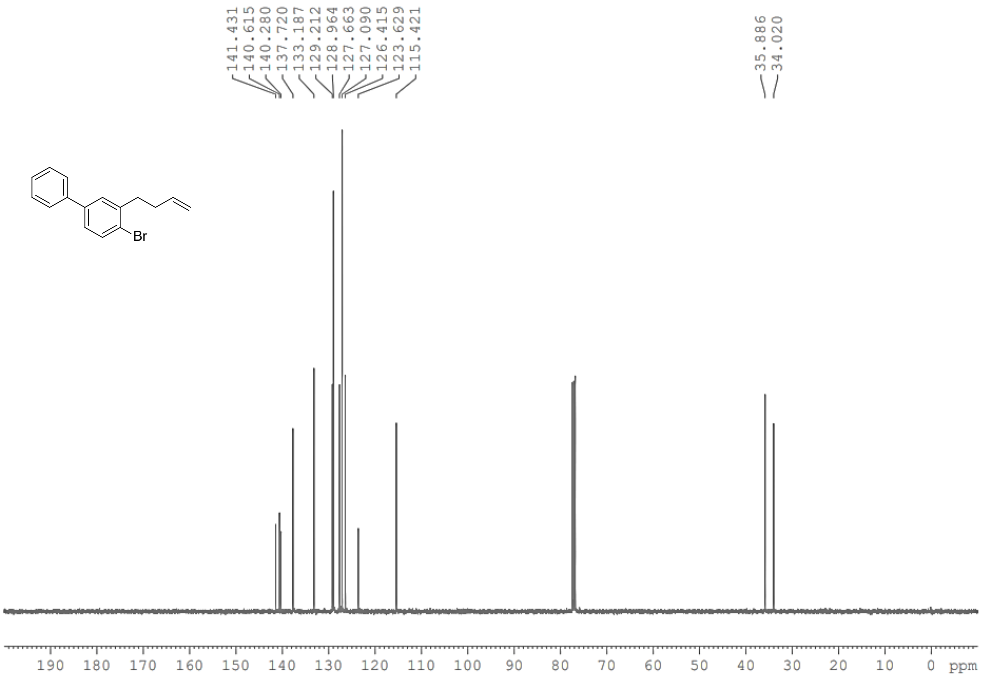

Supplementary Figure 77. ^{1}H NMR spectra of compound **S20** (400 MHz, $CDCl_3$)

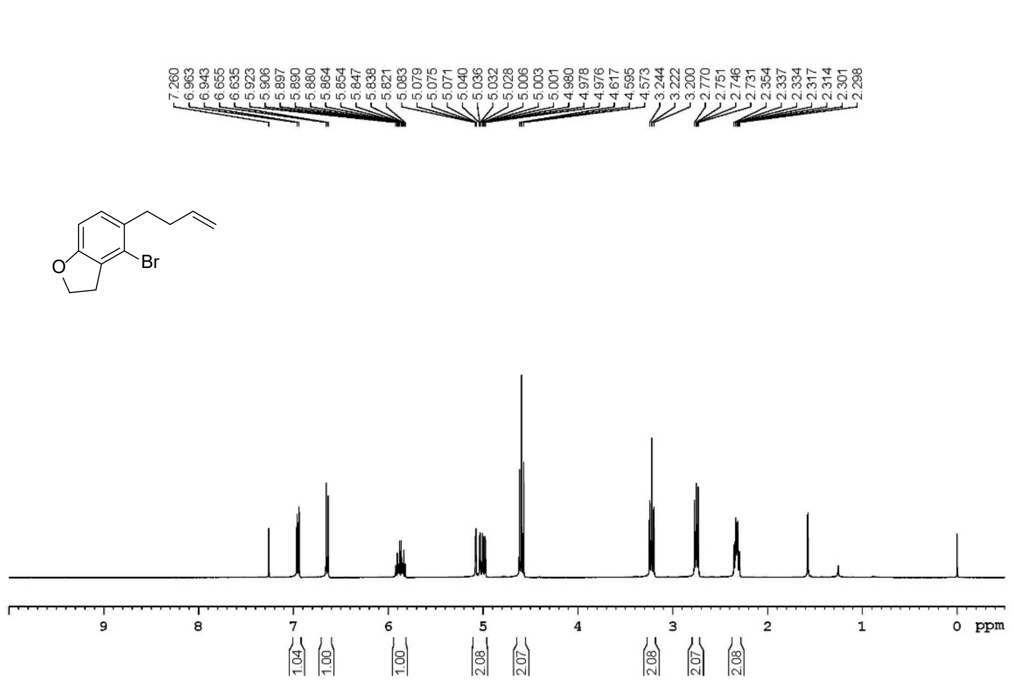

Supplementary Figure 78. ^{13}C NMR spectra of compound **S20** (100 MHz, $CDCl_3$)

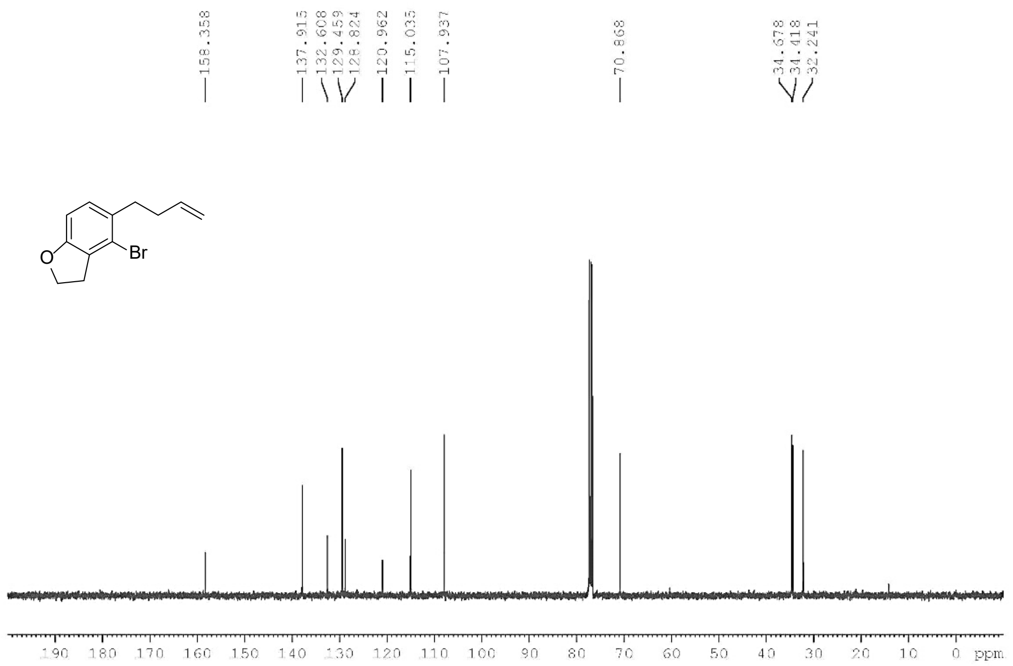

Supplementary Figure 79. ^{1}H NMR spectra of compound **S21** (400 MHz, $CDCl_3$)

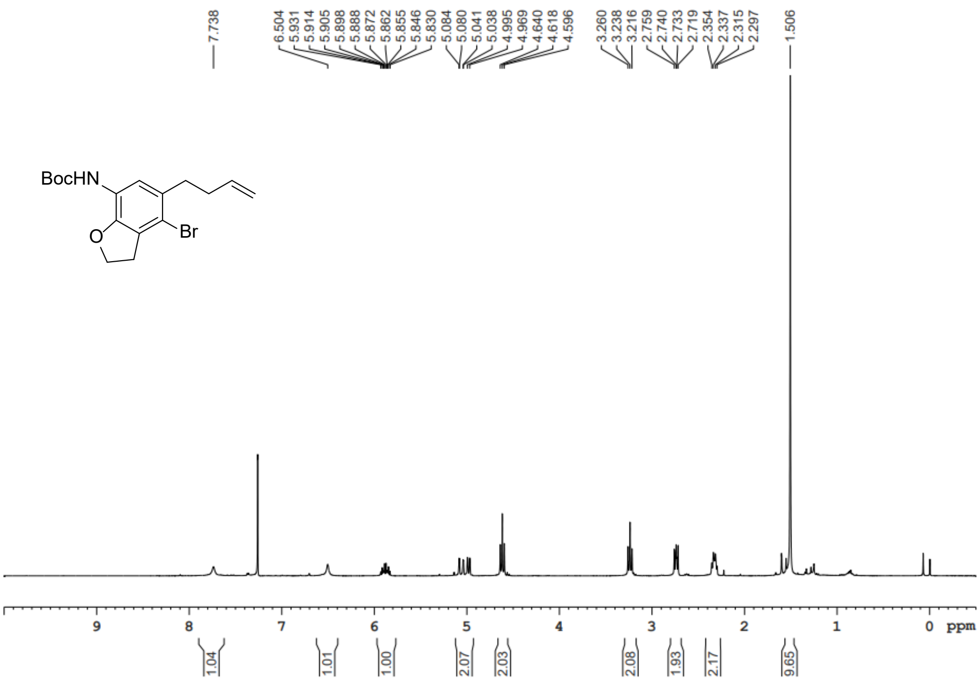

Supplementary Figure 80. ^{13}C NMR spectra of compound **S21** (100 MHz, $CDCl_3$)

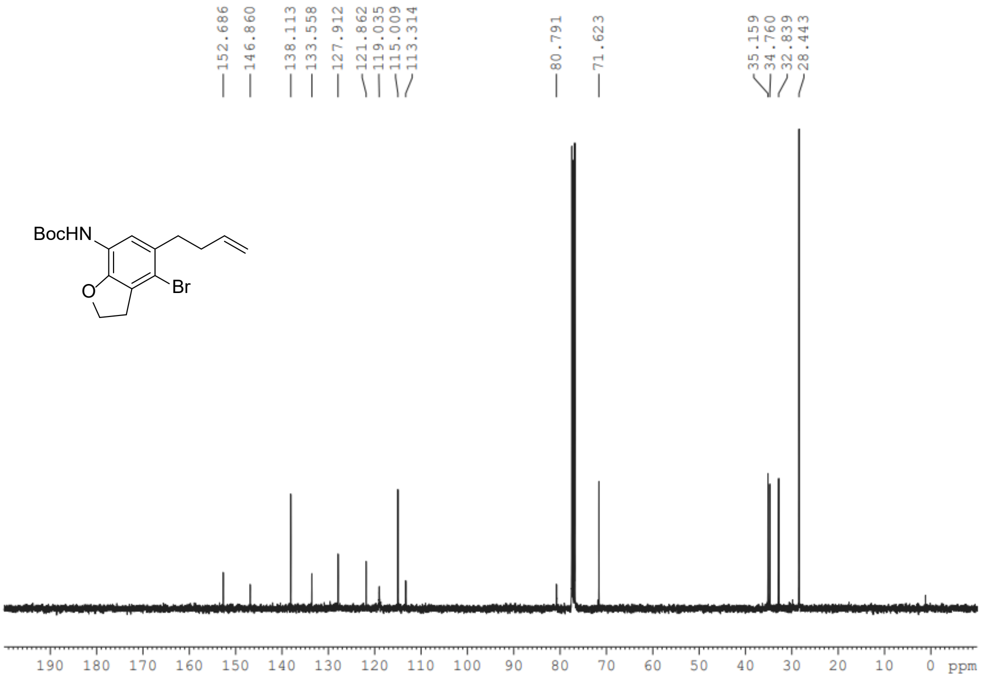

Supplementary Figure 81. ^{1}H NMR spectra of compound **S22** (400 MHz, $CDCl_3$)

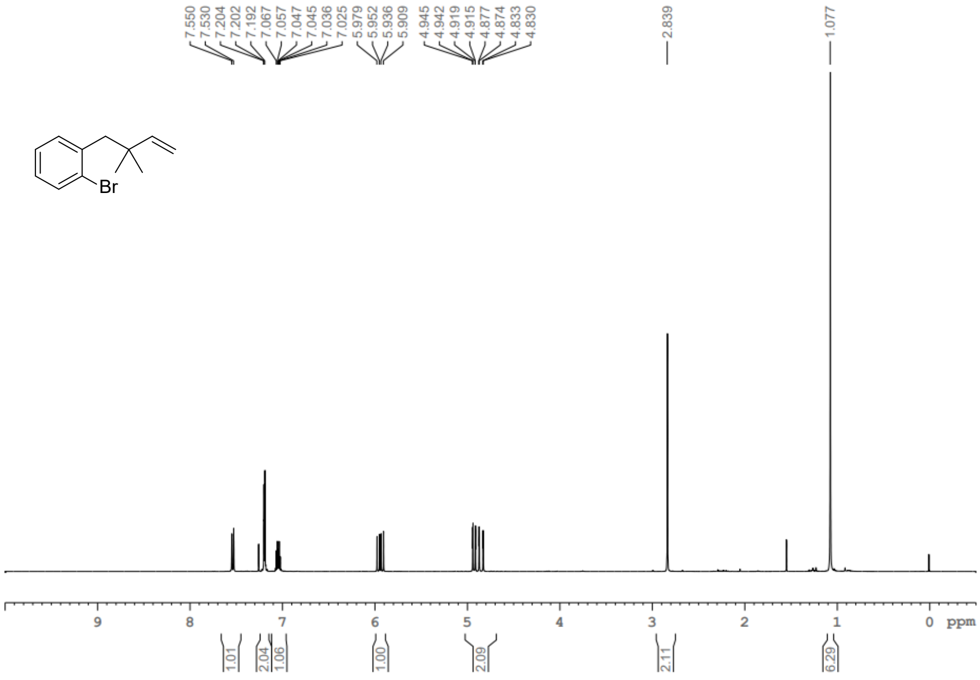

Supplementary Figure 82. ^{13}C NMR spectra of compound **S22** (100 MHz, $CDCl_3$)

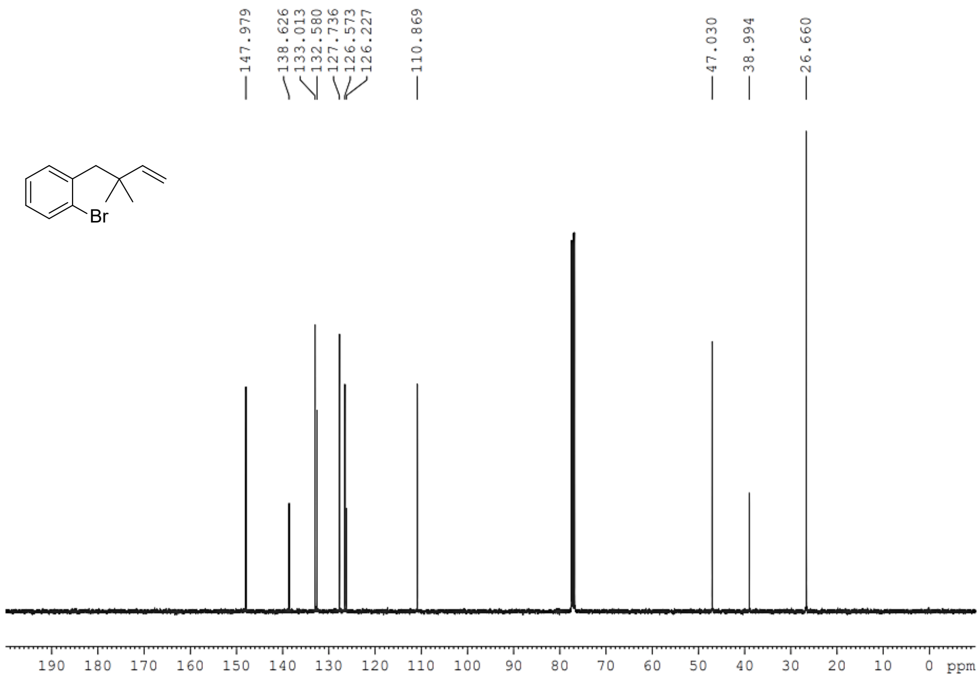

Supplementary Figure 83. ^{1}H NMR spectra of compound **S23** (400 MHz, $CDCl_3$)

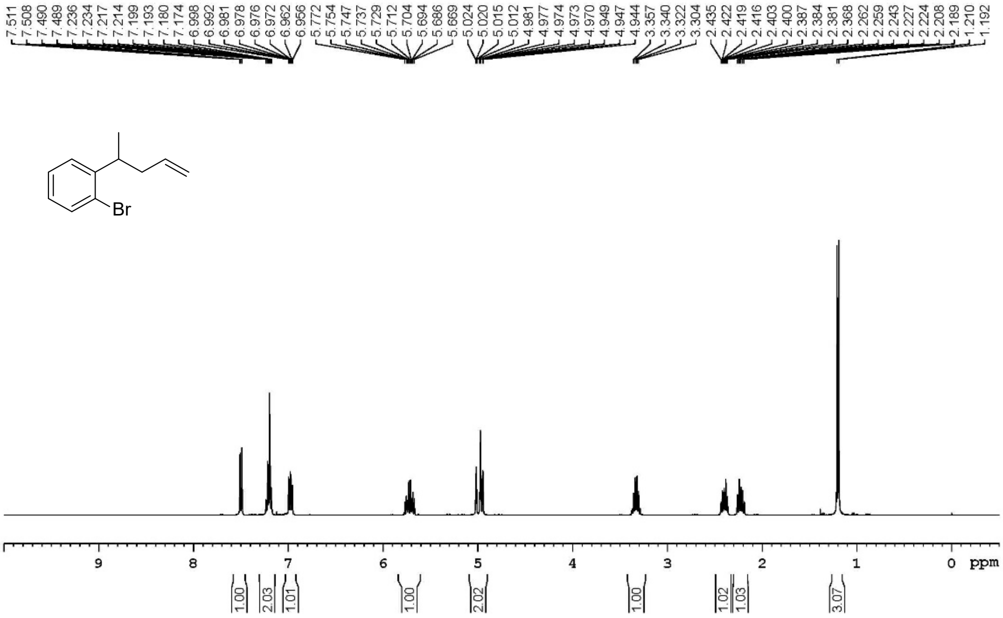

Supplementary Figure 84. ^{13}C NMR spectra of compound **S23** (100 MHz, $CDCl_3$)

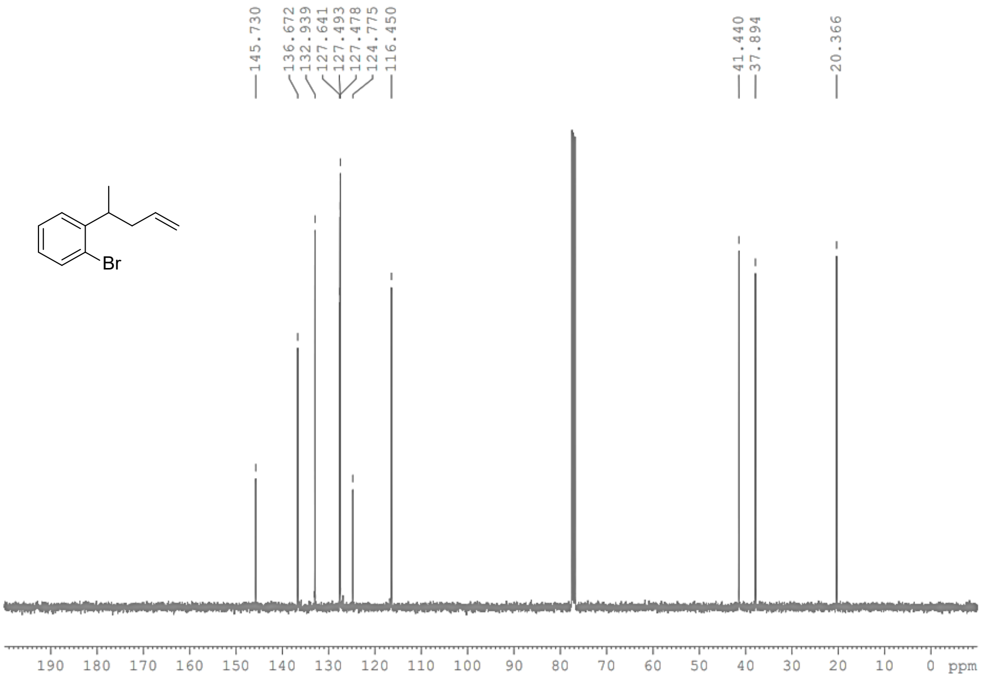

Supplementary Figure 85. ^{1}H NMR spectra of compound **S24** (400 MHz, $CDCl_3$)

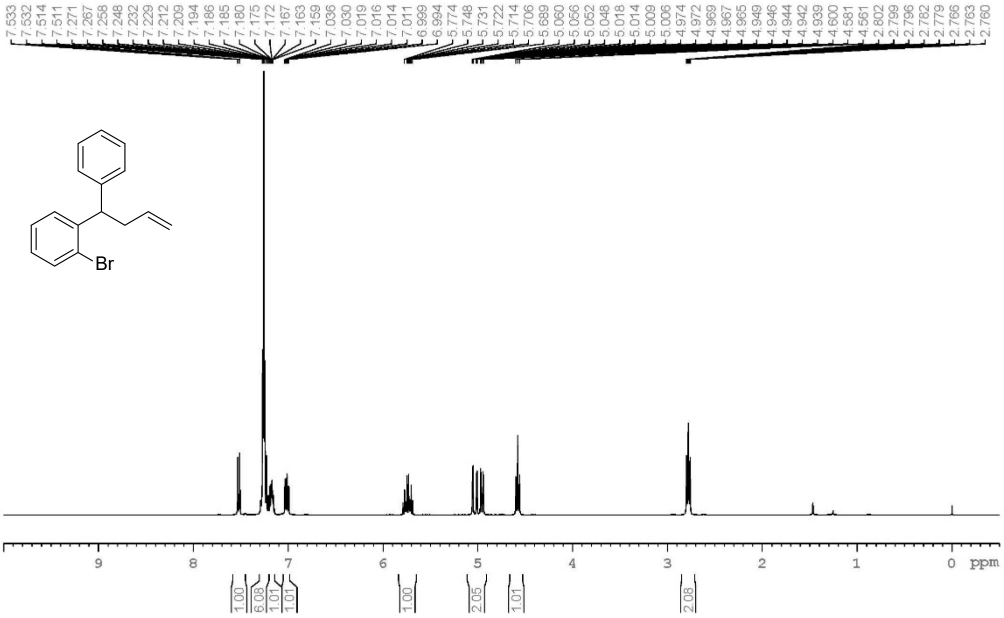

Supplementary Figure 86. ^{13}C NMR spectra of compound **S24** (100 MHz, $CDCl_3$)

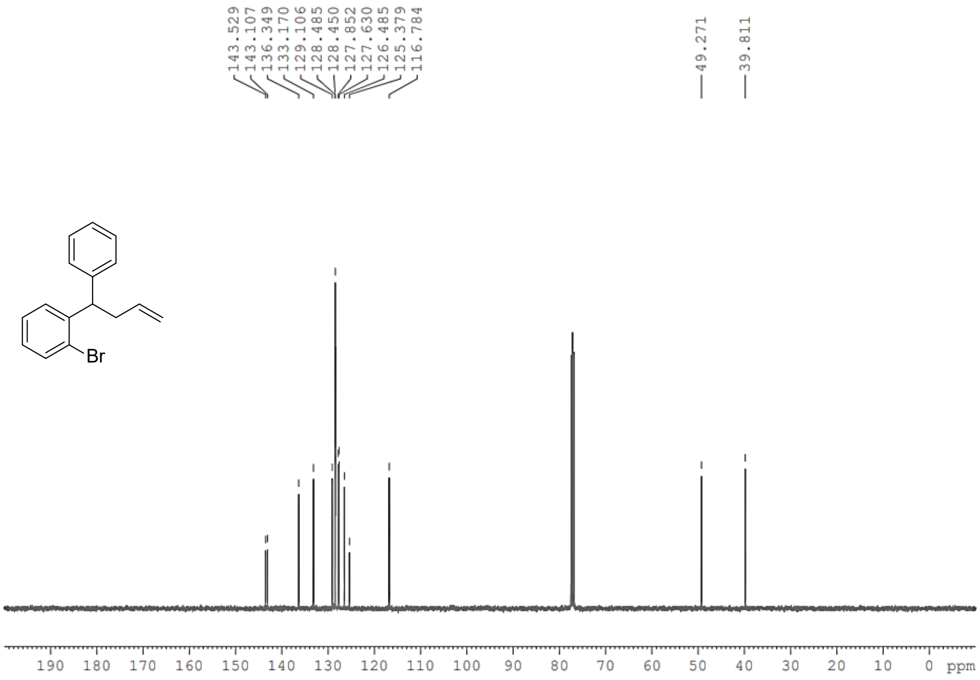

Supplementary Figure 87. ^{1}H NMR spectra of compound **S25** (400 MHz, $CDCl_3$)

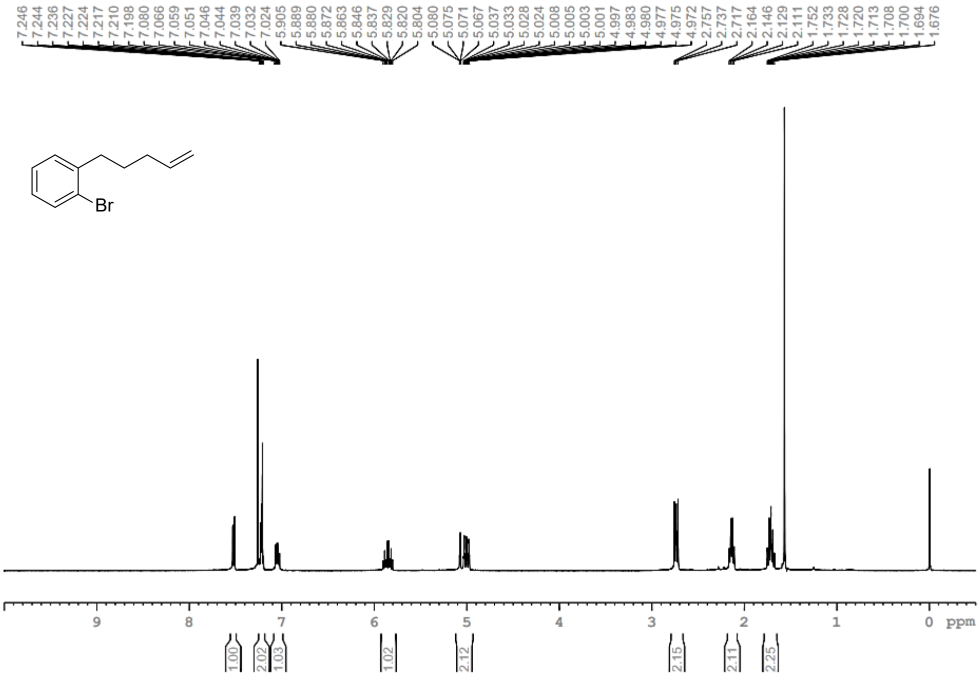

Supplementary Figure 88. ^{13}C NMR spectra of compound **S25** (100 MHz, $CDCl_3$)

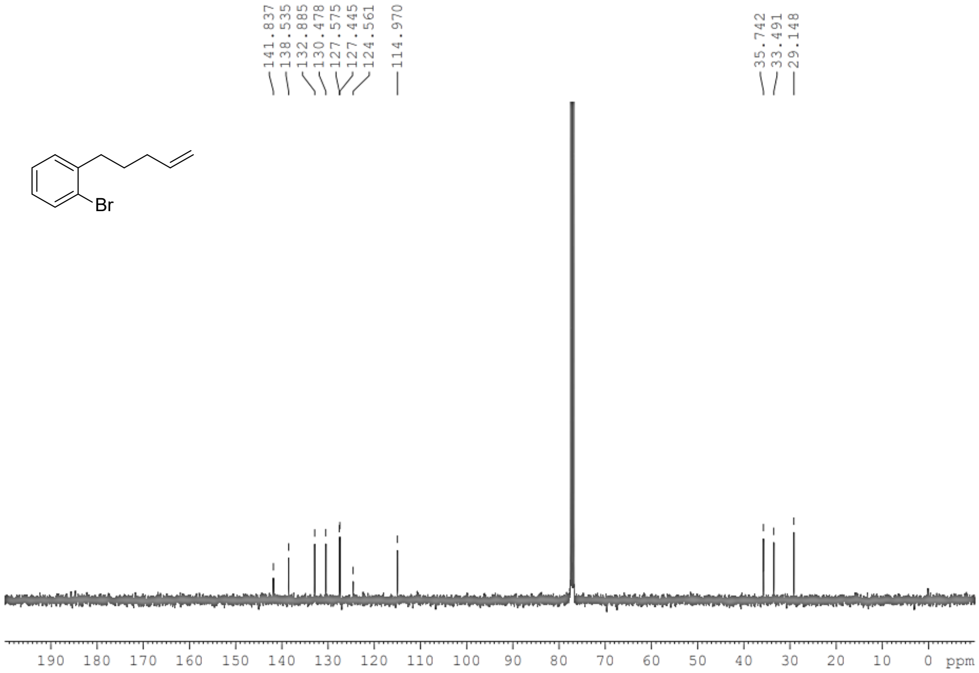

Supplementary Figure 89. ^{1}H NMR spectra of compound **S26** (400 MHz, $CDCl_3$)

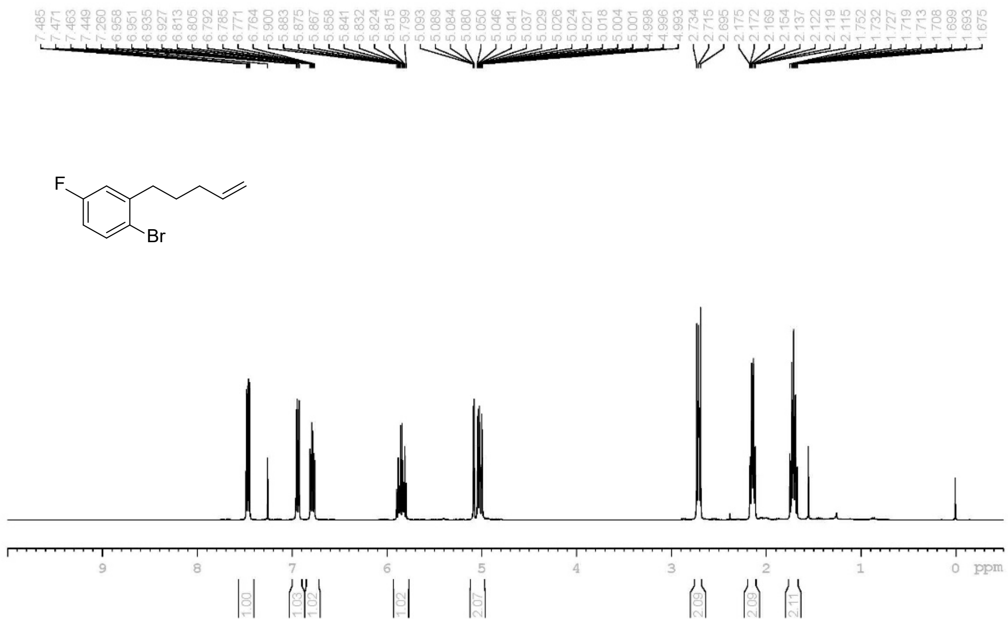

Supplementary Figure 90. ^{13}C NMR spectra of compound **S26** (100 MHz, $CDCl_3$)

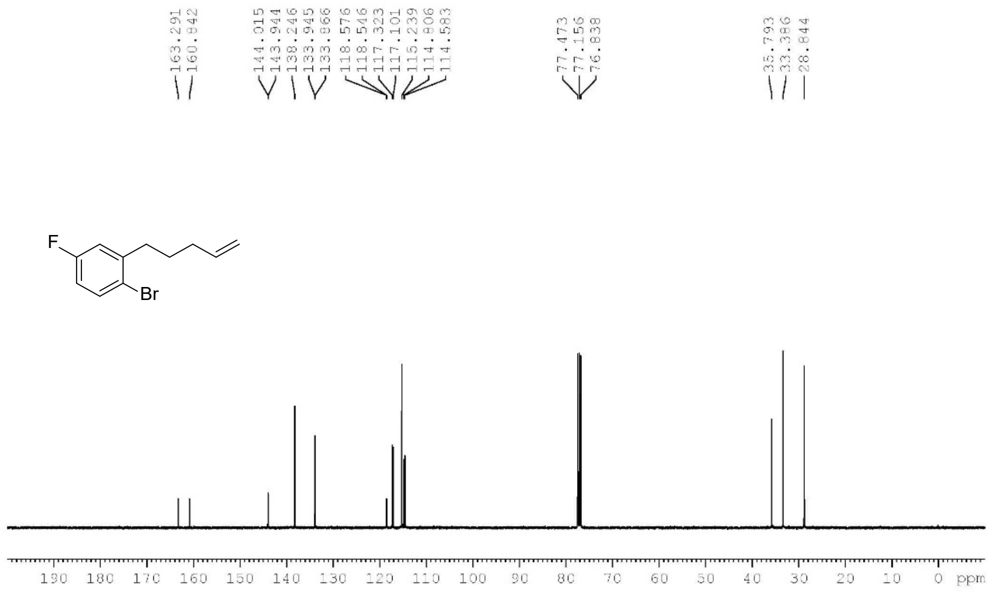

Supplementary Figure 91. ^{19}F NMR spectra of compound **S26** (376 MHz, $CDCl_3$)

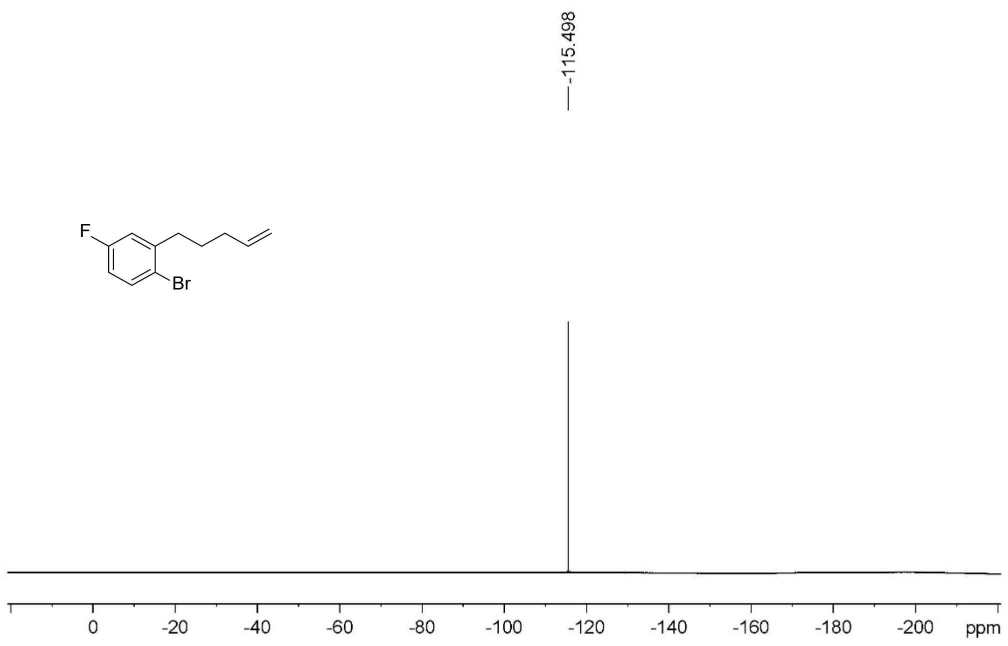

Supplementary Figure 92. ^{1}H NMR spectra of compound **S27** (400 MHz, $CDCl_3$)

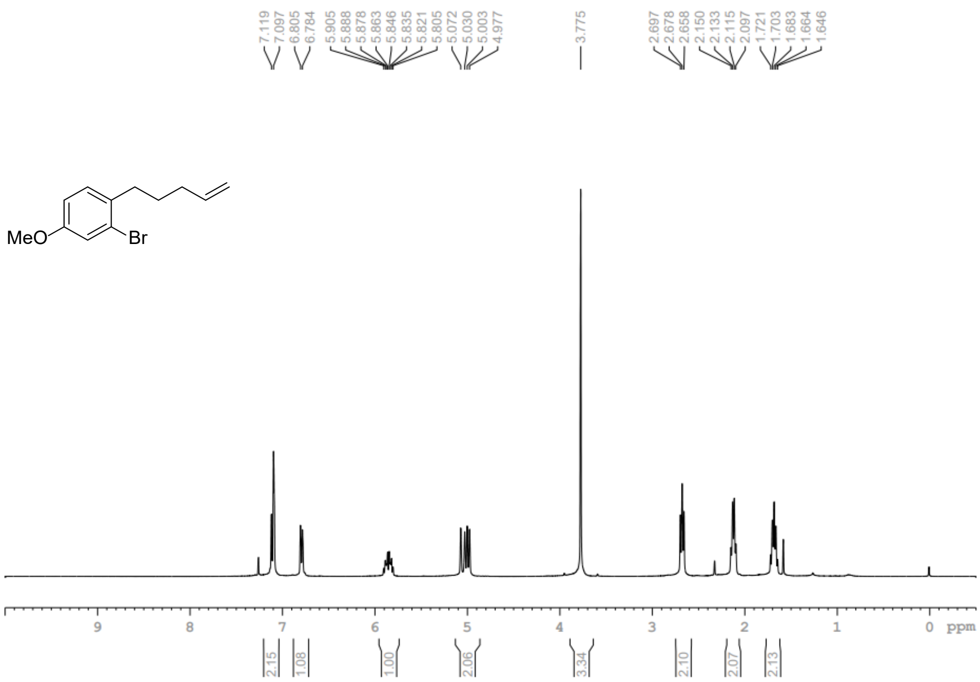

Supplementary Figure 93. ^{13}C NMR spectra of compound **S27** (100 MHz, $CDCl_3$)

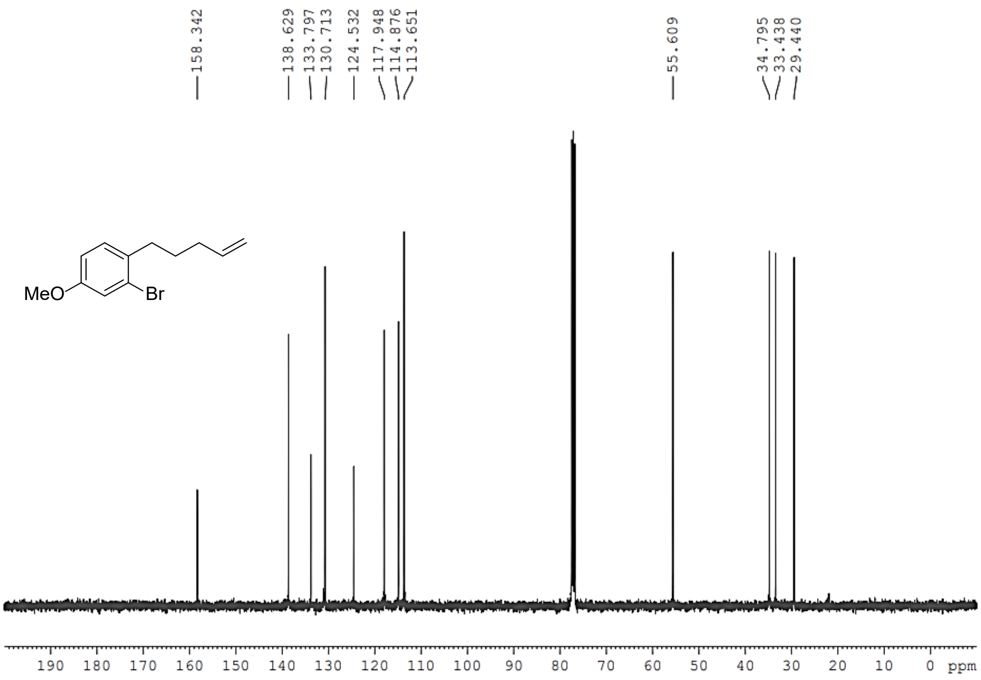

Supplementary Figure 94. ^{1}H NMR spectra of compound **3** (400 MHz, $CDCl_3$)

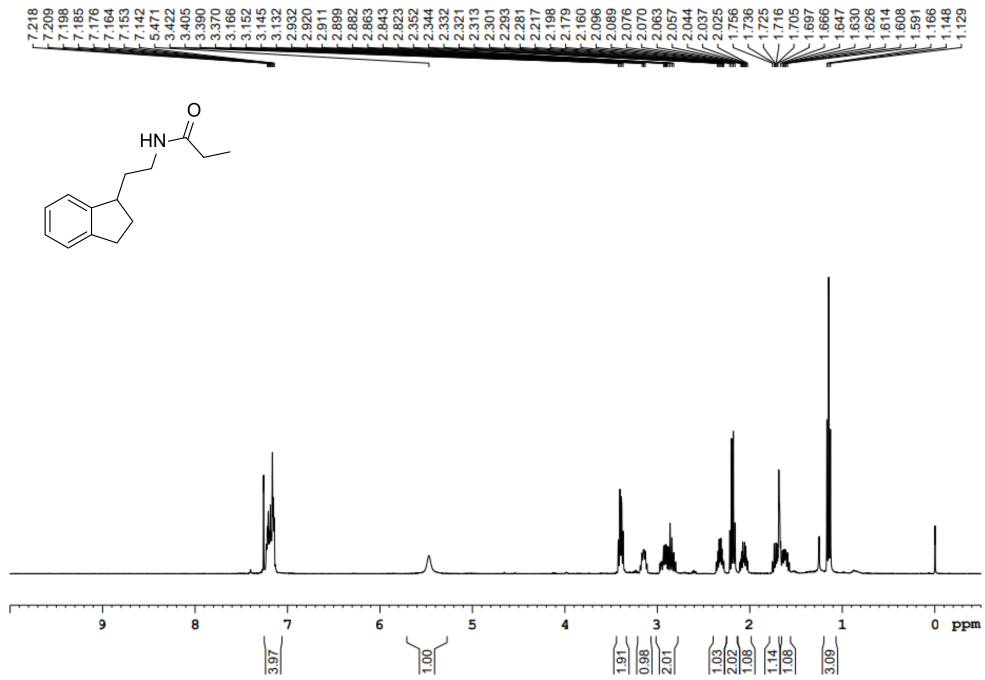

Supplementary Figure 95. ^{13}C NMR spectra of compound **3** (100 MHz, $CDCl_3$)

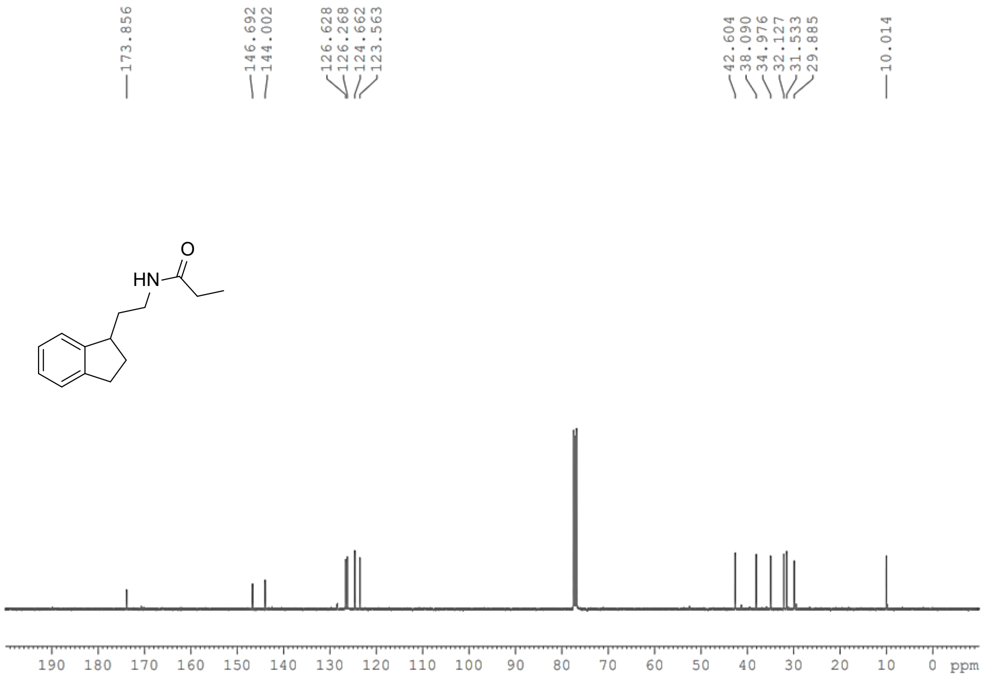

Supplementary Figure 96. ^{1}H NMR spectra of compound **4** (400 MHz, $CDCl_3$)

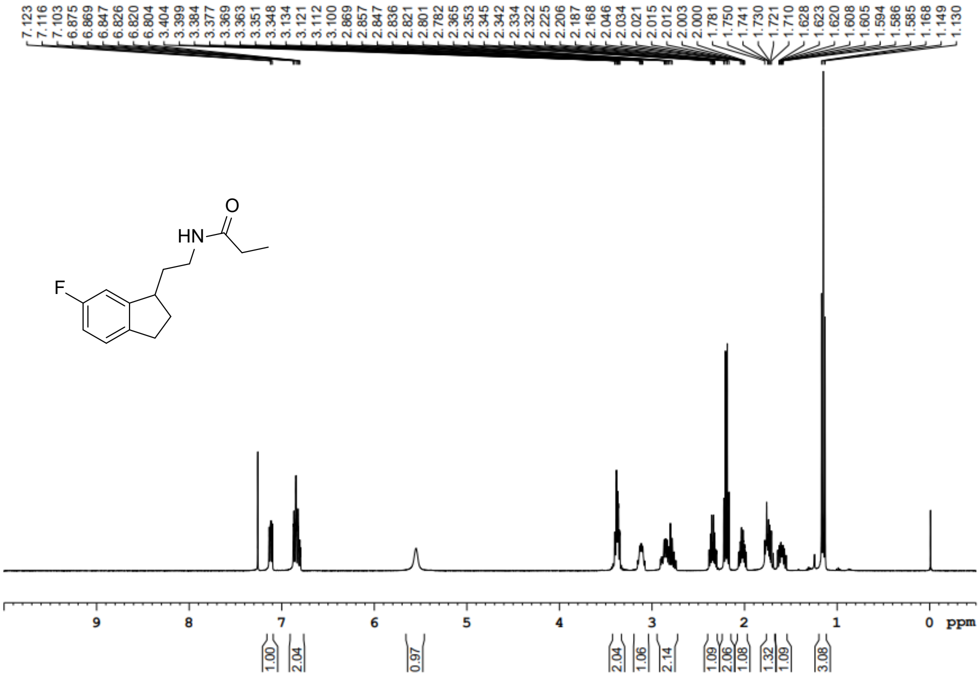

Supplementary Figure 97. ^{13}C NMR spectra of compound **4** (100 MHz, $CDCl_3$)

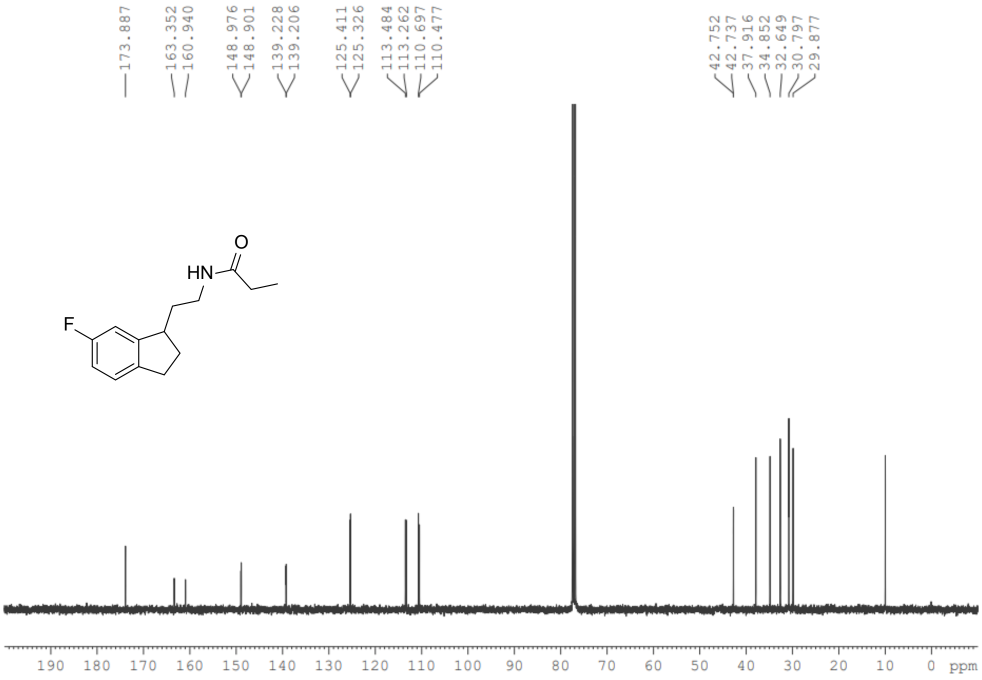

Supplementary Figure 98. ^{19}F NMR spectra of compound **4** (376 MHz, $CDCl_3$)

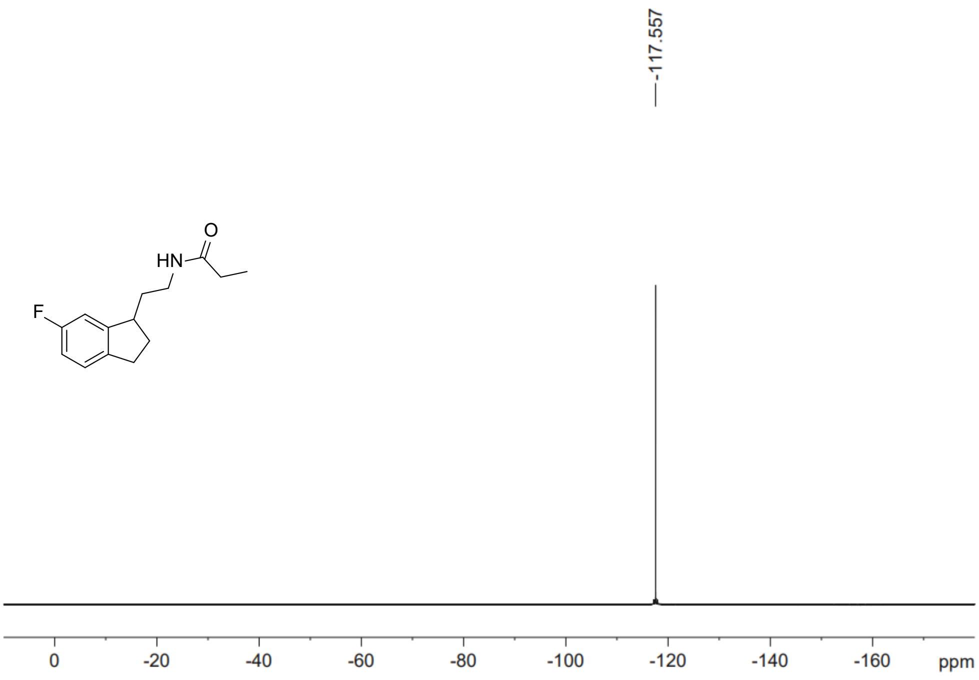

Supplementary Figure 99. ^{1}H NMR spectra of compound **5** (400 MHz, $CDCl_3$)

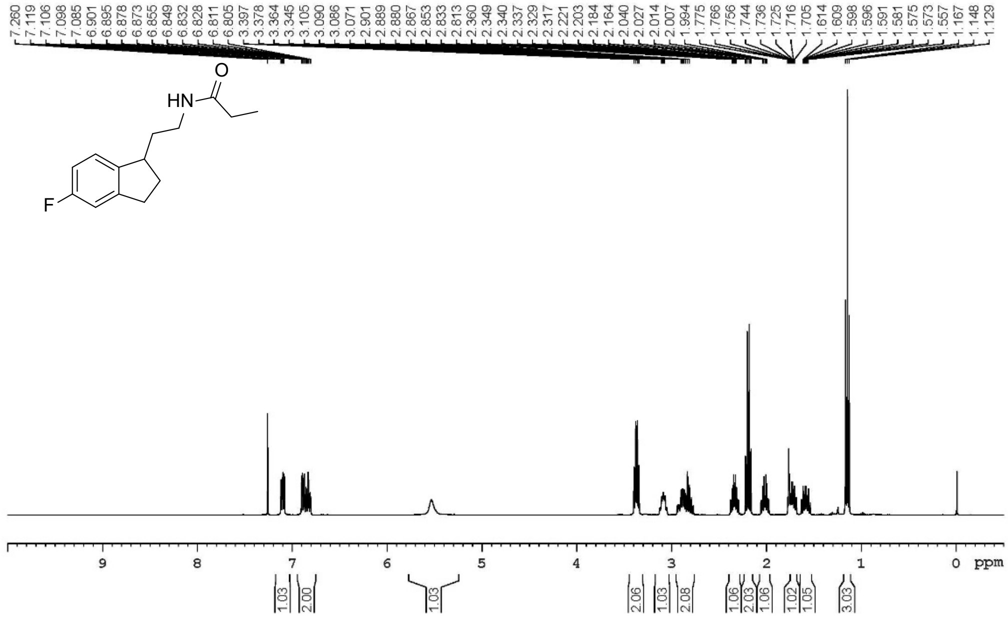

Supplementary Figure 100. ^{13}C NMR spectra of compound **5** (100 MHz, $CDCl_3$)

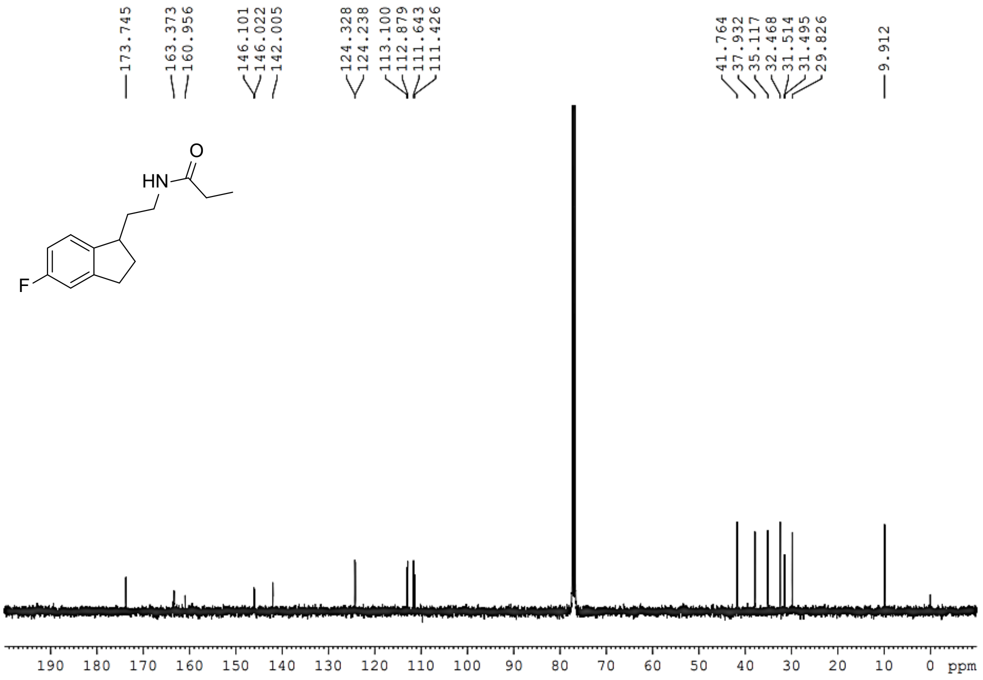

Supplementary Figure 101. ^{19}F NMR spectra of compound **5** (376 MHz, $CDCl_3$)

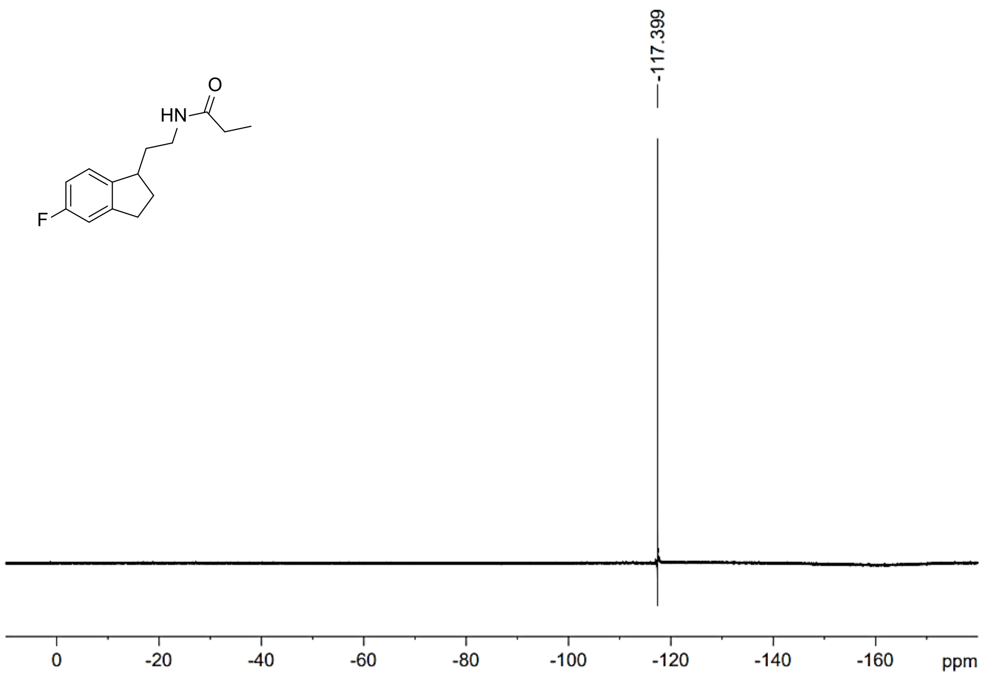

Supplementary Figure 102. ^{1}H NMR spectra of compound **6** (400 MHz, $CDCl_3$)

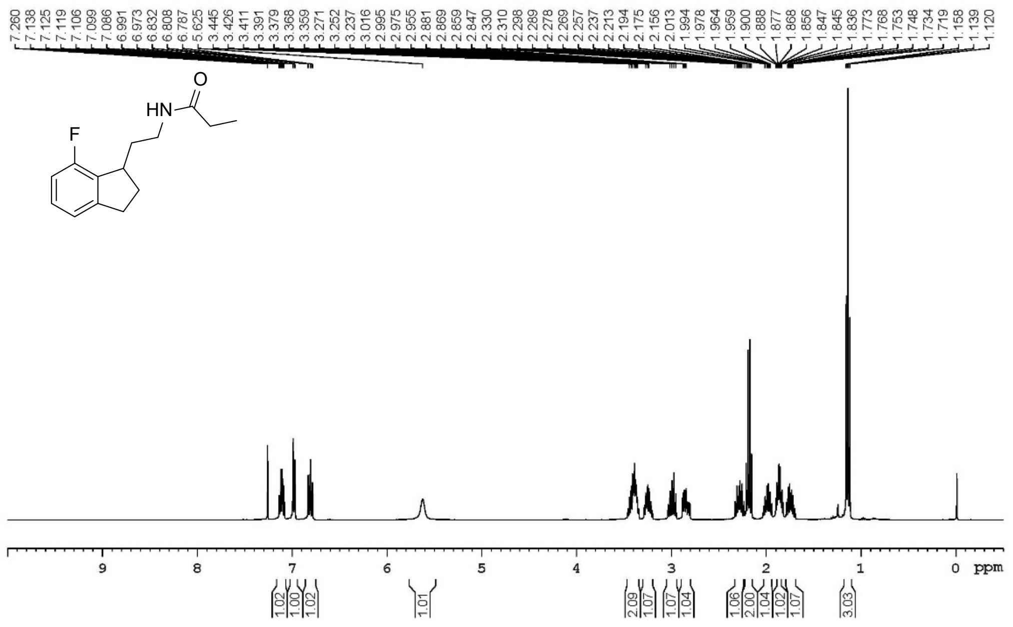

Supplementary Figure 103. ^{13}C NMR spectra of compound **6** (100 MHz, $CDCl_3$)

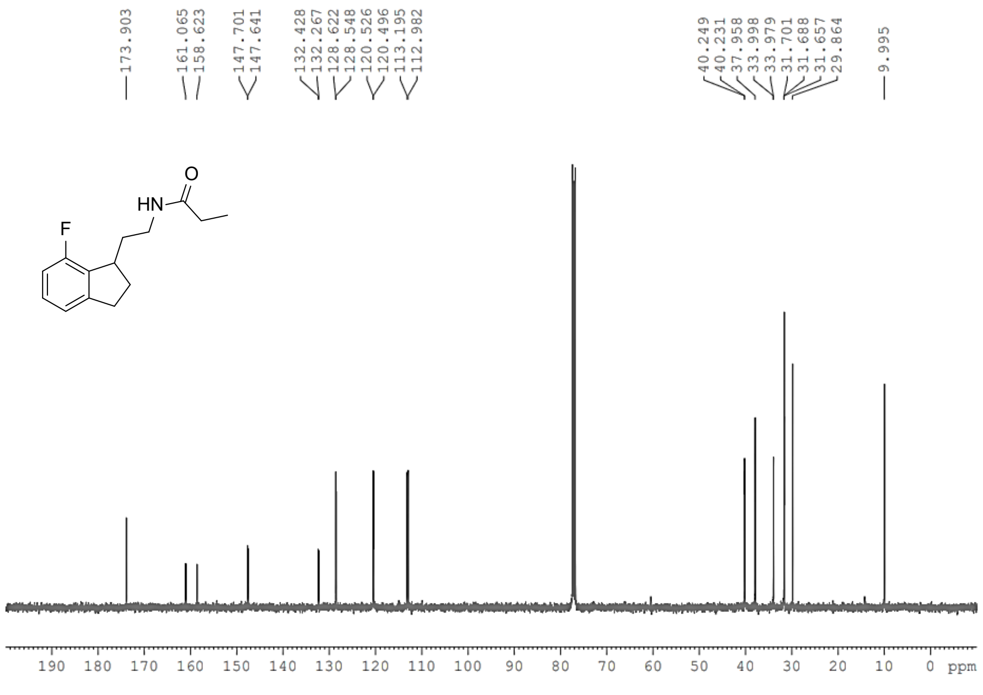

Supplementary Figure 104. ^{19}F NMR spectra of compound **6** (376 MHz, $CDCl_3$)

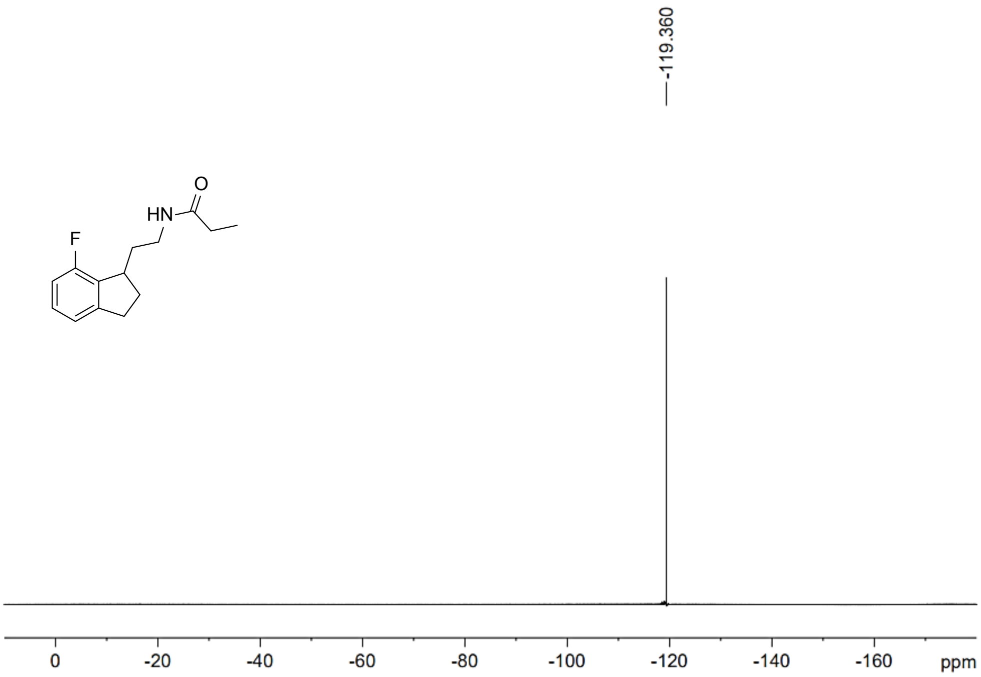

Supplementary Figure 105. ^{1}H NMR spectra of compound **7** (400 MHz, $CDCl_3$)

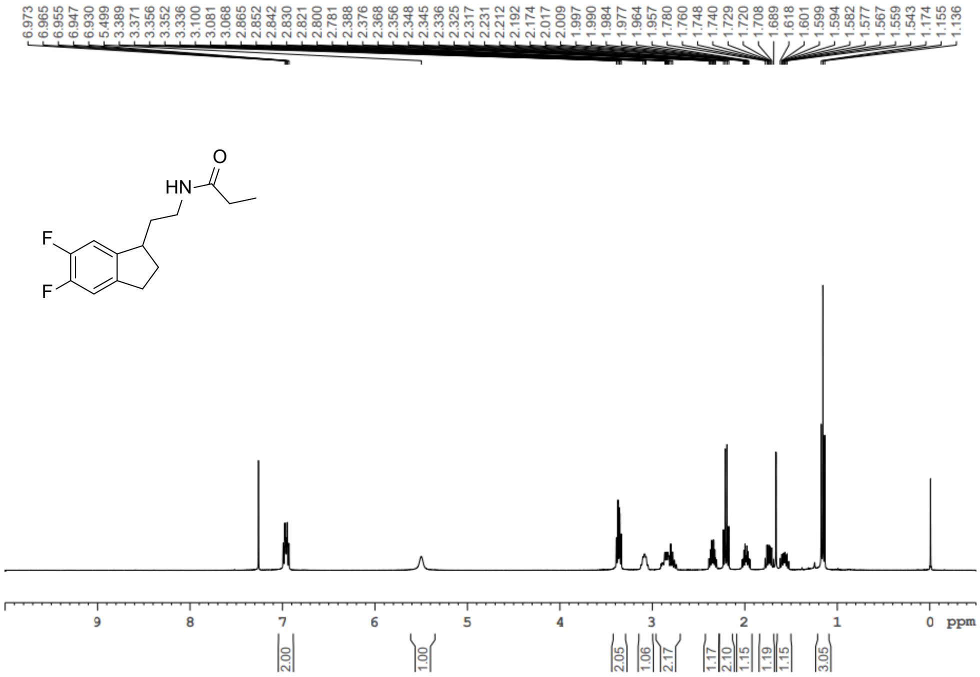

Supplementary Figure 106. ^{13}C NMR spectra of compound **7** (100 MHz, $CDCl_3$)

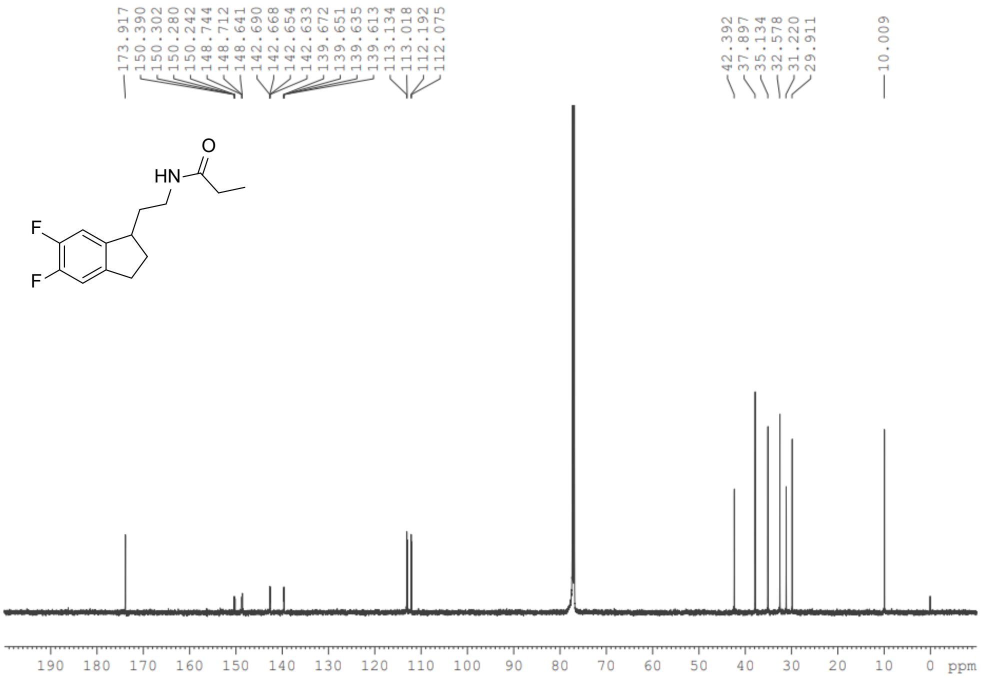

Supplementary Figure 107. ^{19}F NMR spectra of compound **7** (376 MHz, $CDCl_3$)

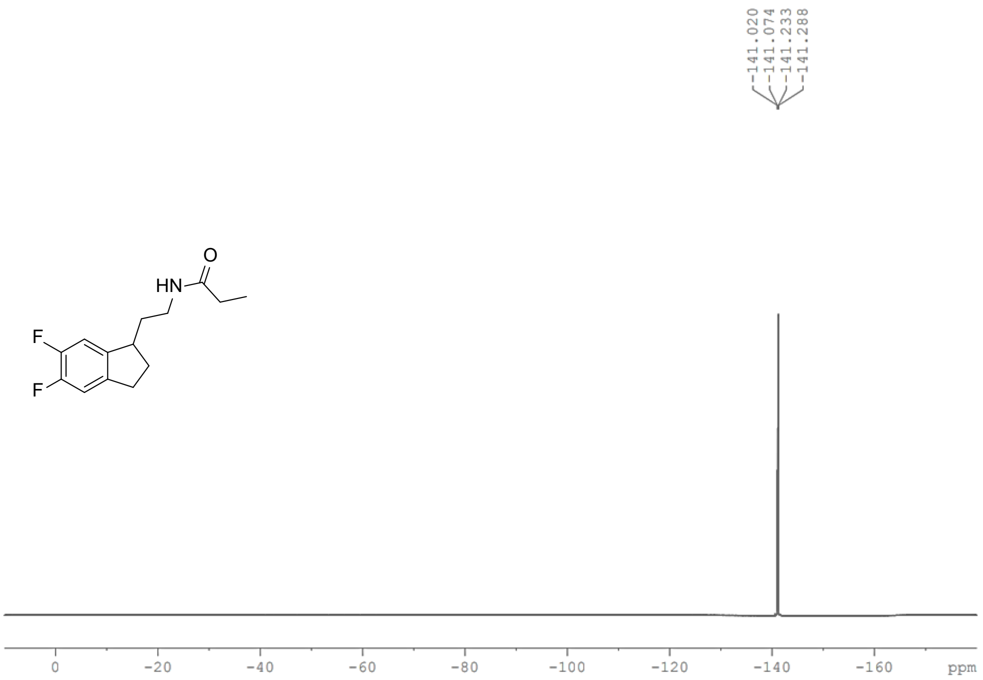

Supplementary Figure 108. ^{1}H NMR spectra of compound **8** (600 MHz, $CDCl_3$)

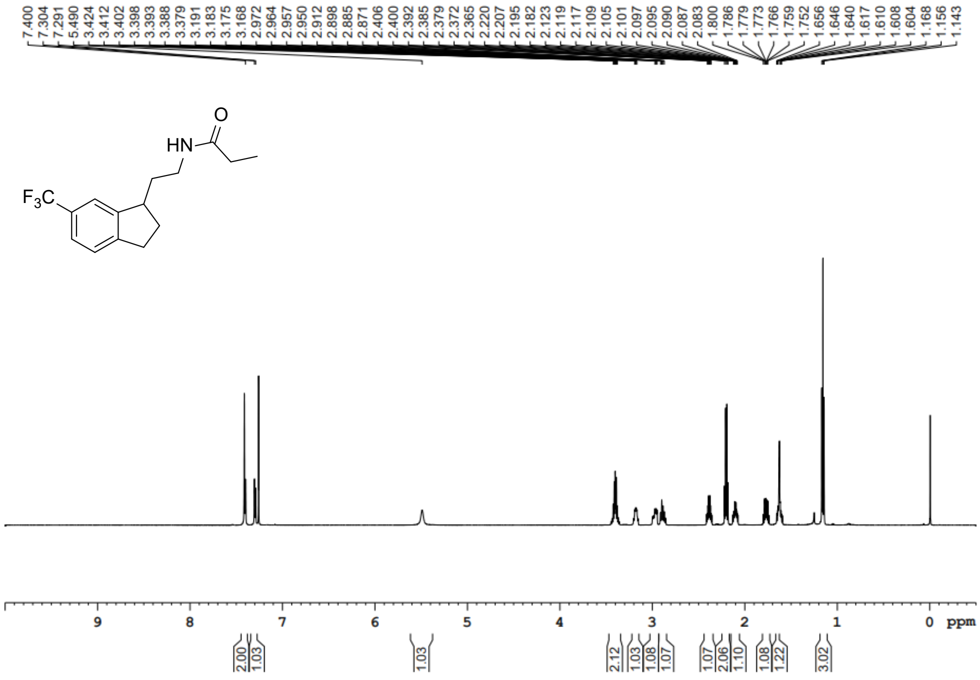

Supplementary Figure 109. ^{13}C NMR spectra of compound **8** (150 MHz, $CDCl_3$)

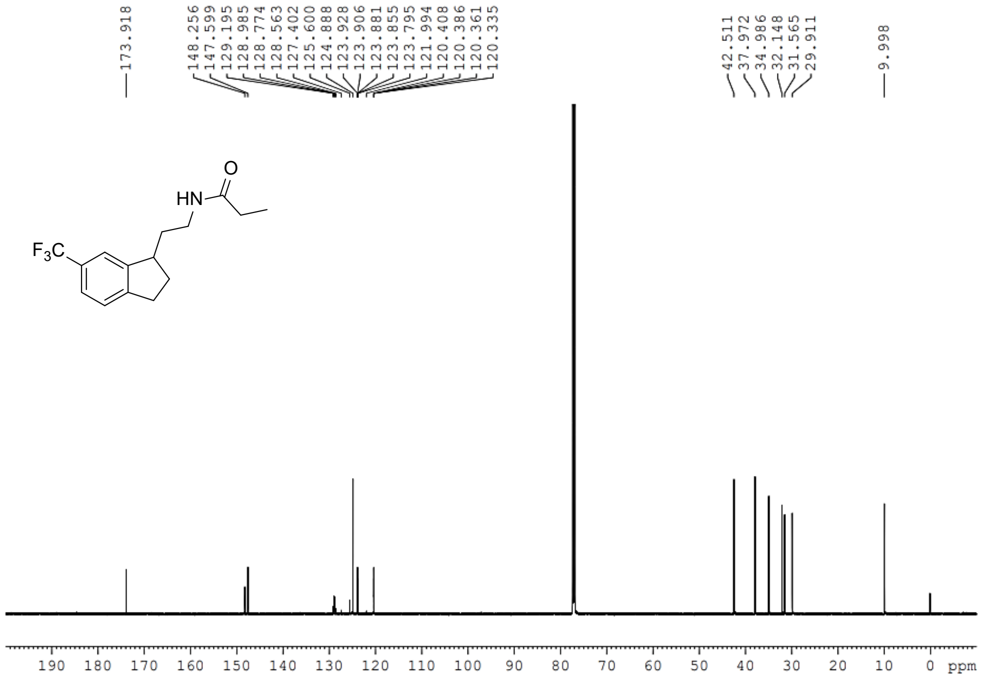

Supplementary Figure 110. ^{19}F NMR spectra of compound **8** (376 MHz, $CDCl_3$)

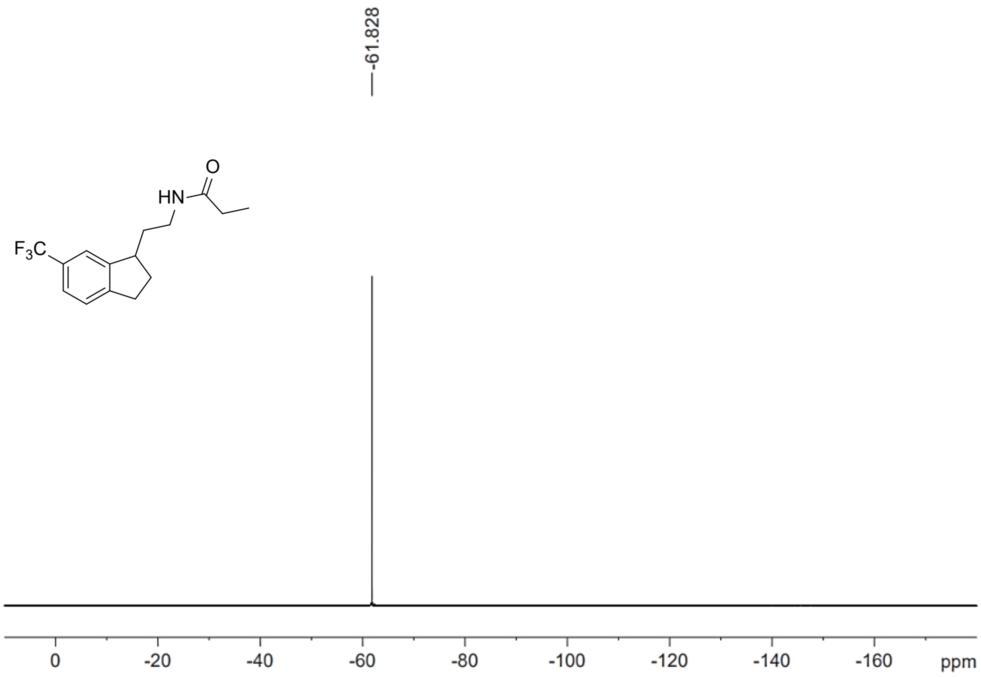

Supplementary Figure 111. ^{1}H NMR spectra of compound **9** (400 MHz, $CDCl_3$)

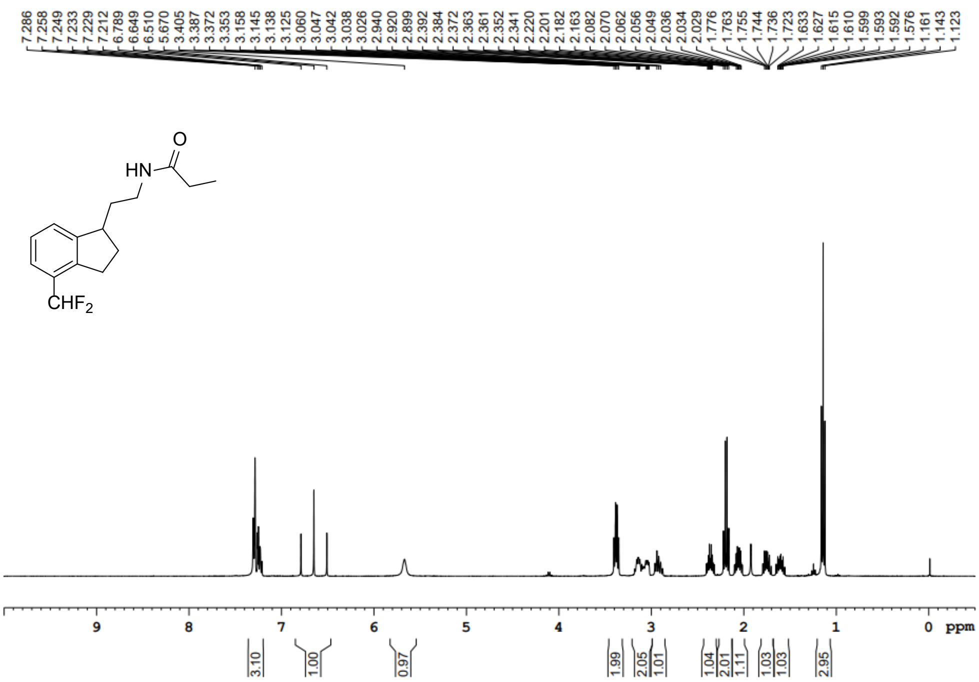

Supplementary Figure 112. ^{13}C NMR spectra of compound **9** (100 MHz, $CDCl_3$)

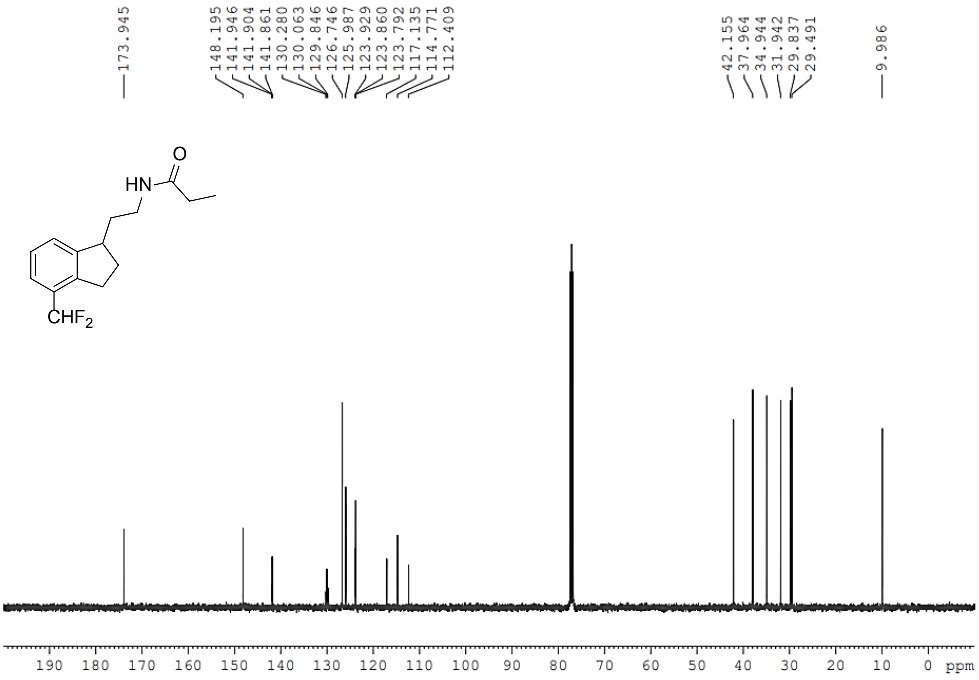

Supplementary Figure 113. ^{19}F NMR spectra of compound **9** (376 MHz, $CDCl_3$)

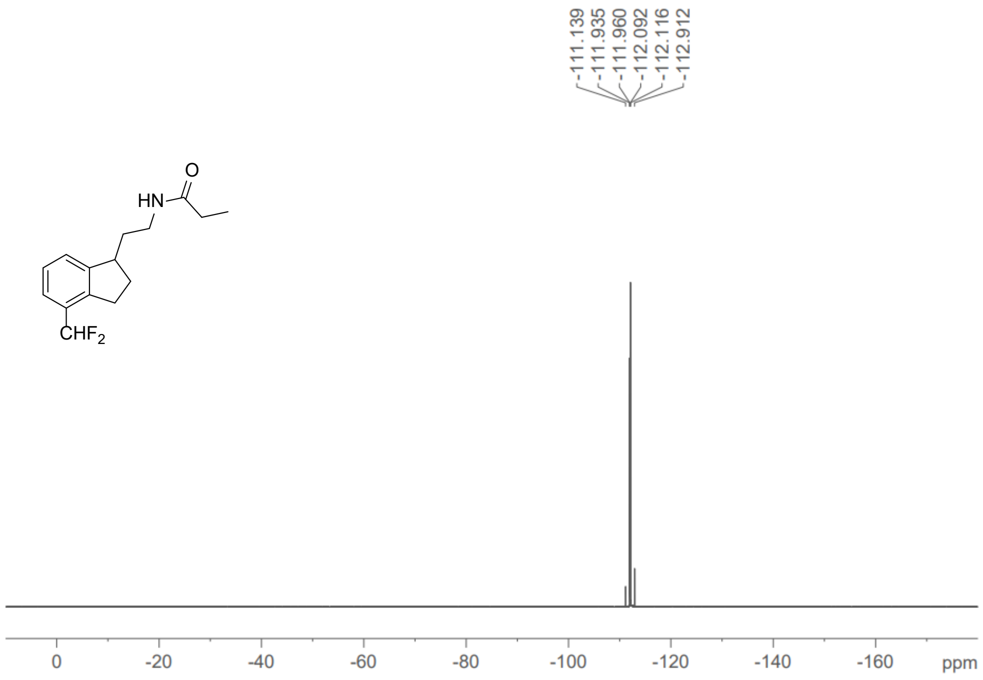

Supplementary Figure 114. ^{1}H NMR spectra of compound **10** (400 MHz, $CDCl_3$)

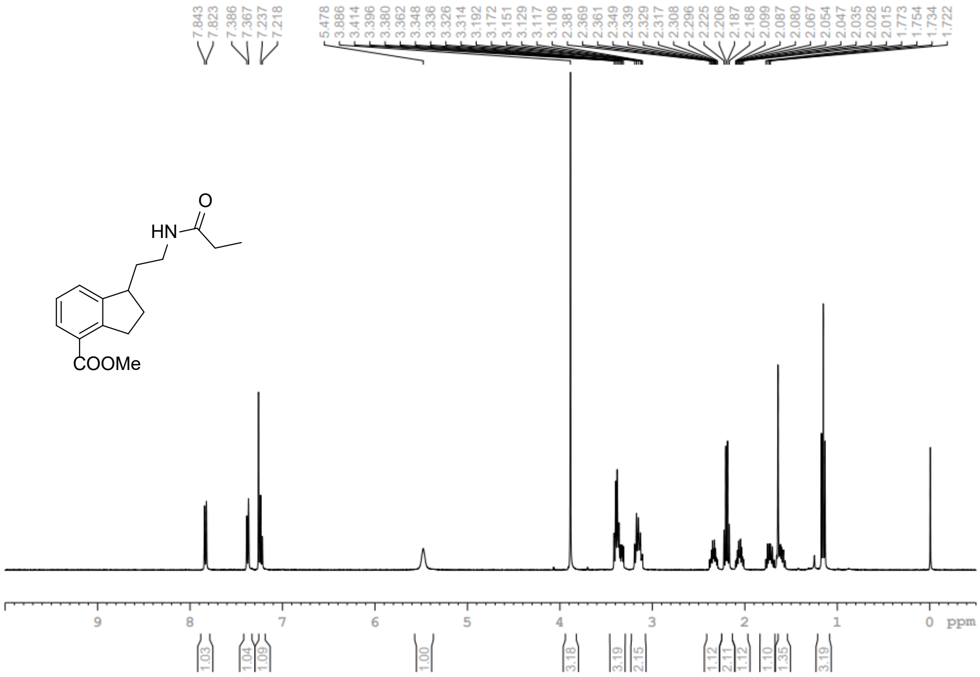

Supplementary Figure 115. ^{13}C NMR spectra of compound **10** (100 MHz, $CDCl_3$)

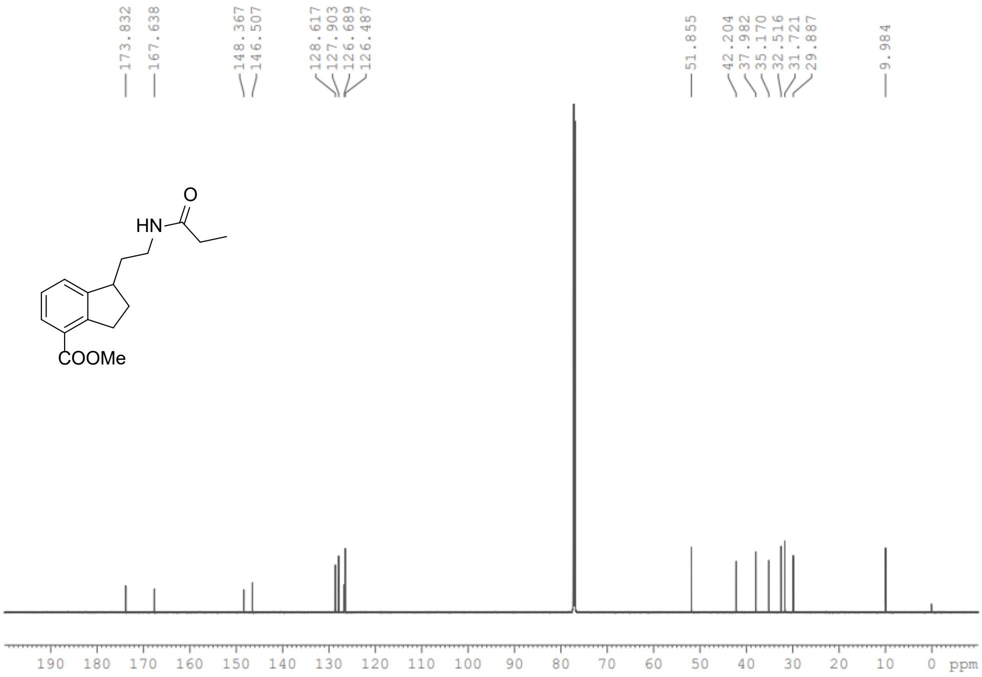

Supplementary Figure 116. ^{1}H NMR spectra of compound **11** (400 MHz, $CDCl_3$)

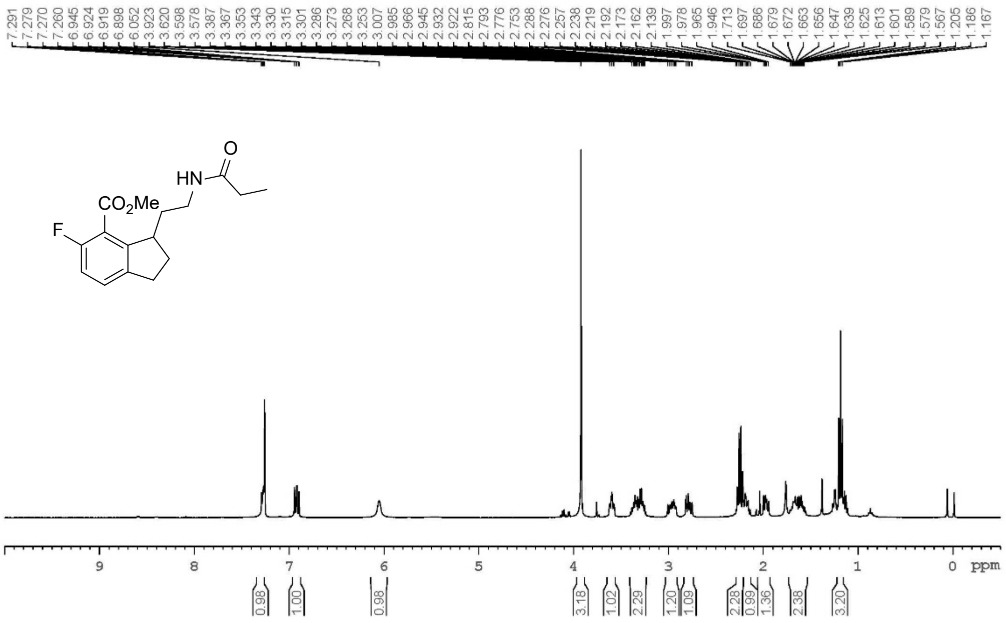

Supplementary Figure 117. ^{13}C NMR spectra of compound **11** (100 MHz, $CDCl_3$)

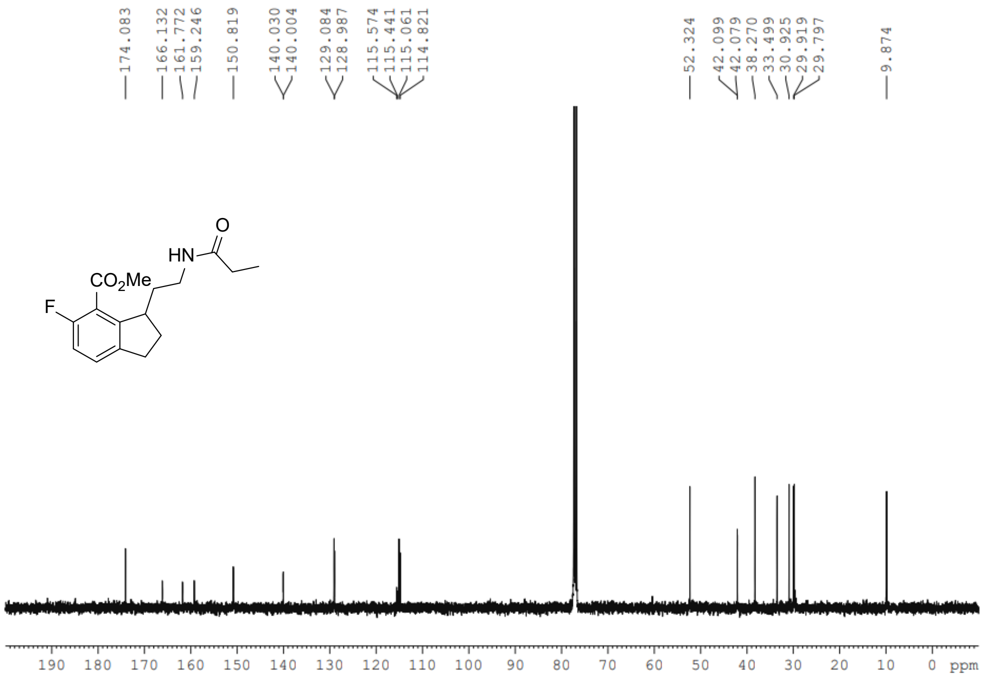

Supplementary Figure 118. ^{19}F NMR spectra of compound **11** (376 MHz, $CDCl_3$)

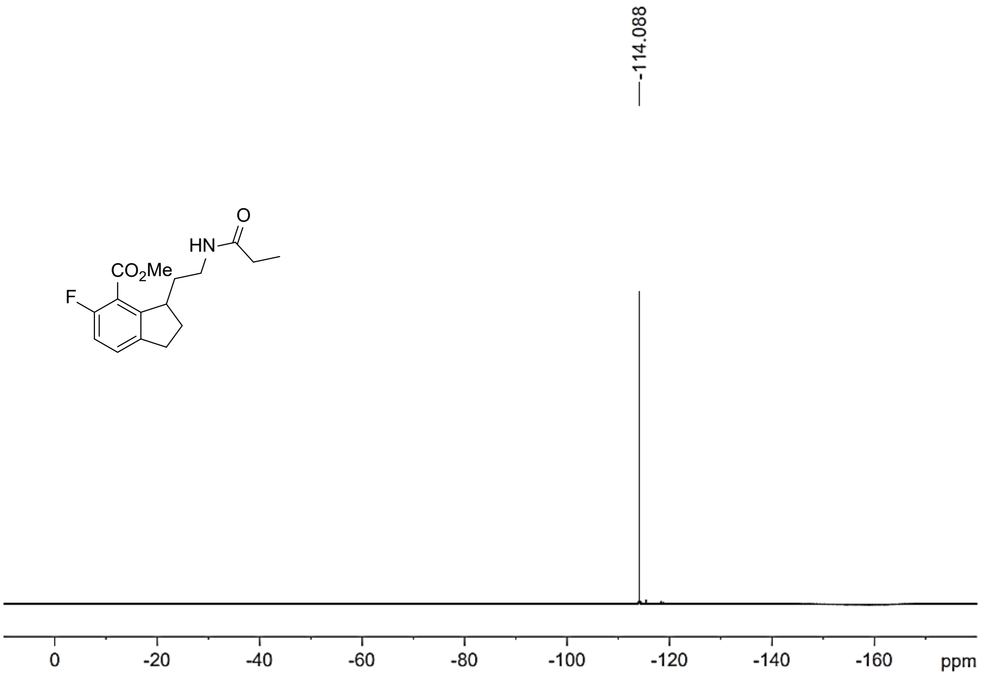

Supplementary Figure 119. ^{1}H NMR spectra of compound **12** (400 MHz, $CDCl_3$)

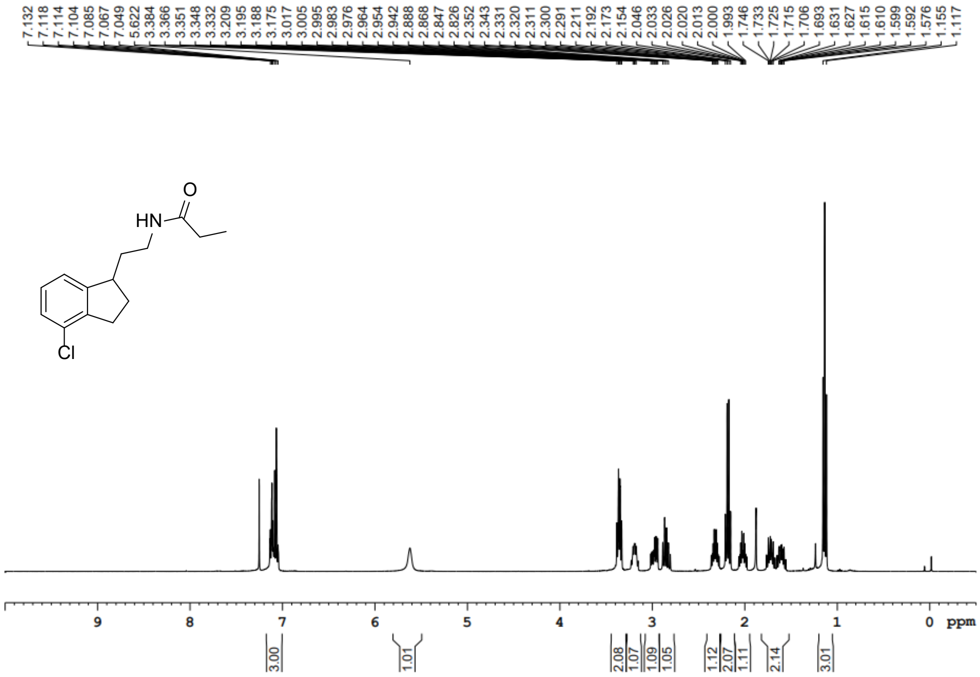

Supplementary Figure 120. ^{13}C NMR spectra of compound **12** (100 MHz, $CDCl_3$)

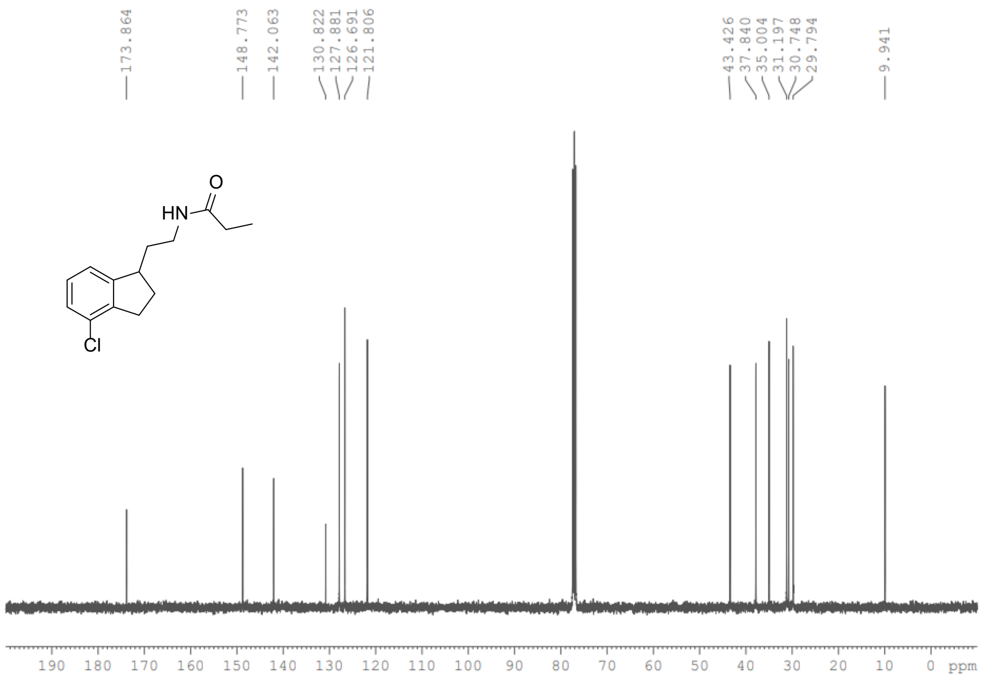

Supplementary Figure 121. ^{1}H NMR spectra of compound **13** (400 MHz, $CDCl_3$)

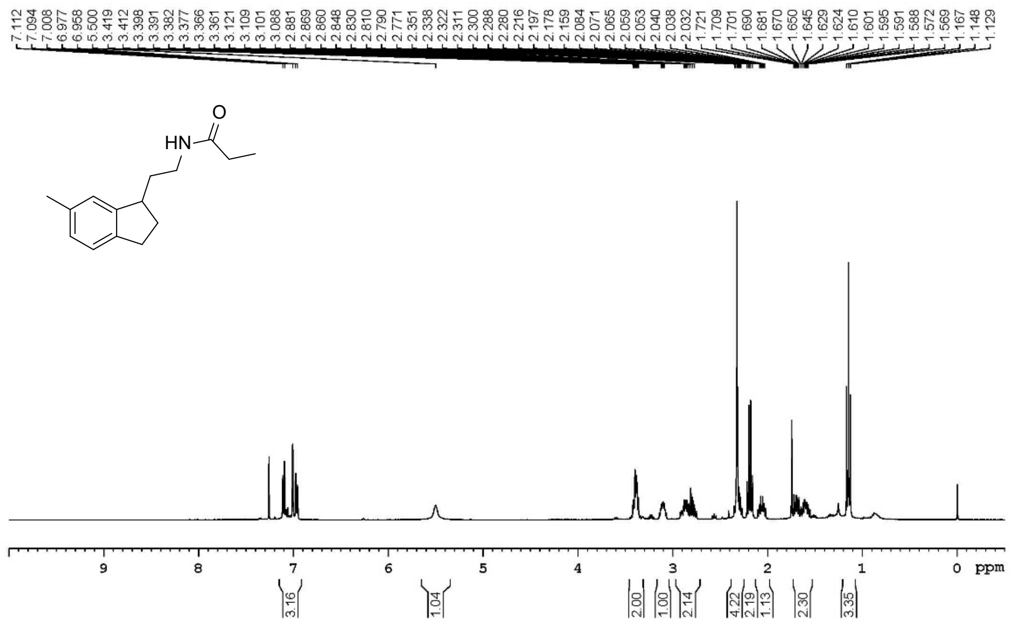

Supplementary Figure 122. ^{13}C NMR spectra of compound **13** (100 MHz, $CDCl_3$)

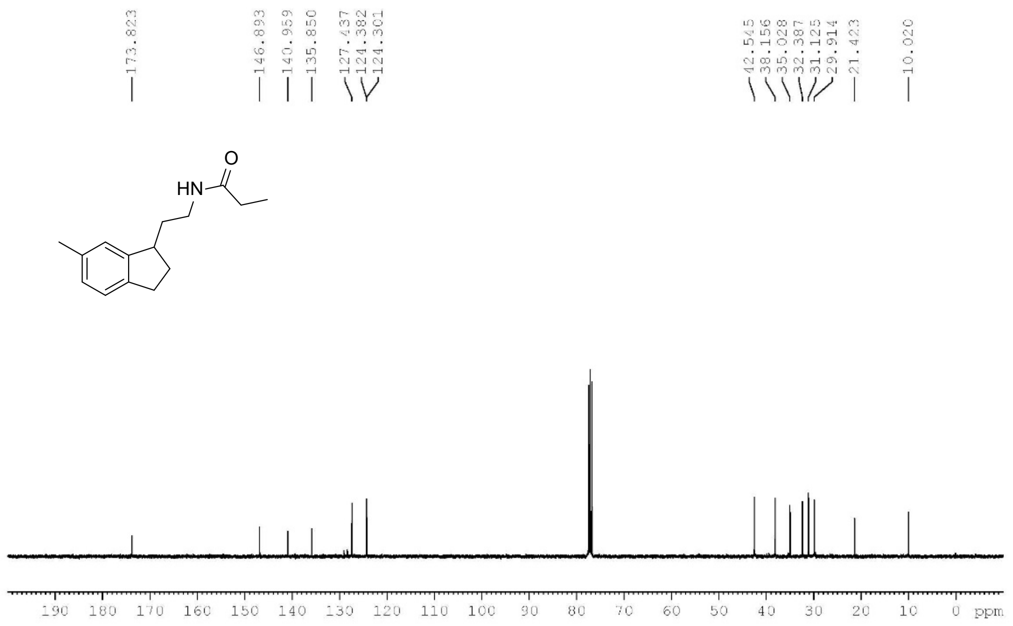

Supplementary Figure 123. ^{1}H NMR spectra of compound **14** (400 MHz, $CDCl_3$)

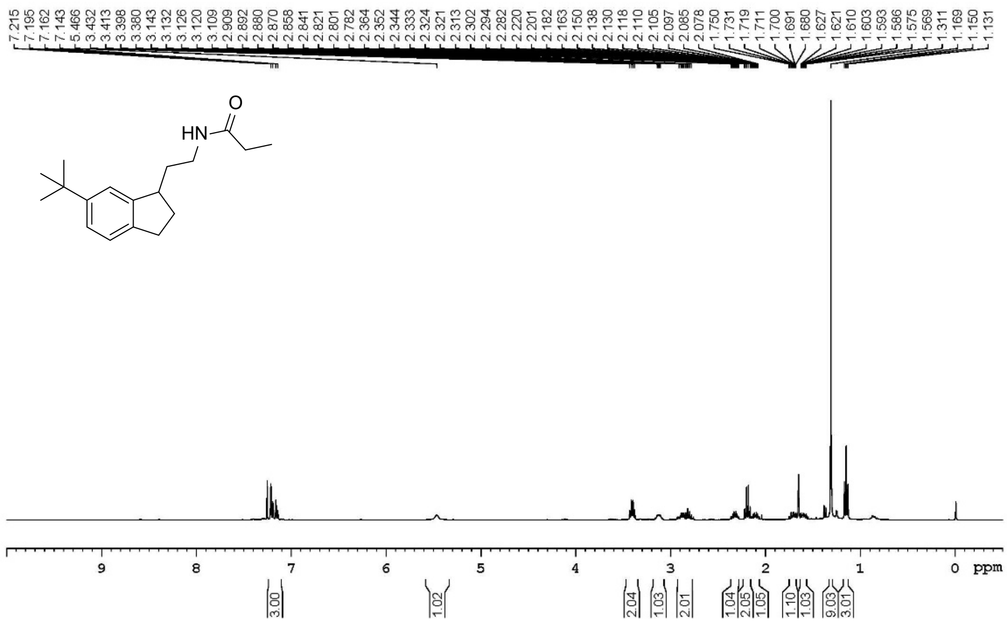

Supplementary Figure 124. ^{13}C NMR spectra of compound **14** (100 MHz, $CDCl_3$)

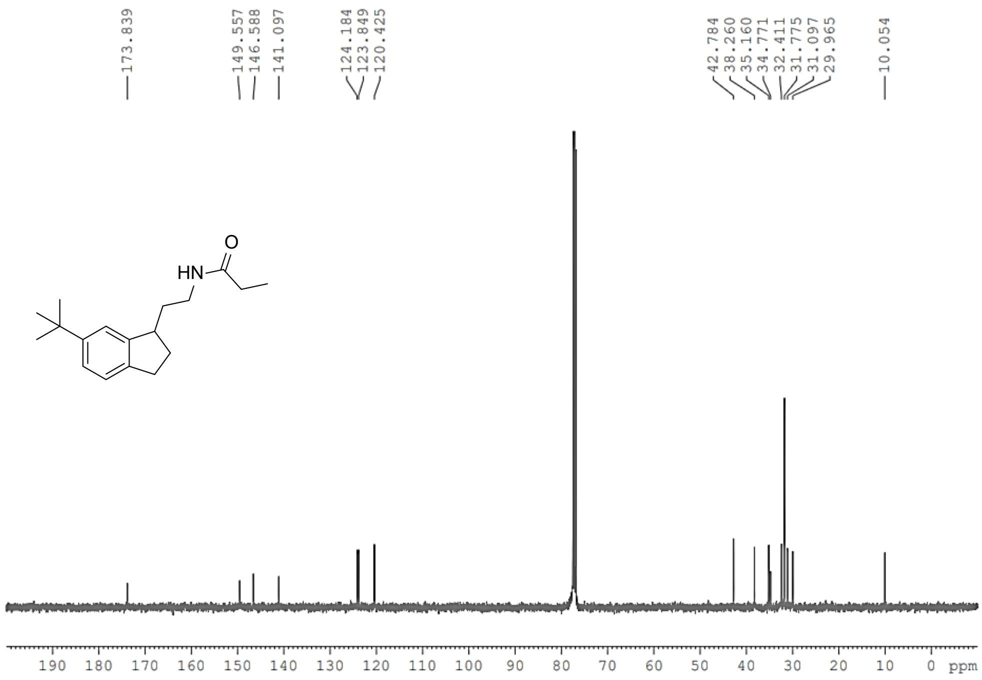

Supplementary Figure 125. ^{1}H NMR spectra of compound **15** (600 MHz, $CDCl_3$)

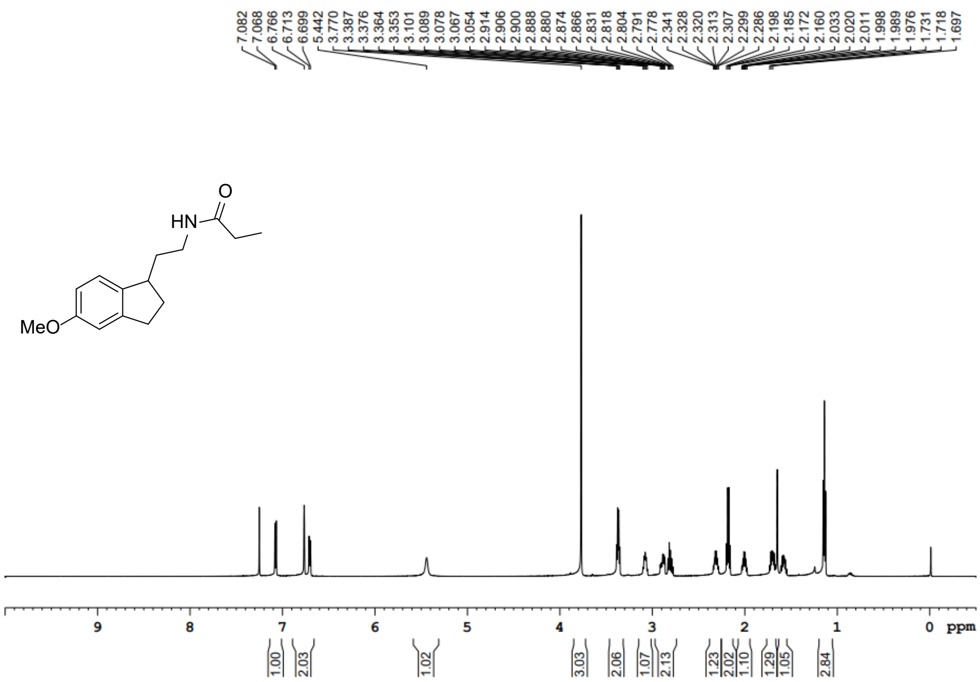

Supplementary Figure 126. ^{13}C NMR spectra of compound **15** (150 MHz, $CDCl_3$)

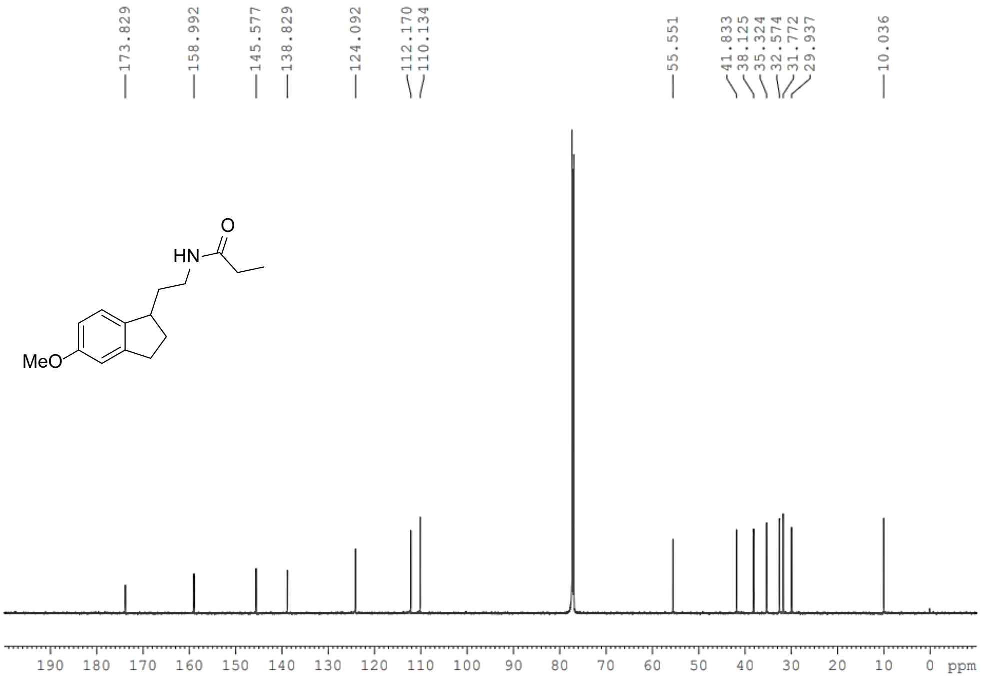

Supplementary Figure 127. ^{1}H NMR spectra of compound **16** (400 MHz, $CDCl_3$)

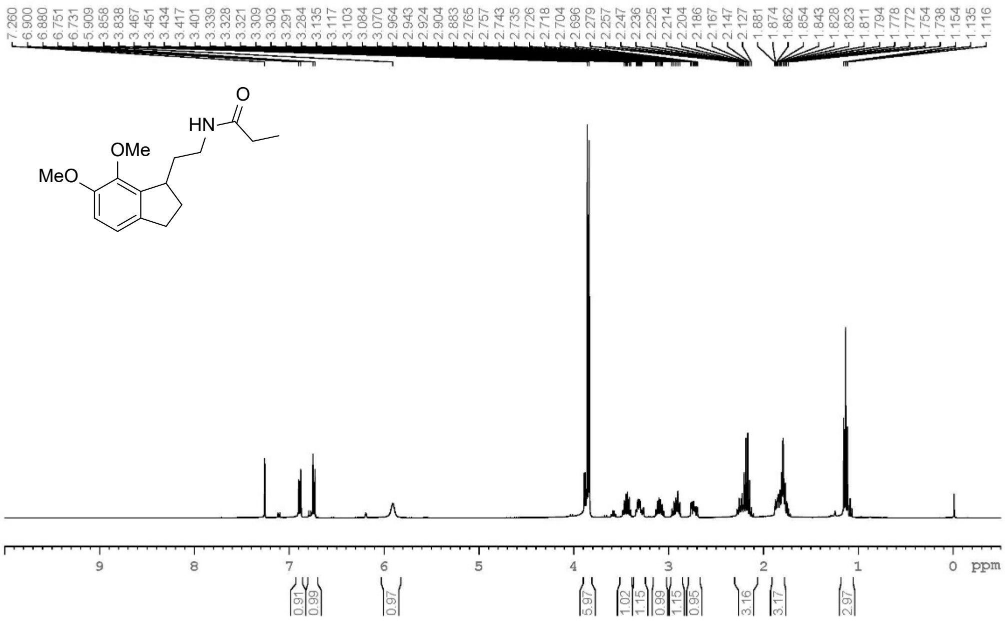

Supplementary Figure 128. ^{13}C NMR spectra of compound **16** (100 MHz, $CDCl_3$)

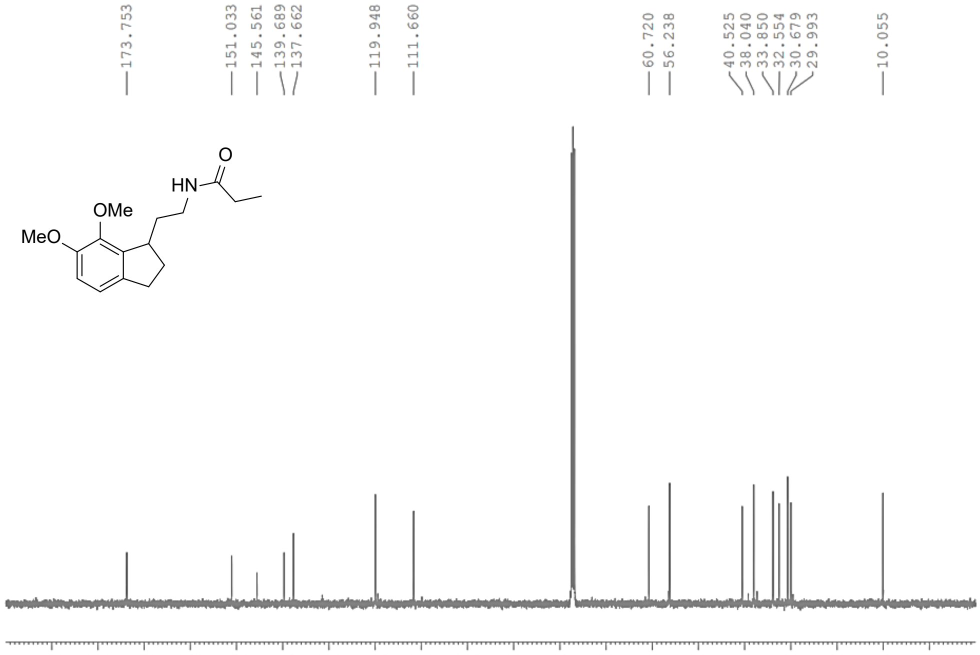

Supplementary Figure 129. ^{1}H NMR spectra of compound **17** (400 MHz, $CDCl_3$)

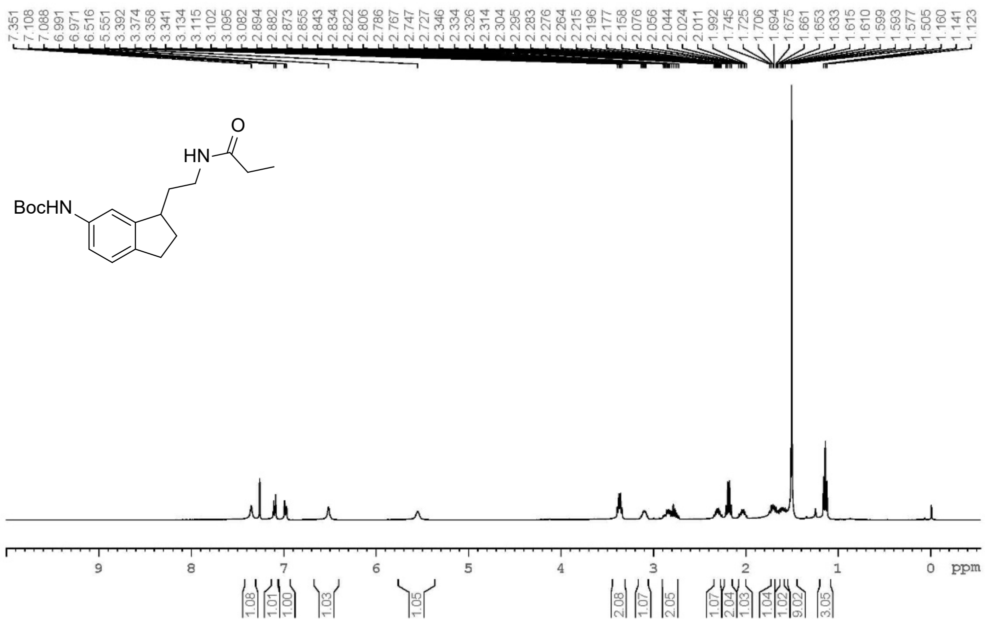

Supplementary Figure 130. ^{13}C NMR spectra of compound **17** (100 MHz, $CDCl_3$)

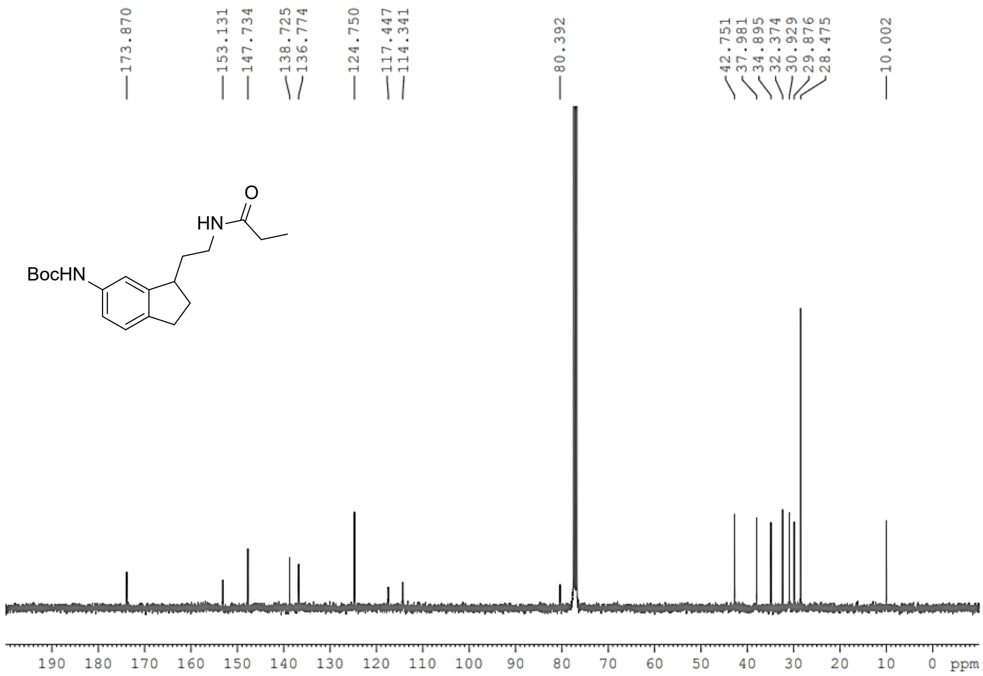

Supplementary Figure 131. ^{1}H NMR spectra of compound **18** (400 MHz, $CDCl_3$)

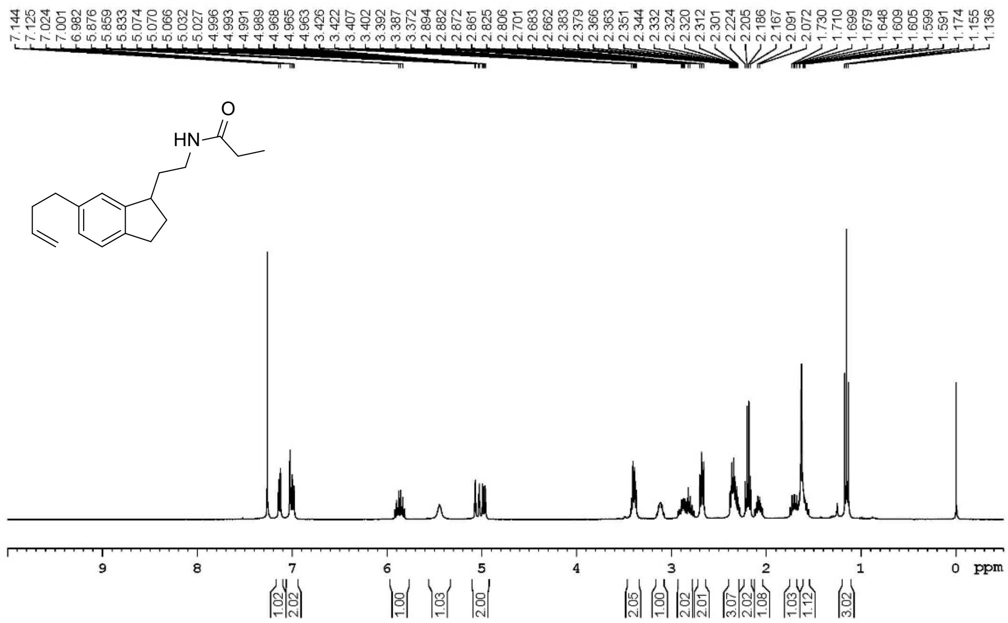

Supplementary Figure 132. ^{13}C NMR spectra of compound **18** (100 MHz, $CDCl_3$)

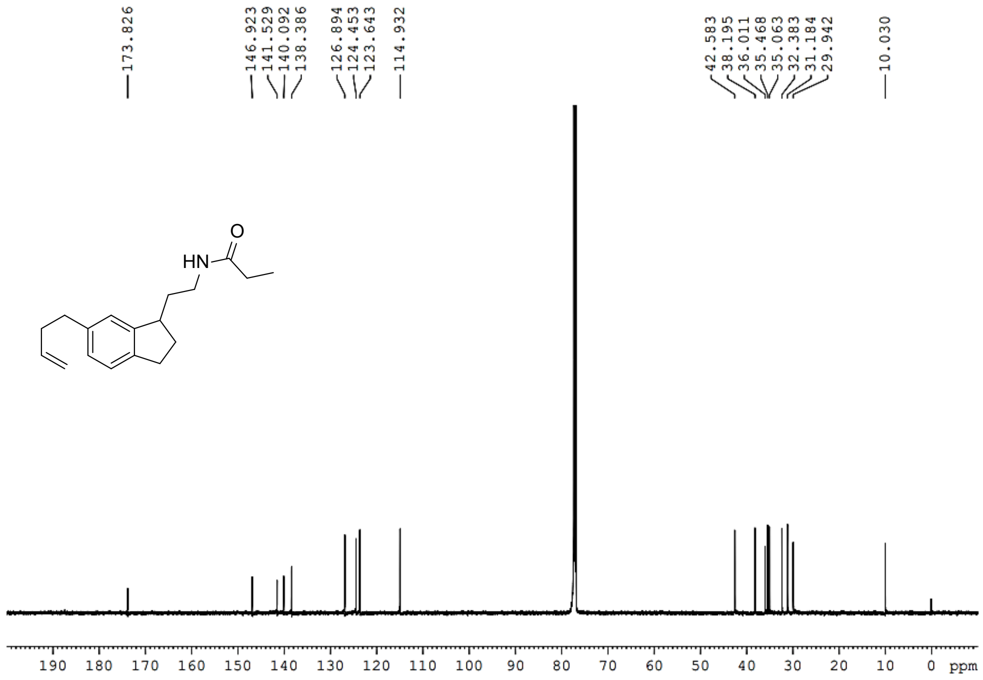

Supplementary Figure 133. ^{1}H NMR spectra of compound **19** (400 MHz, $CDCl_3$)

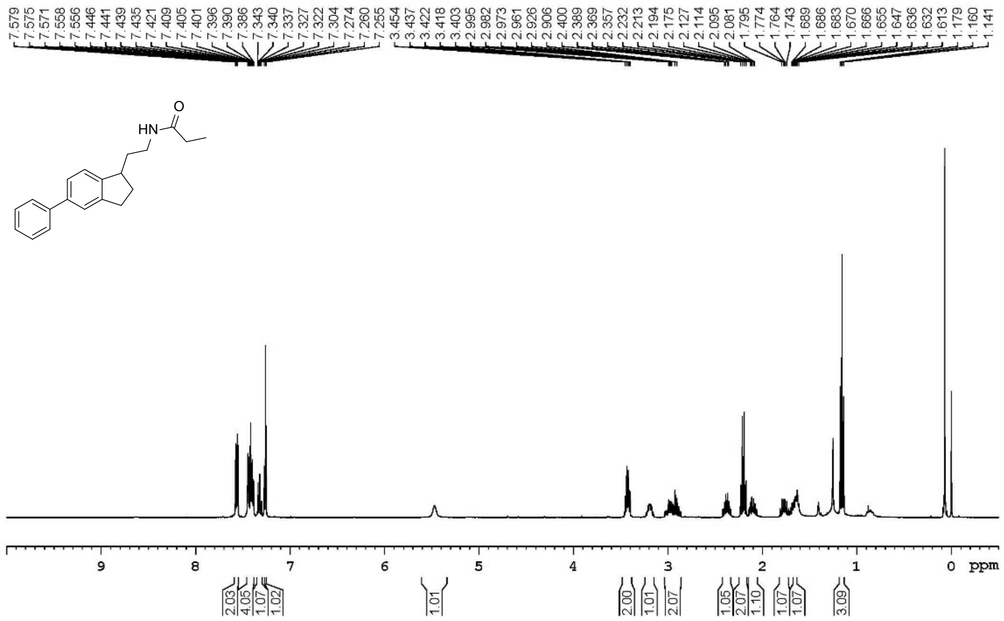

Supplementary Figure 134. ^{13}C NMR spectra of compound **19** (100 MHz, $CDCl_3$)

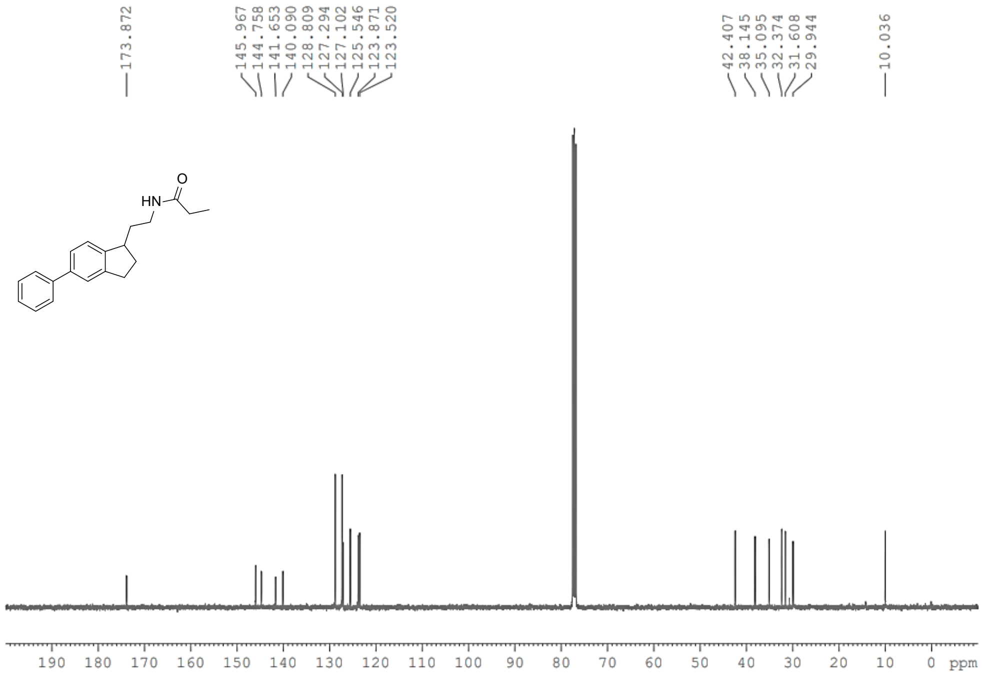

Supplementary Figure 135. ^{1}H NMR spectra of compound **20** (400 MHz, $CDCl_3$)

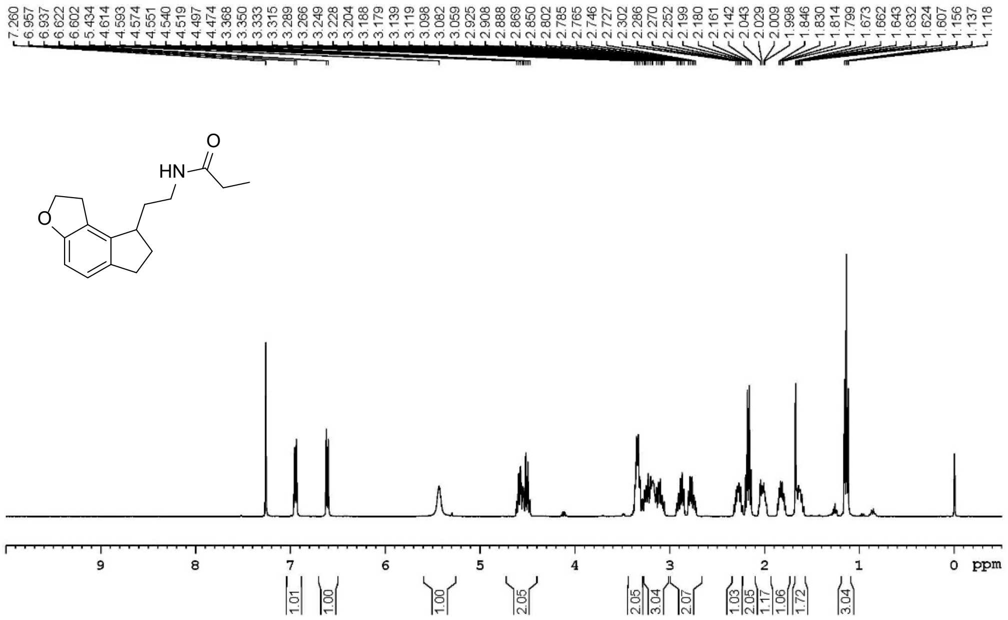

Supplementary Figure 136. ^{13}C NMR spectra of compound **20** (100 MHz, $CDCl_3$)

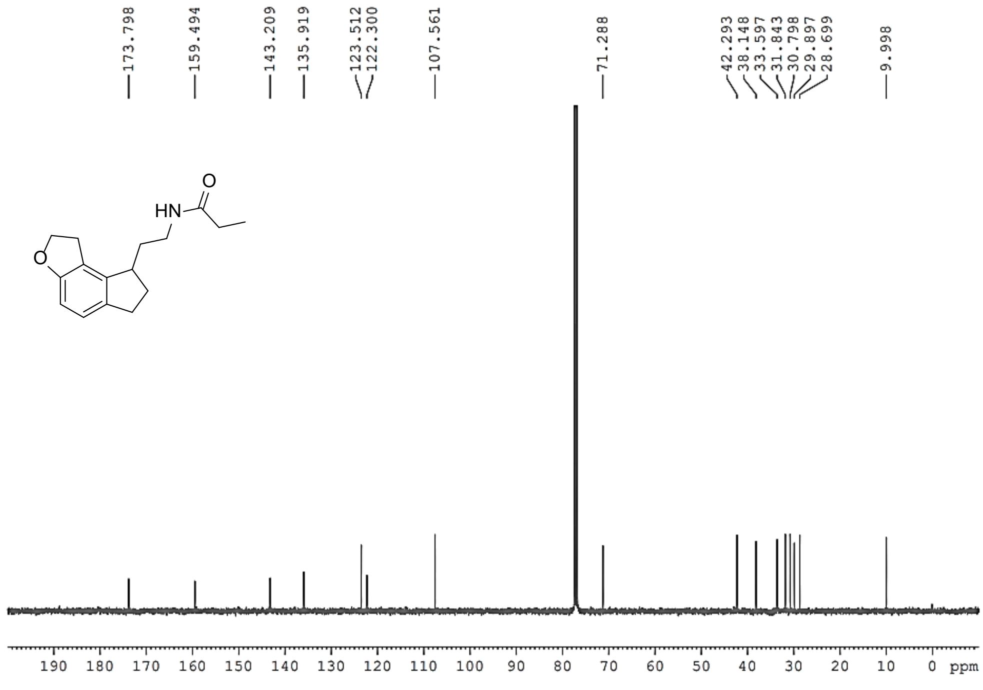

Supplementary Figure 137. ^{1}H NMR spectra of compound **21** (600 MHz, $CDCl_3$)

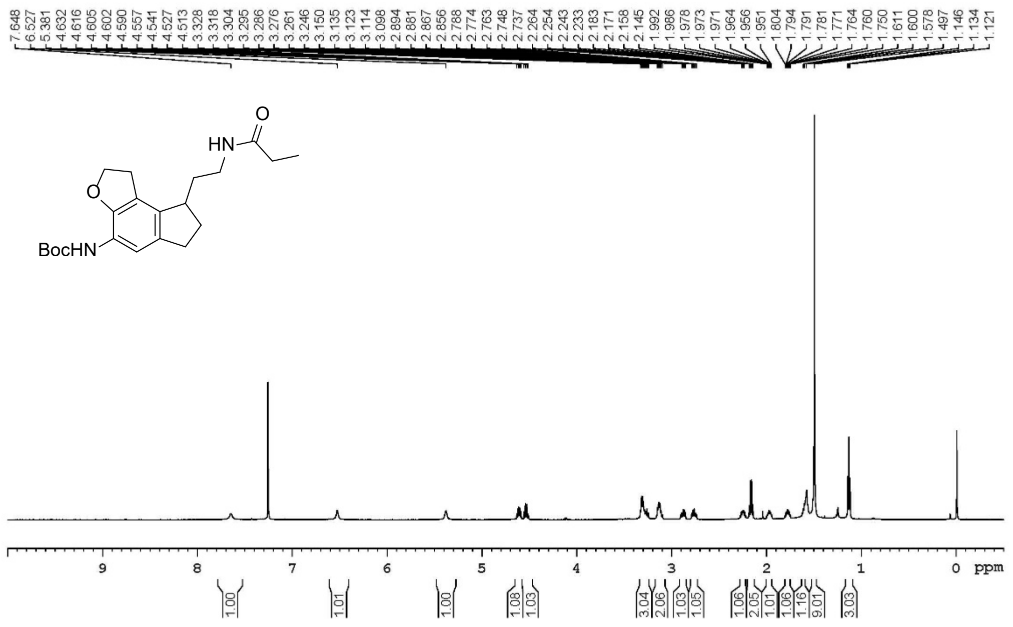

Supplementary Figure 138. ^{13}C NMR spectra of compound **21** (150 MHz, $CDCl_3$)

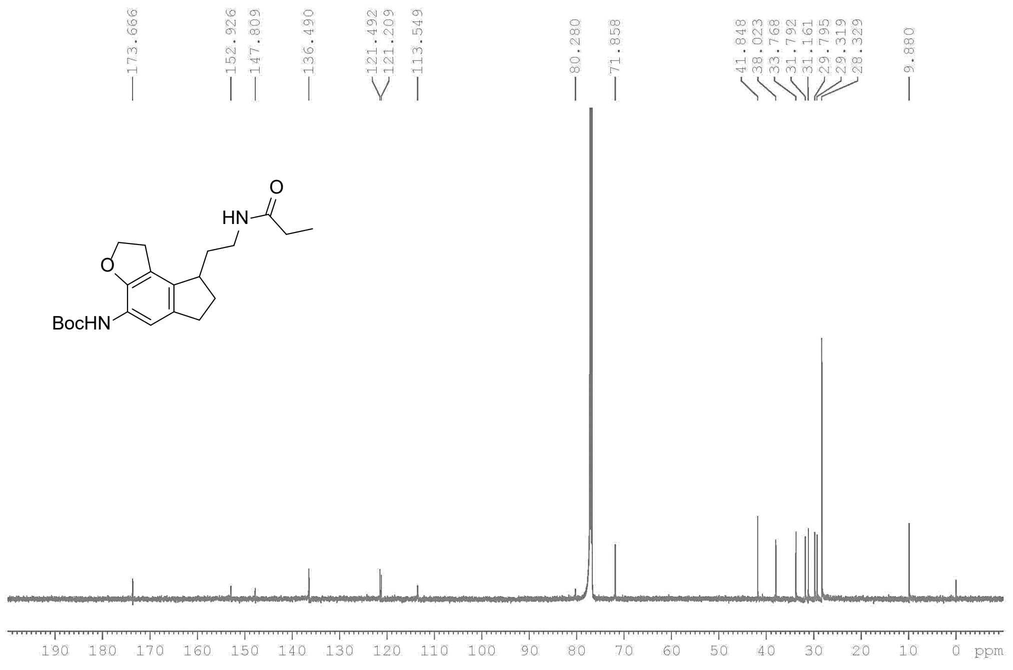

Supplementary Figure 139. ^{1}H NMR spectra of compound **22** (400 MHz, $CDCl_3$)

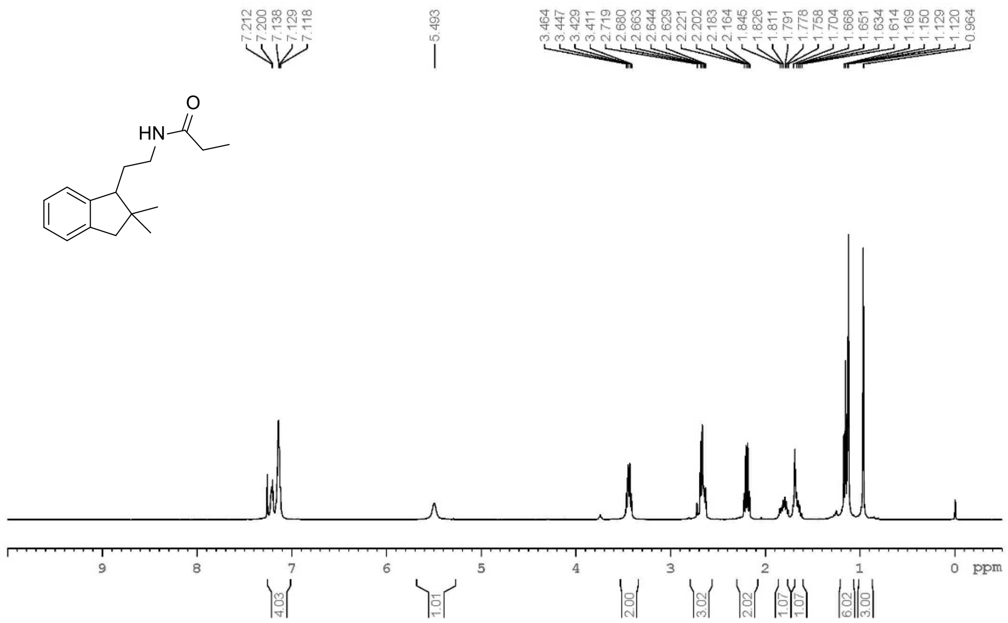

Supplementary Figure 140. ^{13}C NMR spectra of compound **22** (100 MHz, $CDCl_3$)

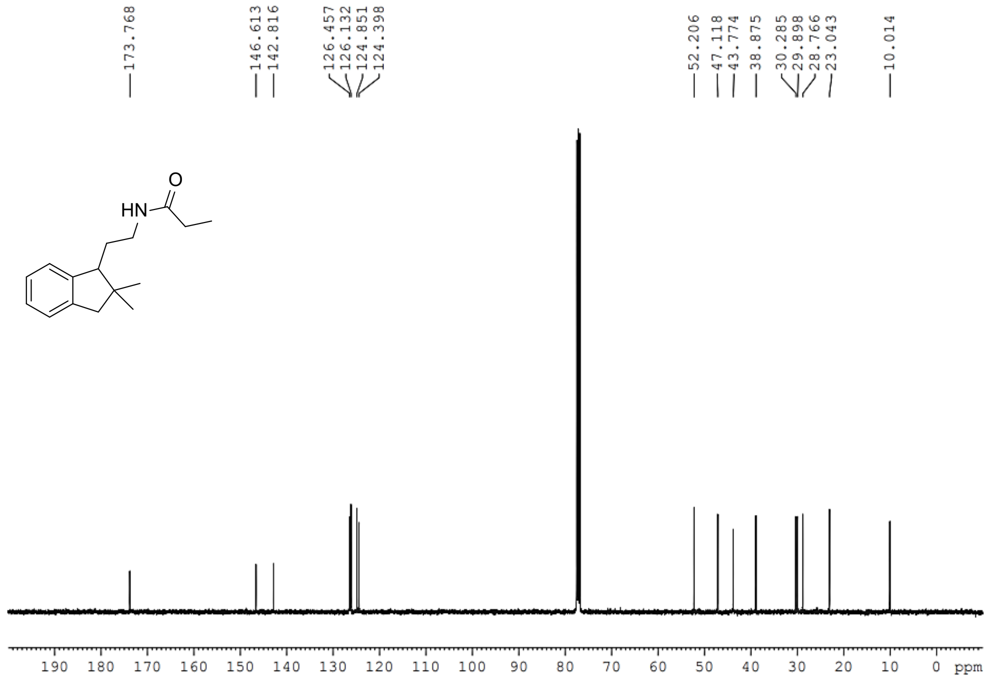

Supplementary Figure 141. ^{1}H NMR spectra of compound **23** (400 MHz, $CDCl_3$)

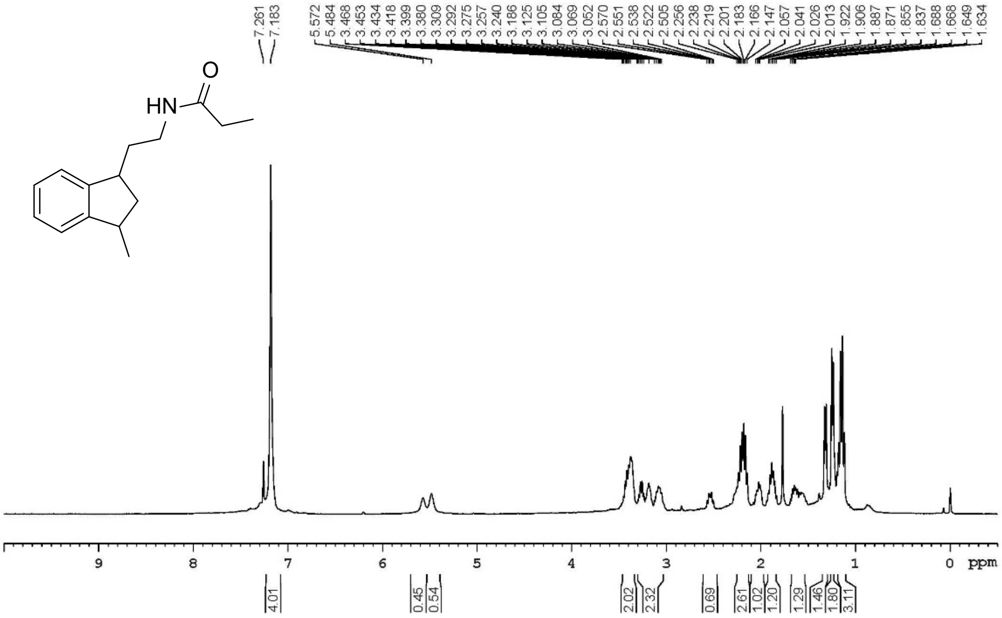

Supplementary Figure 142. ^{13}C NMR spectra of compound **23** (100 MHz, $CDCl_3$)

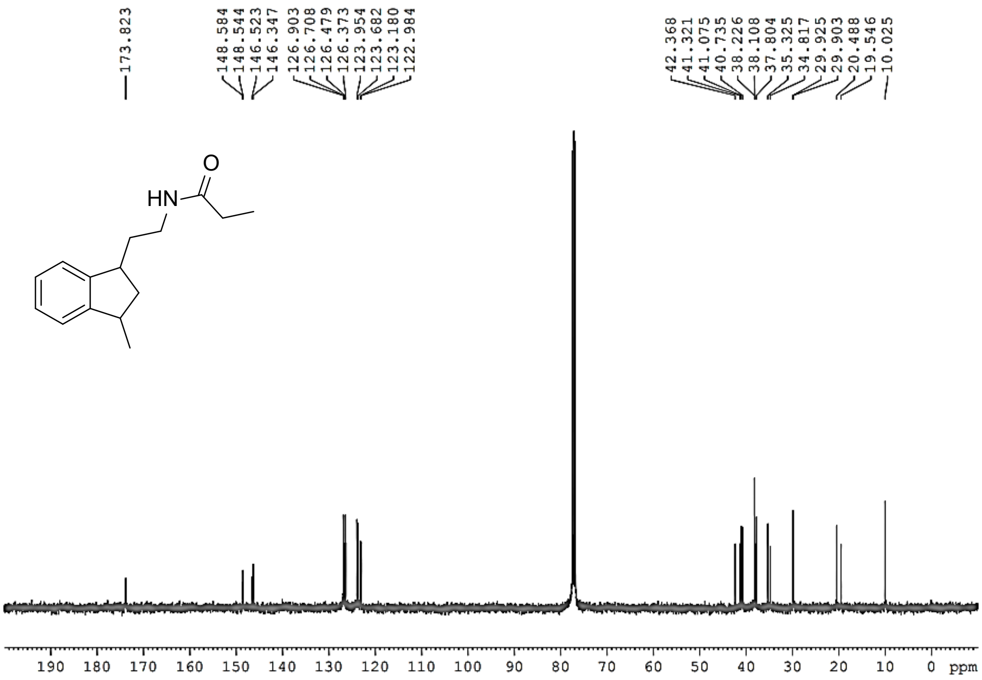

Supplementary Figure 143. ^{1}H NMR spectra of compound **24** (400 MHz, $CDCl_3$)

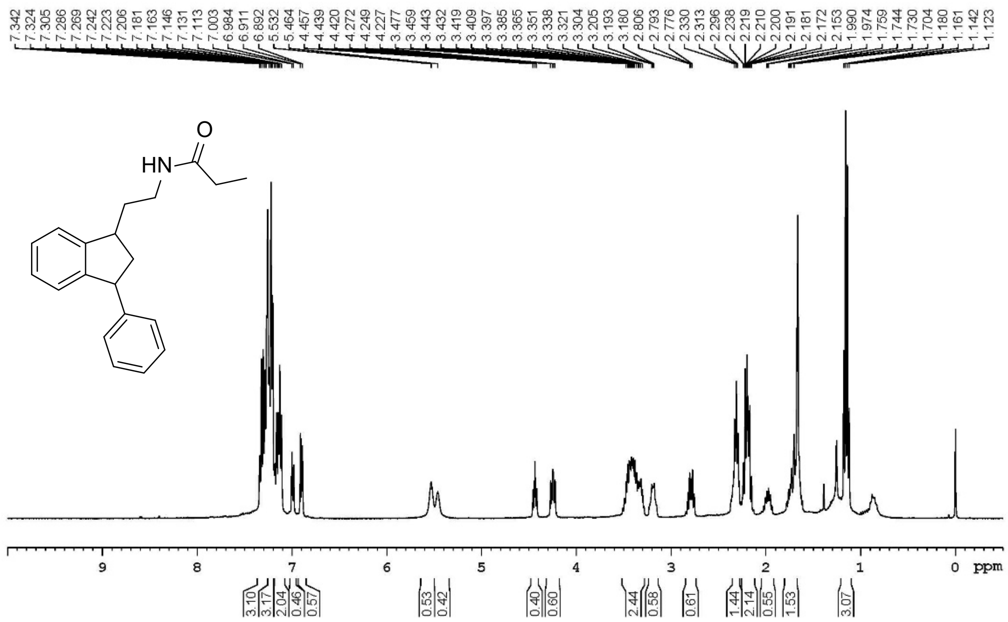

Supplementary Figure 144. ^{13}C NMR spectra of compound **24** (100 MHz, $CDCl_3$)

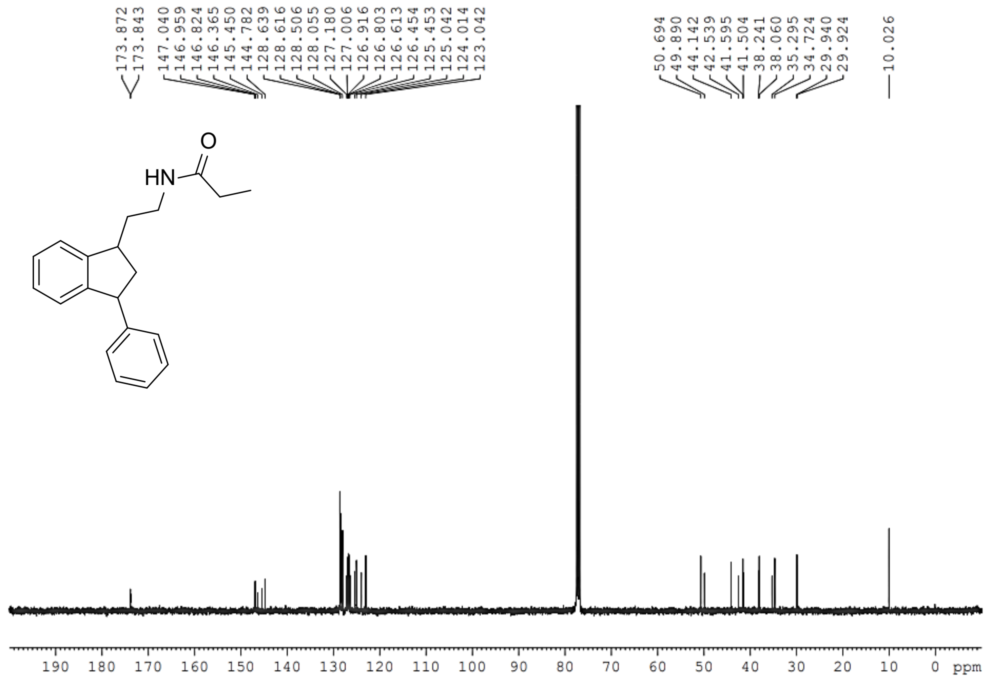

Supplementary Figure 145. ^{1}H NMR spectra of compound **25** (400 MHz, $CDCl_3$)

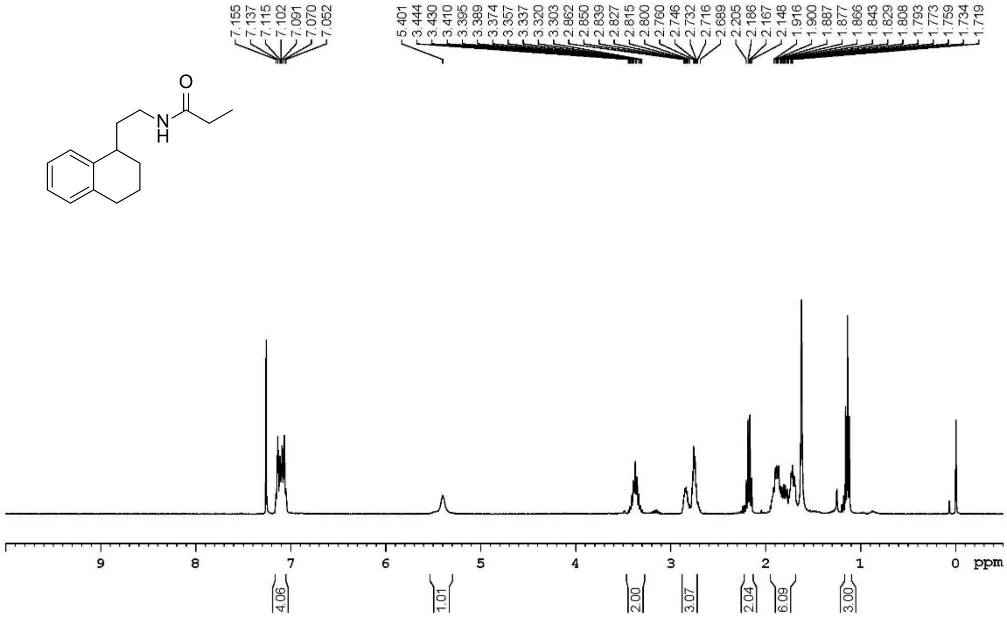

Supplementary Figure 146. ^{13}C NMR spectra of compound **25** (100 MHz, $CDCl_3$)

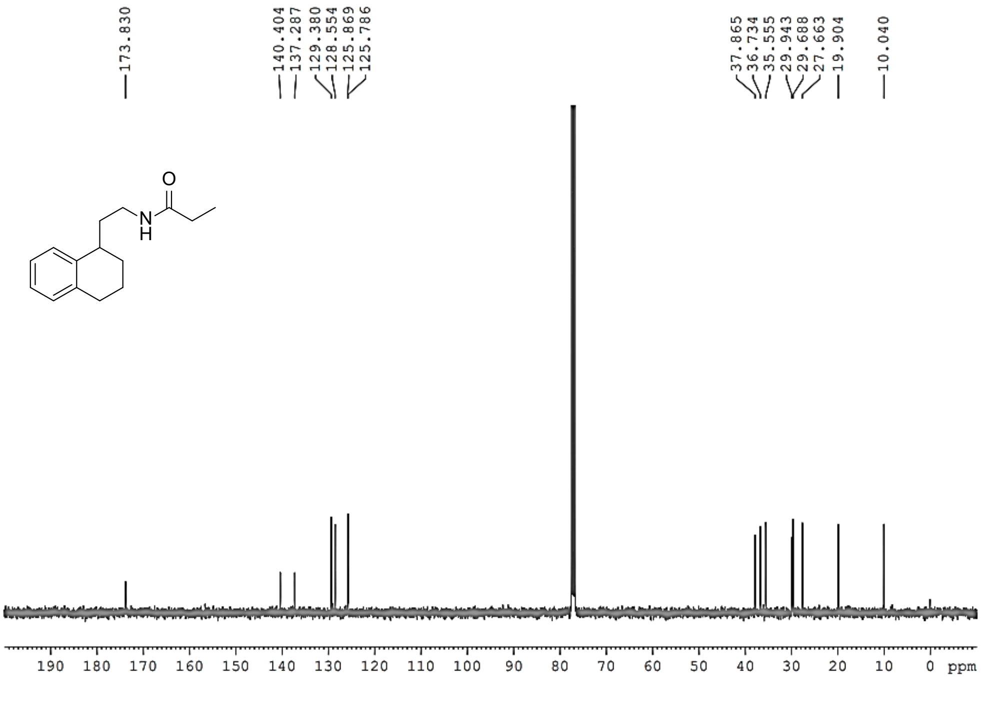

Supplementary Figure 147. ^{1}H NMR spectra of compound **26** (400 MHz, $CDCl_3$)

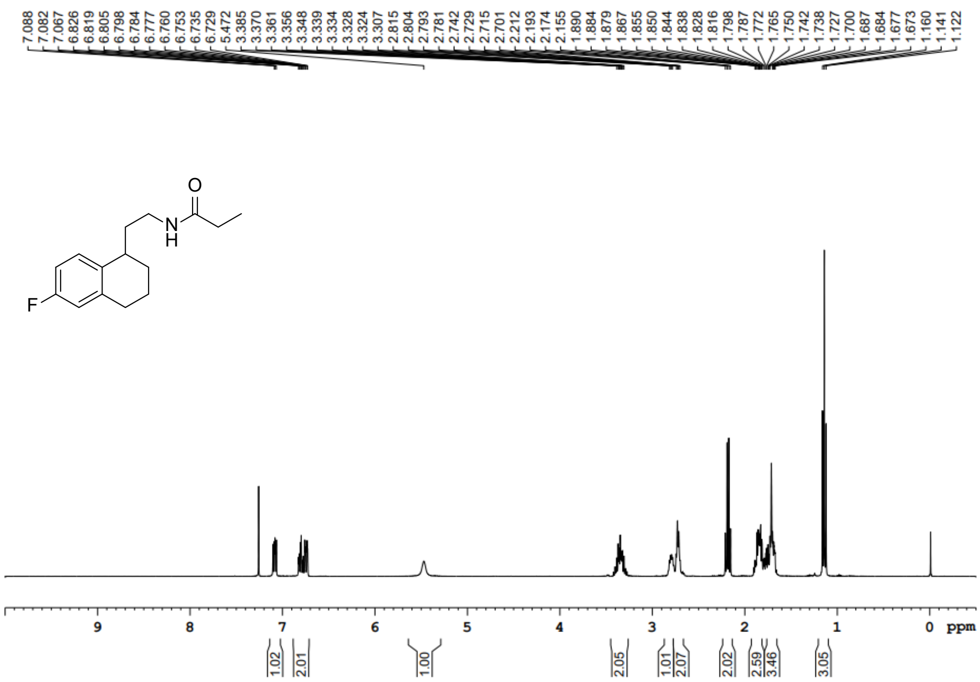

Supplementary Figure 148. ^{13}C NMR spectra of compound **26** (100 MHz, $CDCl_3$)

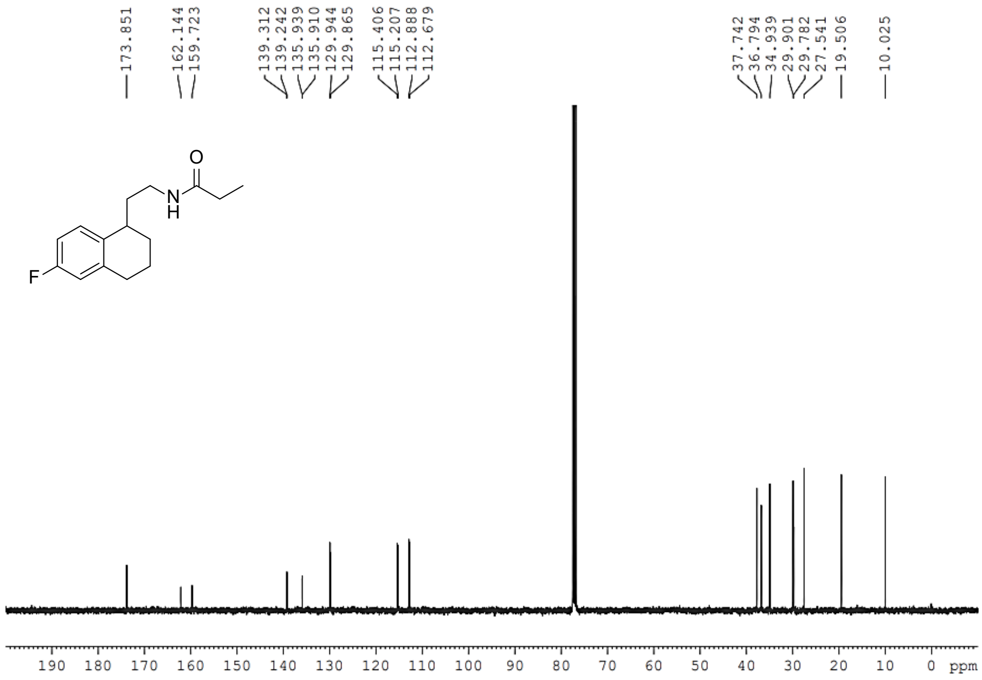

Supplementary Figure 149. ^{19}F NMR spectra of compound **26** (376 MHz, $CDCl_3$)

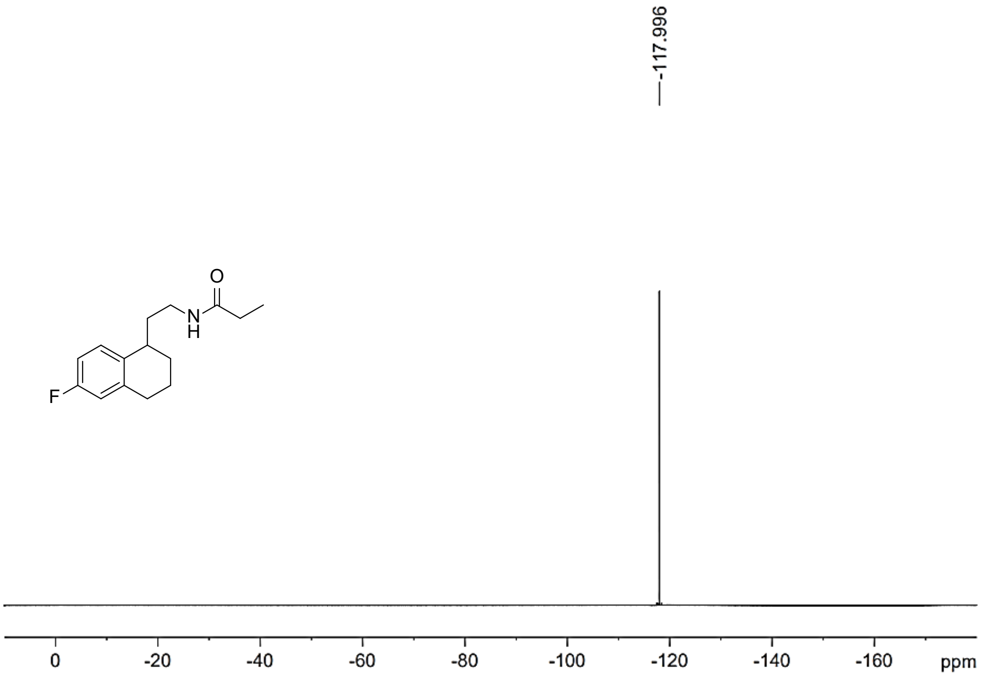

Supplementary Figure 150. ^{1}H NMR spectra of compound **27** (400 MHz, $CDCl_3$)

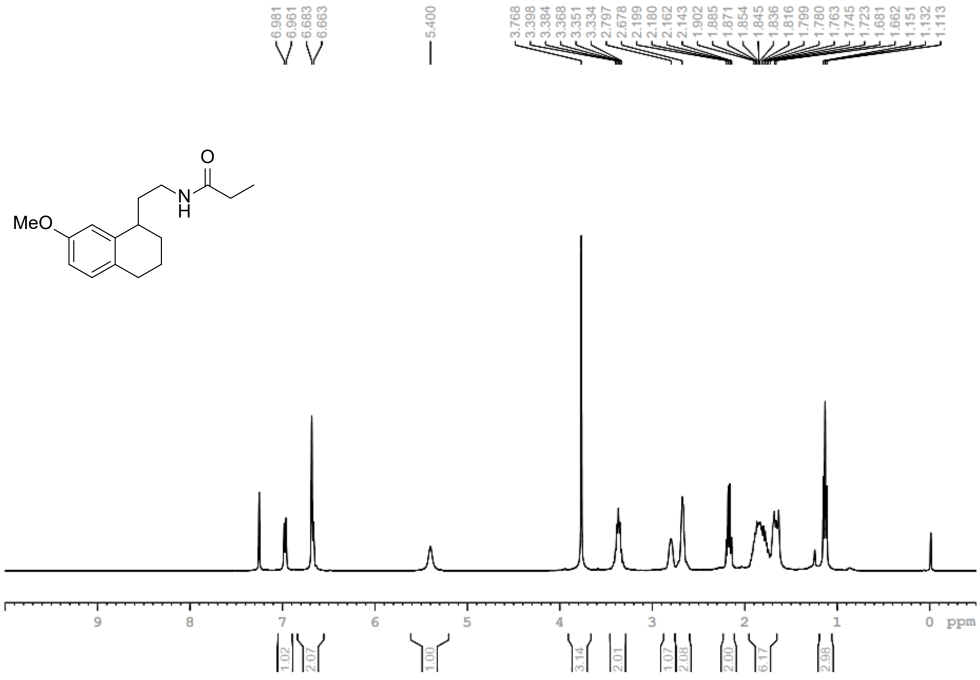

Supplementary Figure 151. ^{13}C NMR spectra of compound **27** (100 MHz, $CDCl_3$)

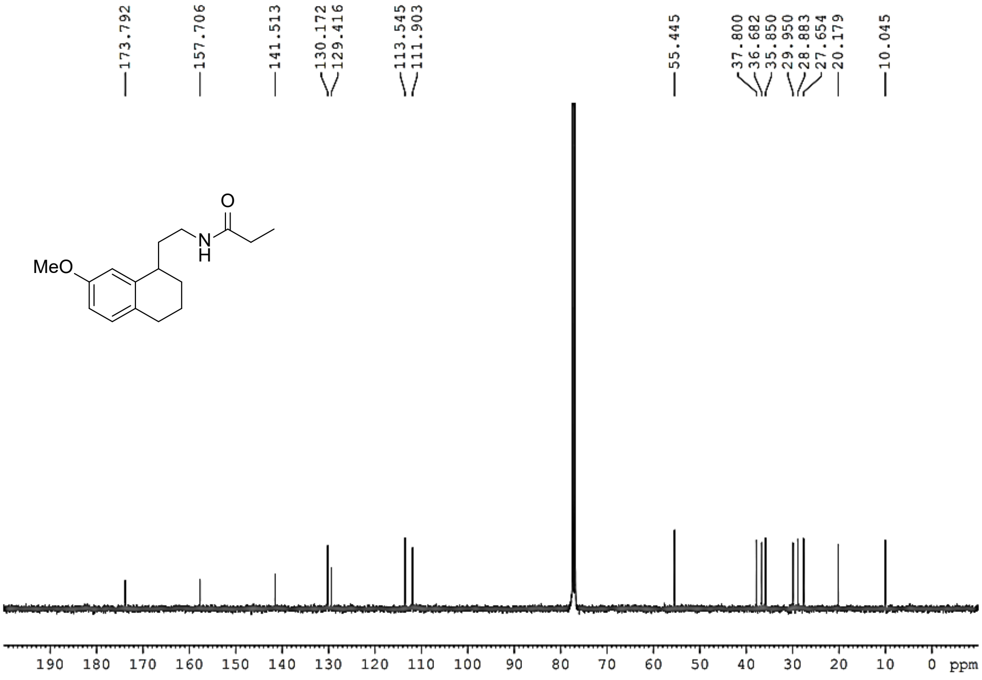

Supplementary Figure 152. ^{1}H NMR spectra of compound **28** (400 MHz, $CDCl_3$)

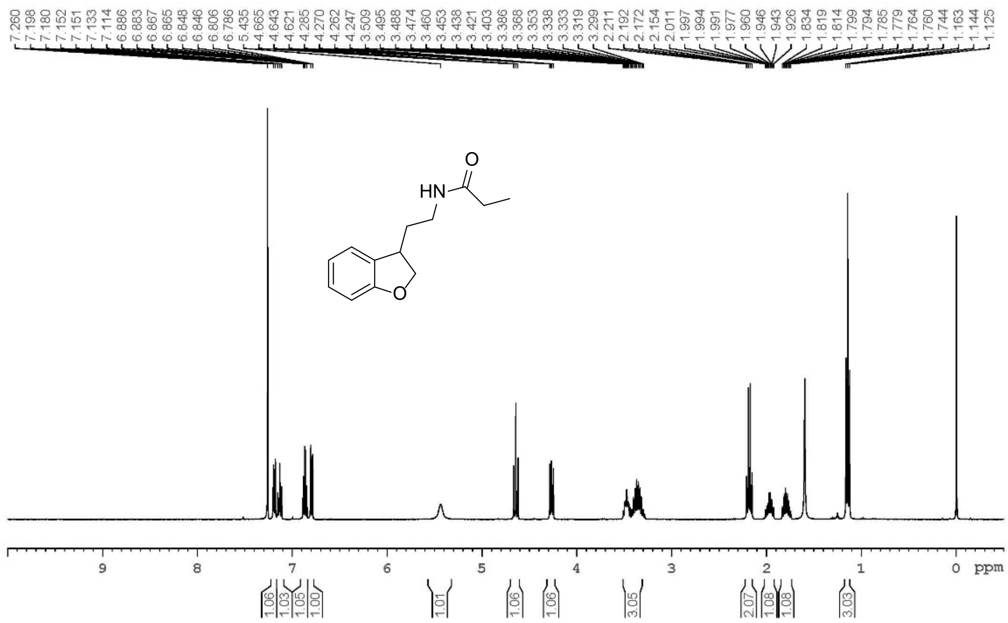

Supplementary Figure 153. ^{13}C NMR spectra of compound **28** (100 MHz, $CDCl_3$)

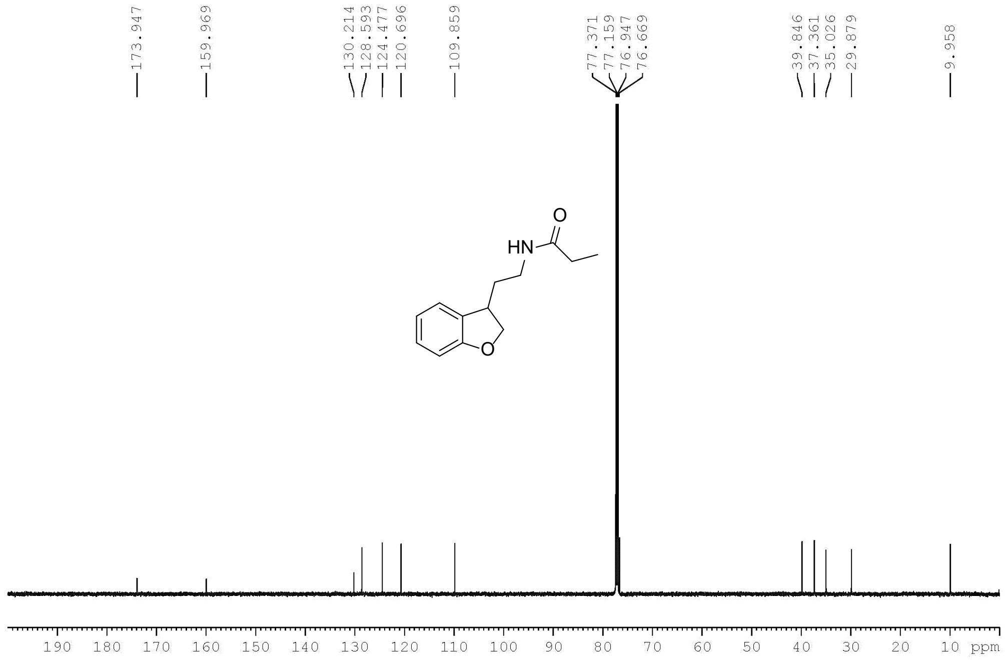

Supplementary Figure 154. ^{1}H NMR spectra of compound **29** (400 MHz, $CDCl_3$)

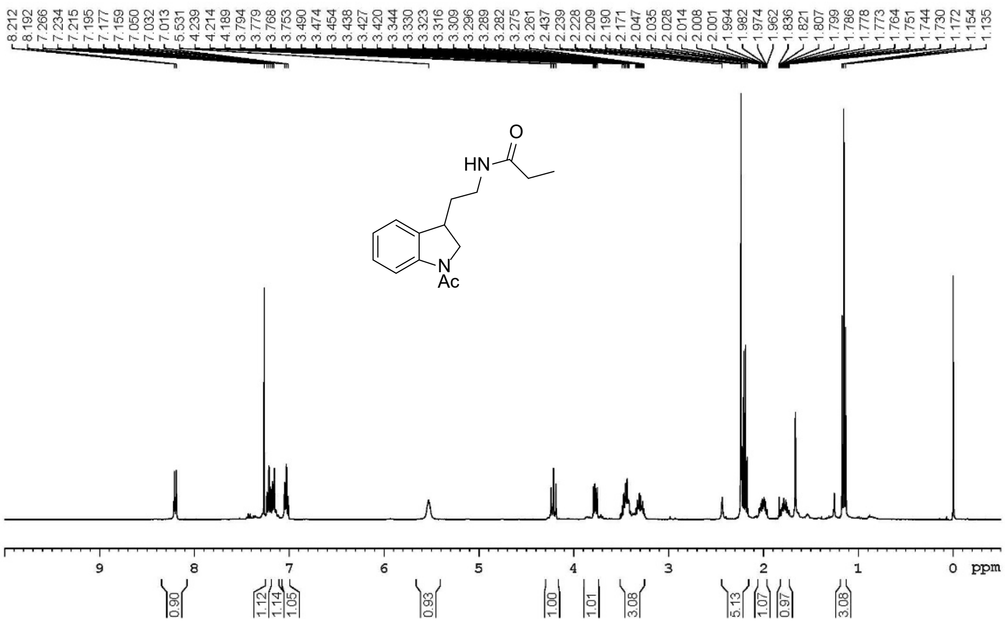

Supplementary Figure 155. ^{13}C NMR spectra of compound **29** (100 MHz, $CDCl_3$)

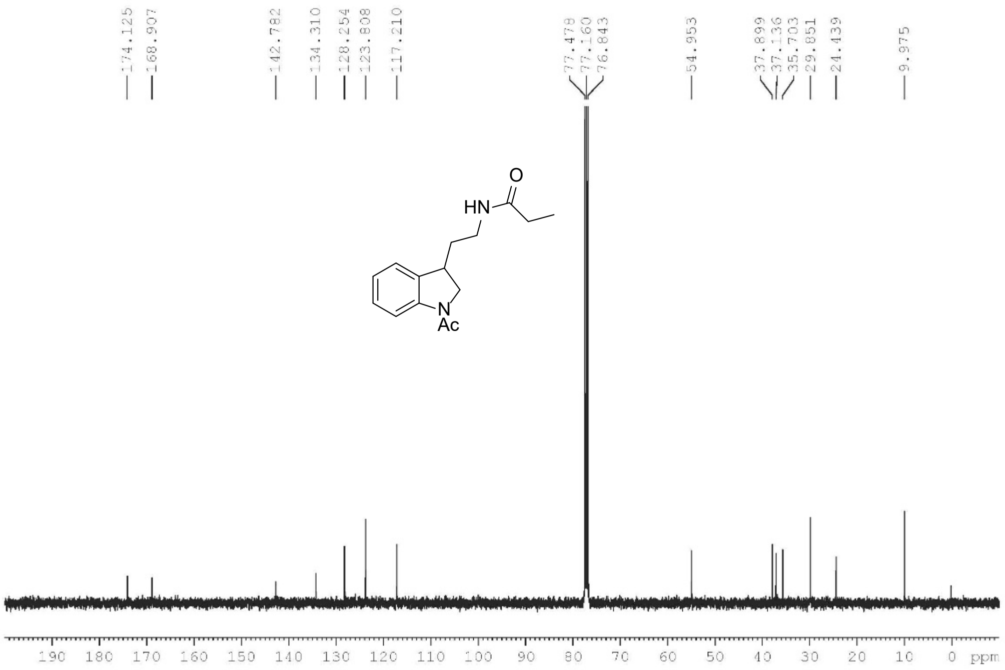

Supplementary Figure 156. ^{1}H NMR spectra of compound **30** (400 MHz, $CDCl_3$)

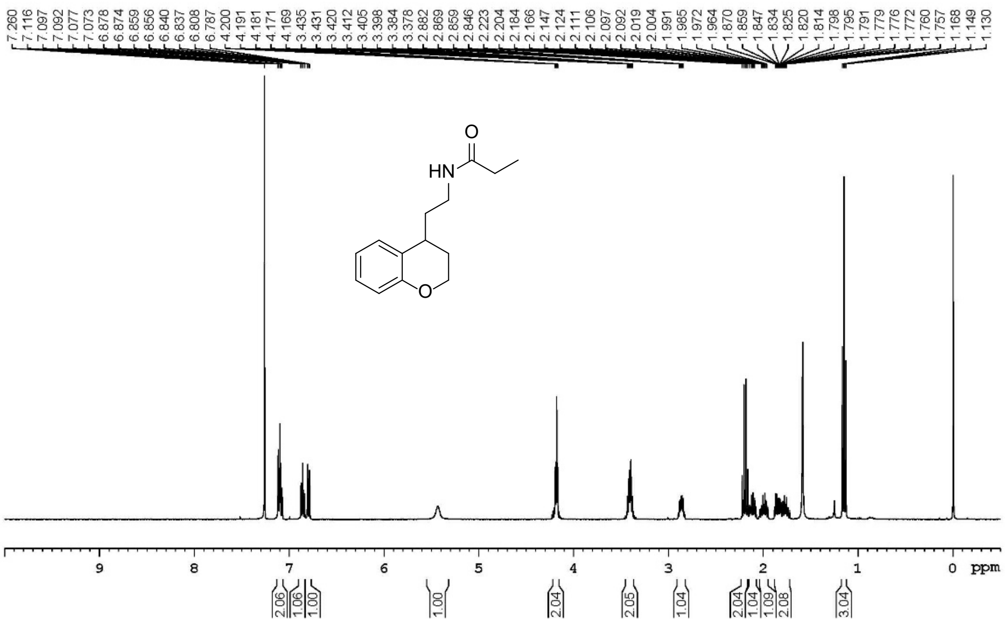

Supplementary Figure 157. ^{13}C NMR spectra of compound **30** (100 MHz, $CDCl_3$)

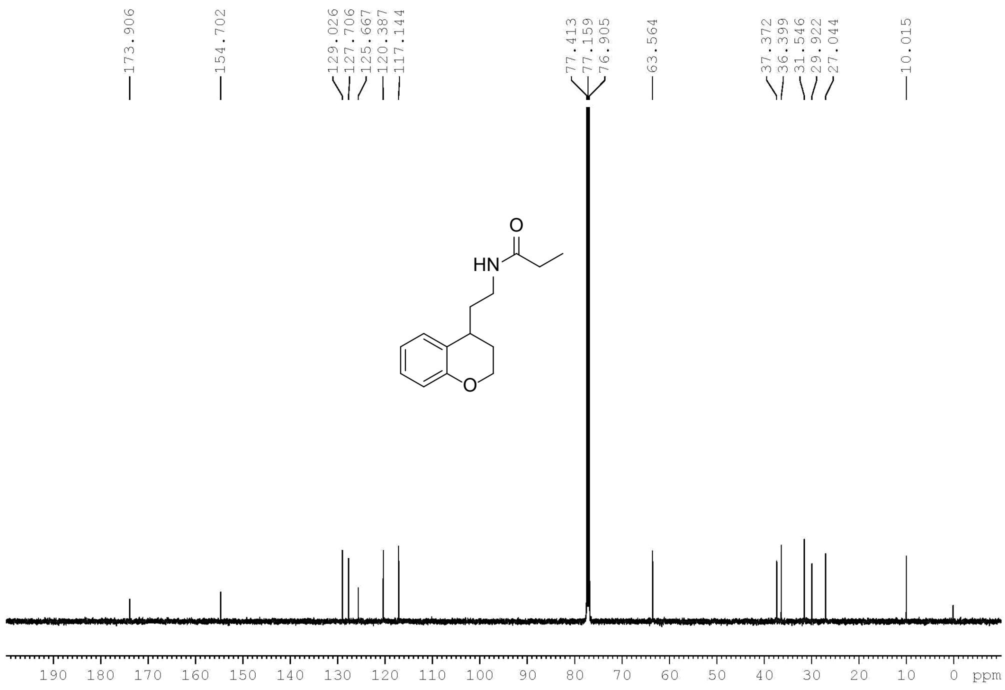

Supplementary Figure 158. ^{1}H NMR spectra of compound **31** (400 MHz, $CDCl_3$)

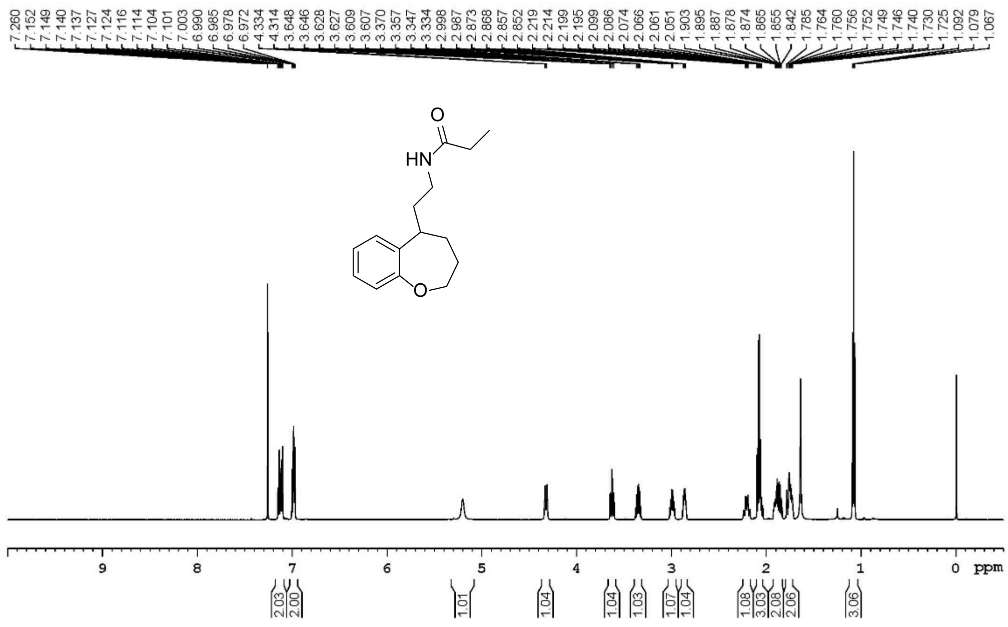

Supplementary Figure 159. ^{13}C NMR spectra of compound **31** (100 MHz, $CDCl_3$)

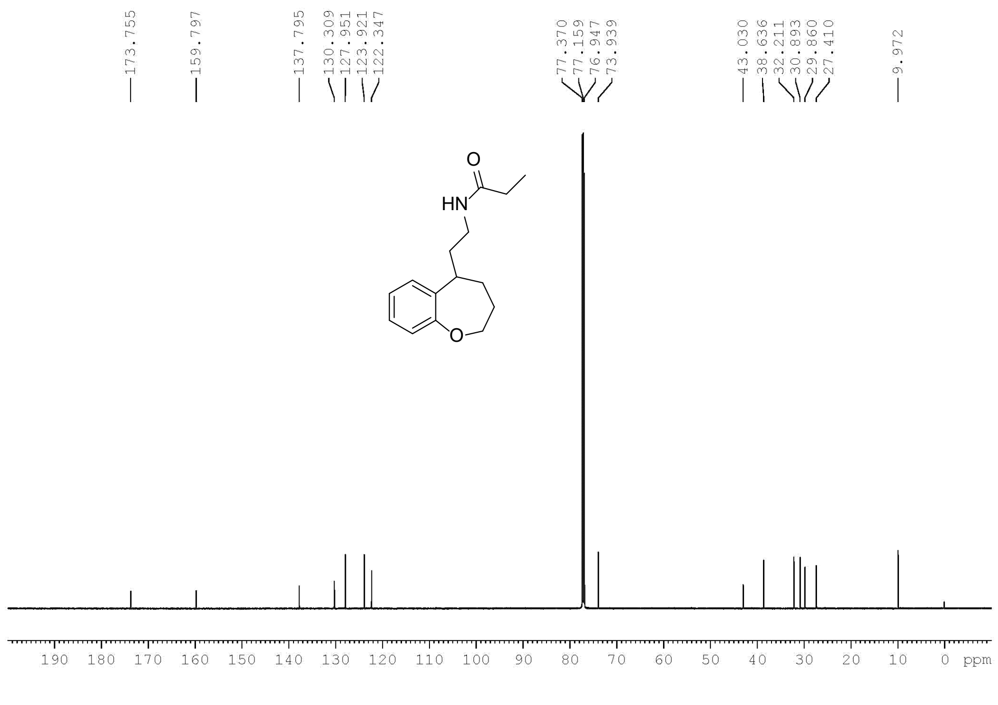

Supplementary Figure 160. ^{1}H NMR spectra of compound **32** (400 MHz, $CDCl_3$)

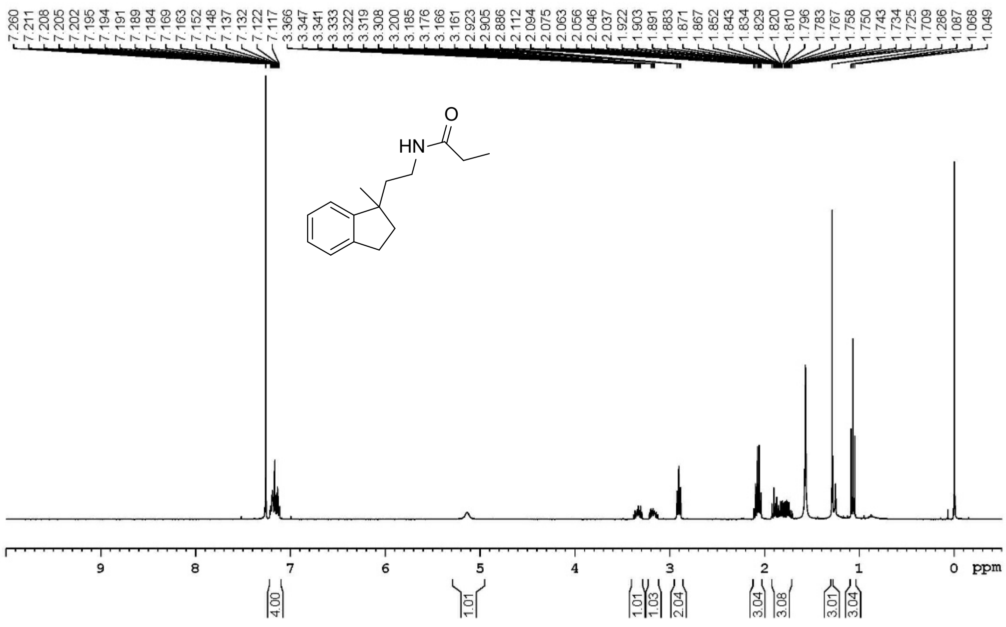

Supplementary Figure 161. ^{13}C NMR spectra of compound **32** (100 MHz, $CDCl_3$)

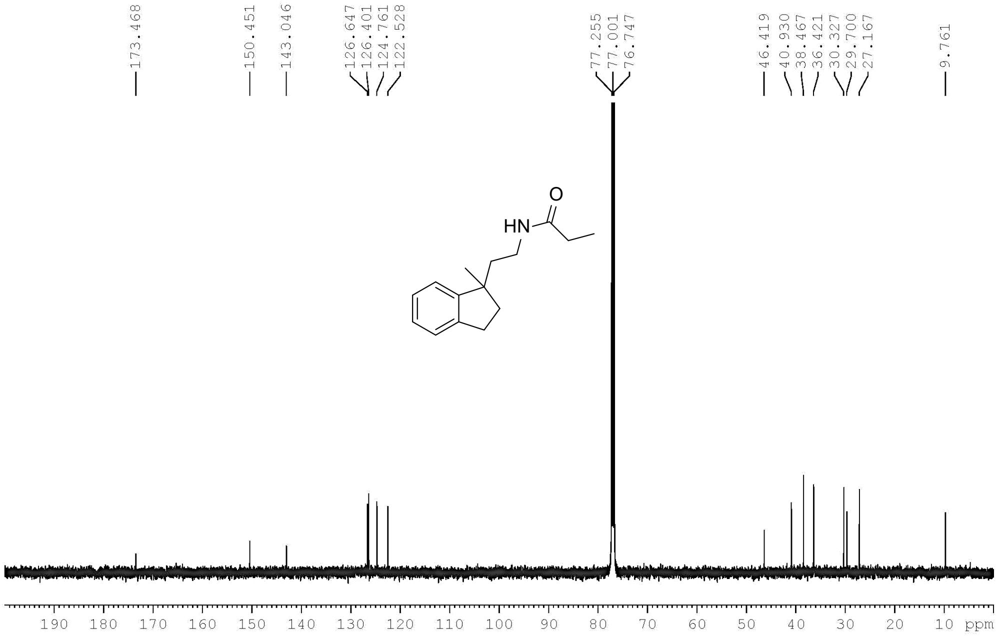

Supplementary Figure 162. ^{1}H NMR spectra of compound **33** (400 MHz, $CDCl_3$)

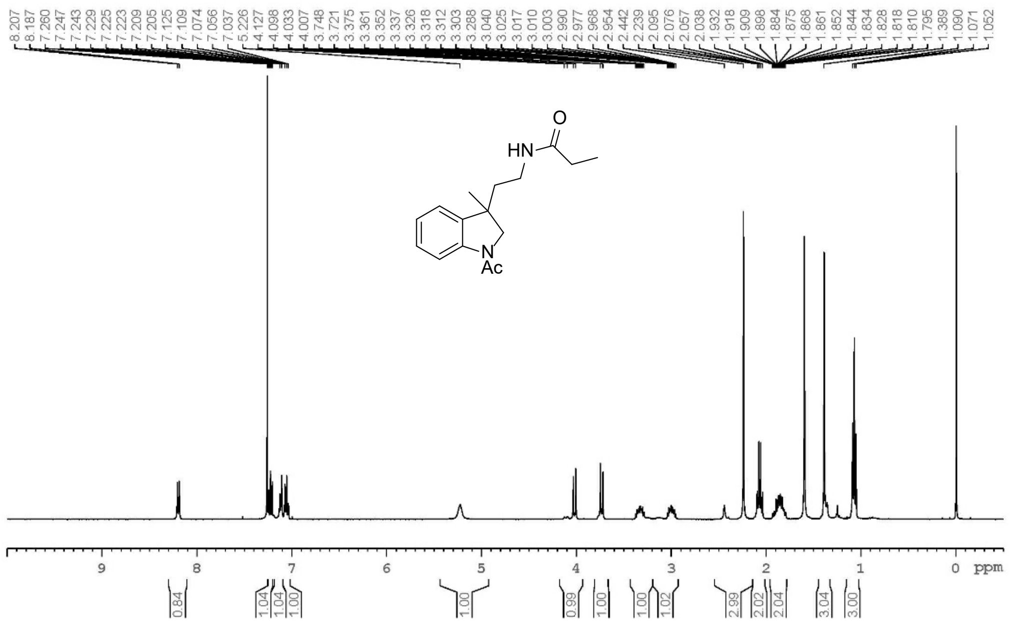

Supplementary Figure 163. ^{13}C NMR spectra of compound **33** (100 MHz, $CDCl_3$)

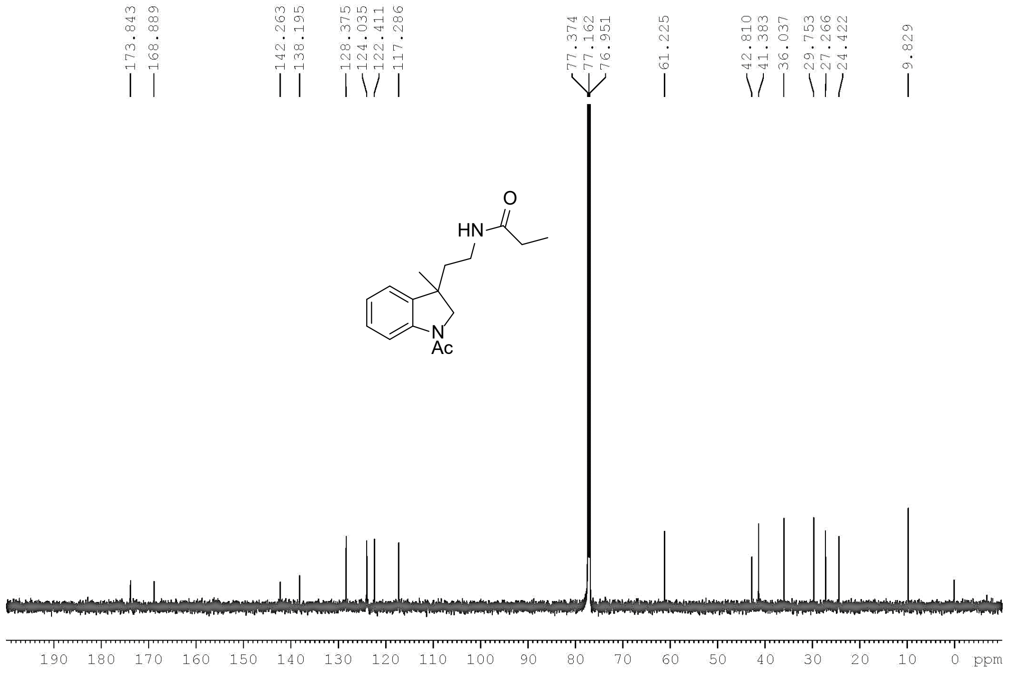

Supplementary Figure 164. ^{1}H NMR spectra of compound **34** (400 MHz, $CDCl_3$)

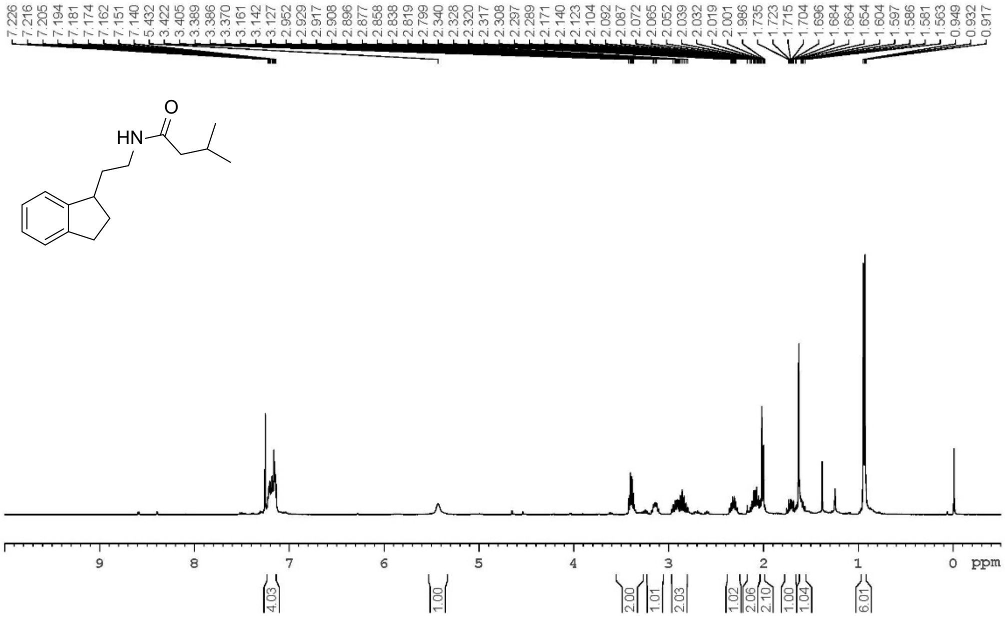

Supplementary Figure 165. ^{13}C NMR spectra of compound **34** (100 MHz, $CDCl_3$)

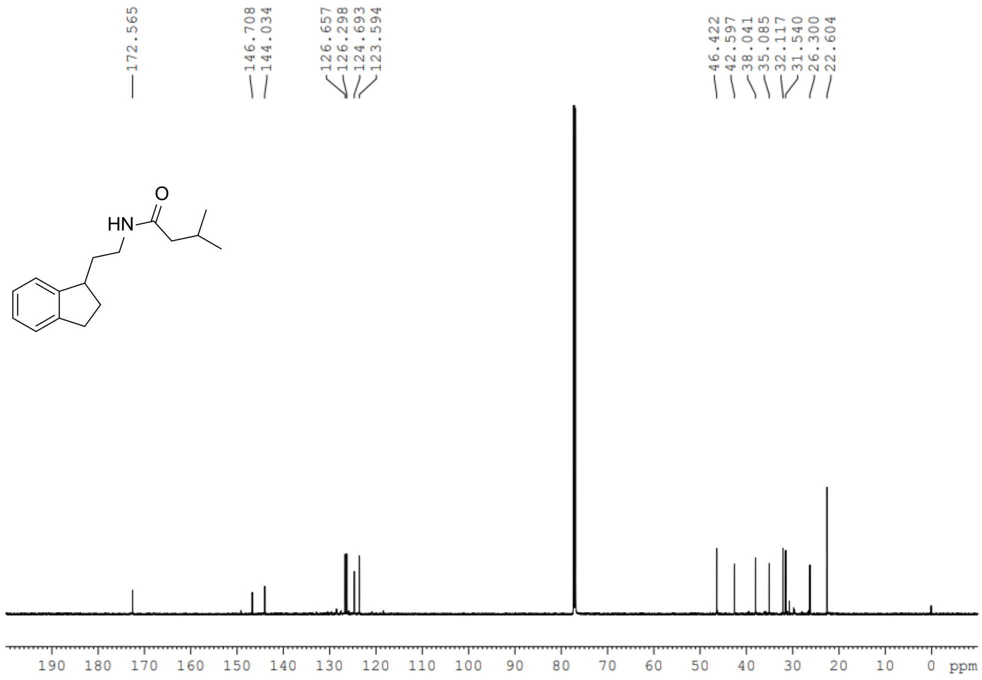

Supplementary Figure 166. ^{1}H NMR spectra of compound **35** (600 MHz, $CDCl_3$)

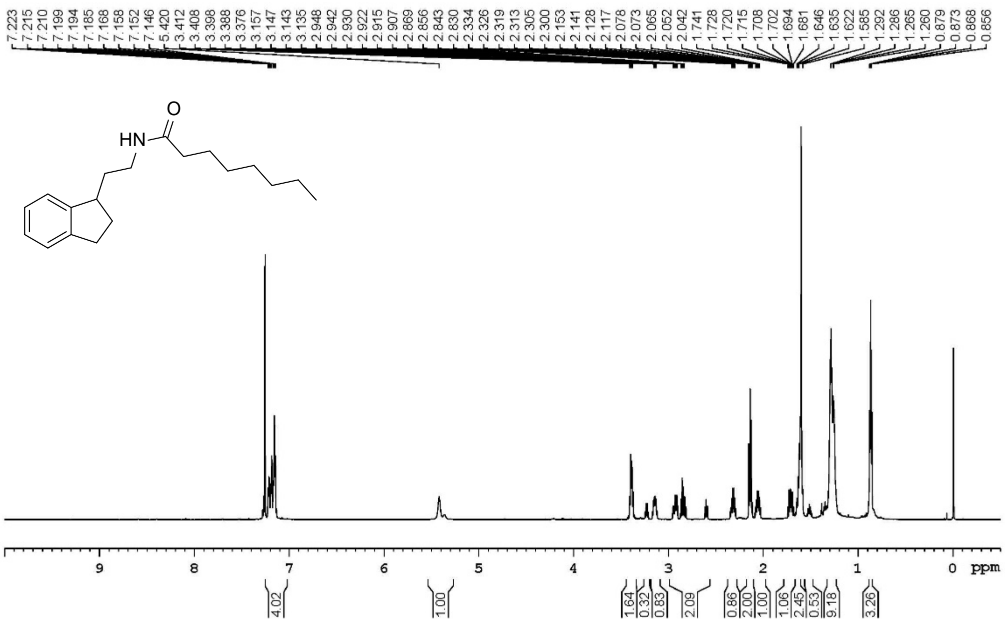

Supplementary Figure 167. ^{13}C NMR spectra of compound **35** (150 MHz, $CDCl_3$)

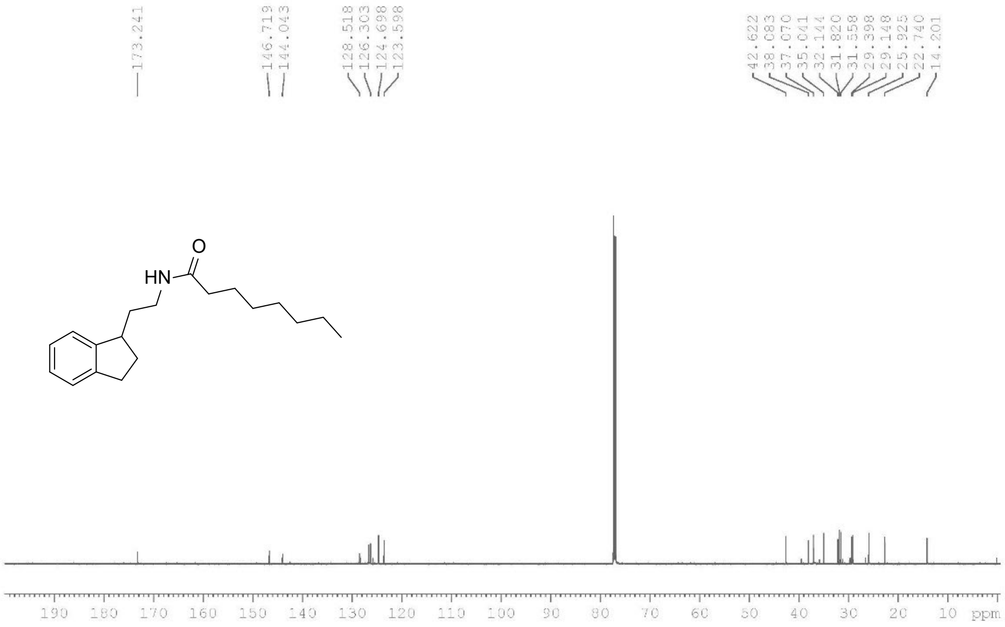

Supplementary Figure 168. ^{1}H NMR spectra of compound **36** (400 MHz, $CDCl_3$)

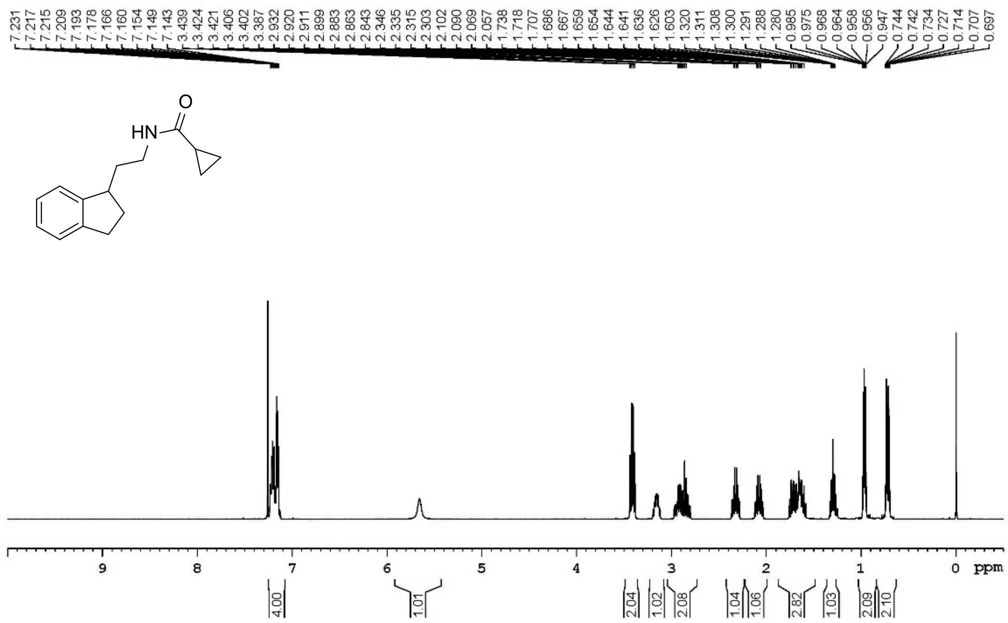

Supplementary Figure 169. ^{13}C NMR spectra of compound **36** (100 MHz, $CDCl_3$)

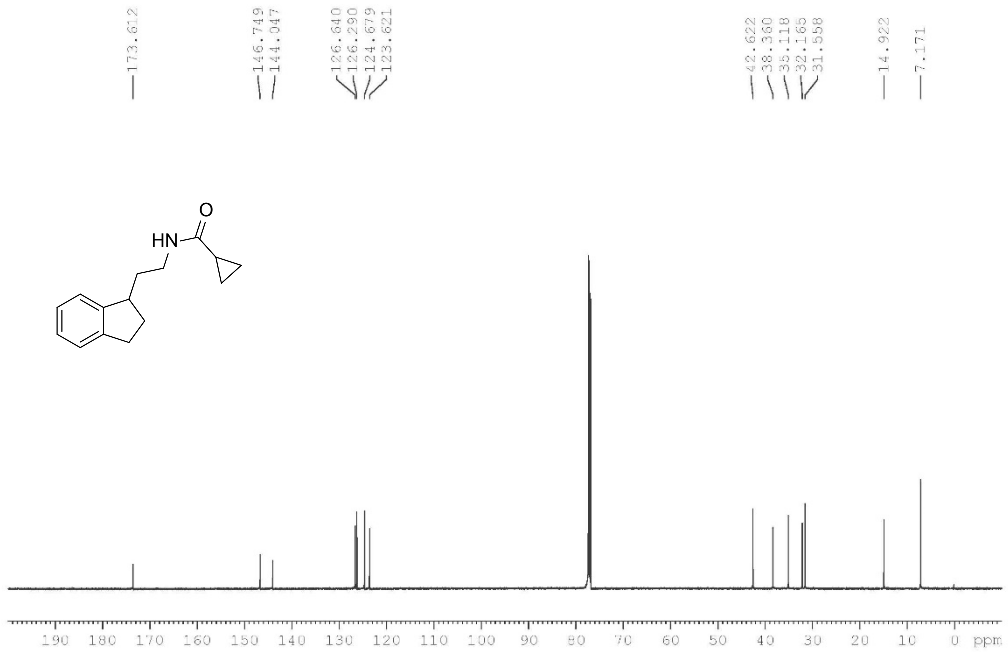

Supplementary Figure 170. ^{1}H NMR spectra of compound **37** (400 MHz, $CDCl_3$)

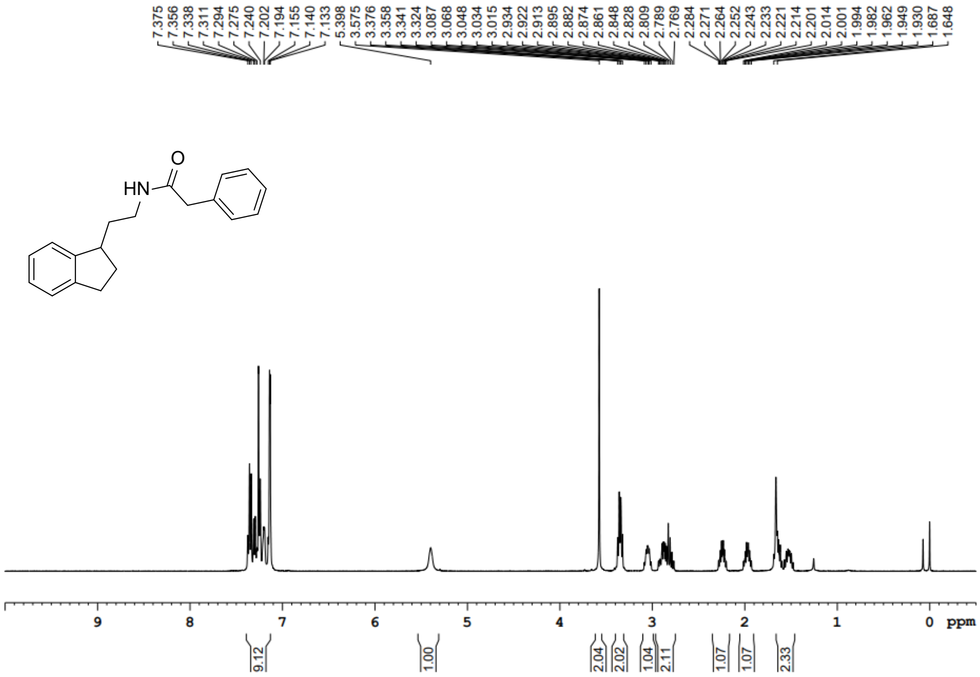

Supplementary Figure 171. ^{13}C NMR spectra of compound **37** (100 MHz, $CDCl_3$)

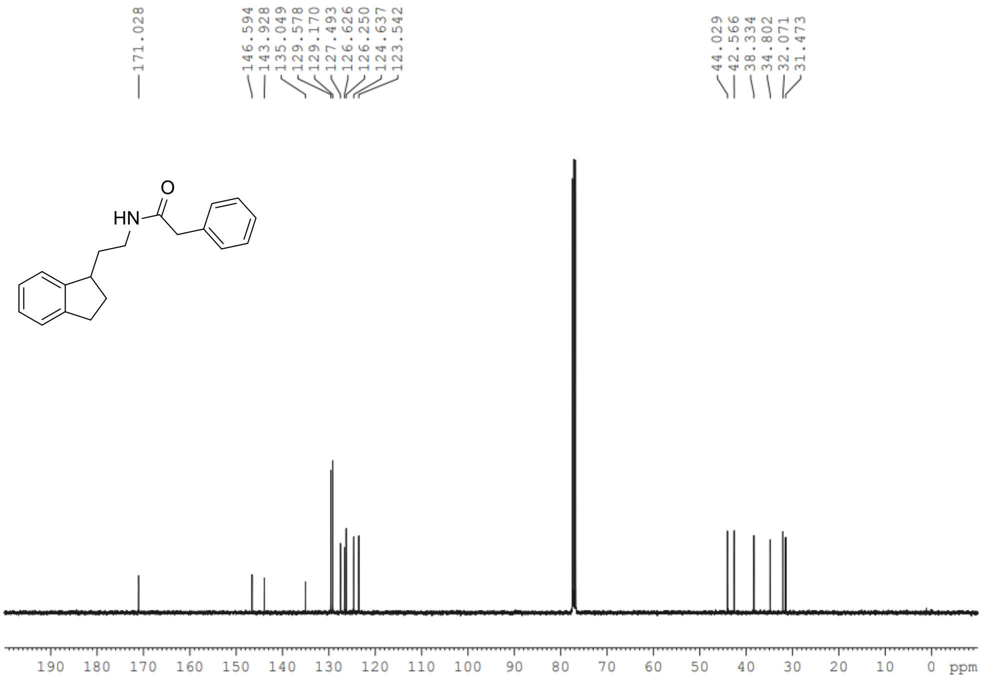

Supplementary Figure 172. ^{1}H NMR spectra of compound **38** (400 MHz, $CDCl_3$)

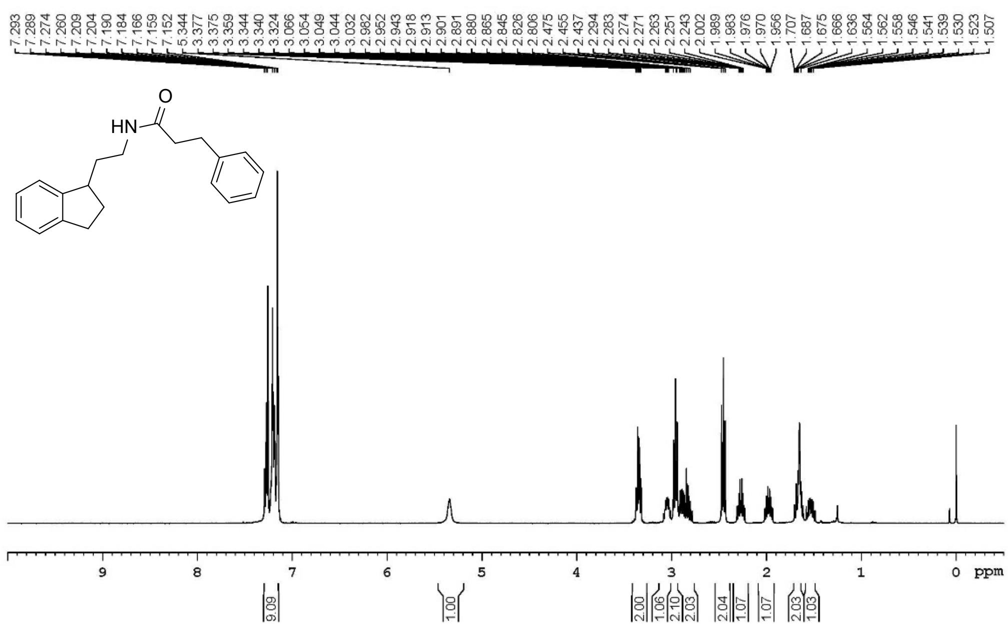

Supplementary Figure 173. ^{13}C NMR spectra of compound **38** (100 MHz, $CDCl_3$)

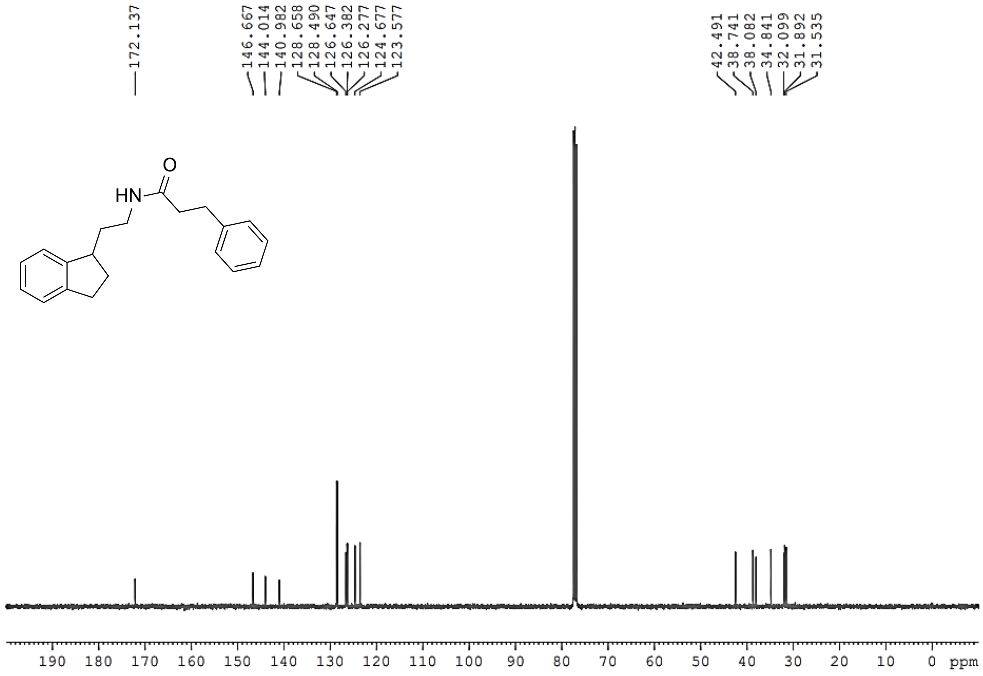

Supplementary Figure 174. ^{1}H NMR spectra of compound **39** (600 MHz, $CDCl_3$)

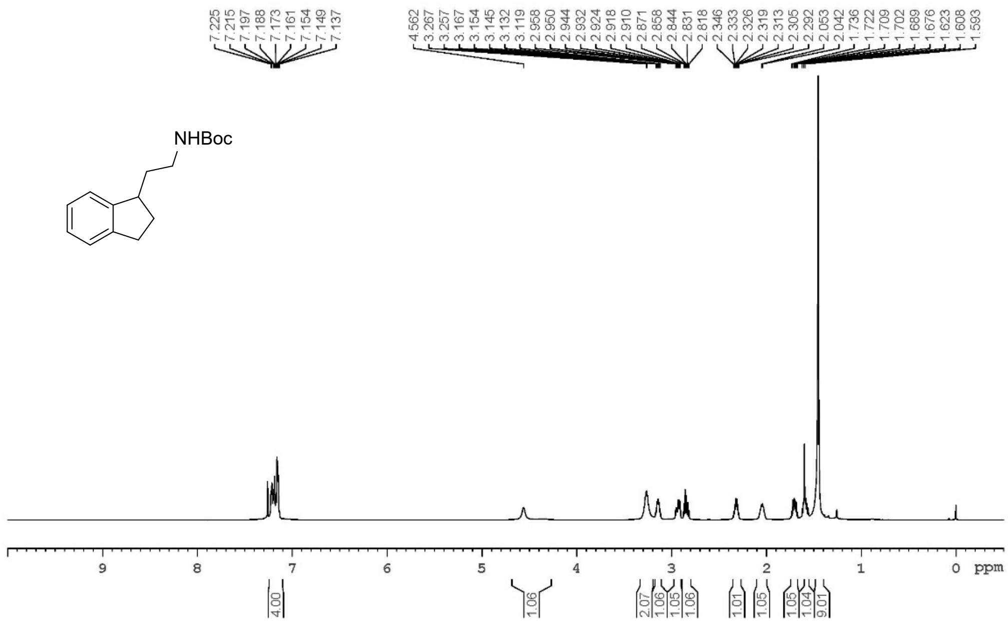

Supplementary Figure 175. ^{13}C NMR spectra of compound **39** (150 MHz, $CDCl_3$)

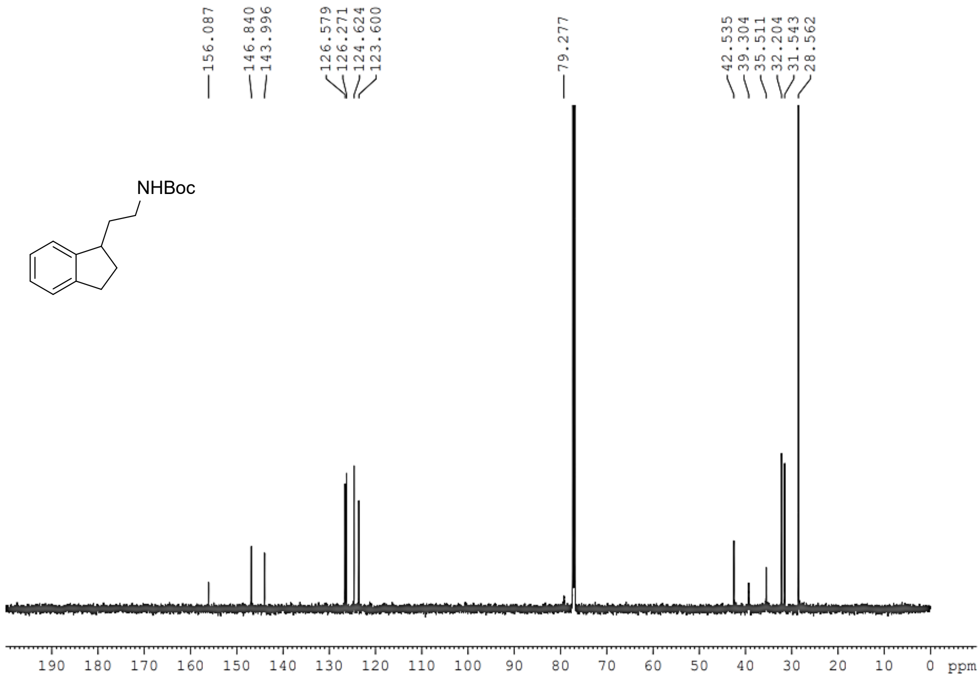

Supplementary Figure 176. ^{1}H NMR spectra of compound **40** (400 MHz, $CDCl_3$)

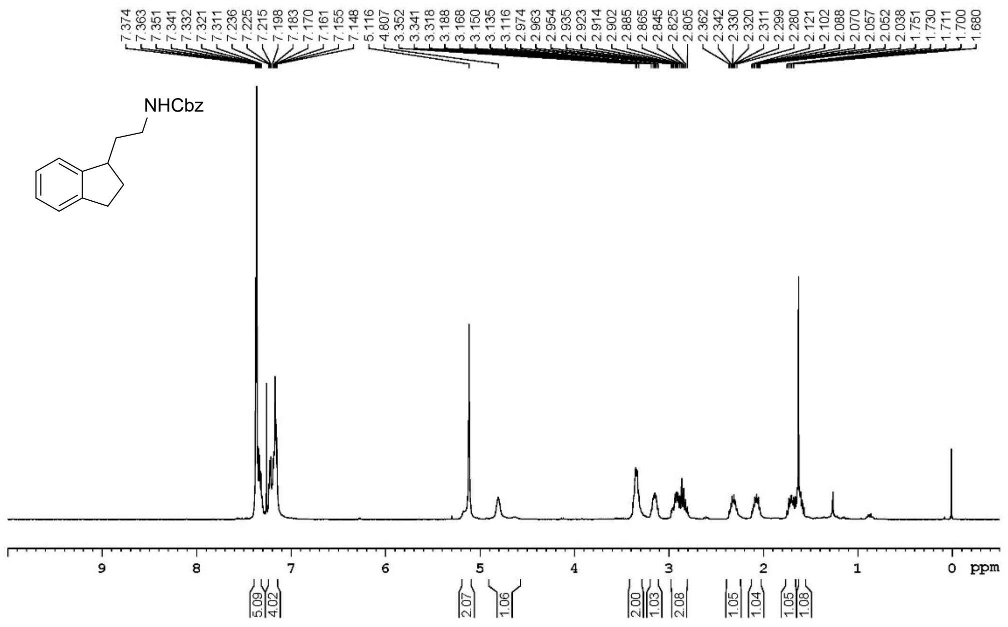

Supplementary Figure 177. ^{13}C NMR spectra of compound **40** (100 MHz, $CDCl_3$)

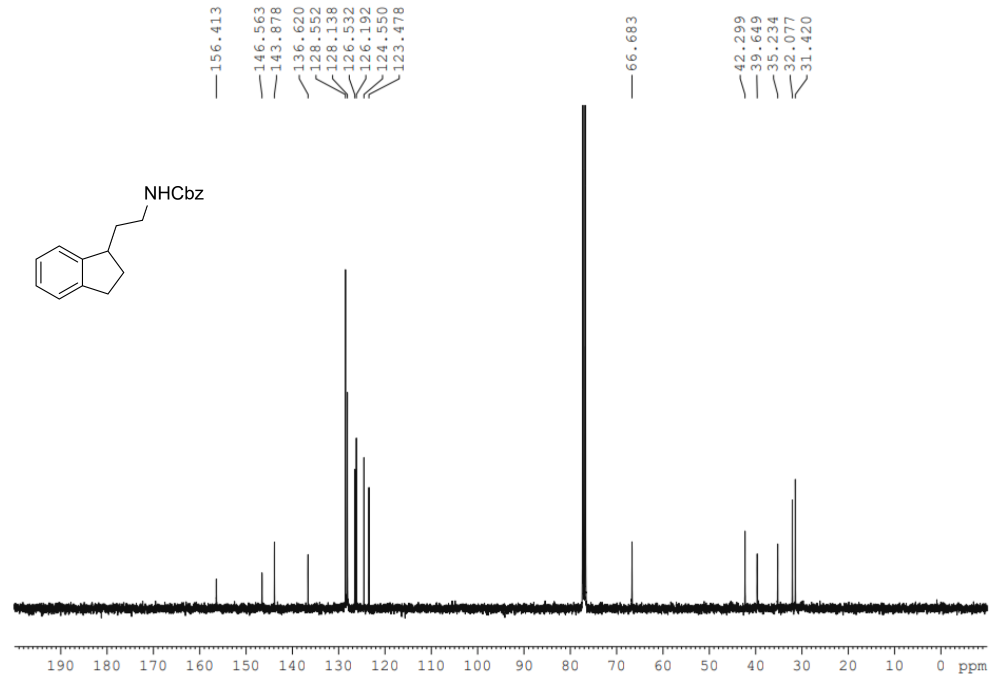

Supplementary Figure 178. ^{1}H NMR spectra of compound **41** (400 MHz, $CDCl_3$)

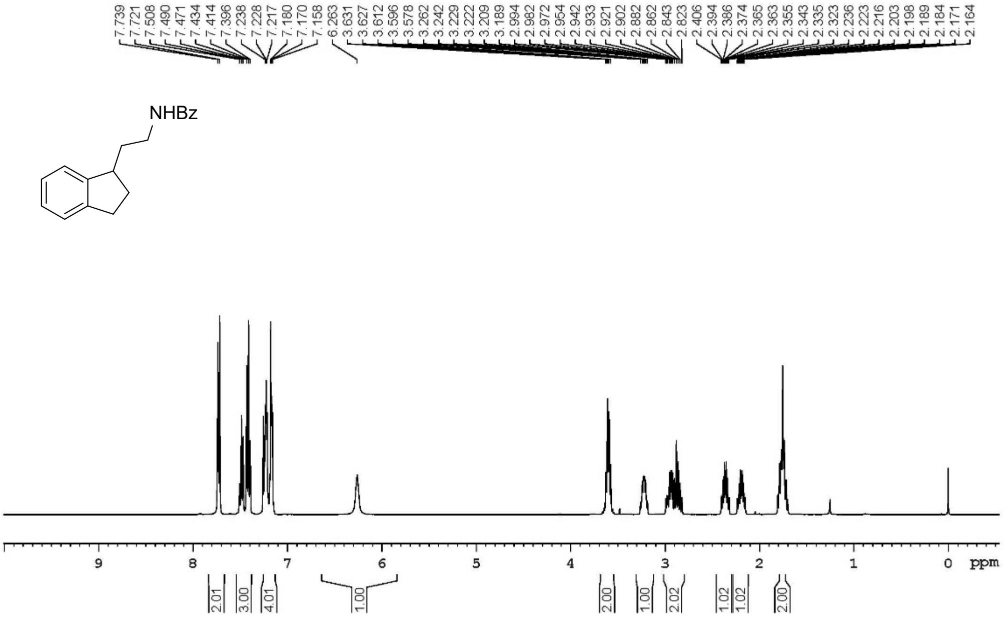

Supplementary Figure 179. ^{13}C NMR spectra of compound **41** (100 MHz, $CDCl_3$)

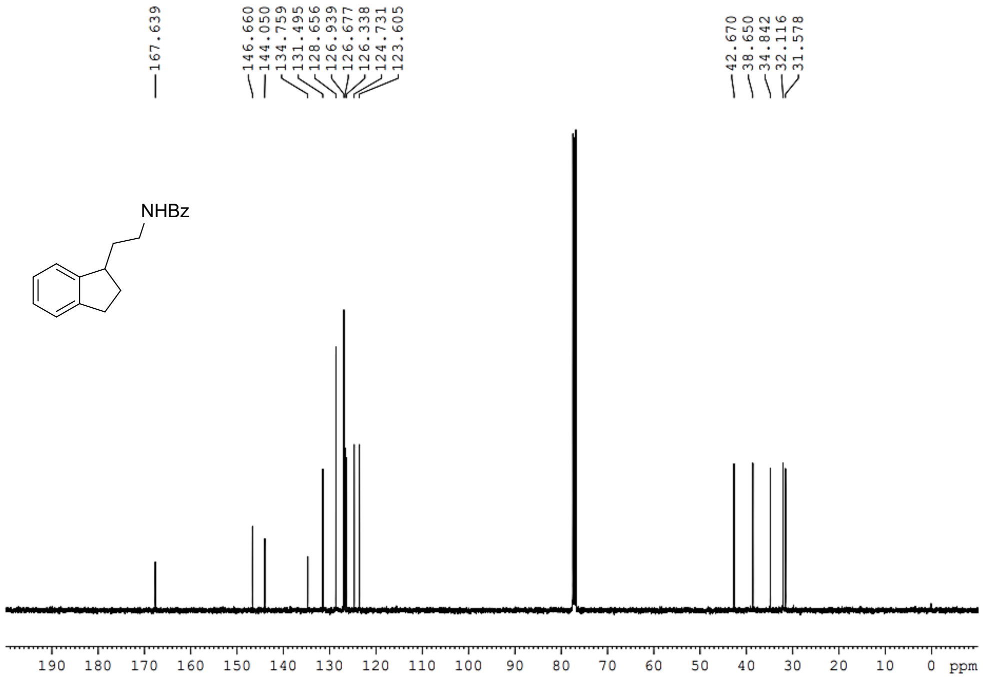

Supplementary Figure 180. ^{1}H NMR spectra of compound **42** (400 MHz, $CDCl_3$)

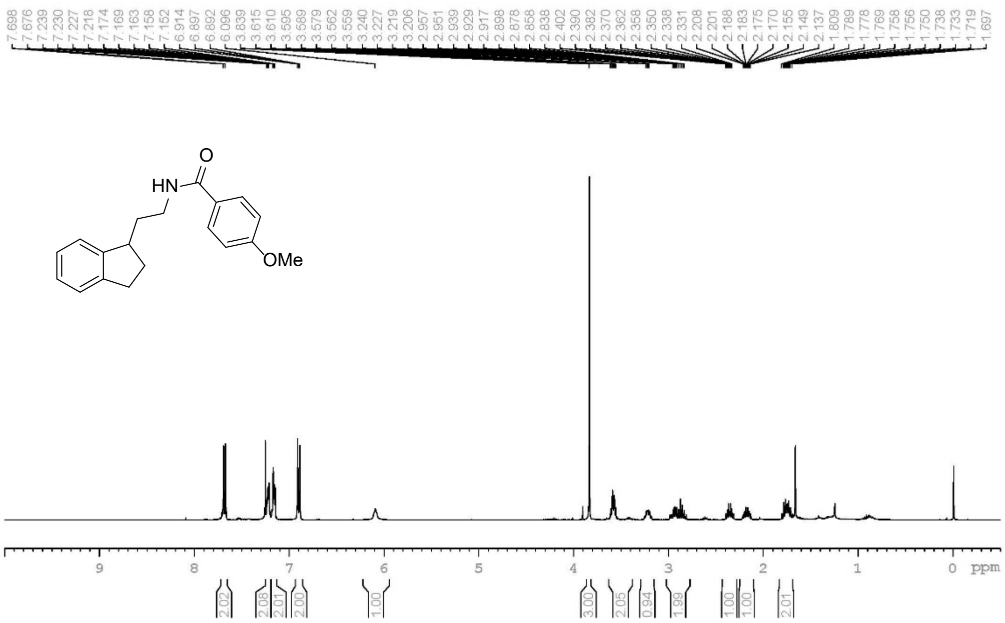

Supplementary Figure 181. ^{13}C NMR spectra of compound **42** (100 MHz, $CDCl_3$)

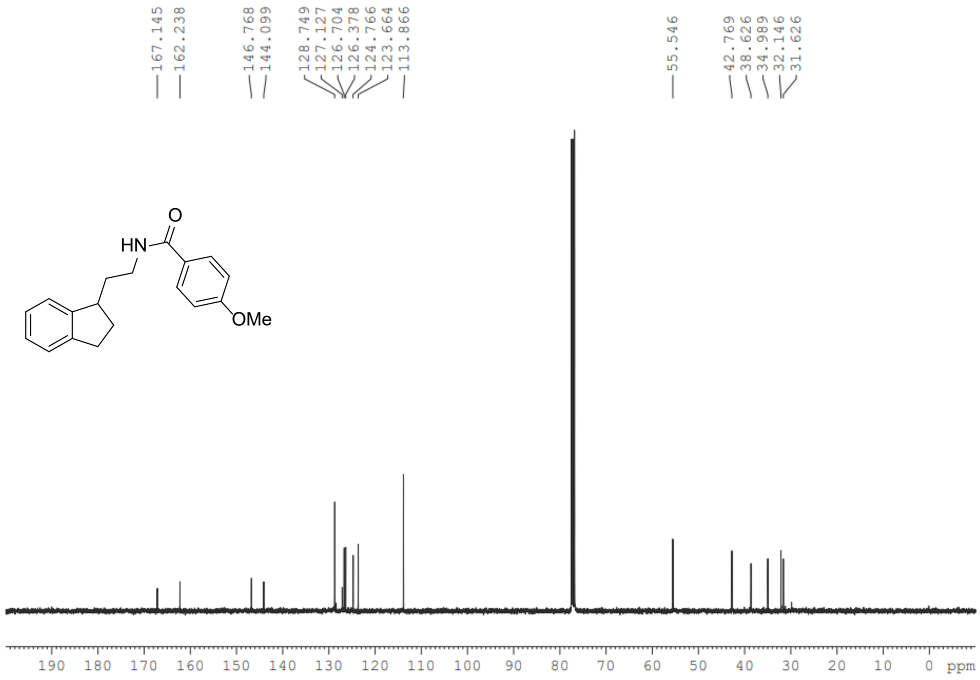

Supplementary Figure 182. ^{1}H NMR spectra of compound **43** (400 MHz, $CDCl_3$)

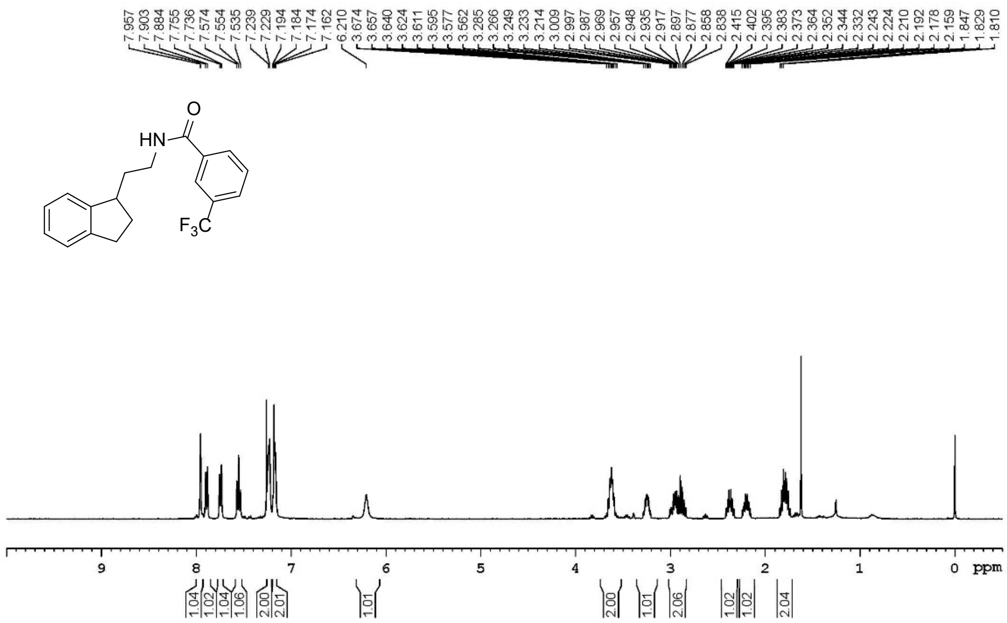

Supplementary Figure 183. ^{13}C NMR spectra of compound **43** (100 MHz, $CDCl_3$)

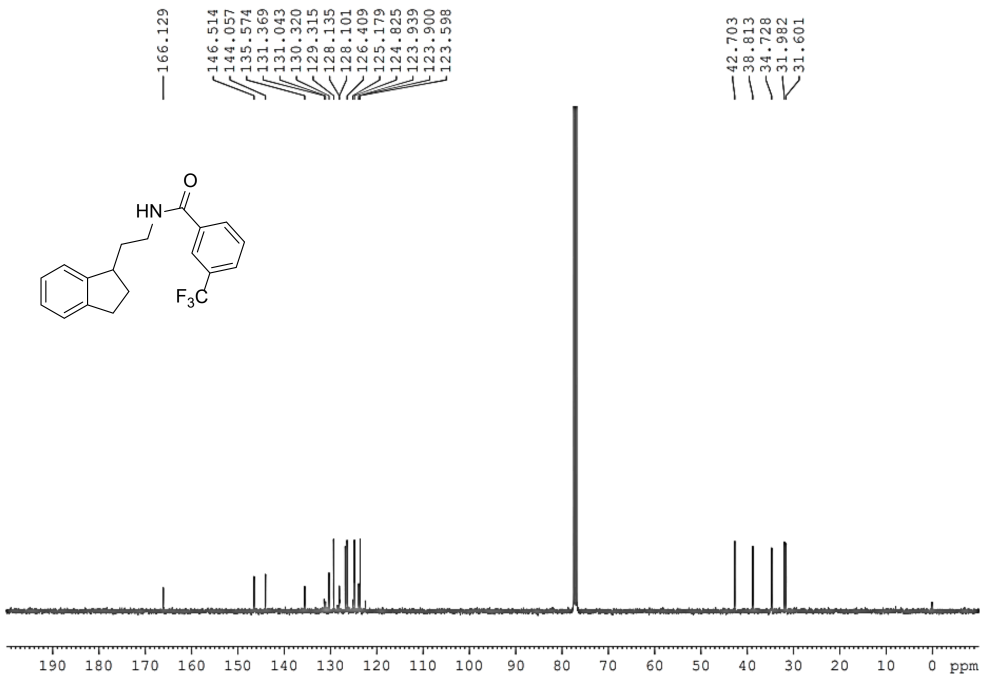

Supplementary Figure 184. ^{19}F NMR spectra of compound **43** (376 MHz, $CDCl_3$)

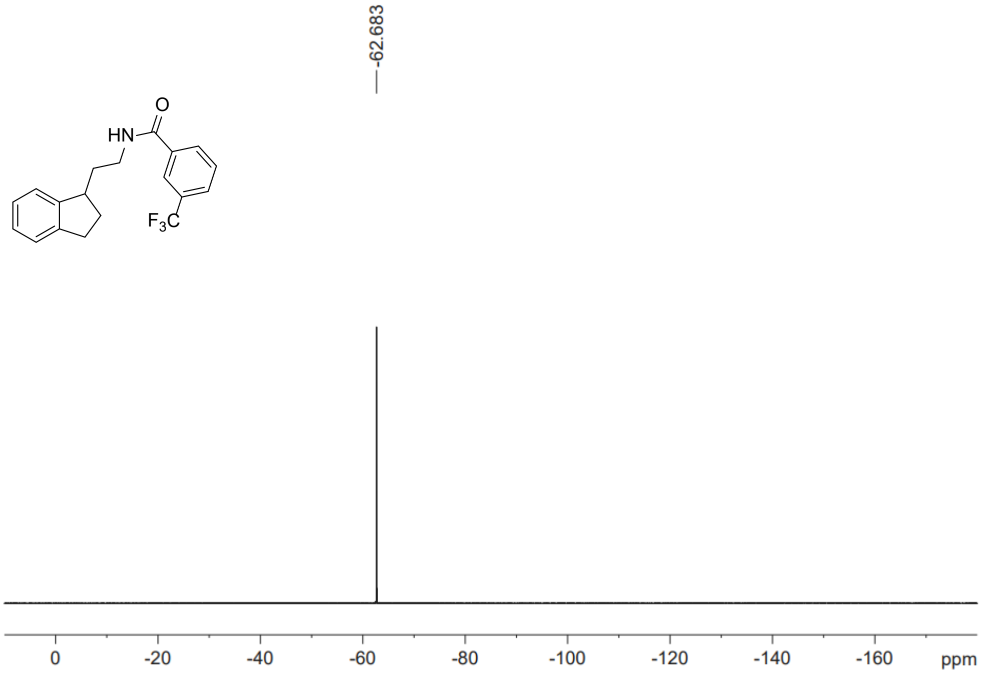

Supplementary Figure 185. ^{1}H NMR spectra of compound **44** (600 MHz, $CDCl_3$)

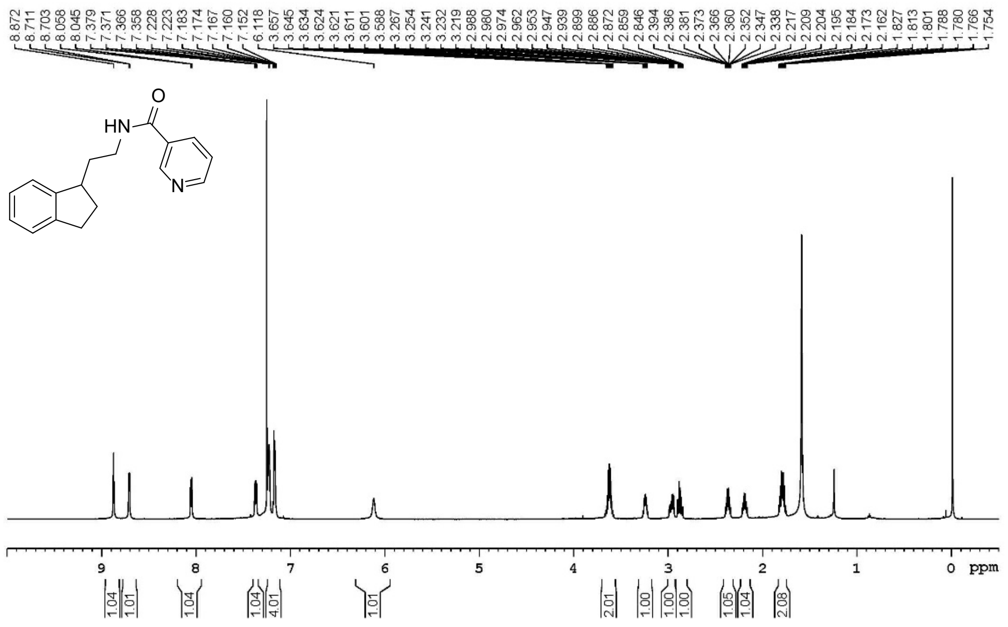

Supplementary Figure 186. ^{13}C NMR spectra of compound **44** (150 MHz, $CDCl_3$)

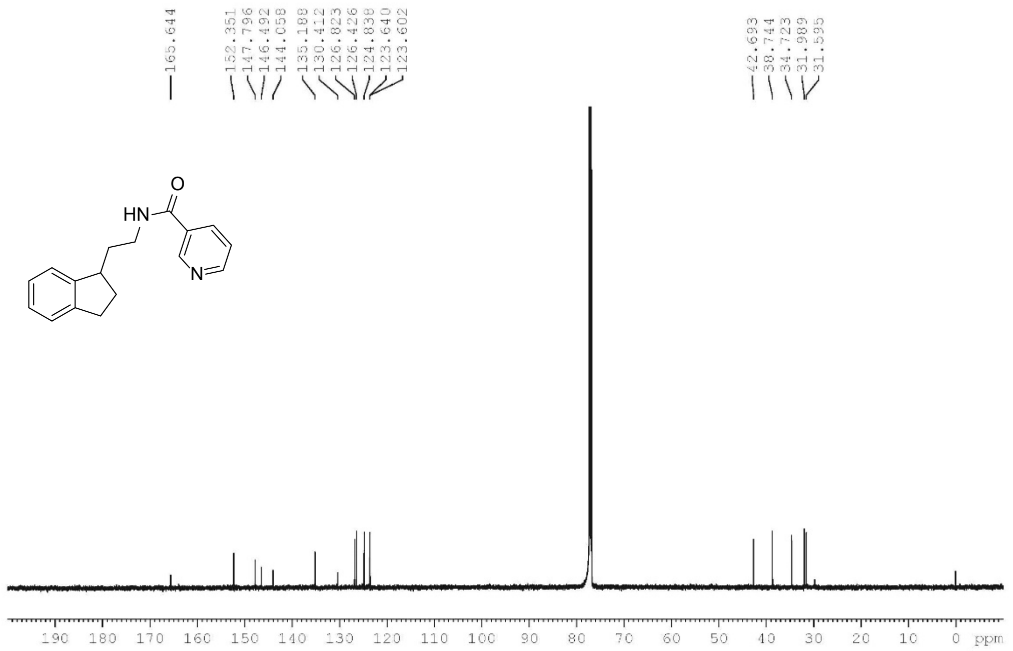

Supplementary Figure 187. ^{1}H NMR spectra of compound **45** (400 MHz, $CDCl_3$)

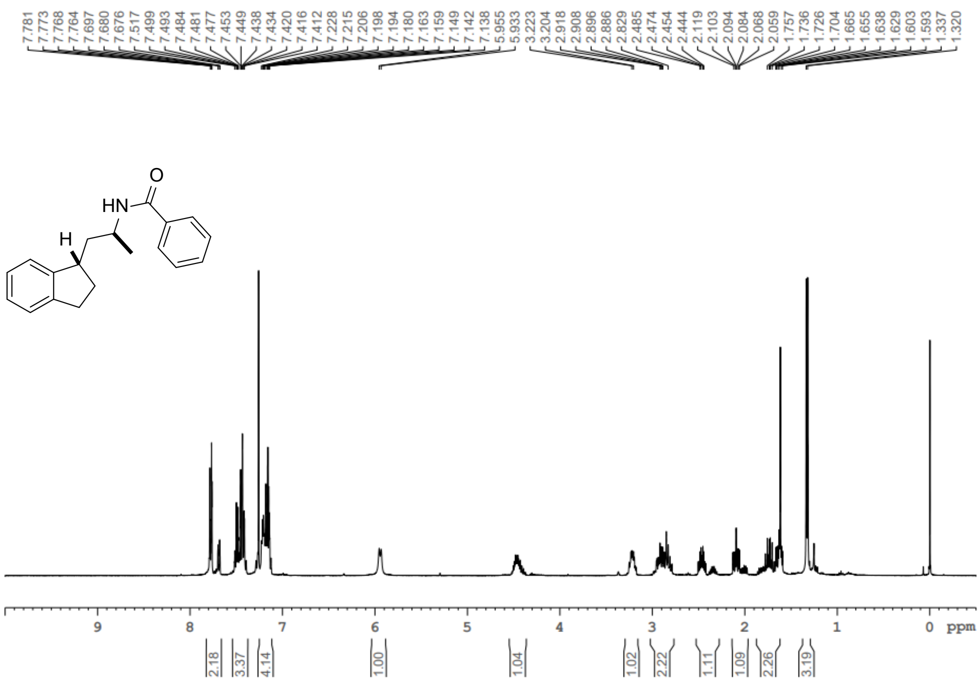

Supplementary Figure 188. ^{13}C NMR spectra of compound **45** (100 MHz, $CDCl_3$)

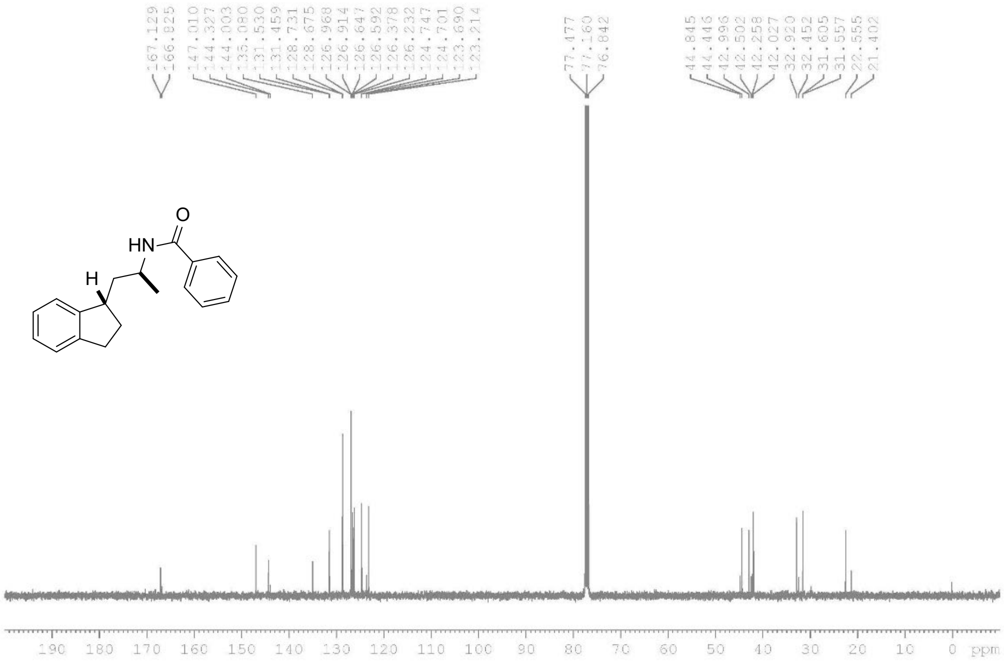

Supplementary Figure 189. ^{1}H NMR spectra of compound **46** (400 MHz, $CDCl_3$)

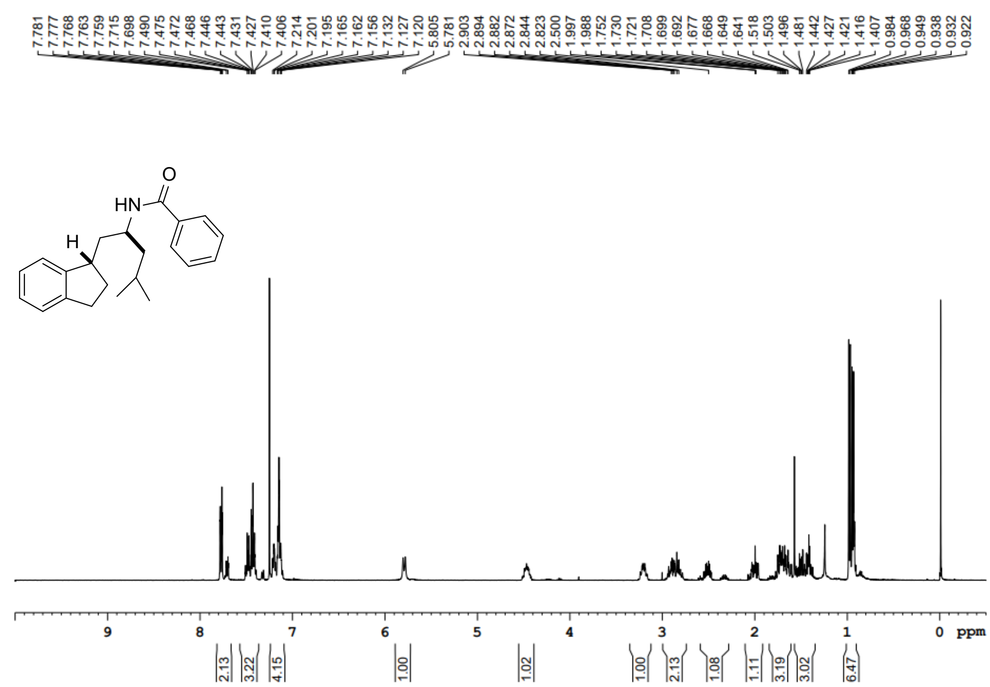

Supplementary Figure 190. ^{13}C NMR spectra of compound **46** (100 MHz, $CDCl_3$)

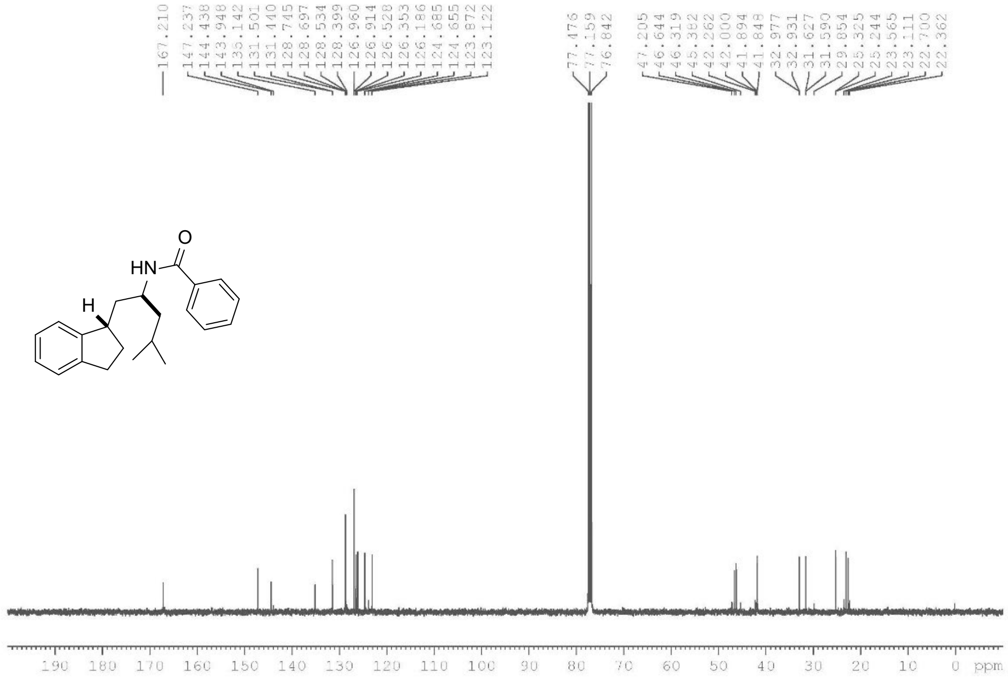

Supplementary Figure 191. ^{1}H NMR spectra of compound **47** (400 MHz, $CDCl_3$)

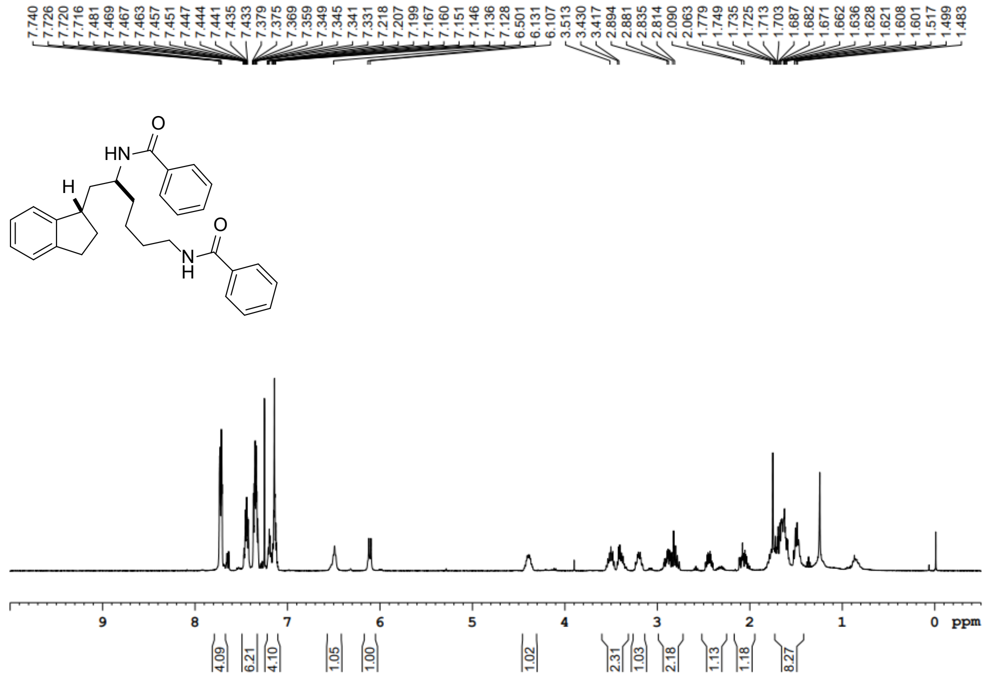

Supplementary Figure 192. ^{13}C NMR spectra of compound **47** (100 MHz, $CDCl_3$)

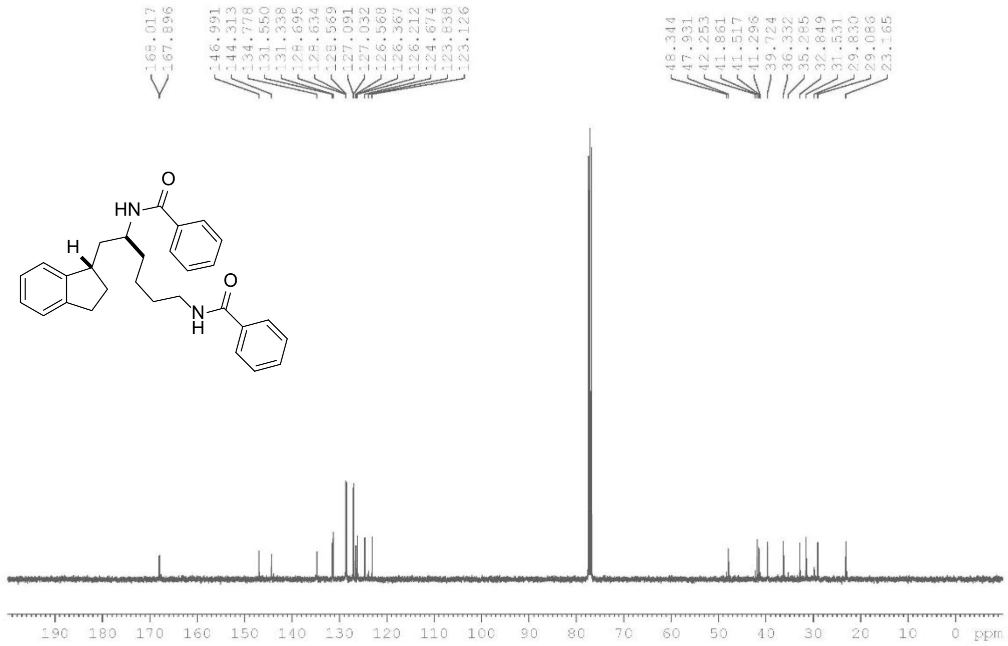

Supplementary Figure 193. ^{1}H NMR spectra of compound **48** (600 MHz, $CDCl_3$)

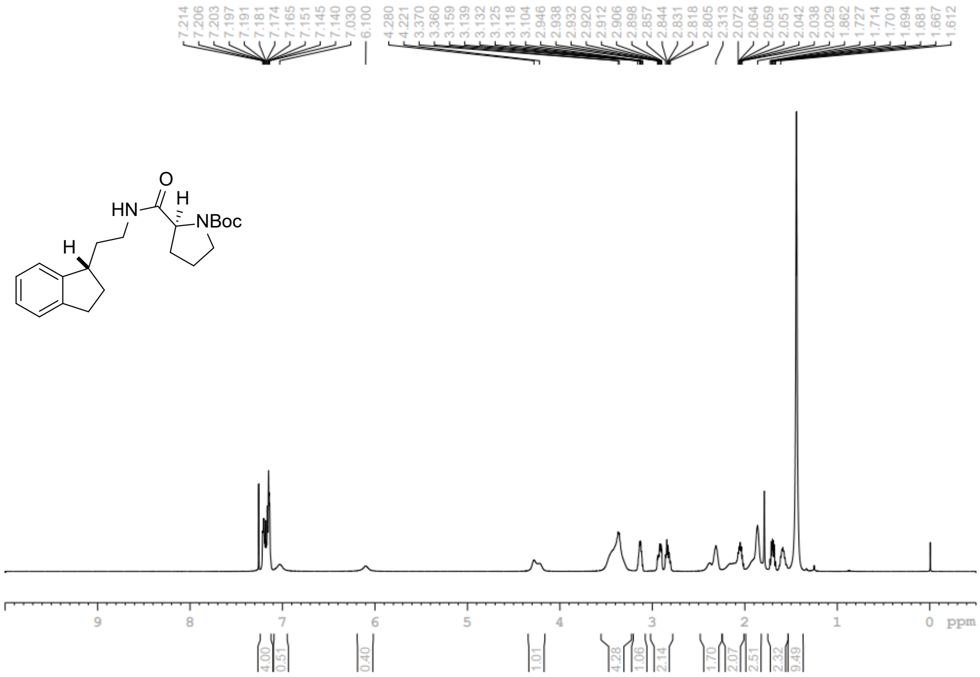

Supplementary Figure 194. ^{13}C NMR spectra of compound **48** (150 MHz, $CDCl_3$)

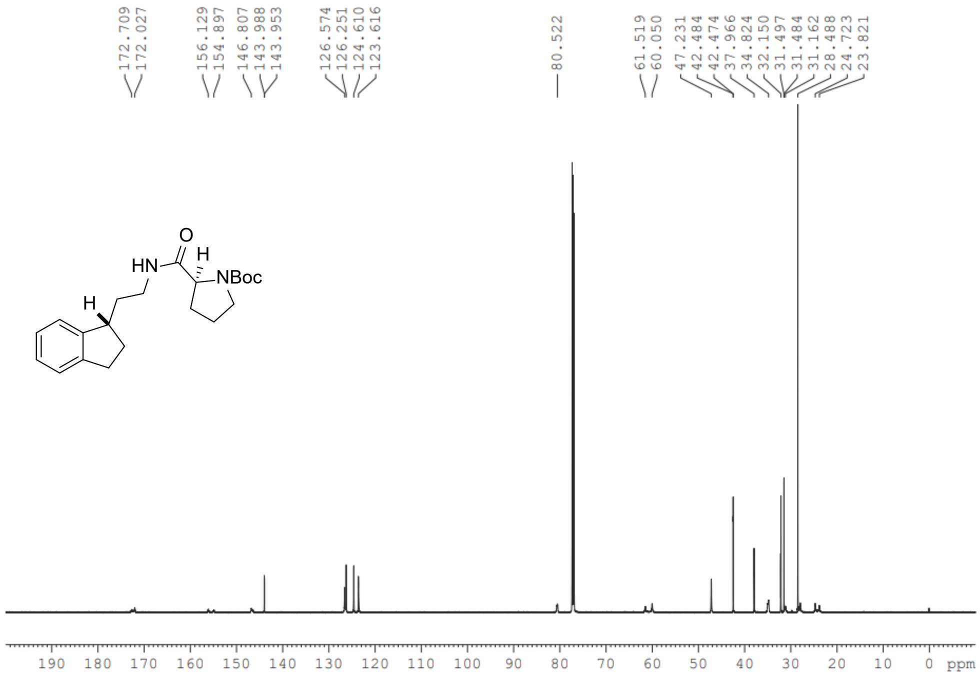

Supplementary Figure 195. ^{1}H NMR spectra of compound **49** (400 MHz, $CDCl_3$)

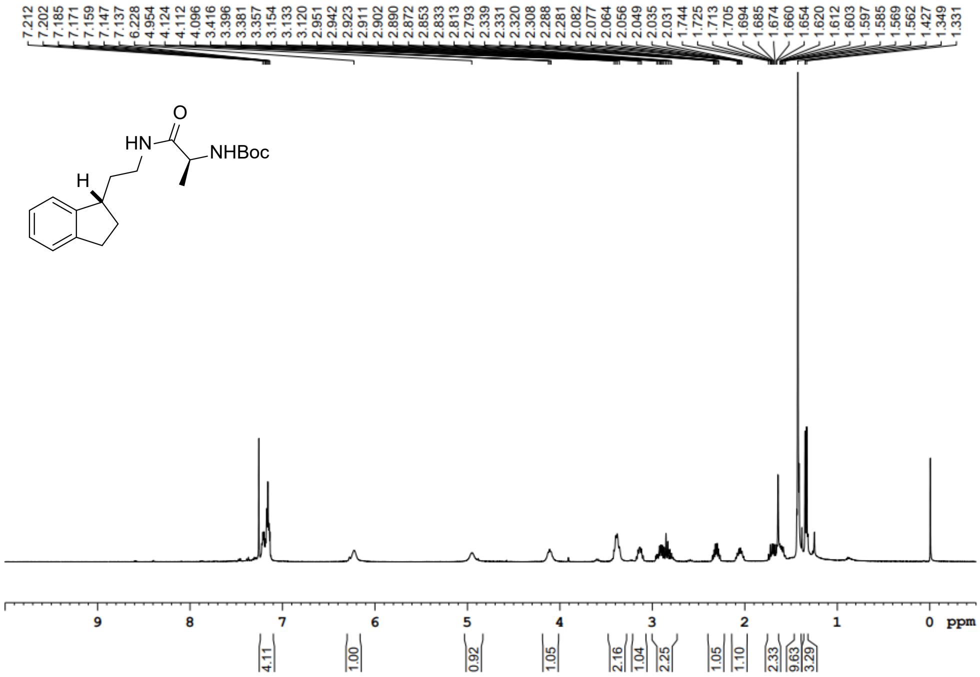

Supplementary Figure 196. ^{13}C NMR spectra of compound **49** (100 MHz, $CDCl_3$)

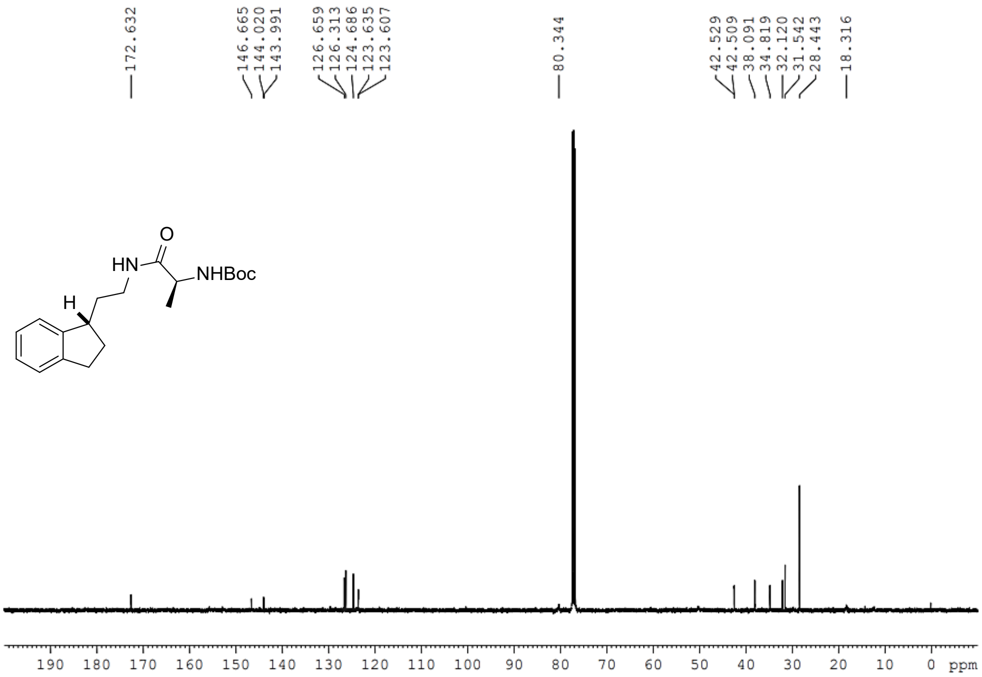

Supplementary Figure 197. ^{1}H NMR spectra of compound **50** (400 MHz, $CDCl_3$)

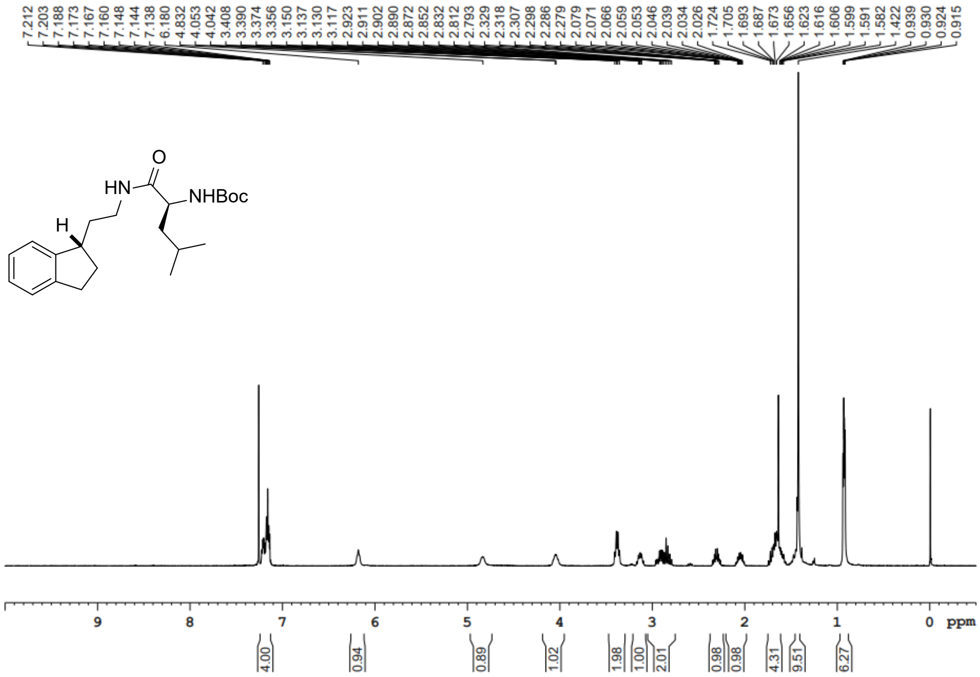

Supplementary Figure 198. ^{13}C NMR spectra of compound **50** (100 MHz, $CDCl_3$)

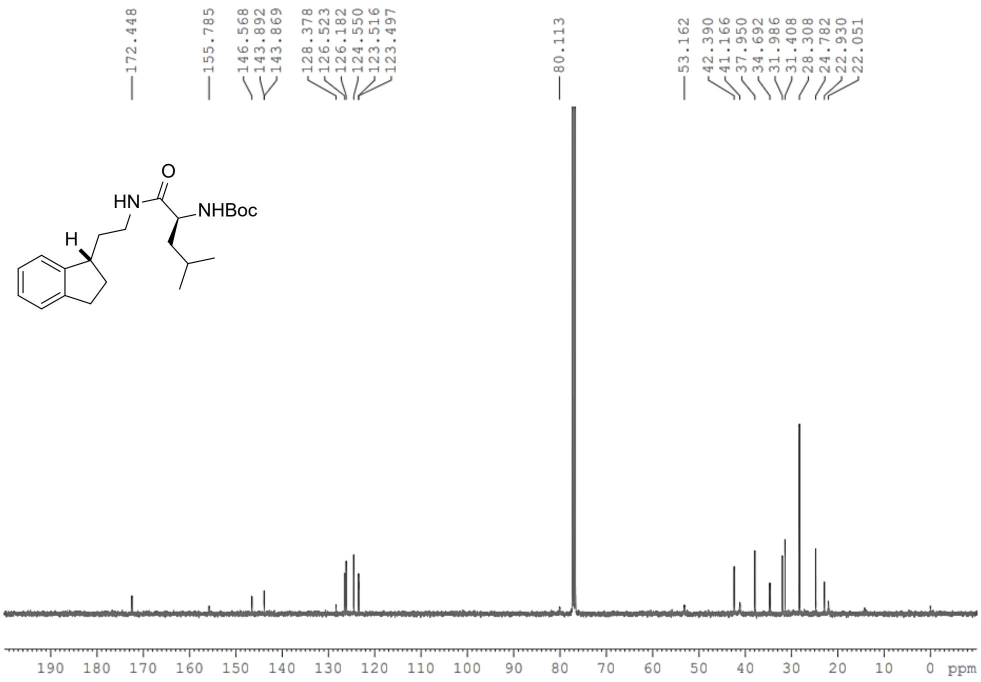

Supplementary Figure 199. ^{1}H NMR spectra of compound **51** (400 MHz, $CDCl_3$)

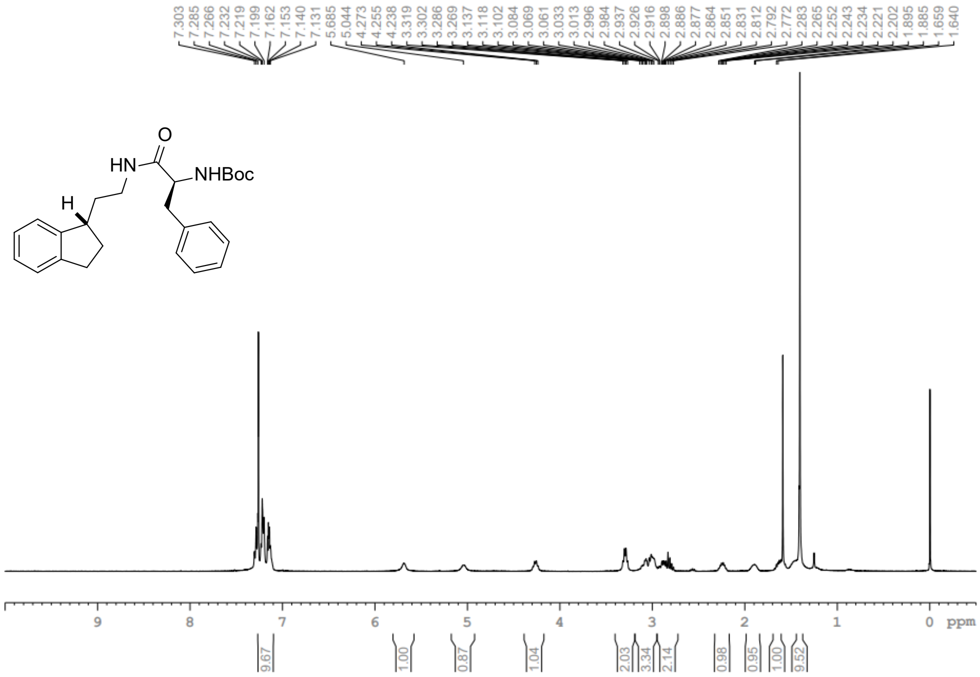

Supplementary Figure 200. ^{13}C NMR spectra of compound **51** (100 MHz, $CDCl_3$)

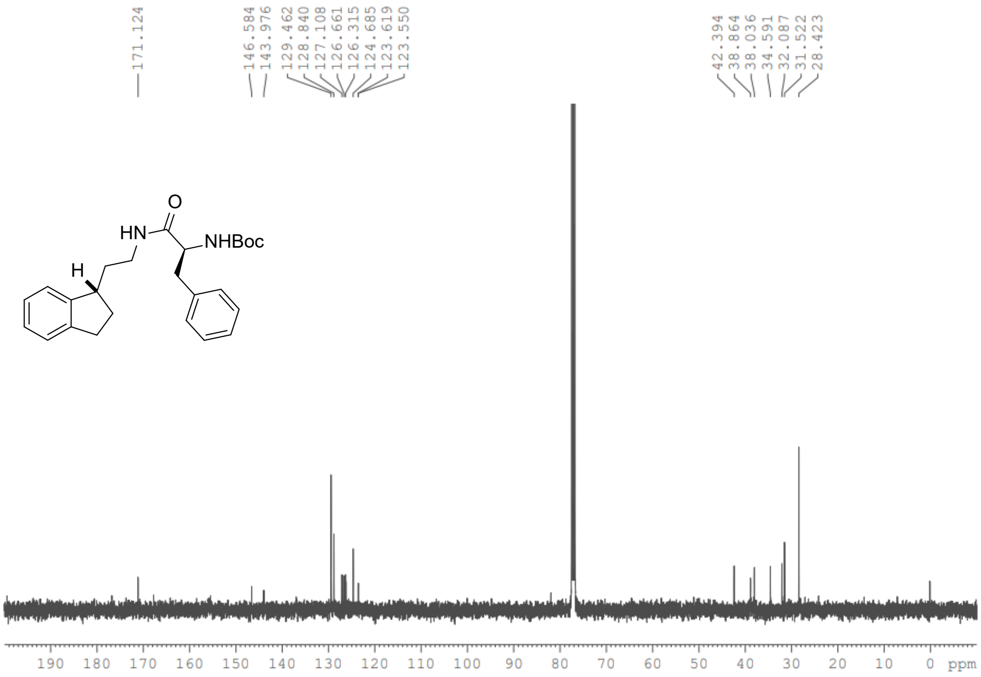

Supplementary Figure 201. ^{1}H NMR spectra of compound **52** (400 MHz, $CDCl_3$)

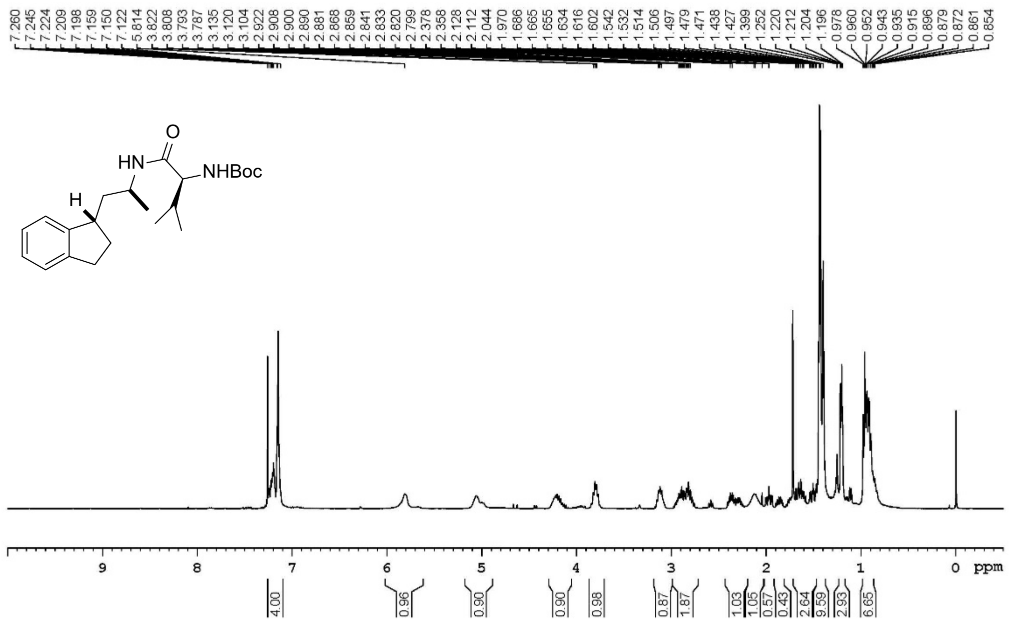

Supplementary Figure 202. ^{13}C NMR spectra of compound **52** (100 MHz, $CDCl_3$)

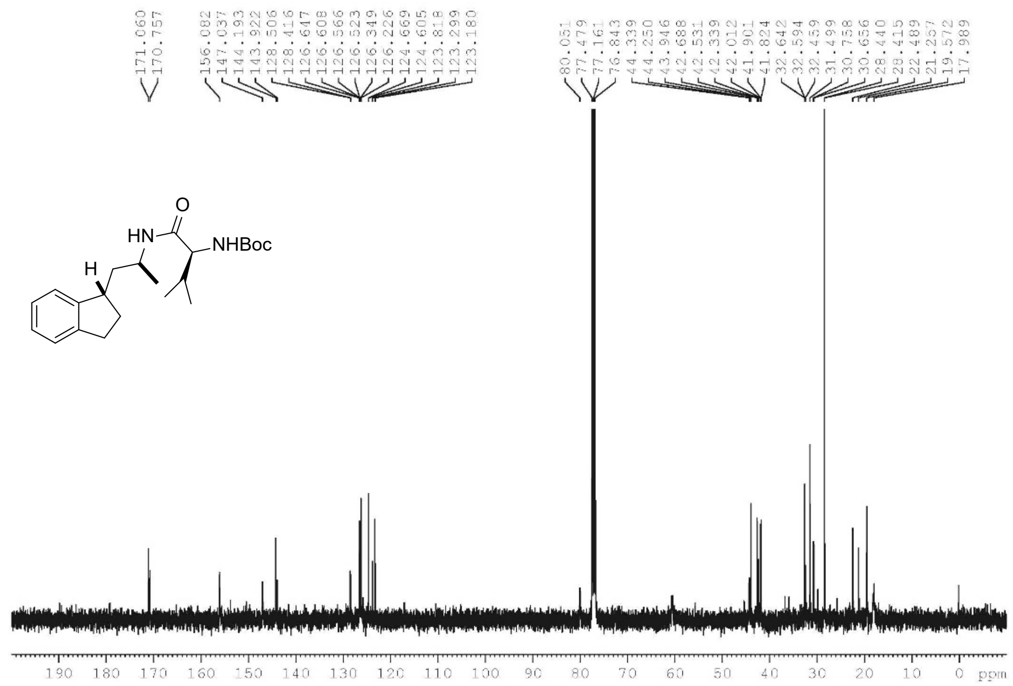

Supplementary Figure 203. ^{1}H NMR spectra of compound **53** (400 MHz, $CDCl_3$)

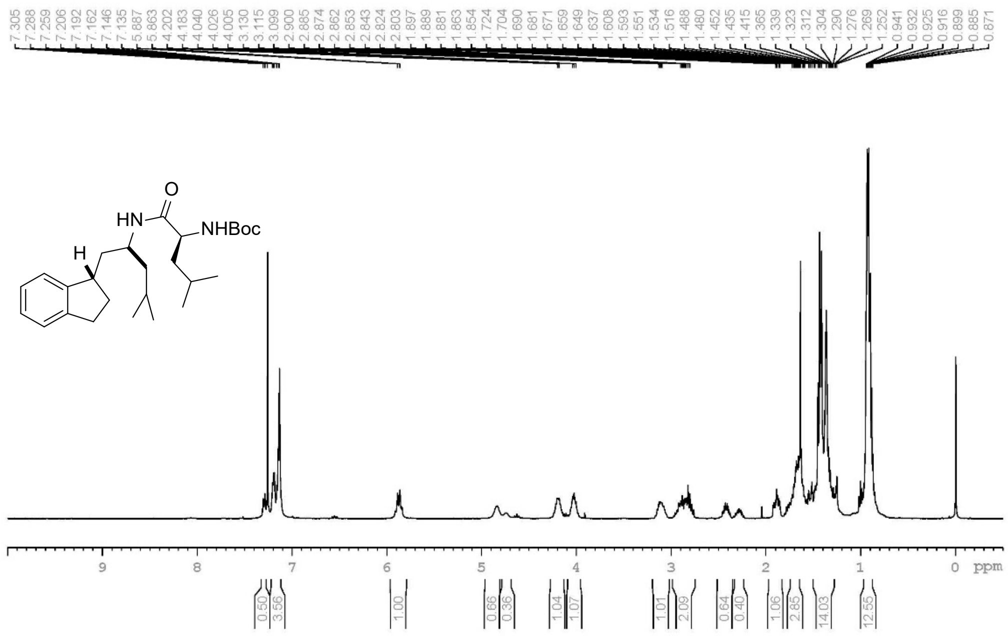

Supplementary Figure 204. ^{13}C NMR spectra of compound **53** (100 MHz, $CDCl_3$)

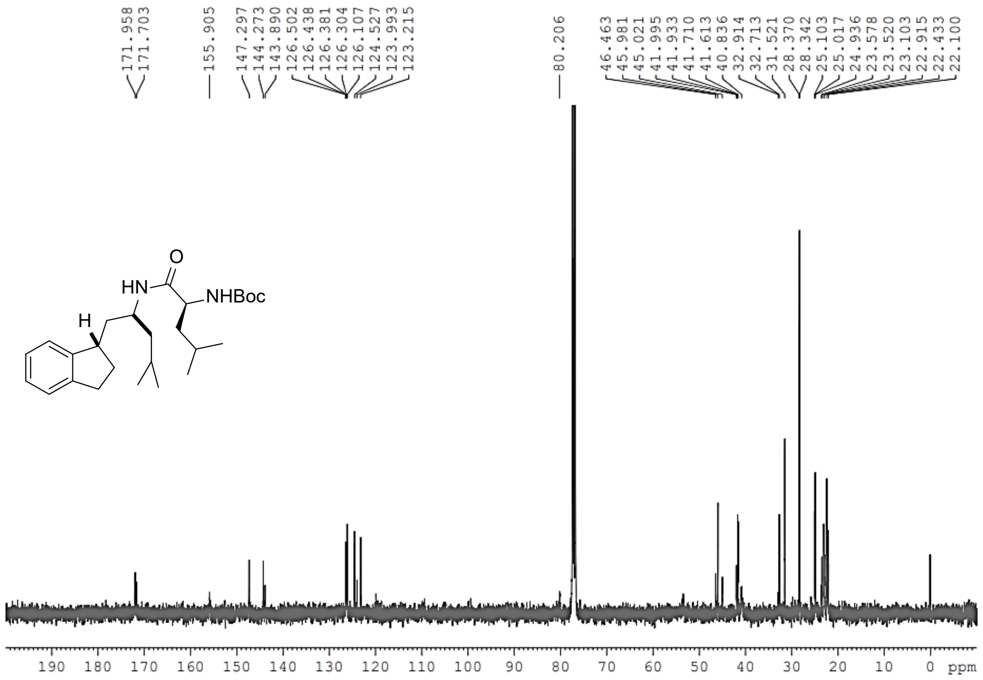

Supplementary Figure 205. ^{1}H NMR spectra of compound **54** (400 MHz, $CDCl_3$)

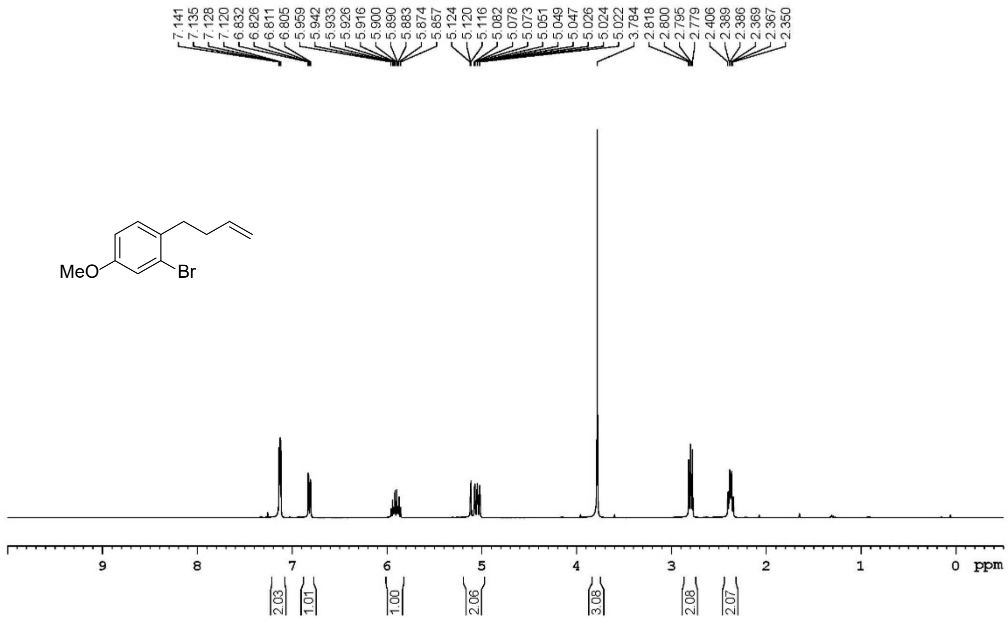

Supplementary Figure 206. ^{13}C NMR spectra of compound **54** (100 MHz, $CDCl_3$)

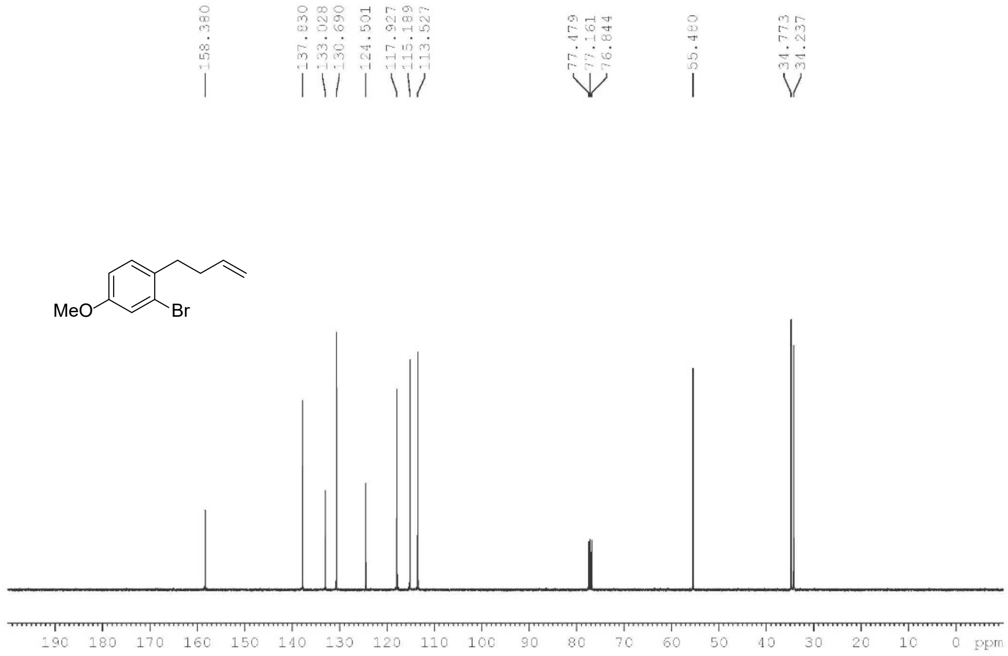

Supplementary Figure 207. ^{1}H NMR spectra of compound **55** (400 MHz, $CDCl_3$)

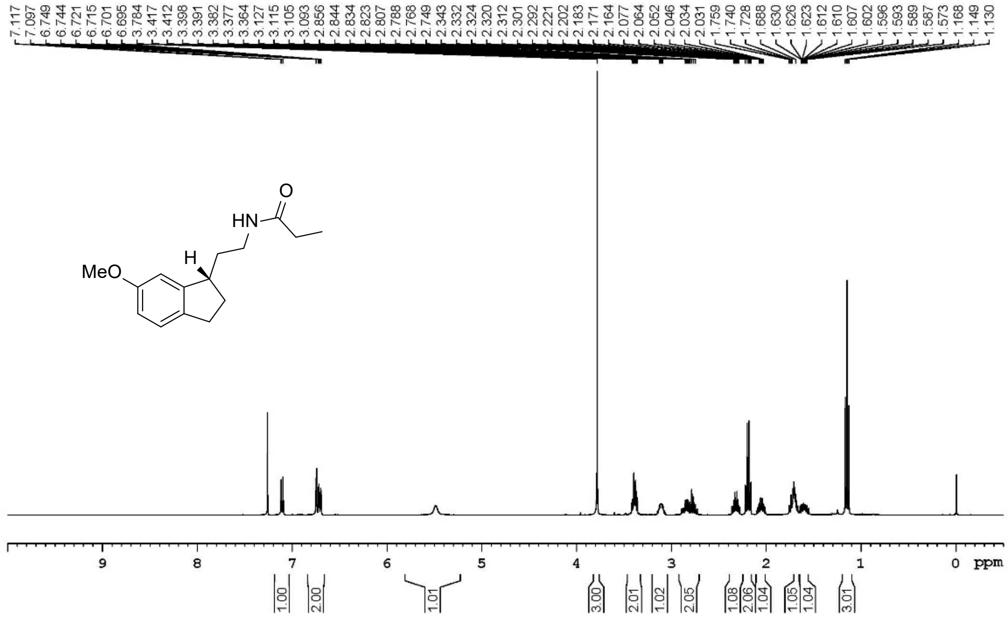

Supplementary Figure 208. NMR spectra of compound **55** (100 MHz, $CDCl_3$)

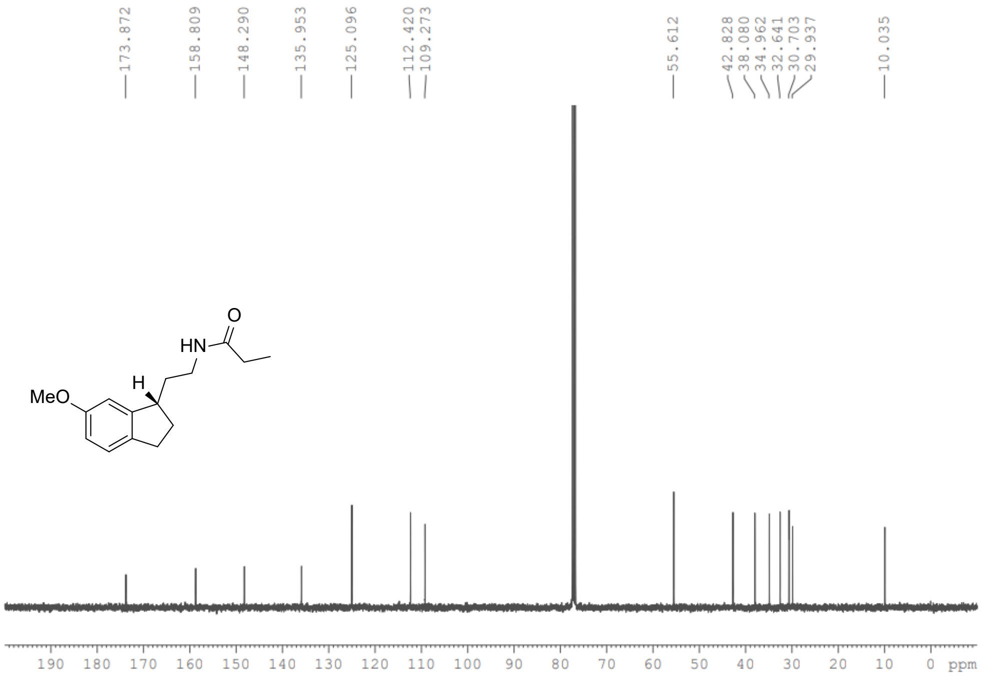

Supplementary Figure 209. ^{1}H NMR spectra of compound **56** (400 MHz, $CDCl_3$)

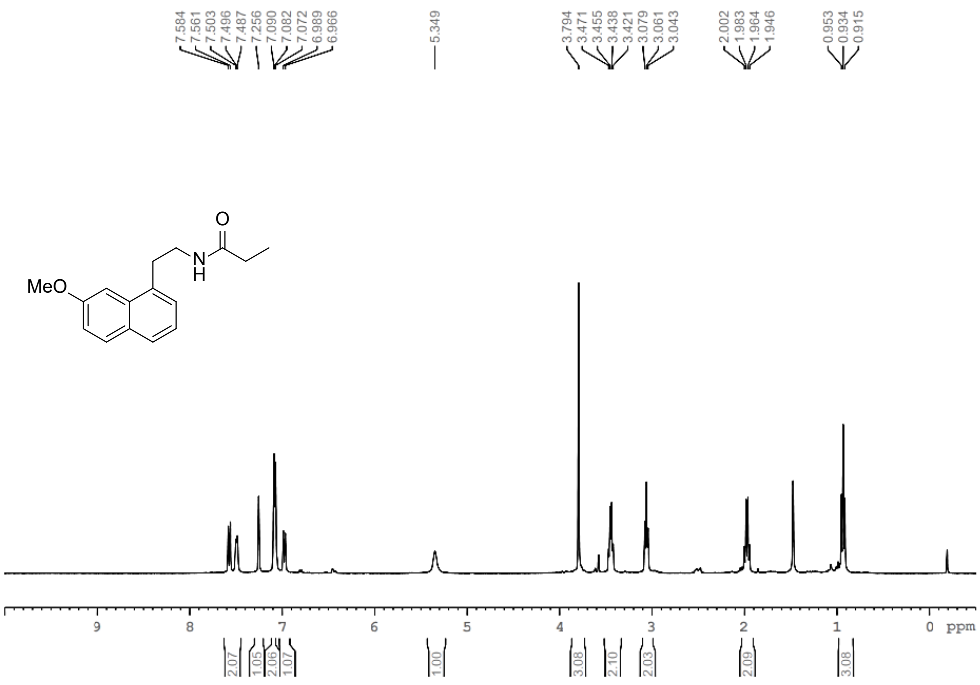

Supplementary Figure 210. ^{13}C NMR spectra of compound **56** (100 MHz, $CDCl_3$)

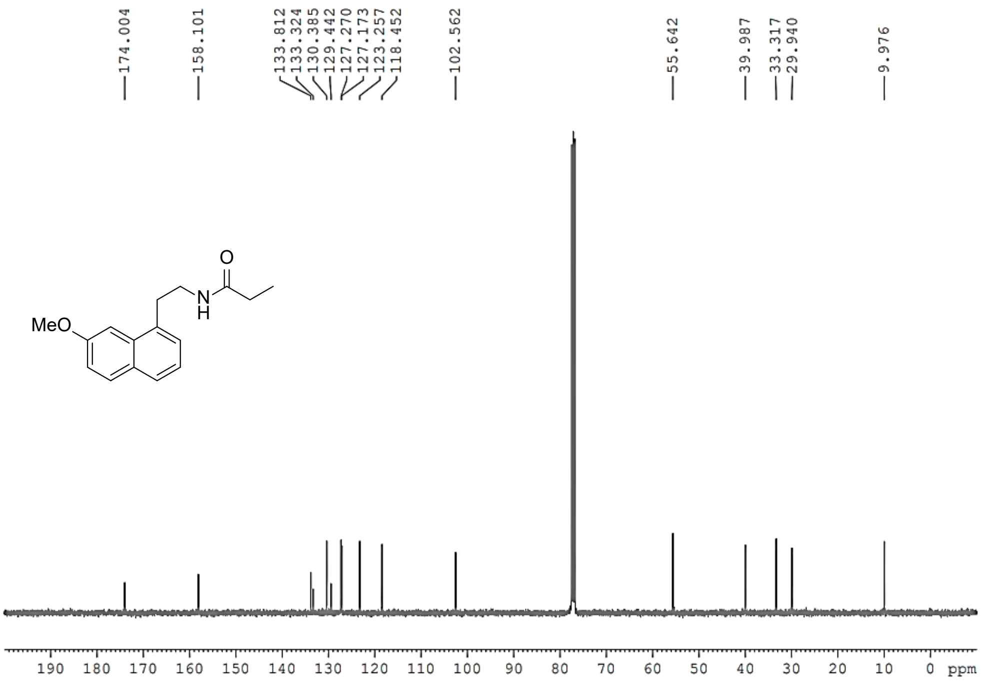

Supplementary Figure 211. ^{1}H NMR spectra of compound **60** (400 MHz, $CDCl_3$)

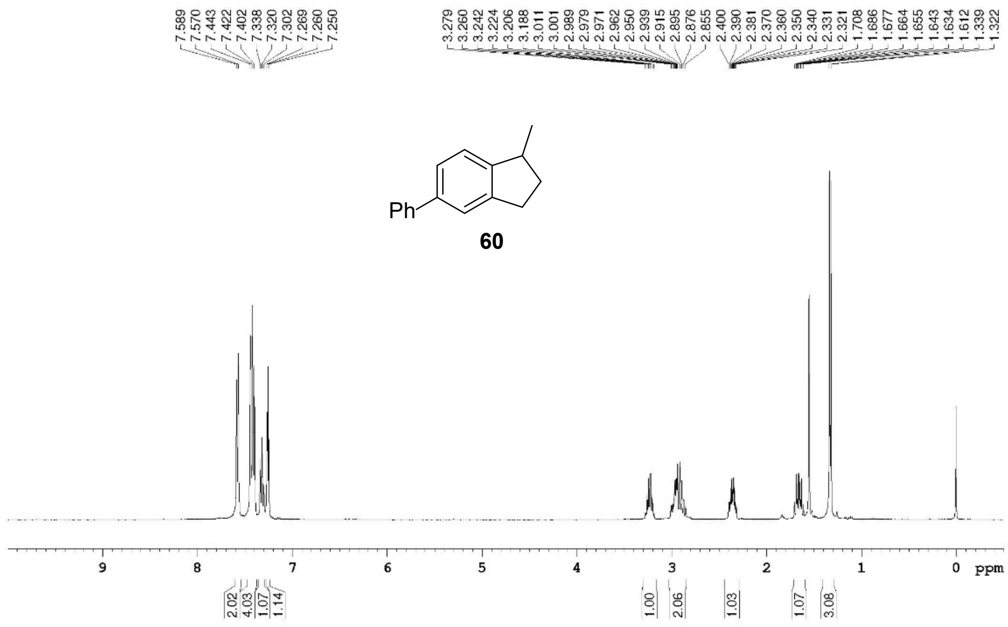

Supplementary Figure 212. ^{13}C NMR spectra of compound **60** (100 MHz, $CDCl_3$)

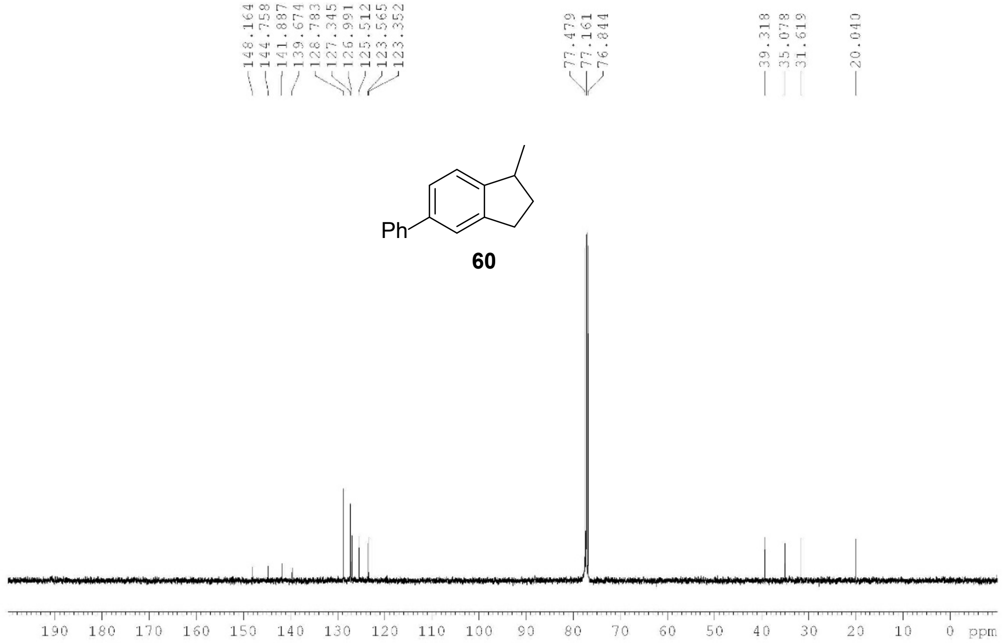

Supplement: Supplementary file 1 — Supplementary Information [file 41467_2023_43748_MOESM1_ESM.pdf]
